# Design, Synthesis, and Biological Evaluation of 5′,7-Disubstituted 7-Deaza-adenosine Analogues as Irreversible Pan-FGFR Inhibitors

**DOI:** 10.3390/ph18111745

**Published:** 2025-11-17

**Authors:** Jung Hoon Park, Phuong Thao Tran, Hye Lin Ko, Seonghee Mun, Sung Chul Jang, Dong Hyun Moon, Jaeho Han, Jieun Kim, Gibae Kim, Hongseok Choi, Seung Woo Kim, Minjae Kim, Sang Kook Lee, Byung Woo Han, Keon Wook Kang, Lak Shin Jeong

**Affiliations:** 1 Republic of Korea Research Institute of Pharmaceutical Sciences, College of Pharmacy, Seoul National University, Seoul 08826, Republic of Korea; parkjhking@snu.ac.kr (J.H.P.); 202133296@snu.ac.kr (P.T.T.); hyelinko@snu.ac.kr (H.L.K.); seongheemun@snu.ac.kr (S.M.); tobok95@snu.ac.kr (S.C.J.); moondh@snu.ac.kr (D.H.M.); gh03292@snu.ac.kr (J.H.); jieun0821@snu.ac.kr (J.K.); gibae.kim@austin.utexas.edu (G.K.); choihs906@snu.ac.kr (H.C.); kung1000@snu.ac.kr (S.W.K.); mingae93@snu.ac.kr (M.K.); sklee61@snu.ac.kr (S.K.L.); bwhan@snu.ac.kr (B.W.H.); kwkang@snu.ac.kr (K.W.K.); 2 ^2^ Research Institute of Natural Products Research Institute, College of Pharmacy, Seoul National University, Seoul 08826, Republic of Korea; 3 Future Medicine Co., Ltd., 54 Changup-ro, Sujeong-gu, Seongnam 13449, Republic of Korea

**Keywords:** pan-FGFR inhibitors, covalent inhibitors, nucleoside analogues, X-ray crystallography, molecular dynamics simulations, metabolic stability

## Abstract

**Background/Objectives**: Fibroblast growth factor receptors (FGFRs) are frequently dysregulated in diverse cancers and represent important therapeutic targets. Here, we report the design and synthesis of a novel nucleoside-based scaffold which enables irreversible pan-FGFR inhibition as a potential anticancer strategy. **Methods**: A series of nucleoside analogues was synthesized and assessed through structure–activity relationship studies. Structural analyses, including X-ray co-crystallography and molecular dynamics simulations, were performed to define key determinants of potency and selectivity. Biochemical assays against FGFR1–4 proteins, cellular antiproliferative assays in HCT116 (FGFR1 amplification) and RT4 (FGFR3-TACC3) models, metabolic stability evaluations and covalent bonding confirmation were conducted to characterize representative compounds. **Results**: SAR studies revealed that fused aromatic substituents and 4′-thio ribose enhanced FGFR potency, whereas enantiomeric inversion of ribose reduced activity. X-ray co-crystallography further demonstrated that two hydroxyl groups form a key water-mediated hydrogen bond network, uniquely stabilizing the ligand and enhancing potency of inhibitors compared to reference compounds. The 7-methoxy-5-methylbenzo[*b*]thiophene scaffold and ribose moiety emerged as critical features. Compounds **13f**, **19e**, and **22f** demonstrated potent inhibition of FGFR1-4 and dose-dependent suppression of FGFR1-mediated signaling, with strong antiproliferative activity in both FGFR-driven and wild-type cancer models. Compound **22f** showed efficient irreversible covalent engagement of FGFRs, confirmed at the protein and cellular levels, and exhibited improved metabolic stability. **Conclusions**: Nucleoside analogues represent a privileged scaffold for covalent pan-FGFR inhibition. The findings highlight their potential as promising therapeutic candidates for targeting FGFR-driven malignancies. Future efforts will focus on further improving stability and optimizing physicochemical properties to advance these compounds toward translational development.

## 1. Introduction

The fibroblast growth factor receptor (FGFR) family comprises four receptor tyrosine kinases, FGFR1 through FGFR4, each containing three extracellular immunoglobulin (Ig)-like binding domains, a transmembrane domain, and an intracellular domain comprising a two-part tyrosine kinase [1]. Among the 22 known fibroblast growth factor (FGF) ligands, 18 different FGFs are capable of binding and activating these receptors by inducing receptor dimerization [2,3,4,5]. Upon ligand binding and dimerization, FGFRs undergo autophosphorylation, triggering a cascade of downstream signaling pathways. These include the Ras/MEK/ERK and PI3K/AKT pathways, which regulate key cellular processes such as proliferation, differentiation, angiogenesis, and survival [6]. Genomic alterations in FGFRs, including amplifications, mutations, and gene fusions, were found in 7.1% of all cancers. FGFR1 alterations are the most prevalent (49% of the aberrations), followed by FGFR3 (26%), FGFR2 (19%), and FGFR4 (7%) [2]. FGFR1 amplification is particularly common, occurring in approximately 20% of non-small cell lung cancers (NSCLC) [1,2] and 7% of urothelial carcinomas, leading to protein overexpression and oncogenic signaling dependency [7]. FGFR2 fusions are found in 10–20% of intrahepatic cholangiocarcinomas [8,9], while FGFR3 fusions are especially prevalent in urothelial carcinomas and glioblastomas. Notably, FGFR3–TACC3 (Transforming Acidic Coiled-Coil containing protein 3) is the most frequently reported fusion aberration, likely due to the close genomic proximity of FGFR3 and TACC3 on chromosome 4p16. This fusion mediates microtubule binding and induces ligand-independent activation of the MAPK pathway, thereby promoting bladder cancer and glioblastoma [2]. Additionally, FGFR4 mutations have been identified in 7–8% of rhabdomyosarcomas [10].

**Figure 1:**
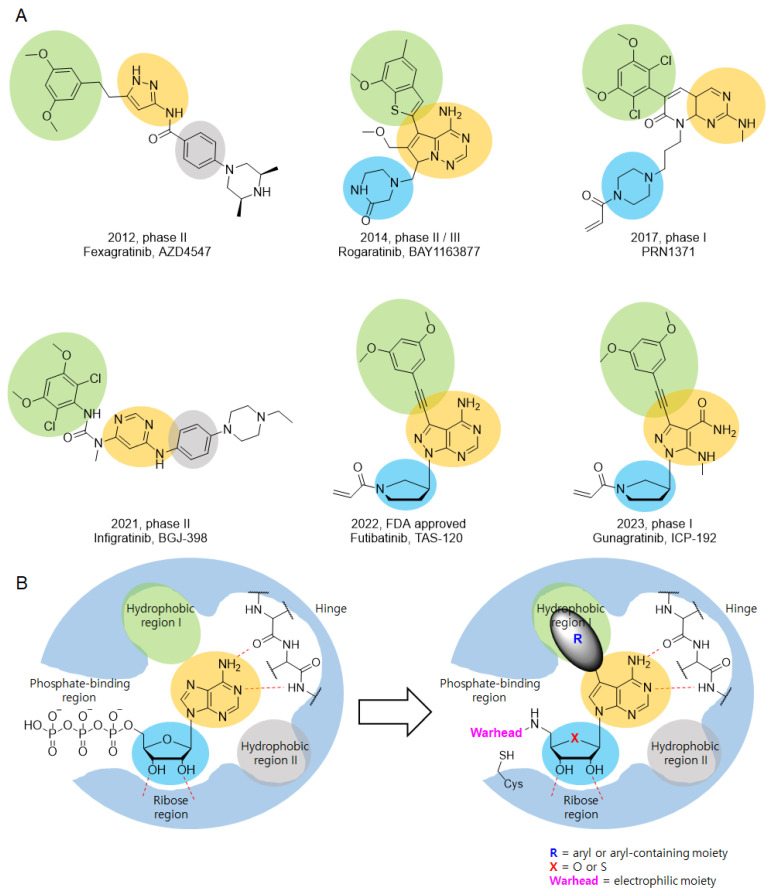
Structural basis of the FGFR and FGFR inhibitors with design rationale. (**A**) Chemical structure of selective FGFR inhibitors. (**B**) Five regions of the ATP binding site and design rationale of nucleoside-based inhibitors. Hydrogen bond (red dashed line).

First-generation FGFR inhibitors, such as ponatinib [11], dovitinib [12], lucitanib [13], and nintedanib [14], were developed as multitargeted tyrosine kinase inhibitors (TKIs). While some clinical benefits were observed in early-phase trials, their lack of kinase selectivity for FGFRs over other kinases led to off-target toxicities and limited clinical utility [15], including soft tissue mineralization and hyperphosphatemia [16]. These adverse events hindered their clinical utility, prompting the development of more selective second-generation FGFR TKIs, such as fexagratinib [17], rogaratinib [18], PRN1371 [19], infigratinib [20], futibatinib [21], and gunagratinib [22], which were rationally designed to achieve high potency and selectivity for FGFRs over other kinases (Figure 1A). These agents have demonstrated improved safety profiles and clinical responses in FGFR-driven tumors. Nevertheless, even with enhanced selectivity, isoform-selective FGFR inhibitors are often limited by tumor heterogeneity and the rapid emergence of bypass signaling through untargeted FGFR isoforms [23]. In this regard, pan-FGFR inhibitors that simultaneously target FGFR1–4 ensure a more compelling therapeutic strategy than isoform-selective inhibitors. By providing comprehensive inhibition across the receptor family, pan-FGFR inhibitors effectively address tumor heterogeneity and overcome resistance mechanisms associated with isoform-restricted therapies. Moreover, pan-FGFR inhibition broadens therapeutic coverage across diverse tumor types harboring various FGFR abnormalities, thereby mitigating resistance and maintaining therapeutic efficacy in tumors that co-express or switch between FGFR isoforms. Importantly, these advantages are achieved while minimizing off-target toxicities inherent to earlier-generation multitargeted TKIs, potentially improving clinical outcomes.

Recent structural studies of selective FGFR inhibitors have highlighted key pharmacophores essential for potent and selective binding (Figure 1). Engagement of hydrophobic pocket I (green) and hydrogen bonding with the hinge region (orange) represent common strategies to enhance affinity and selectivity, while certain inhibitors such as fexagratinib improve potency by extending into hydrophobic pocket II (gray). Unlike hydrophobic pocket II, the ribose pocket (blue) is characterized by the presence of water molecules and polar residues capable of forming hydrogen bonds, thus providing opportunities for additional stabilizing interactions. However, conventional FGFR inhibitors such as futibatinib or gunagratinib contain predominantly hydrophobic moieties and therefore cannot establish hydrogen bonds in this region. In contrast, nucleoside-based scaffolds can effectively engage the ribose pocket through their hydroxyl groups in a manner similar to the natural ligand ATP, forming additional hydrogen bonds (Figure 1B) [24] that enhance potency and prolong the target engagement, thereby conferring a unique advantage over conventional FGFR inhibitors. In addition to oxo-nucleosides, 4′-thioadenosine derivatives have also been reported to exhibit strong kinase inhibitory effects [25], further supporting nucleosides as privileged scaffolds for kinase inhibition. Therefore, in this study, we synthesized not only the oxo-nucleoside series but also 4′-thioadenosine analogues to directly compare their inhibitory properties.

Notably, futibatinib incorporates an acrylamide electrophilic warhead that covalently binds to a conserved cysteine residue within the FGFR active site. This irreversible mode of inhibition enables sustained kinase inactivation and may help overcome resistance mechanisms, such as transient target occupancy or gatekeeper mutations, which limit the efficacy of reversible inhibitors.

Guided by these structural insights, we designed a novel class of nucleoside-based irreversible FGFR inhibitors (Figure 1B). These compounds were engineered to (i) effectively occupy hydrophobic pocket I and the hinge region, (ii) establish unique ribose pocket hydrogen bonds via the nucleoside ribose moiety, and (iii) incorporate an electrophilic warhead to covalently target the conserved cysteine residue, thereby enhancing the duration of inhibition. This rational design strategy aimed to synergize reversible high-affinity interactions with irreversible covalent engagement, highlighting nucleoside analogues as a privileged scaffold for potent and durable pan-FGFR inhibition.

## 2. Results and Discussion

### 2.1. Chemistry

**Scheme 1:**
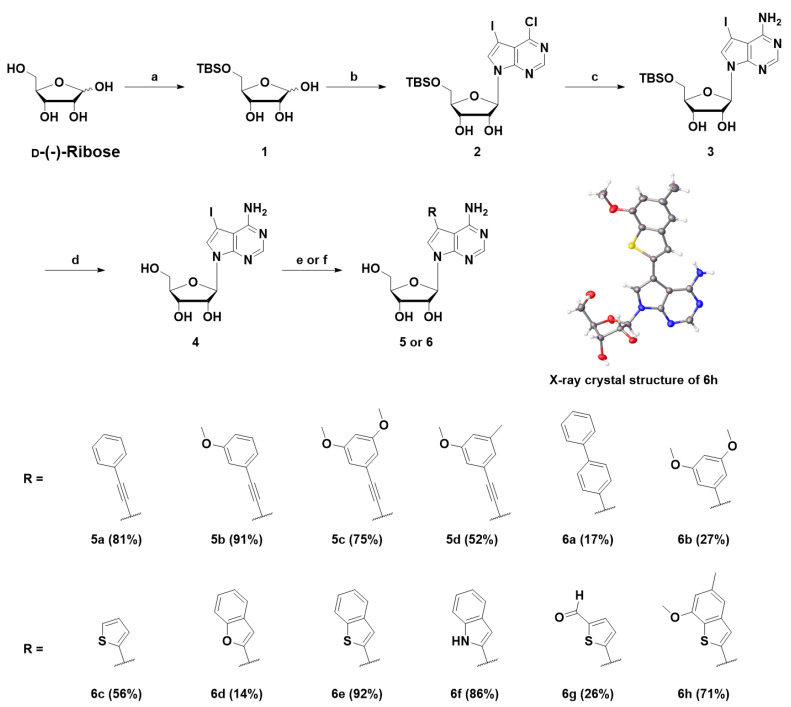
Synthesis of 7-substituted-7-deaza-adenosine derivatives **5a**–**d, 6a**–**h**. Reagents and conditions: (a) TBSCl, pyridine, 0 °C to rt, 17 h, 39%; (b) 6-Chloro-7-iodo-7-deazapurine, DBU, DIAD, PEt_3_, ACN, 0 °C to rt, 17 h, 33%; (c) NH_3_/*tert*-BuOH, 90 °C, 17 h, 81%; (d) 50% HCO_2_H/H_2_O, THF, rt, 24 h, 76%; (e) alkynes, PdCl_2_(PPh_3_)_2_, CuI, Et_3_N, DMF, MW, 50 °C, 1 h, 75–91% for **5a**–**c** or ((3-methoxy-5-methylphenyl)ethynyl)trimethylsilane, PdCl_2_(PPh_3_)_2_, CuI, CsF, Et_3_N, DMF, MW, 50 °C, 1 h, 52% for **5d**; (f) boronic acids or boronic esters, Pd(PPh_3_)_4_, Na_2_CO_3_, DMF/H_2_O, MW, 70 °C, 2 h, 14–92% for **6a**–**h**.

The synthetic route of the 7-substituted-7-deaza-adenosine derivatives is shown in Scheme 1. The TBS-protected glycosyl donor **1** was prepared from commercially available D-(–)-ribose, which provided the nucleoside scaffold **2** through a Mitsunobu condensation. The 6-amino compound **3** was obtained from the 6-chloro compound **2** by S_N_Ar with ammonia in *t*-BuOH, followed by deprotection of the TBS group with 50% formic acid to furnish compound **4.** Compounds **5a**–**d** were obtained from intermediate **4** through Sonogashira cross-coupling with alkynes, while compounds **6a**–**h** were derived from the same intermediate via Suzuki cross-coupling with aryl boronic acids. The structure of **6h** was confirmed by X-ray crystallography (Figure S1, Table S1).

**Scheme 2:**
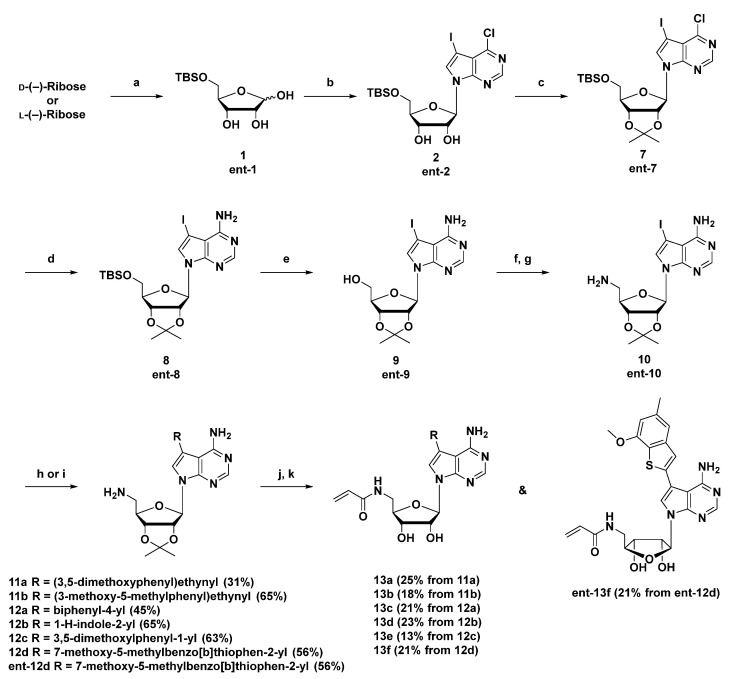
Synthesis of 5′-acrylamide-7-substituted-7-deaza-adenosine derivatives **13a**–**f**, ent-**13f.** Reagents and conditions: (a) TBSCl, pyridine, 0 °C to rt, 17 h, 39%; (b) 6-Chloro-7-iodo-7-deazapurine, DBU, DIAD, PEt_3_, ACN, 0 °C to rt, 17 h, 33%; (c) 2,2-Dimethoxypropane, *p*-toluenesulfonic acid, CH_2_Cl_2_, rt, 6 h, 72%; (d) NH_3_/tert-BuOH, 90 °C, 17 h, 94%; (e) 1M TBAF, THF, rt, 40 min, 86%; (f) PPh_3_, DIAD, DPPA, THF, 0 °C to rt, 17 h; (g) (i) PPh_3_, THF, rt, 17 h, (ii) NH_4_OH, THF, 65 °C, 2 h, 62%; (h) alkynes, PdCl_2_(PPh_3_)_2_, CuI, Et_3_N, DMF, MW, 50 °C, 1 h, 31–65%; (i) boronic acids or boronic esters, Pd(PPh_3_)_4_, Na_2_CO_3_, DMF/H_2_O, MW, 70 °C, 2 h, 45–65%; (j) acrylic acid, EDC·HCl, DMAP, DMF, 0 °C, 2 h; (k) 50% HCO_2_H/H_2_O, THF, rt, 24 h, 18–25%.

The synthetic route of the 5′-acrylamide-7-substituted-7-deaza-adenosine derivatives is shown in Scheme 2. Acetonide protection using 2,2-dimethoxypropane yielded compound **7**. Amino compound **8** was obtained from **7** through an S_N_Ar with ammonia in *t*-BuOH, followed by TBS deprotection with 1 M TBAF to furnish compound **9**. The key amino intermediate **10** was obtained from the 5′-hydroxyl compound **9** through the Mitsunobu reaction to introduce an azide, followed by the Staudinger reaction. Various alkyne and aryl moieties were introduced via Sonogashira and Suzuki cross-coupling reactions to provide compounds **11a**–**b**, **12a**–**d**. To incorporate an acrylamide warhead at the 5′-amine, EDC-mediated coupling with acrylic acid was performed, followed by deprotection of the acetonide group to give the final compounds **13a**–**f**. To investigate the effect of enantiomeric configuration on FGFR inhibition, the compounds derived from L-(–)-ribose were labeled with the “ent-” prefix to indicate their enantiomeric relationship to the D-(–)-ribose-derived counterparts. Compound ent-**13f** was synthesized through the same sequence of reactions, and the structure of ent-**7** was confirmed by X-ray crystallography (Figure S2, Table S2).

**Scheme 3:**
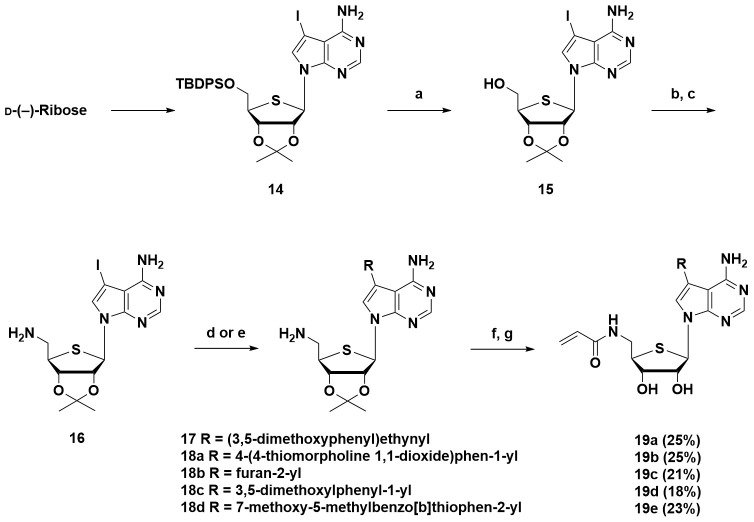
Synthesis of 5′-acrylamide-7-substituted-7-deaza-4′-thioadenosine derivatives **19a**–**e**. Reagents and conditions: (a) 1M TBAF, THF, rt, 40 min, 95%; (b) PPh_3_, DIAD, DPPA, THF, 0 °C to rt, 17 h; (c) (i) PPh_3_, THF, rt, 17 h (ii) NH_4_OH, THF, 65 °C, 2 h, 47%; (d) alkyne, PdCl_2_(PPh_3_)_2_, CuI, Et_3_N, DMF, MW, 50 °C, 1 h; (e) boronic acids or boronic esters, Pd(PPh_3_)_4_, Na_2_CO_3_, DMF/H_2_O, MW, 70 °C, 2 h; (f) acrylic acid, EDC·HCl, DMAP, DMF, 0 °C, 2 h; (g) 50% HCO_2_H/H_2_O, THF, rt, 24 h, 18–25%.

The synthetic route of the 5′-acrylamide-7-substituted-7-deaza-4′-thioadenosine derivatives is shown in Scheme 3. The 4′-thioadenosine **14** was synthesized from D-(–)-Ribose according to previously reported procedures [24]. Subsequent deprotection of the TBDPS using 1 M TBAF afforded compound **15**. The 5′-hydroxyl group was then transformed into an azide via the Mitsunobu reaction, followed by the Staudinger reduction to yield the key 5′-amino intermediate **16**. The compounds **17** and **18a**–**d** were obtained from **16** through the Sonogashira and Suzuki cross-coupling reactions with 1-ethynyl-3,5-dimethoxybenzene and various aryl substituents, respectively. Finally, EDC-mediated coupling with acrylic acid and subsequent acetonide deprotection provided the final compounds **19a**–**e**.

**Scheme 4:**
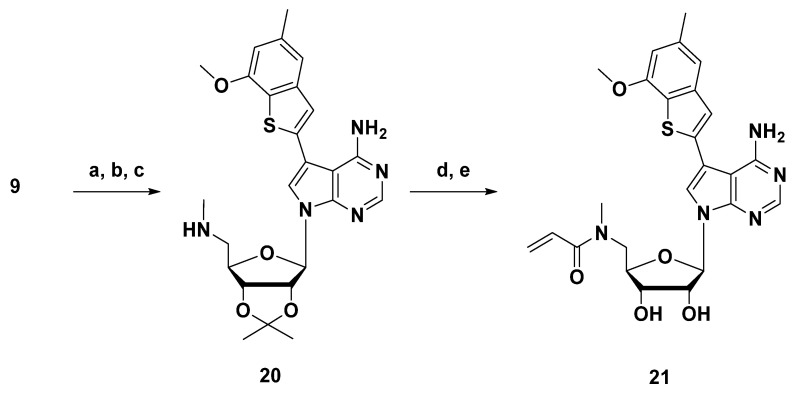
Synthesis of N-methyl-5′-acrylamide-7-substituted-7-deaza-adenosine **21.** Reagents and conditions: (a) MsCl, Et_3_N, CH_2_Cl_2_, 0 °C, 40 min; (b) 2M CH_3_NH_2_ in THF, 75 °C, 17 h; (c) boronic acid, Pd(PPh_3_)_4_, Na_2_CO_3_, DMF/H_2_O, MW, 70 °C, 2 h, 46%; (d) acrylic acid, EDC·HCl, DMAP, DMF, 0 °C, 2 h; (e) 50% HCO_2_H/H_2_O, THF, rt, 24 h, 22%.

To obtain compound **21**, in which a methyl group is introduced to the nitrogen of acrylamide, compound **20** was synthesized from **9** by S_N_2 reaction with the methylamine in a sealed tube, and subsequent Suzuki coupling with 7-methoxy-5-methylbenzo[*b*]thiophene boronic acid. Then, EDC-mediated coupling followed by acetonide deprotection furnished **21** (Scheme 4).

**Scheme 5:**
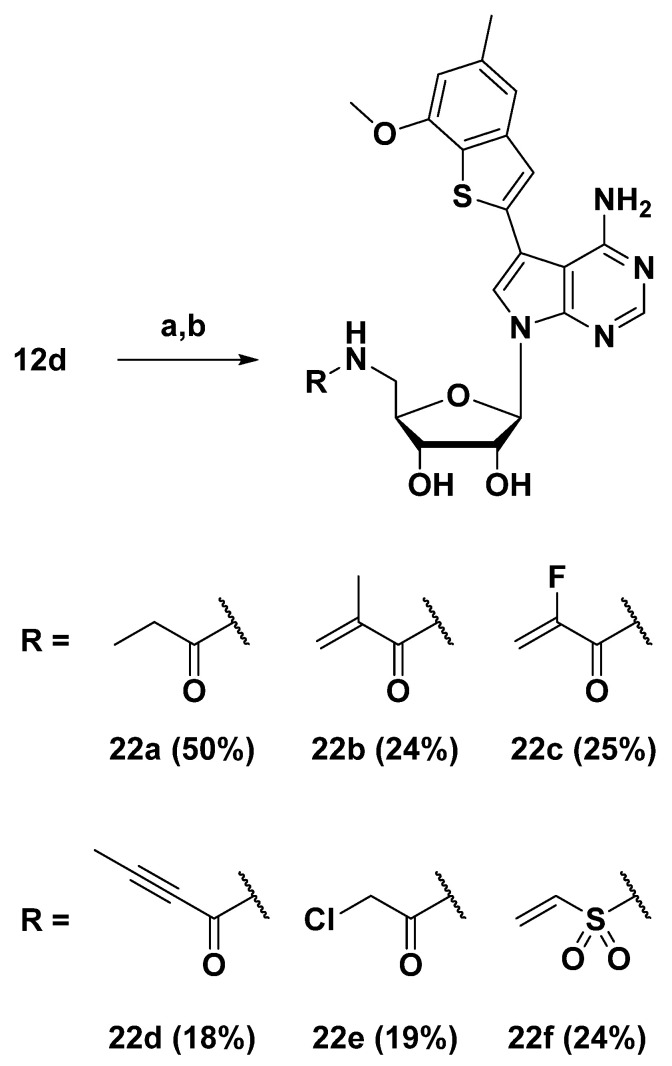
Synthesis of 5′-amide derivatives with diverse electrophilic warheads **22a**–**f**. Reagents and conditions: (a) corresponding acid, EDC·HCl, DMAP, DMF, 0 °C, 2 h for **22a**–**e** or 2-chloroethanesulfonyl chloride, Et_3_N, CH_2_Cl_2_, 0 °C to rt, 17 h for **22f**; (b) 50% HCO_2_H/H_2_O, THF, rt, 24 h, 18–50%.

To investigate the effect of various electrophilic warheads on FGFR inhibition, a series of 5′-amide derivatives **22a**–**f** was synthesized from intermediate **12d** (Scheme 5). Compounds **22a**–**e** were obtained by coupling **12d** with the corresponding carboxylic acids using the EDC coupling reaction. In the case of compound **22f**, 2-chloroethanesulfonyl chloride was used in the presence of Et_3_N in CH_2_Cl_2_ at 0 °C to room temperature overnight. The crude products were treated with 50% formic acid to deprotect the acetonide group, affording the desired products **22a**–**f**.

### 2.2. Biological Evaluation

#### 2.2.1. Biochemical Potencies of Compounds **5a**–**d**, **6a**–**h**

To investigate the characteristics of hydrophobic pocket I of FGFR1–4, we conducted a structure–activity relationship (SAR) study by introducing various aromatic substituents to the C7 position of 7-deaza-adenosine (Table 1). The kinase inhibitory activity of the synthesized compounds was evaluated by KinaseProfiler™ radiometric assay under isoform-optimized conditions. Comparison between **5a**, containing a phenylacetylene moiety, and **6a**, in which the acetylene linker was replaced with a bioisosteric biphenyl group, revealed that **5a** exhibited markedly enhanced activity against FGFR1–3. This suggests that the shorter, rigid, linear acetylene linker better aligns within the binding pocket than the longer biphenyl, which also likely suffers from entropic penalties or suboptimal fit due to its free rotation. The reduced efficacy of **6a**, relative to fused-ring derivatives **6d**–**f**, further supports that short and rigid planar aromatic rings are favored within the hydrophobic pocket. Compound **5b**, containing a methoxy group, showed consistently increased inhibitory activity across FGFR1–4 compared to **5a**. This is due to the methoxy group acting as a hydrogen bond acceptor, enabling additional polar interactions with the kinase domain. Further substitution of **5b** with either a second methoxy (**5c**) or a methyl group (**5d**) maintained potencies. However, the differences were not significant at 1 μM. A removal of acetylene linker in **5c** with a direct aryl connection (**6b**) resulted in a 3.5-fold enhancement against FGFR4.

**Figure 2:**
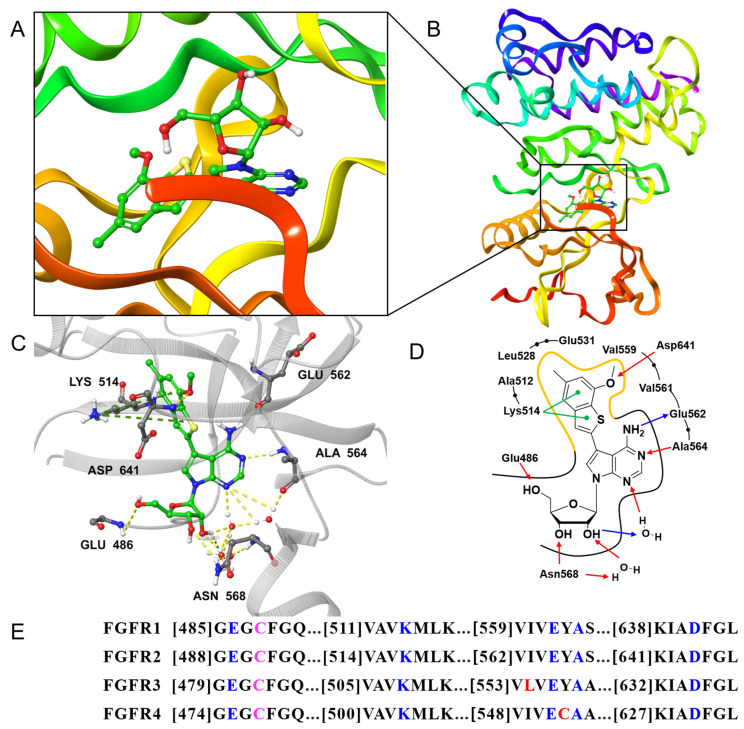
Structure of FGFR1 in complex with compound **6h** (PDB ID: 9WFK). (**A**) Conformation of **6h** (green) within the ligand-binding pocket. (**B**) Overall receptor structure shown as a rainbow-colored ribbon. (**C**) Details of the receptor-ligand interactions. Hydrogen bond (yellow) and cation–*π* interaction (green) with FGFR1 residues are highlighted in dashed lines. (**D**) Schematic diagram of ligand-interacting residues. Hydrogen bonds (blue and red arrows), cation–*π* interactions (green lines with filled circles at the aromatic end), and the hydrophobic pocket I (orange). (**E**) Sequence alignment of the residues of FGFR1–4 proteins. The conserved key interacting amino acids (blue and pink), and non-conserved amino acids (red).

Among fused-ring analogs, a clear trend was observed in activity: benzofuran-2-yl (**6d**) < indole-2-yl (**6f**) < benzothiophen-2-yl (**6e**), with **6e** showing the most pronounced effect, particularly for FGFR4. These trends can be rationalized by the difference in aromaticity, π-electron density of the ring, and the polarizability of the heteroatom [26,27]. The indole (**6f**), being more π-electron-rich than benzofuran (**6d**) due to the lower electronegativity of nitrogen and stronger aromaticity, exhibits enhanced π interactions (cation–π interaction or π–π stacking) resulting in higher potency for **6f** over **6d** (Figure 2D). However, despite the slightly lower aromaticity of the benzothiophene (**6e**) than indole (**6f**), the high polarizability of the sulfur atom compensated by enabling strong dispersion force-driven π interactions, thereby enhancing the overall binding affinity. Compared to **6e**, compound **6c** showed reduced activity notably in FGFR4, indicating that fused aromatic systems better support key hydrophobic and π interactions. Moreover, **6g**, containing a strong electron-withdrawing formyl group, showed further decreased activity than **6c**, reinforcing the role of π-electron density in maintaining effective binding. Finally, **6h**, which incorporates the same electron-donating methoxy-methyl substitution pattern as **5d** onto a highly polarizable benzothiophene that engages strong dispersion forces, exhibited similar potency to **5d** for FGFR1–3 and a 3.3-fold improvement against FGFR4, demonstrating IC_50_ values of 2, 4, 6, and 299 nM for FGFR1–4, respectively.

#### 2.2.2. Crystal Structure of FGFR1 in Complex with Compound **6h**

To elucidate the binding mode of novel nucleoside inhibitors and explain the SAR of synthesized compounds, we attempted to solve the crystal structure of FGFR1 in complex with **6h**. We successfully obtained the co-crystal structure in complex with **6h** using the soaking method and solved its structure at 1.89 Å resolution (Figure 2, Table S3). The 7-methoxy-5-methylbenzo[*b*]thiophene moiety of **6h** is perfectly fit in hydrophobic pocket I by two cation–*π* interactions with Lys514 and a hydrogen bond with Asp641. This observation is consistent with the finding that the π-electron density of the aromatic system and a methoxy substituent are crucial for optimal binding to the hydrophobic pocket. The adenine scaffold of **6h** forms a canonical bidentate hydrogen bond with the hinge region, specifically engaging the carbonyl oxygen of Glu562 (1.93 Å) and the backbone NH of Ala564 (2.20 Å), which are common for other FGFR1 ligands. Notably, the ribose moiety of **6h** occupied the ribose pocket and formed multiple water-mediated hydrogen bond networks, including direct interaction with Asn568 with distances ranging from 1.98 to 3.19 Å. These interactions likely contribute to the enhanced binding affinity mediated by the ribose moiety. The key amino acid residues involved in ligand interaction are conserved among FGFR1–4 (blue in Figure 2E), suggesting that similar binding modes may likely be preserved across all isoforms.

#### 2.2.3. Structures, Biochemical Potencies, and Antiproliferation Efficacy of Compounds **13a**–**f**, ent-**13f**, **19a**–**e**

To determine whether the introduction of a 5′-acrylamide moiety enhances the potency while retaining the key SAR trends observed in Table 1, and simultaneously confers the covalent bonding, we further evaluated the biochemical potency and antiproliferative efficacy of the analogues in FGFR1-amplified HCT116 and FGFR3–TACC3 fusion RT4 cancer cells (Table 2). Interestingly, when comparing compounds sharing a dimethoxyphenyl moiety (**13a** vs. **13e** and **19a** vs. **19d**), the SAR trend slightly diverged from that in Table 1. Specifically, compounds with the acetylene linker (**13a** and **19a**) demonstrated better inhibition against FGFR1–2 than their counterparts (**13e** and **19d**). Notably, in FGFR1, **19a** (IC_50_ = 20 nM) was 3.8-fold more potent than **19d** (IC_50_ = 76 nM), while showing an inconsistent trend in FGFR3 and limited activity against FGFR4. Furthermore, a comparison between **13a** and **13b** revealed that **13b** was 3.2-fold more potent against FGFR1 (IC_50_ = 25 nM) than **13a** (IC_50_ = 81 nM), revealing a small nonpolar methyl group is more favorable than a methoxy group. Notably, comparing **13b** and **13f**, both containing the same methoxy-methylbenzene moiety, the fused-ring compound **13f** consistently outperformed the acetylene-linked **13b** across all FGFR subtypes (IC_50_ = 2, 4, 3, and 102 nM against FGFR1–4, respectively). The impact of ribose modification was also examined by replacing the ribose oxygen atom with sulfur to generate 4′-thio sugars. In matched pairs with identical 7-substituents (**13a** vs. **19a**, **13e** vs. **19d**, and **13f** vs. **19e**), the 4′-thio analogues consistently exhibited enhanced FGFR1–4 inhibition, a trend was also confirmed in FGFR-driven cancer cell lines. In particular, 7-methoxy-5-methylbenzo[*b*]thiophene moiety (**19e**) showed the most potent activity in the series, with IC_50_ = 0.9, 4, 3, and 61 nM and K_d_ = 0.57, 0.64, 1.2, and 1.1 nM against FGFR1–4, providing clear evidence of pan-FGFR inhibition (Table 2 and Table S4, Figure S3). In cell-based assays, **19e** also demonstrated the strongest antiproliferative efficacy, with IC_50_ = 0.52, 1.52 μM in HCT116 and RT4 cells, respectively, outperforming the reference FGFR inhibitors futibatinib and AZD4547. Likewise, **13f** exhibited stronger inhibition than futibatinib and AZD4547 in HCT116 cells and outperformed AZD4547 in RT4 cells, further validating its therapeutic potential. Enantiomeric comparison further highlighted the stereochemical sensitivity of the ribose pocket. The L-ribose-derived ent-**13f** displayed significantly reduced activity compared with **13f**, showing approximately 9.5-fold, 12.8-fold, and 5.7-fold weaker inhibition against FGFR1–3, respectively, and complete activity loss against FGFR4. These results emphasize the importance of the proper stereochemical orientation of the ribose for maintaining key hydrogen bonds, thereby enhancing the potency.

**Figure 3:**
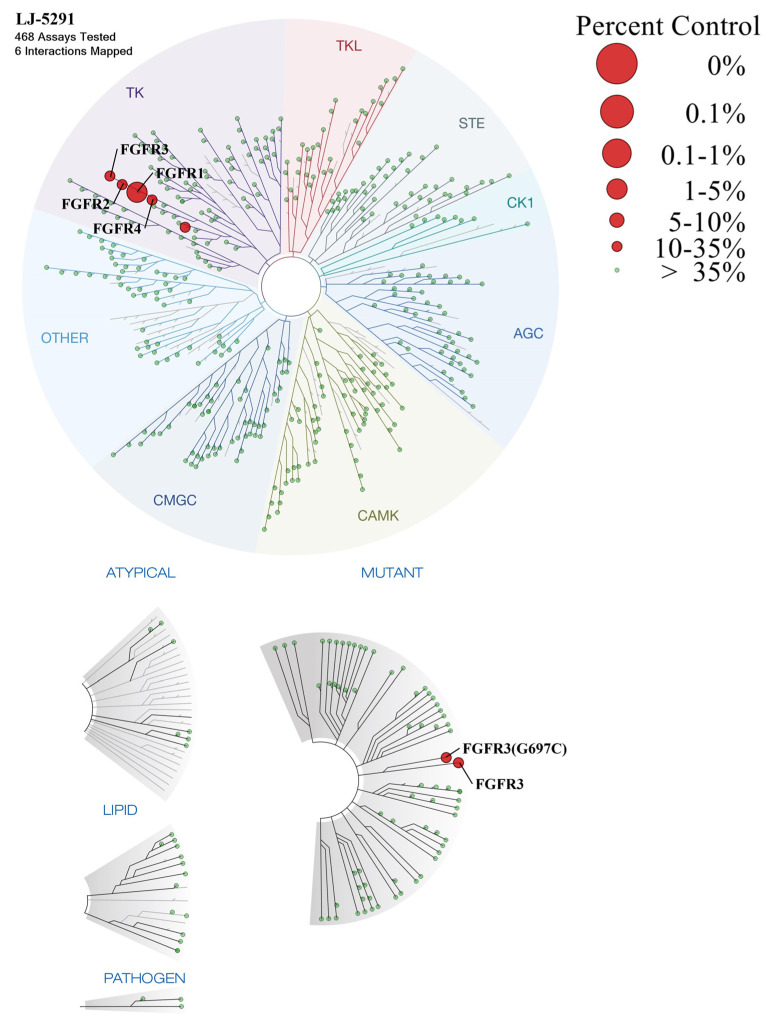
TREEspot™ Interaction Maps. Kinase selectivity profile of compound **13f** at 100 nM against a panel of 468 kinases.

Microsomal stability was evaluated for representative compounds exhibiting strong antiproliferative activity (Table 3). The adenosine analogue **6h** displayed favorable metabolic stability, with half-lives of 68, 65, and 69 min in human, rat, and mouse liver microsomes, respectively, and correspondingly low intrinsic clearance. Notably, the ribose moiety and 7-substituted adenine core showed optimal microsomal stability, suggesting their structural metabolic robustness. In contrast, the acrylamide-containing compound **13f** exhibited poor stability, with half-lives of 9.4, 26.3, and 19.1 min. Interestingly, **19e**, the most potent compound bearing a 4′-thiosugar moiety, showed even shorter half-lives (2.3, 8.3, and 5.1 min) than its oxo counterpart **13f**, indicating higher metabolic liability of the sulfur atom. Given these findings, further investigation was conducted on compound **13f**. To evaluate kinase selectivity, **13f** was profiled across a panel of 468 human kinases and showed strong selectivity toward FGFR1–4 (Figure 3 and Supplementary Materials File S1). The 7-methoxy-5-methyl[*b*]benzothiophene scaffold effectively engages the hydrophobic pocket I of the FGFR ATP-binding site, serving as a key pharmacophore for nucleoside-based FGFR inhibitors. These results underscore its role as a critical determinant of FGFR selectivity, independent of the ribose scaffold. Despite possessing a reactive electrophilic warhead, the compound exhibited high selectivity toward FGFR1–4 with no evidence of nonspecific covalent inhibition against other cysteine-containing kinases, indicating that the observed efficacy is unlikely attributable to off-target driven toxicity.

#### 2.2.4. Structures, Biochemical Potencies, and Antiproliferation Efficacy of Compounds **21**, and **22a**–**f**

Additionally, SAR studies were conducted with various electrophilic warheads (Table 4). Most electrophiles exhibited comparable inhibitory activity against FGFR1–4, with compound **22b** demonstrating the strongest inhibition of FGFR4, whereas compound **22d** showed relatively weak inhibition. Notably, compound **22f** displayed potent antiproliferative effects in both cancer cell lines (IC_50_ = 0.58 and 0.67 μM, respectively), surpassing the efficacy of the reference compounds. Interestingly, compound **22a**, having a propionyl amide, lacking an electrophilic property, still exhibited significant biochemical and cellular activity. This suggests substantial reversible binding affinity to FGFR and strongly supports the validity of our structural design strategy. Compound **21**, which incorporates a methyl substituent on the amide nitrogen, showed similar potency to **13f** against FGFR1–3, but exhibited approximately 1.5-fold weaker inhibition of FGFR4.

**Figure 4:**
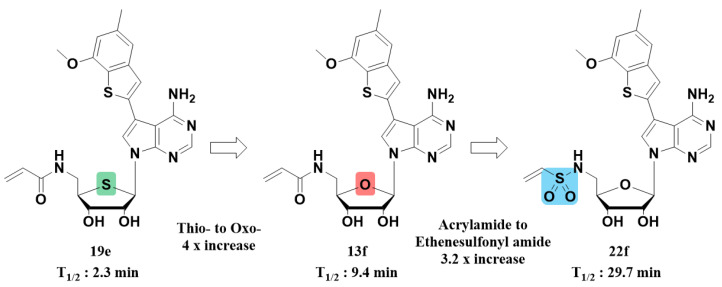
Stepwise structural optimization leading to enhanced microsomal stability.

In terms of metabolic stability (Table 5), the α,β-saturated analogue **22a** displayed similar half-life to **13f**, suggesting that the rapid metabolism is not solely attributable to the α,β-unsaturated electrophilic group. Moreover, compound **21**, despite replacing the nitrogen proton with a methylene group, showed comparable or lower stability than **13f**, indicating that the nitrogen proton is unlikely to be the main contributor to metabolic degradation. Remarkably, **22f** possessing a sulfonamide warhead, with its strong electron-withdrawing effect and sp^3^-hybridized geometry, exhibited improved of half-life, likely due to reduced susceptibility to CYP-mediated oxidative metabolism compared to the planar and less electron-withdrawing acrylamide group (Figure 4). However, the reference compound futibatinib exhibited superior microsomal stability compared to compound **22f**. Nevertheless, our findings indicate that microsomal stability can be further improved by modulating the electrophilic warhead. Therefore, if alternative warhead with improved metabolic stability are introduced while maintaining the strong hydrogen-bonding interactions mediated by the ribose moiety, the nucleoside scaffold could yield highly optimized covalent FGFR inhibitors with improved druggability.

To further evaluate the effect on FGFR1-mediated signaling of compound **22f**, we analyzed downstream signaling proteins by Western blot (Figure S4). Treatment with **22f** led to a dose-dependent inhibition of FGFR1 phosphorylation, while total FGFR1 expression remained unaffected. Consistent with FGFR1 inhibition, phosphorylation of AKT and ERK1/2 also decreased without significant changes in total AKT and ERK1/2 levels. These results demonstrate that **22f** effectively suppresses FGFR1 activation and downstream signaling cascades.

#### 2.2.5. Anticancer Activity of Compounds **13a**–**f**, **19b**–**e**, **21**, and **22a**–**f**

To evaluate whether the compounds exhibit antiproliferative effects beyond FGFR-altered models, five wild-type cancer cell lines with reported FGFR expression or signaling dependency were selected and tested using the SRB assay (Table 6) [28,29,30,31,32,33,34]. Notably, the antiproliferative activity observed in wild-type cancer models showed strong correlation with the in vitro FGFR inhibition data. Compounds that lacked FGFR1–4 inhibitory activity (**19b** and **19c**) or that failed to suppress proliferation in FGFR-altered cancer cell models (**13a** and **19d**) correspondingly exhibited weak antiproliferative effects in the five tested cell lines. Similarly, compound **13b**, which showed moderate inhibition in enzymatic and FGFR-altered cellular assays, displayed moderate efficacy in the wild-type models. In contrast, compounds with potent inhibition of all four FGFR isoforms (**13f**, **19e**, **21**, and **22a**–**f**) demonstrated superior antiproliferative efficacy, with sub-micromolar activity, suggesting a strong correlation between pan-FGFR targeting and broader anticancer effects. These results demonstrate that the compounds can inhibit cancer cell proliferation even in the absence of FGFR alterations, supporting their therapeutic potential in both cancers with or without FGFR genomic aberrations.

#### 2.2.6. Covalent Binding Confirmation via Intact Mass Spectrometry and Western Blot Analysis

**Figure 5:**
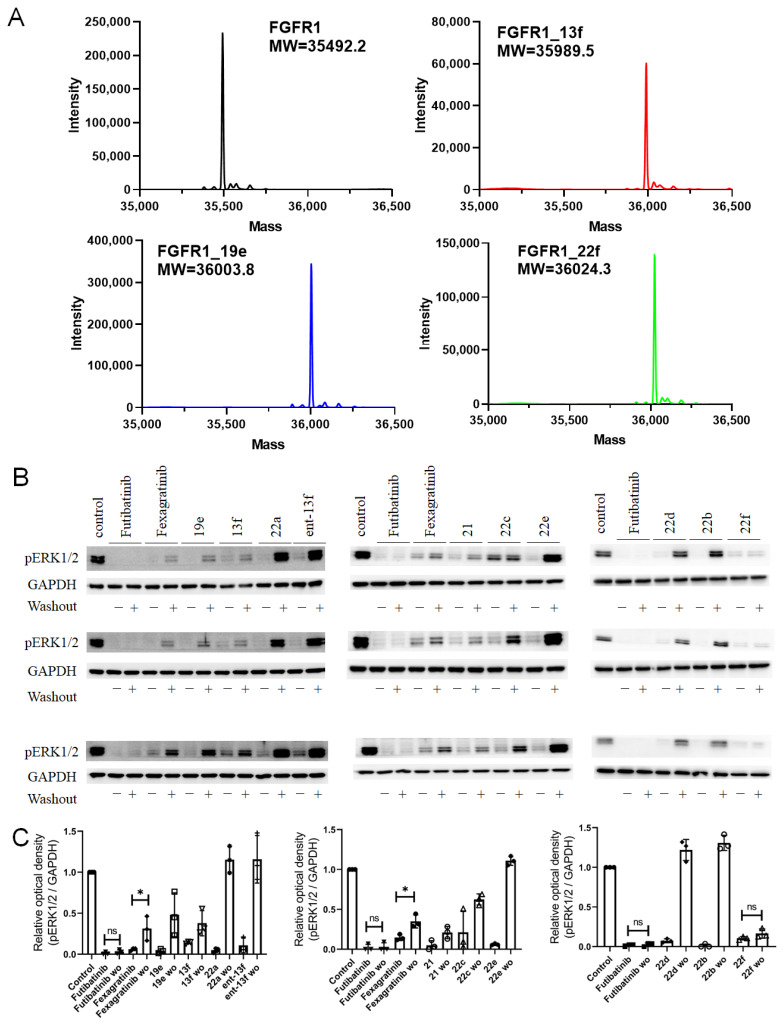
Covalent binding confirmation using in-tact MS analysis of FGFR1 protein and Western blot analysis of pERK1/2 levels in FGFR1-amplified HCT116 cells. (**A**) Deconvoluted intact mass spectra of FGFR1 protein with and without inhibitors: apo FGFR1 (black), FGFR1/**13f** mixture (red), FGFR1/**19e** mixture (blue), and FGFR1/**22f** mixture (Green). (**B**) Western blot analysis of pERK1/2 levels in FGFR1-amplified HCT116 cancer cells treated with the indicated inhibitors for 2 h, followed by 2.5 h washout (wo). (**C**) Quantification of pERK1/2 levels relative to GAPDH from (**B**), shown as mean ± SEM (n = 3).* *p* < 0.05; ns, not significant.

To confirm the covalent bonding to the conserved cysteine residue in FGFRs, both protein-level and cellular-level irreversibility assays were performed (Figure 5). Compounds **13f** (MW: 495.6), **19e** (MW: 511.6), and **22f** (MW: 531.6) were incubated with recombinant FGFR1 protein. Subsequent intact mass spectrometric analysis revealed clear mass shifts, indicative of covalent adduct formation (Figure 5A). To further assess target engagement at the cellular level, pERK1/2 phosphorylation was monitored in FGFR1-amplified HCT116 cells after 2 h compound treatment followed by 2.5 h washout step (Figure 5B,C). The irreversible FGFR inhibitor futibatinib maintained suppression of pERK1/2, while the reversible inhibitor fexagratinib exhibited signal recovery. Compound **22a**, containing a propionylamide, showed complete pERK1/2 signal recovery despite having comparable IC_50_ values to **13f**, indicating a lack of irreversible engagement. In contrast, **13f** and **21** partially retained pERK suppression after washout, suggesting partial covalent engagement. Several analogues failed to maintain pERK suppression, including ent-**13f**, **22e** (chloroacetamide), **22d** (butynoic amide), and **22b** (methacrylamide). The enantiomer ent-**13f** likely lacked the appropriate spatial orientation within the binding pocket for covalent engagement. Compounds **22b** and **22d** may suffer from steric hindrance due to their bulky or rigid electrophilic moieties, whereas **22e** may be limited by inefficient distance for S_N_2 reaction. Finally, **22f**, which contains a highly electrophilic ethenesulfonylamide group, showed persistent suppression of pERK1/2 even after washout, suggesting efficient irreversible binding. This may be attributed to the strong electron-withdrawing nature of the sulfonyl group, which enhances the electrophilicity of the warhead toward the nucleophilic cysteine residue within the FGFR1 active site. As the cysteine residue is conserved across all FGFR isoforms, the covalent binding observed with FGFR1 is expected to extend to FGFR2–4 as well (pink in Figure 2E).

#### 2.2.7. Analysis of Molecular Dynamics

**Figure 6:**
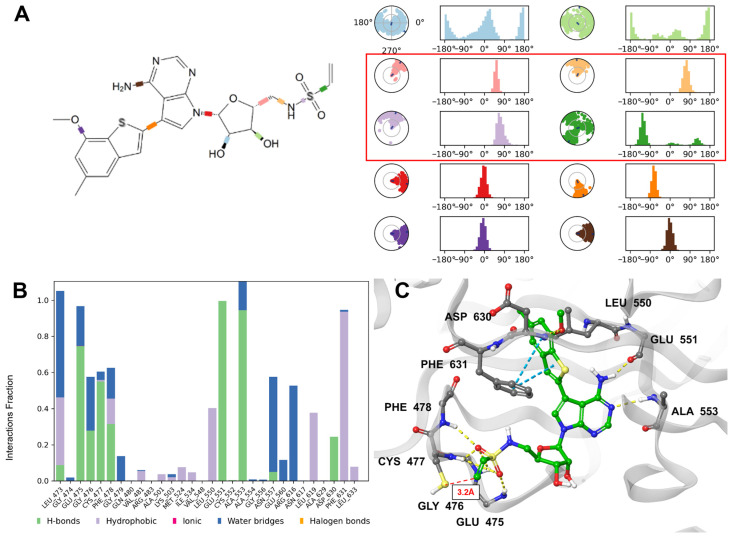
Molecular dynamics (MD) analysis of the **22f** at FGFR4 (PDB ID: 4R6V) (**A**) Ligand torsions plot summarizing the conformational evolution of every rotatable bond in the ligand throughout the simulation trajectory (100 ns). (**B**) Protein-ligand interaction fractions with key residues. (**C**) Representative binding mode of **22f** (green). Hydrogen bond (yellow) and *π*–*π* interaction (sky blue). The timing of the snapshot is represented as the red arrows in Figure S5A.

While non-conserved amino acids in the hinge region of FGFR4 (Cys552, Ala554, etc.) are known to reduce isoform selectivity [35,36], our lead compounds demonstrated uniformly strong dissociation constant (K_d_) values across all FGFR isoforms (Table S4). In addition, compound **22f** exhibited effective covalent bonding with FGFR1 in both protein and cellular levels. To rationalize this pan-FGFR inhibition and effective covalency, we performed three independent 100 ns molecular dynamics (MD) simulations of **22f** in complex with FGFR4 (PDB ID: 4R6V). Across all three independent trajectories, MD trajectories, **22f** consistently maintained key interactions, precisely matched with FGFR1 co-crystal structure (Figure 2 and Figure 6B,C). The adenine scaffold formed hydrogen bonds with Glu551 and Ala553, and the 3′-hydroxyl group of the ribose moiety participated in both direct hydrogen bonding with Asn557 and water-mediated hydrogen bond networks. Additionally, the 7-methoxy-5-methylbenzo[*b*]thiophene moiety engaged in a stable hydrogen bond with Asp630.

The only notable different interaction pattern was observed at Phe631. In the DFG-out conformation of FGFR4 (PDB ID: 4R6V), Phe631 is oriented inward toward the binding cavity, enabling a π–π stacking interaction with the ligand. In contrast, the DFG-in conformation stabilizes the ligand through cation–π interaction with Lys514 in FGFR1 (equivalent to Lys503 in FGFR4). These findings suggest that compound **22f** can be stabilized in both DFG conformations. Furthermore, amino acids previously reported to modulate isoform selectivity, such as Leu554 in FGFR3 (corresponding to Ile549 in FGFR4), Cys552, and Ala554 in FGFR4, did not make any interaction with **22f**, supporting the pan-FGFR inhibition profile (Figure 2E and Figure 6B).

Importantly, the 5′-ethenesulfonamide moiety of **22f** engaged in a stable hydrogen bond network with the P-loop residues Glu475–Phe478, restricting its torsional freedom, and maintained proximity to the Cys477 for covalent bond formation (Figure 6A and Figure S5C). Although the shortest distance between the electrophilic center and the thiol of Cys477 was measured to be 3.2 Å, the average distance across three independent 100 ns simulations was 4.72 ± 0.02 Å (mean ± SEM Å), which may be suboptimal for efficient covalent bond formation (Figure S5D). However, compound **22f**, which has a strong electron-withdrawing sulfonylamide group, was able to form covalent bonds effectively under cellular conditions compared to **13f** and **19e**.

#### 2.2.8. In Vitro ADME /Tox Profiling of Compound **22f**

The in vitro ADME and toxicity profile of compound **22f** was evaluated by measuring its inhibitory effects on major CYP isoforms, metabolic stability in plasma, and hERG channel inhibition (Table 7). Compound 22f exhibited minimal inhibition against key CYP isoforms, with IC_50_ values > 50 μM for CYP3A4/5, 2D6, and 1A2, and moderate inhibition for CYP2C19 (IC_50_ = 20.7 μM) and CYP2C9 (IC_50_ = 10.8 μM). Metabolic stability in plasma was assessed by determining the half-life (T_1/2_) in mouse, rat, and human plasma, resulting in values of 47.3, 32.6, and 37.3 min, respectively. Additionally, 22f showed negligible hERG inhibition, with an IC_50_ value exceeding 30 μM, suggesting a low risk of cardiotoxicity. In addition, in silico ADMET prediction also indicated moderate human oral absorption (41.1%) and low serum albumin binding (logK_HSA_), further supporting the overall drug-like profile of **22f**.

## 3. Materials and Methods

### 3.1. Chemistry

^1^H NMR (400, 500, 600, and 800 MHz) and ^13^C NMR (100, 125, 150, and 200 MHz) spectra were measured in CDCl_3_, CD_3_OD, or DMSO-*d*_6_, and chemical shifts are reported as parts per million (δ) relative to the solvent peak. Coupling constants (J) are reported in hertz (Hz). The purities of several potent compounds were also determined by HPLC (Agilent 1260 infinity series with UV detection at 254 nm, 280 nm) using a binary solvent system [H_2_O, CH_3_CN] using the following column: Agilent zorbax eclipse XDB-C18 column [5.0 μM, 4.6 nm i.d. × 250 mm] (Agilent Technologies, Santa Clara, CA, USA). The HPLC purity was more than 95% purity. High Resolution Mass spectra (HRMS) was operated as Fast atom bombardment (FAB) and Electrospray ionization (ESI) methods. Flash column chromatography was performed on silica gel 60 (230–400 mesh). Unless otherwise noted, materials were obtained from commercial suppliers and were used without purification. All solvents were purified and dried by standard techniques just before use.

#### 3.1.1. Synthesis of Compound **14**

Compound **14** was prepared and identified in the previous study [24].

#### 3.1.2. Synthesis of 7-Substituted-7-deaza Adenosine Derivatives (**5a**–**d**, **6a**–**h**)

Compounds **1**, **2**, and their enantiomer ent-**1**, ent-**2** were synthesized from D- and L-ribose, respectively, following the same synthetic route. Both compounds showed identical NMR spectra and yields.

##### (3*R*,4*S*,5*R*)-5-(((Tert-butyldimethylsilyl)oxy)methyl)tetrahydrofuran-2,3,4-triol (**1**) and (3*R*,4*S*,5*S*)-5-(((Tert-butyldimethylsilyl)oxy)methyl)tetrahydrofuran-2,3,4-triol (ent-**1**)

D-Ribose or L-Ribose (15 g, 0.1 mol) was dissolved in dry pyridine (200 mL), and a solution of TBSCl (15 g, 0.1 mol) in pyridine (50 mL) was added dropwise at 0 °C. The reaction mixture was stirred at 0 °C and then allowed to warm to room temperature, continuing to stir for 17 h. Then the mixture was evaporated under reduced pressure and the residue was purified by column chromatography on silica gel to give compounds **1** or ent-**1** (9.9 g, 39%) as a white solid. ^1^H NMR (400 MHz, CD_3_OD) δ 5.12 (d, *J* = 1.1 Hz, 1H, H-1, α/β), 4.09 (dd, *J* = 5.9, 5.0 Hz, 1H, H-2), 3.89 (td, *J* = 5.9, 3.7 Hz, 1H, H-3), 3.83–3.70 (m, 3H, H-4, H-5), 0.92 (s, 9H, Si-(CH_3_)_3_), 0.10 (s, 6H, Si-(CH_3_)_2_); ^13^C NMR (100 MHz, CD_3_OD) δ 102.9, 84.4, 77.0, 72.3, 66.1, 26.4, 26.4, 19.2, -5.2; Note: The NMR spectra exhibit signals corresponding to both α- and β-anomers due to the presence of a free 1′-OH group, resulting in overlapping peaks. Attempts to resolve individual anomers were unsuccessful due to rapid interconversion in solution.

##### (2*R*,3*S*,4*R*,5*R*)-2-(((Tert-butyldimethylsilyl)oxy)methyl)-5-(4-chloro-5-iodo-7H-pyrrolo[2,3-*d*]pyrimidin-7-yl)tetrahydrofuran-3,4-diol (**2**) and (2*S*,3*S*,4*R*,5*S*)-2-(((Tert-butyldimethylsilyl)oxy)methyl)-5-(4-chloro-5-iodo-7*H*-pyrrolo[2,3-*d*]pyrimidin-7-yl)tetrahydrofuran-3,4-diol (ent-**2**)

To a stirred suspension of 6-Chloro-7-iodo-7-deazapurine (7.1 g, 25.3 mmol) in dry CH_3_CN (50 mL) was added DBU (3.9 mL, 27.8 mmol) at room temperature under N_2_. The resulting mixture was stirred for 10 min. After being cooled to 0 °C, the DIAD (10.7 g, 53.1 mmol) was added dropwise, followed by slow addition of triethylphosphine (6.0 g, 50.6 mmol), and the resulting mixture was stirred for 20 min at the same temperature. To a stirred solution, compound **1** or ent-**1,** which was dissolved in CH_3_CN (50 mL), was added dropwise and the resulting mixture was stirred for 17 h at room temperature. Then the mixture was evaporated under reduced pressure and extracted with EtOAc thrice, and the organic phase was washed with brine, and the combined organic layers were dried over anhydrous MgSO_4_, filtered, and evaporated under reduced pressure. The residue was purified by column chromatography on silica gel to give compounds **2** or ent-**2** (4.9 g, 33%) as a yellow oil. ^1^H NMR (400 MHz, CDCl_3_) δ 8.46 (s, 1H), 7.88 (s, 1H), 6.31 (d, *J* = 5.5 Hz, 1H), 5.38 (s, 1H), 4.52 (s, 1H), 4.41 (s, 1H), 4.29 (d, *J* = 2.3 Hz, 1H), 3.94 (dd, *J* = 11.4, 2.3 Hz, 1H), 3.84 (dd, *J* = 11.4, 2.3 Hz, 1H), 3.48 (s, 1H), 0.91 (s, 9H), 0.13 (d, *J* = 0.9 Hz, 6H); ^13^C NMR (100 MHz, CDCl_3_) δ 152.3, 150.6, 150.2, 132.6, 117.3, 89.6, 86.3, 72.4, 63.6, 52.1, 26.1, 18.6, −5.2, −5.3; MS (ESI) *m*/*z* 526.0398 [M + H]^+^.

##### (2*R*,3*R*,4*S*,5*R*)-2-(4-Amino-5-iodo-7H-pyrrolo[2,3-*d*]pyrimidin-7-yl)-5-(((tert-butyldimethylsilyl)oxy)methyl)tetrahydrofuran-3,4-diol (**3**)

A solution of the **2** (1.56 g, 2.97 mmol) in NH_3_/*t*-BuOH (excess) was stirred at 90 °C for 17 h in a steel bomb, then the solvent was evaporated. The residue was purified by silica gel column chromatography to give **3** (1.22 g, 81%) as a yellow solid. ^1^H NMR (CDCl_3_, 400 MHz): δ 7.97 (s, 1H), 7.50 (s, 1H), 6.34 (d, *J* = 5.9 Hz, 1H), 6.13 (s, 2H), 4.42 (t, *J* = 5.3 Hz, 1H), 4.38–4.36 (m, 1H), 4.26 (d, *J* = 1.8 Hz, 1H), 3.87 (dd, *J* = 38.2, 9.4 Hz, 2H), 0.91 (t, *J* = 15.1 Hz, 9H), 0.11 (td, *J* = 15.1, 3.2 Hz, 6H); ^13^C NMR (CDCl_3_, 100 MHz) δ 156.8, 151.2, 149.2, 126.5, 104.0, 87.9, 85.6, 71.9, 63.5, 50.8, 26.1, 18.6, −5.2, −5.3; MS (ESI) *m*/*z* 507.1013 [M + H]^+^.

##### (2*R*,3*R*,4*S*,5*R*)-2-(4-Amino-5-iodo-7*H*-pyrrolo[2,3-*d*]pyrimidin-7-yl)-5-(hydroxymethyl)tetrahydrofuran-3,4-diol (**4**)

To a solution of compound **3** in THF was added 50% aqueous formic acid solution, and the resulting mixture was stirred for 24 h at room temperature. Acidic solution was basified using a weakly basic anion-exchange resin (Dowex^®^ 66 free base) and stirred for an additional 1 h, filtered, and concentrated under reduced pressure. The residue was purified by silica gel column chromatography to afford **4** (718 mg, 76%) as a white solid. ^1^H NMR (400 MHz, DMSO-*d*_6_) δ 8.10 (s, 1H), 7.67 (s, 1H), 6.69 (s, 2H), 6.02 (d, *J* = 6.4 Hz, 1H), 5.32 (d, *J* = 6.0 Hz, 1H), 5.17 (t, *J* = 5.1 Hz, 1H), 5.13 (d, *J* = 4.1 Hz, 1H), 4.35 (q, *J* = 5.5 Hz, 1H), 4.07 (s, 1H), 3.88 (q, *J* = 3.5 Hz, 1H), 3.64–3.60 (m, 1H), 3.55–3.50 (m, 1H); ^13^C NMR (100 MHz, DMSO-*d*_6_) δ 157.2, 152.0, 150.2, 127.2, 103.3, 86.9, 85.2, 73.9, 70.6, 61.6, 51.9;

#### 3.1.3. General Procedure for Sonogashira Coupling for the Preparation of **5a**–**d**

To a microwave vial equipped with a septum, containing starting material, CuI (25 mol%), and PdCl_2_(PPh_3_)_2_ (10 mol %) was added a degassed mixture of DMF/Et_3_N (4:1). The resulting mixture was degassed with nitrogen for 5 min before adding corresponding alkynes (2.0 equiv) and heated in microwave for 1h at 50 °C. Then the mixture was extracted with EtOAc thrice, and the organic phase was washed with brine, and the combined organic layers were dried over anhydrous MgSO_4_, filtered, and evaporated under reduced pressure. The residue was purified by column chromatography on silica to give **5a**–**d**.

##### (2*R*,3*R*,4*S*,5*R*)-2-(4-Amino-5-(phenylethynyl)-7*H*-pyrrolo[2,3-*d*]pyrimidin-7-yl)-5-(hydroxymethyl)tetrahydrofuran-3,4-diol (**5a**)

38 mg (81%); yellow solid; ^1^H NMR (400 MHz, DMSO-*d*_6_) δ 8.15 (s, 1H), 7.90 (s, 1H), 7.59 (dd, *J* = 7.5, 2.1 Hz, 2H), 7.45–7.41 (m, 3H), 6.74 (s, 2H), 6.06 (d, *J* = 6.4 Hz, 1H), 5.37 (d, *J* = 6.4 Hz, 1H), 5.22 (t, *J* = 5.5 Hz, 1H), 5.15 (d, *J* = 5.0 Hz, 1H), 4.40 (q, *J* = 5.8 Hz, 1H), 4.10 (dd, *J* = 8.0, 4.8 Hz, 1H), 3.91 (q, *J* = 3.4 Hz, 1H), 3.67–3.53 (m, 2H); ^13^C NMR (100 MHz, DMSO-*d*_6_) δ 157.6, 152.8, 131.1, 128.7, 128.5, 127.2, 122.5, 102.2, 94.7, 91.1, 87.2, 85.3, 83.0, 74.1, 70.6, 61.5; HRMS (ESI) found 367.1406 [calcd. for C_19_H_19_N_4_O_4_^+^ (M + H)^+^ 367.1401].

##### (2*R*,3*R*,4*S*,5*R*)-2-(4-Amino-5-((3-methoxy-5-methylphenyl)ethynyl)-7*H*-pyrrolo[2,3-*d*]pyrimidin-7-yl)-5-(hydroxymethyl)tetrahydrofuran-3,4-diol (**5b**)

46 mg (91%); yellow solid; ^1^H NMR (400 MHz, DMSO-*d*_6_) δ 8.16 (s, 1H), 7.90 (s, 1H), 7.33 (t, *J* = 8.2 Hz, 1H), 7.16 (td, *J* = 2.5, 1.4 Hz, 2H), 7.00–6.97 (m, 1H), 6.74 (s, 2H), 6.06 (d, *J* = 5.9 Hz, 1H), 5.38 (d, *J* = 6.4 Hz, 1H), 5.22 (s, 1H), 5.16 (d, *J* = 3.2 Hz, 1H), 4.40 (q, *J* = 5.3 Hz, 1H), 4.10 (s, 1H), 3.91 (q, *J* = 3.5 Hz, 1H), 3.67–3.54 (m, 2H); ^13^C NMR (100 MHz, DMSO-*d*_6_) δ 159.2, 157.6, 152.8, 149.9, 145.0, 129.9, 127.4, 123.6, 123.6, 116.0, 114.9, 94.7, 91.1, 87.2, 85.3, 82.8, 74.1, 70.6, 61.6, 55.3; HRMS (ESI) found 397.1498 [calcd for C_20_H_21_N_4_O_5_^+^ (M + H)^+^ 397.1506].

##### (2*R*,3*R*,4*S*,5*R*)-2-(4-Amino-5-((3,5-dimethoxyphenyl)ethynyl)-7*H*-pyrrolo[2,3-*d*]pyrimidin-7-yl)-5-(hydroxymethyl)tetrahydrofuran-3,4-diol (**5c**)

82 mg (75%); yellow solid; ^1^H NMR (400 MHz, DMSO-*d*_6_) δ 8.16 (s, 1H), 7.90 (s, 1H), 6.76 (d, *J* = 2.3 Hz, 2H), 6.55 (t, *J* = 2.3 Hz, 1H), 6.06 (d, *J* = 5.9 Hz, 1H), 5.39 (d, *J* = 6.4 Hz, 1H), 5.23 (t, *J* = 5.7 Hz, 1H), 5.17 (d, *J* = 5.0 Hz, 1H), 4.40 (q, *J* = 5.8 Hz, 1H), 4.10 (dd, *J* = 8.0, 4.8 Hz, 1H), 3.92 (q, *J* = 3.4 Hz, 1H), 3.77 (s, 6H), 3.67–3.53 (m, 2H); ^13^C NMR (100 MHz, DMSO-*d*_6_) δ 160.4, 157.6, 152.9, 149.9, 127.5, 124.0, 108.9, 102.1, 101.5, 94.6, 91.2, 87.2, 85.4, 82.7, 74.1, 70.6, 61.6, 55.4; HRMS (ESI) found 427.1623 [calcd for C_21_H_23_N_4_O_6_^+^ (M + H)^+^ 427.1612].

##### (2*R*,3*R*,4*S*,5*R*)-2-(4-Amino-5-((3-methoxy-5-methylphenyl)ethynyl)-7*H*-pyrrolo[2,3-*d*]pyrimidin-7-yl)-5-(hydroxymethyl)tetrahydrofuran-3,4-diol (**5d**)

27 mg (52%); yellow solid; ^1^H NMR (400 MHz, DMSO-*d*_6_) δ 8.16 (s, 1H), 7.89 (s, 1H), 7.00 (s, 1H), 6.96 (s, 1H), 6.81 (s, 1H), 6.74 (s, 2H), 6.06 (d, *J* = 6.0 Hz, 1H), 5.38 (d, *J* = 6.4 Hz, 1H), 5.22 (t, *J* = 5.5 Hz, 1H), 5.16 (d, *J* = 4.6 Hz, 1H), 4.40 (q, *J* = 5.8 Hz, 1H), 4.10 (q, *J* = 4.1 Hz, 1H), 3.92 (q, *J* = 3.4 Hz, 1H), 3.77 (s, 3H), 3.67–3.53 (m, 2H), 2.29 (s, 3H); ^13^C NMR (100 MHz, DMSO-*d*_6_) δ 159.2, 157.6, 152.8, 149.8, 139.5, 127.3, 124.2, 123.2, 115.6, 113.2, 102.2, 94.7, 91.3, 87.2, 85.3, 82.5, 74.1, 70.6, 61.6, 55.2, 40.1, 39.9, 39.7, 39.5, 39.3, 39.1, 38.9, 20.9; HRMS (ESI) found 411.1668 [calcd for C_21_H_23_N_4_O_5_^+^ (M + H)^+^ 411.1663].

#### 3.1.4. General Procedure for Suzuki Coupling for the Preparation of **6a**–**h**

In a microwave vial, starting material (1 equiv), corresponding boronic ester (1.2 equiv), PdCl_2_(PPh_3_)_2_ (6 mol%), and sodium carbonate (2 equiv) were taken, and the vial was sealed with a septum. To this mixture was added degassed DMF/H_2_O (0.14 M/0.36 M), and the reaction mixture was heated in a microwave at 70 °C. The reaction was quenched after 1 h with water and extracted with EtOAc thrice. The organic phase was washed with brine, and the combined organic layers were dried over anhydrous MgSO_4_, filtered, and evaporated under reduced pressure. The residue was purified by column chromatography on silica to give **6a**–**h**.

##### (2*R*,3*R*,4*S*,5*R*)-2-(5-([1,1′-Biphenyl]-4-yl)-4-amino-7*H*-pyrrolo[2,3-*d*]pyrimidin-7-yl)-5-(hydroxymethyl)tetrahydrofuran-3,4-diol (**6a**)

9 mg (17%); yellow solid; ^1^H NMR (400 MHz, DMSO-*d*_6_) δ 8.17 (s, 1H), 7.81 (s, 1H), 7.79 (s, 1H), 7.75 (s, 1H), 7.73 (s, 1H), 7.61 (s, 1H), 7.58 (s, 1H), 7.56 (s, 1H), 7.49 (t, *J* = 7.8 Hz, 2H), 7.38 (t, *J* = 7.3 Hz, 1H), 6.25 (s, 2H), 6.14 (d, *J* = 6.4 Hz, 1H), 5.36 (d, *J* = 6.4 Hz, 1H), 5.22 (t, *J* = 5.7 Hz, 1H), 5.15 (d, *J* = 5.0 Hz, 1H), 4.48 (q, *J* = 5.9 Hz, 1H), 4.12 (dd, *J* = 8.0, 4.8 Hz, 1H), 3.92 (q, *J* = 3.5 Hz, 1H), 3.66–3.54 (m, 2H); ^13^C NMR (100 MHz, DMSO-*d*_6_) δ 157.4, 151.8, 151.0, 139.7, 138.5, 133.6, 129.1, 128.9, 127.5, 127.3, 126.6, 121.4, 116.0, 100.5, 87.1, 85.2, 73.9, 70.7, 61.7; HRMS (ESI) found 419.1724 [calcd for C_23_H_23_N_4_O_4_^+^ (M + H)^+^ 419.1714].

##### (2*R*,3*R*,4*S*,5*R*)-2-(4-Amino-5-(3,5-dimethoxyphenyl)-7*H*-pyrrolo[2,3-*d*]pyrimidin-7-yl)-5-(hydroxymethyl)tetrahydrofuran-3,4-diol (**6b**)

14 mg (27%); yellow solid; ^1^H NMR (400 MHz, DMSO-*d*_6_) δ 8.15 (s, 1H), 7.59 (s, 1H), 6.61 (d, *J* = 2.3 Hz, 2H), 6.50 (t, *J* = 2.3 Hz, 1H), 6.11 (d, *J* = 6.4 Hz, 1H), 5.35 (d, *J* = 6.4 Hz, 1H), 5.22 (t, *J* = 5.7 Hz, 1H), 5.14 (d, *J* = 4.6 Hz, 1H), 4.45 (q, *J* = 5.9 Hz, 1H), 4.11 (q, *J* = 4.3 Hz, 1H), 3.90 (q, *J* = 3.7 Hz, 1H), 3.79 (s, 6H), 3.66–3.51 (m, 2H); ^13^C NMR (100 MHz, DMSO-*d*_6_) δ 160.8, 157.3, 151.7, 150.8, 136.4, 121.3, 116.3, 106.4, 98.9, 87.0, 85.1, 73.8, 70.6, 61.6, 55.3; HRMS (ESI) found 403.1603 [calcd for C_19_H_23_N_4_O_6_^+^ (M + H)^+^ 403.1612].

##### (2*R*,3*R*,4*S*,5*R*)-2-(4-Amino-5-(thiophen-2-yl)-7*H*-pyrrolo[2,3-*d*]pyrimidin-7-yl)-5-(hydroxymethyl)tetrahydrofuran-3,4-diol (**6c**)

25 mg (56%); yellow solid; ^1^H NMR (400 MHz, DMSO-*d*_6_) δ 8.16 (s, 1H), 7.63 (s, 1H), 7.57 (d, *J* = 5.0 Hz, 1H), 7.19–7.15 (m, 2H), 6.35 (s, 2H), 6.11 (d, *J* = 6.4 Hz, 1H) 5.37 (d, *J* = 6.4 Hz, 1H), 5.22 (t, *J* = 5.3 Hz, 1H), 5.16 (d, *J* = 4.6 Hz, 1H), 4.44 (q, *J* = 5.9 Hz, 1H), 4.10 (dd, *J* = 7.3, 4.6 Hz, 1H), 3.91 (d, *J* = 3.2 Hz, 1H), 3.66–3.50 (m, 2H); ^13^C NMR (100 MHz, DMSO-*d*_6_) δ 157.3, 152.1, 150.7, 135.6, 128.3, 126.4, 125.9, 122.1, 108.5, 100.7, 87.0, 85.2, 73.9, 70.6, 61.6; HRMS (ESI) found 349.0969 [calcd for C_15_H_17_N_4_O_4_S^+^ (M + H)^+^ 349.0965].

##### (2*R*,3*R*,4*S*,5*R*)-2-(4-Amino-5-(benzofuran-2-yl)-7*H*-pyrrolo[2,3-*d*]pyrimidin-7-yl)-5-(hydroxymethyl)tetrahydrofuran-3,4-diol (**6d**)

7 mg (14%); yellow solid; ^1^H NMR (400 MHz, DMSO-*d*_6_) δ 8.18 (s, 1H), 7.98 (d, *J* = 7.8 Hz, 1H), 7.88 (d, *J* = 7.4 Hz, 1H), 7.78 (s, 1H), 7.45 (s, 1H), 7.39 (dtd, *J* = 17.6, 7.5, 1.4 Hz, 2H), 6.54 (s, 2H), 6.13 (d, *J* = 6.0 Hz, 1H), 5.39 (d, *J* = 6.4 Hz, 1H), 5.24 (t, *J* = 5.5 Hz, 1H), 5.16 (d, *J* = 5.1 Hz, 1H), 4.46 (q, *J* = 6.0 Hz, 1H), 4.12 (q, *J* = 4.1 Hz, 1H), 3.92 (q, *J* = 3.4 Hz, 1H), 3.65 (td, *J* = 8.2, 4.0 Hz, 1H), 3.55 (qd, *J* = 6.0, 3.8 Hz, 1H); ^13^C NMR (100 MHz, DMSO-*d*_6_) δ 157.3, 153.8, 152.3, 151.2, 151.1, 128.9, 123.9, 123.5, 122.8, 120.7, 111.1, 105.5, 101.6, 99.4, 87.1, 85.3, 73.9, 70.5, 61.6; HRMS (ESI) found 383.1339 [calcd for C_19_H_19_N_4_O_5_^+^ (M + H)^+^ 383.1350].

##### (2*R*,3*R*,4*S*,5*R*)-2-(4-Amino-5-(benzo[*b*]thiophen-2-yl)-7*H*-pyrrolo[2,3-*d*]pyrimidin-7-yl)-5-(hydroxymethyl)tetrahydrofuran-3,4-diol (**6e**)

47 mg (92%); yellow solid; ^1^H NMR (400 MHz, DMSO-*d*_6_) δ 8.18 (s, 1H), 8.11 (s, 1H), 7.68–7.64 (m, 2H), 7.32–7.26 (m, 2H), 7.13 (s, 1H), 7.01 (s, 2H), 6.13 (d, *J* = 6.0 Hz, 1H), 5.42 (d, *J* = 4.6 Hz, 1H), 5.24 (t, *J* = 5.5 Hz, 1H), 5.19 (d, *J* = 3.2 Hz, 1H), 4.45 (d, *J* = 5.1 Hz, 1H), 4.13 (s, 1H), 3.93 (q, *J* = 3.5 Hz, 1H), 3.67 (dt, *J* = 11.9, 4.4 Hz, 1H), 3.60–3.54 (m, 1H); ^13^C NMR (100 MHz, DMSO-*d*_6_) δ 157.4, 152.2, 150.9, 140.4, 138.9, 136.1, 124.7, 124.3, 123.6, 122.9, 122.4, 122.2, 108.8, 100.4, 87.1, 85.3, 73.9, 70.6, 61.6; HRMS (ESI) found 399.1129 [calcd for C_19_H_19_N_4_O_4_S^+^ (M + H)^+^ 399.1122].

##### (2*R*,3*R*,4*S*,5*R*)-2-(4-Amino-5-(1H-indol-2-yl)-7*H*-pyrrolo[2,3-*d*]pyrimidin-7-yl)-5-(hydroxymethyl)tetrahydrofuran-3,4-diol (**6f**)

42 mg (86%); yellow solid; ^1^H NMR (400 MHz, DMSO-*d*_6_) δ 11.42 (s, 1H), 8.18 (s, 1H), 7.70 (s, 1H), 7.56 (d, *J* = 7.8 Hz, 1H), 7.40 (d, *J* = 7.8 Hz, 1H), 7.11 (t, *J* = 7.3 Hz, 1H), 7.02 (t, *J* = 7.3 Hz, 1H), 6.54 (s, 3H), 6.15 (d, *J* = 5.9 Hz, 1H), 5.41 (d, *J* = 6.4 Hz, 1H), 5.21 (d, *J* = 3.7 Hz, 2H), 4.46 (q, *J* = 5.6 Hz, 1H), 4.14 (d, *J* = 3.2 Hz, 1H), 3.95 (d, *J* = 3.2 Hz, 1H), 3.67–3.56 (m, 2H); ^13^C NMR (100 MHz, DMSO-*d*_6_) δ 157.5, 152.1, 150.7, 136.8, 132.3, 128.9, 121.7, 121.3, 119.8, 119.4, 111.3, 108.1, 100.6, 100.4, 87.1, 85.2, 74.0, 70.7, 61.8; HRMS (ESI) found 382.1523 [calcd for C_19_H_20_N_5_O_4_^+^ (M + H)^+^ 382.1510].

##### 5-(4-Amino-7-((2*R*,3*R*,4*S*,5*R*)-3,4-dihydroxy-5-(hydroxymethyl)tetrahydrofuran-2-yl)-7*H*-pyrrolo[2,3-*d*]pyrimidin-5-yl)thiophene-2-carbaldehyde (**6g**)

25 mg (26%); orange solid; ^1^H NMR (400 MHz, DMSO-*d*_6_) δ 9.91 (s, 1H), 8.19 (s, 1H), 8.06 (d, *J* = 3.7 Hz, 1H), 7.89 (s, 1H), 7.32 (d, *J* = 3.7 Hz, 1H), 6.58 (s, 2H), 6.12 (d, *J* = 6.4 Hz, 1H), 5.40 (d, *J* = 6.4 Hz, 1H), 5.23 (t, *J* = 5.5 Hz, 1H), 5.17 (d, *J* = 5.0 Hz, 1H), 4.43 (q, *J* = 5.8 Hz, 1H), 4.11 (dd, *J* = 8.2, 5.0 Hz, 1H), 3.92 (q, *J* = 3.5 Hz, 1H), 3.68–3.52 (m, 2H); ^13^C NMR (100 MHz, DMSO-*d*_6_) δ 184.0, 157.4, 152.4, 151.3, 145.9, 141.7, 139.4, 127.4, 123.6, 108.4, 100.0, 87.1, 85.3, 74.0, 70.5, 61.5; HRMS (ESI) found 377.0925 [calcd for C_16_H_17_N_4_O_5_S^+^ (M + H)^+^ 377.0914].

##### (2*R*,3*R*,4*S*,5*R*)-2-(4-Amino-5-(7-methoxy-5-methylbenzo[*b*]thiophen-2-yl)-7*H*-pyrrolo[2,3-*d*]pyrimidin-7-yl)-5-(hydroxymethyl)tetrahydrofuran-3,4-diol (**6h**)

42 mg (71%); yellow solid; ^1^H NMR (400 MHz, DMSO-*d*_6_) δ 8.19 (s, 1H), 7.79 (s, 1H), 7.34 (s, 1H), 7.28 (s, 1H), 6.81 (s, 1H), 6.54 (s, 2H), 6.13 (d, *J* = 6.0 Hz, 1H), 5.38 (d, *J* = 6.0 Hz, 1H), 5.24 (s, 1H), 5.15 (s, 1H), 4.45 (d, *J* = 5.5 Hz, 1H), 4.13 (s, 1H), 3.94 (m, 4H), 3.66 (d, *J* = 9.2 Hz, 1H), 3.56 (d, *J* = 11.5 Hz, 1H), 2.43 (s, 3H); ^13^C NMR (100 MHz, DMSO-*d*_6_) δ 157.3, 153.3, 152.1, 150.9, 142.0, 136.3, 135.8, 124.3, 122.9, 122.5, 116.2, 108.9, 106.5, 100.4, 87.1, 85.2, 74.0, 70.6, 61.5, 55.7, 21.5; HRMS (ESI) found 443.1380 [calcd for C_21_H_23_N_4_O_5_S^+^ (M + H)^+^ 443.1384].

#### 3.1.5. Synthesis of Enantiomer of 7-Substituted-7-deaza adenosine Derivatives

Compound **7**–**10**, **12d**, and **13f**, and their enantiomers ent-**7**–**10**, ent-**12d***,* and ent-**13f** were synthesized from D- and L-ribose, respectively, following the same synthetic route. Both compounds showed identical NMR spectra and yields.

##### 7-((3a*R*,4*R*,6*R*,6a*R*)-6-(((Tert-butyldimethylsilyl)oxy)methyl)-2,2-dimethyltetrahydrofuro[3,4-*d*][1,3]dioxol-4-yl)-4-chloro-5-iodo-7*H*-pyrrolo[2,3-*d*]pyrimidine (**7**) and 7-((3a*R*,4*S*,6*S*,6a*R*)-6-(((Tert-butyldimethylsilyl)oxy)methyl)-2,2-dimethyltetrahydrofuro[3,4-*d*][1,3]dioxol-4-yl)-4-chloro-5-iodo-7*H*-pyrrolo[2,3-*d*]pyrimidine (ent-**7**)

To a solution of **2** or ent-**2** (5.7 g, 10.9 mmol) in acetone (50 mL) was added 2,2-dimethoxypropane (13.4 mL, 109 mmol) and p-toluenesulfonic acid monohydrate (31 mg, 0.001 mmol). The reaction mixture was stirred for 6 h at room temperature. Then the mixture was evaporated under reduced pressure and extracted with EtOAc (3 × 100 mL), and the organic phase was washed with brine, and the combined organic layers were dried over anhydrous MgSO_4_, filtered, and evaporated under reduced pressure. The residue was purified by column chromatography on silica gel to give compounds **7** or ent-**7** (4.4 g, 72%) as a colorless oil. ^1^H NMR (400 MHz, CDCl_3_) δ 8.65 (s, 1H), 7.78 (s, 1H), 6.43 (d, *J* = 3.2 Hz, 1H), 4.94 (q, *J* = 2.9 Hz, 1H), 4.90 (q, *J* = 2.7 Hz, 1H), 4.40 (q, *J* = 2.6 Hz, 1H), 3.92 (dd, *J* = 11.4, 2.7 Hz, 1H), 3.81 (dd, *J* = 11.4, 3.2 Hz, 1H), 1.65 (s, 3H), 1.38 (s, 3H), 0.92 (s, 9H), 0.12 (d, *J* = 2.3 Hz, 6H); ^13^C NMR (100 MHz, CDCl_3_) δ 152.8, 151.2, 150.6, 132.2, 117.6, 114.4, 91.0, 86.5, 85.7, 81.1, 63.7, 52.4, 27.5, 26.2, 25.5, 18.6, −5.1, −5.3; MS (ESI) *m*/*z* 566.0753 [M + H]^+^.

##### 7-((3a*R*,4*R*,6*R*,6a*R*)-6-(((Tert-butyldimethylsilyl)oxy)methyl)-2,2-dimethyltetrahydrofuro[3,4-*d*][1,3]dioxol-4-yl)-5-iodo-7*H*-pyrrolo[2,3-*d*]pyrimidin-4-amine (**8**) and 7-((3a*R*,4*S*,6*S*,6a*R*)-6-(((Tert-butyldimethylsilyl)oxy)methyl)-2,2-dimethyltetrahydrofuro[3,4-*d*][1,3]dioxol-4-yl)-5-iodo-7*H*-pyrrolo[2,3-*d*]pyrimidin-4-amine (ent-**8**)

A solution of the **7** or ent-**7** (4.4 g, 7.78 mmol) in a saturated solution of NH_3_/*t*-BuOH (excess) was stirred at 90 °C for 24 h in a steel bomb. The steel bomb containing the reaction mixture was cooled to room temperature, and the solvent was evaporated under reduced pressure. The residue was purified by silica gel column chromatography to give **8** or ent-**8** (4.0 g, 94%) as a yellow sticky oil. ^1^H NMR (400 MHz, CDCl_3_) δ 8.28 (s, 1H), 7.40 (s, 1H), 6.34 (d, *J* = 2.8 Hz, 1H), 5.80 (s, 2H), 4.97 (q, *J* = 3.1 Hz, 1H), 4.90 (q, *J* = 3.1 Hz, 1H), 4.33 (q, *J* = 3.2 Hz, 1H), 3.89 (dd, *J* = 11.5, 3.2 Hz, 1H), 3.79 (dd, *J* = 11.0, 3.7 Hz, 1H), 1.63 (s, 3H), 1.38 (d, *J* = 11.9 Hz, 3H), 0.92 (s, 9H), 0.08 (d, *J* = 12.9 Hz, 6H); ^13^C NMR (100 MHz, CDCl_3_) δ 156.8, 152.2, 150.1, 126.9, 114.2, 90.5, 86.3, 85.5, 81.0, 63.6, 50.8, 27.5, 26.2, 25.6, 18.6, −5.1, −5.3; MS (ESI) *m*/*z* 547.1360 [M + H]^+^.

##### ((3a*R*,4*R*,6*R*,6a*R*)-6-(4-Amino-5-iodo-7*H*-pyrrolo[2,3-*d*]pyrimidin-7-yl)-2,2-dimethyltetrahydrofuro[3,4-*d*][1,3]dioxol-4-yl)methanol (9) ((3a*S*,4*S*,6*S*,6a*S*)-6-(4-Amino-5-iodo-7*H*-pyrrolo[2,3-*d*]pyrimidin-7-yl)-2,2-dimethyltetrahydrofuro[3,4-*d*][1,3]dioxol-4-yl)methanol (ent-9)

To a solution of **8** or ent-**8** (4.0 g, 7.4 mmol) in THF (100 mL) was added a solution of 1.0 M TBAF in THF (11.1 mL, 11.1 mmol) at room temperature under N_2_. After being stirred for 40 min at room temperature, the reaction mixture was quenched with aqueous ammonium chloride solution (30 mL). Then the mixture was evaporated under reduced pressure and extracted with EtOAc (3 × 100 mL), and the organic phase was washed with brine, and the combined organic layers were dried over anhydrous MgSO_4_, filtered, and evaporated under reduced pressure. The residue was purified by column chromatography on silica to give **9** or ent-**9** (2.8 g, 86%) as a white solid. ^1^H NMR (400 MHz, CD_3_OD) δ 8.10 (s, 1H), 7.58 (s, 1H), 6.18 (d, *J* = 3.7 Hz, 1H), 5.10 (q, *J* = 3.2 Hz, 1H), 4.97 (q, *J* = 2.9 Hz, 1H), 4.27 (d, *J* = 2.8 Hz, 1H), 3.73 (qd, *J* = 12.4, 3.7 Hz, 2H), 1.60 (s, 3H), 1.36 (s, 3H); ^13^C NMR (100 MHz, CD_3_OD) δ 158.9, 152.9, 150.8, 129.6, 115.4, 105.5, 92.1, 87.1, 85.4, 82.6, 63.4, 51.8, 27.6, 25.6; MS (ESI) *m*/*z* 433.0396 [M + H]^+^.

##### 7-((3a*R*,4*R,*6*R*,6a*R*)-6-(Aminomethyl)-2,2-dimethyltetrahydrofuro[3,4-*d*][1,3]dioxol-4-yl)-5-iodo-7*H*-pyrrolo[2,3-*d*]pyrimidin-4-amine (**10**) and 7-((3a*S*,4*S*,6*S*,6a*S*)-6-(Aminomethyl)-2,2-dimethyltetrahydrofuro[3,4-*d*][1,3]dioxol-4-yl)-5-iodo-7*H*-pyrrolo[2,3-*d*]pyrimidin-4-amine (ent-**10**)

To dry THF (70 mL) at 0 °C, PPh_3_ (3.4 g, 13.0 mmol) was added under N_2_. The solution was allowed to stir for 10 min, and DIAD (2.56 mL, 13.0 mmol) was added dropwise. The resulting mixture was stirred for an additional time until it became a white suspension. The ice bath was removed, and to the warming solution was added DPPA (2.8 mL, 1.3 mmol) over a period of 5 min, followed by the addition of **9** or ent-**9** (2.3 g, 5.2 mmol), which was dissolved in dry THF (30 mL). The reaction mixture was stirred for 17 h at room temperature. Then the mixture was quenched with H_2_O (10 mL) and extracted with EtOAc (3 × 100 mL). The organic phase was washed with brine, and the combined organic layers were dried over anhydrous MgSO_4_, filtered, and evaporated under reduced pressure to give crude products and used in next step without further purification. To a solution of crude products in dry THF (20 mL) was added PPh_3_ (4.1 g, 15.6 mmol), and it was stirred for 17 h at room temperature under N_2_. Then to the reaction mixture ammonium hydroxide (2 mL) was added and stirred for 2 h at 65 °C. After being cooled to room temperature, the mixture was evaporated under reduced pressure. The residue was purified by column chromatography to give **10** or ent-**10** (1.4 g, 62% for 2 steps) as a yellow solid. ^1^H NMR (400 MHz, CDCl_3_) δ 8.18 (s, 1H), 7.15 (s, 1H), 6.16 (s, 2H), 6.02 (d, *J* = 3.2 Hz, 1H), 5.21 (q, *J* = 3.4 Hz, 1H), 4.99 (q, *J* = 3.4 Hz, 1H), 4.24 (q, *J* = 4.3 Hz, 1H), 3.12–3.03 (m, 2H), 2.01 (s, 1H), 1.58 (s, 3H), 1.34 (s, 3H); ^13^C NMR (100 MHz, CDCl_3_) δ 157.3, 152.1, 149.6, 127.9, 114.9, 104.7, 91.2, 85.5, 84.0, 81.5, 51.1, 43.2, 27.4, 25.5; MS (ESI) *m*/*z* 432.0 [M + H]^+^.

#### 3.1.6. General Procedure for Sonogashira Coupling for the Preparation of **11a**,**b**

To a microwave vial equipped with a septum, containing starting material, CuI (25 mol%), and PdCl_2_(PPh_3_)_2_ (10 mol %), a degassed mixture of DMF/Et_3_N (4:1) was added. The resulting mixture was degassed with nitrogen for 5 min before adding corresponding alkynes (2.0 equiv), and heated in a microwave for 1 h at 50 °C. Then the mixture was extracted with EtOAc thrice, the organic phase was washed with brine, and the combined organic layers were dried over anhydrous MgSO_4_, filtered, and evaporated under reduced pressure. The residue is used in the next step without further purification.

#### 3.1.7. General Procedure for Suzuki Coupling for the Preparation of **12a**–**d** and ent-**12d**

In a microwave vial, starting material (1 equiv), corresponding boronic ester (1.2 equiv), PdCl_2_(PPh_3_)_2_ (6 mol %) and sodium carbonate (2 equiv) were taken, and the vial was sealed with a septum. To this mixture was added degassed DMF/H_2_O (0.14 M/0.36 M), and the reaction mixture was heated in a microwave at 70 °C. The reaction was quenched after 1 h with water and extracted with EtOAc thrice. The organic phase was washed with brine, and the combined organic layers were dried over anhydrous MgSO_4_, filtered, and evaporated under reduced pressure. The residue was purified by column chromatography on silica to give **12a**–**12d** and ent-**12d**.

##### 5-([1,1′-Biphenyl]-4-yl)-7-((3a*R*,4*R*,6*R*,6a*R*)-6-(aminomethyl)-2,2-dimethyltetrahydrofuro[3,4-*d*][1,3]dioxol-4-yl)-7*H*-pyrrolo[2,3-*d*]pyrimidin-4-amine (**12a**)

98.8 mg (45%); yellow solid; ^1^H NMR (400 MHz, CD_3_OD) δ 8.18 (s, 1H), 7.73 (d, *J* = 8.2 Hz, 2H), 7.66 (d, *J* = 7.3 Hz, 2H), 7.56 (d, *J* = 8.2 Hz, 2H), 7.45 (t, *J* = 7.8 Hz, 2H), 7.38–7.33 (m, 2H), 7.27–7.19 (m, 2H), 6.27 (d, *J* = 3.2 Hz, 1H), 5.34 (q, *J* = 3.4 Hz, 1H), 4.95 (q, *J* = 3.5 Hz, 1H), 4.21–4.17 (m, 1H), 2.99–2.91 (m, 2H), 1.61 (s, 3H), 1.39 (d, *J* = 9.6 Hz, 3H); ^13^C NMR (100 MHz, CD_3_OD) δ 158.9, 152.8, 151.8, 141.7, 141.6, 134.4, 130.4, 130.3, 130.0, 128.6, 127.9, 124.5, 123.0, 121.3, 119.0, 115.8, 102.7, 91.3, 86.9, 85.3, 83.1, 44.5, 27.5, 25.6; MS (ESI) *m*/*z* 458.2165 [M + H]^+^.

##### 7-((3a*R*,4*R*,6*R*,6a*R*)-6-(aminomethyl)-2,2-dimethyltetrahydrofuro[3,4-*d*][1,3]dioxol-4-yl)-5-(1*H*-indol-2-yl)-7*H*-pyrrolo[2,3-*d*]pyrimidin-4-amine (**12b**)

132 mg (65%); yellow solid; ^1^H NMR (400 MHz, CD_3_OD) δ 8.18 (s, 1H), 7.56 (d, *J* = 7.8 Hz, 1H), 7.50 (s, 1H), 7.41 (d, *J* = 7.8 Hz, 1H), 7.22 (dd, *J* = 19.4, 8.0 Hz, 1H), 7.14 (t, *J* = 7.1 Hz, 1H), 7.05 (t, *J* = 7.1 Hz, 1H), 6.27 (d, *J* = 3.2 Hz, 1H), 5.33 (q, *J* = 3.4 Hz, 1H), 4.95 (q, *J* = 3.5 Hz, 1H), 4.22–4.18 (m, 1H), 2.97–2.95 (m, 2H), 1.61 (s, 3H), 1.38 (s, 3H); ^13^C NMR (100 MHz, CD_3_OD) δ 159.0, 153.0, 151.5, 138.6, 132.6, 130.6, 130.2, 123.3, 122.9, 121.1, 120.8, 115.9, 112.1, 111.0, 102.7, 102.3, 91.3, 87.0, 85.3, 83.1, 44.5, 27.5, 25.6; MS (ESI) *m*/*z* 422.2 [M + H]^+^.

##### 7-((3a*R*,4*R*,6*R*,6a*R*)-6-(Aminomethyl)-2,2-dimethyltetrahydrofuro[3,4-*d*][1,3]dioxol-4-yl)-5-(3,5-dimethoxyphenyl)-7*H*-pyrrolo[2,3-*d*]pyrimidin-4-amine (**12c**)

115 mg (63%); yellow solid; ^1^H NMR (400 MHz, CDCl_3_) δ 8.27 (s, 1H), 7.05 (s, 1H), 6.58 (d, *J* = 2.3 Hz, 2H), 6.45 (t, *J* = 2.3 Hz, 1H), 6.17 (d, *J* = 3.7 Hz, 1H), 5.49 (s, 2H), 5.31 (q, *J* = 3.4 Hz, 1H), 4.96 (q, *J* = 3.7 Hz, 1H), 4.18 (q, *J* = 4.7 Hz, 1H), 3.80 (s, 6H), 3.05 (dd, *J* = 13.3, 4.1 Hz, 1H), 2.96 (q, *J* = 6.4 Hz, 1H), 1.89 (s, 2H), 1.60 (s, 3H), 1.36 (s, 3H); ^13^C NMR (100 MHz, CDCl_3_) δ 161.3, 157.3, 152.4, 150.7, 136.3, 121.2, 117.6, 114.7, 106.9, 102.1, 99.5, 90.5, 86.3, 84.1, 81.6, 55.5, 43.9, 27.4, 25.5; MS (ESI) *m*/*z* 442.2098 [M + H]^+^.

##### 7-((3a*R*,4*R*,6*R*,6a*R*)-6-(Aminomethyl)-2,2-dimethyltetrahydrofuro[3,4-*d*][1,3]dioxol-4-yl)-5-(7-methoxy-5-methylbenzo[*b*]thiophen-2-yl)-7*H*-pyrrolo[2,3-*d*]pyrimidin-4-amine (**12d**) and 7-((3a*S*,4*S*,6*S*,6a*S*)-6-(Aminomethyl)-2,2-dimethyltetrahydrofuro[3,4-*d*][1,3]dioxol-4-yl)-5-(7-methoxy-5-methylbenzo[*b*]thiophen-2-yl)-7*H*-pyrrolo[2,3-*d*]pyrimidin-4-amine (ent-**12d**)

152mg (56%); yellow solid; ^1^H NMR (400 MHz, CDCl_3_) δ 8.28 (s, 1H), 7.21 (s, 1H), 7.19 (s, 2H), 6.61 (s, 1H), 6.18 (d, *J* = 3.2 Hz, 1H), 5.83 (s, 2H), 5.30 (q, *J* = 3.2 Hz, 1H), 4.95 (q, *J* = 3.7 Hz, 1H), 4.17 (q, *J* = 4.6 Hz, 1H), 3.96 (s, 3H), 3.04 (dd, *J* = 13.3, 4.1 Hz, 1H), 2.94 (dd, *J* = 13.2, 5.6 Hz, 1H), 2.45 (s, 3H), 1.75 (s, 2H), 1.60 (s, 3H), 1.36 (s, 3H); ^13^C NMR (100 MHz, CDCl_3_) δ 157.3, 153.8, 152.6, 150.7, 142.0, 136.4, 136.4, 125.7, 122.9, 122.8, 116.1, 114.7, 110.1, 106.3, 102.1, 90.4, 86.6, 84.2, 81.6, 55.7, 43.9, 27.3, 25.5, 22.0; MS (ESI) *m*/*z* 482.1868 [M + H]+.

#### 3.1.8. General Procedure for EDC Coupling and Acetonide Deprotection for the Preparation of **13a**–**f** and ent-**13f**

To a solution of the starting material in anhydrous DMF was added the corresponding acrylic acid derivative (1.8 eq), EDC·HCl (2.0 eq), and DMAP (0.3 eq) sequentially at 0 °C. The reaction mixture was stirred at the same temperature for 2 h. Upon completion, the reaction was quenched with saturated aqueous ammonium chloride solution and diluted with ethyl acetate. The organic layer was extracted with ethyl acetate 3 times, washed with brine, dried over MgSO_4_, and concentrated under reduced pressure to afford the crude product, which was used in the next step without further purification. To a solution of starting material in THF was added 50% aqueous formic acid solution, and the resulting mixture was stirred for 2 days at room temperature. Acidic solution was basified using a weakly basic anion-exchange resin (Dowex^®^ 66 free base) and stirred for an additional 1 h, filtered, and concentrated under reduced pressure. The residue was purified by silica gel column chromatography to give **13a**–**f** and ent-**13f**.

##### *N*-(((2*R*,3*S*,4*R*,5*R*)-5-(4-Amino-5-((3,5-dimethoxyphenyl)ethynyl)-7*H*-pyrrolo[2,3-*d*]pyrimidin-7-yl)-3,4-dihydroxytetrahydrofuran-2-yl)methyl)acrylamide (**13a**)

25.3 mg, (25%); white solid; ^1^H NMR (400 MHz, CD_3_OD) δ 8.16 (s, 1H), 7.61 (s, 1H), 6.68 (d, *J* = 2.3 Hz, 2H), 6.52 (t, *J* = 2.1 Hz, 1H), 6.34–6.23 (m, 2H), 6.05 (d, *J* = 5.9 Hz, 1H), 5.69 (dd, *J* = 8.9, 3.0 Hz, 1H), 4.58 (t, *J* = 5.3 Hz, 1H), 4.19–4.13 (m, 2H), 3.80 (s, 6H), 3.72–3.59 (m, 2H); ^13^C NMR (100 MHz, CD_3_OD) δ 168.4, 162.3, 159.2, 153.6, 150.8, 131.9, 128.7, 127.2, 125.5, 110.1, 104.6, 102.4, 97.5, 92.9, 90.6, 84.5, 82.4, 75.2, 72.9, 55.9, 42.5; HRMS (ESI) found 480.1880 [calcd for C_24_H_26_N_5_O_6_^+^ (M + H)^+^ 480.1878]; HPLC purity: 98.83%.

##### *N*-(((2*R*,3*S*,4*R*,5*R*)-5-(4-Amino-5-((3-methoxy-5-methylphenyl)ethynyl)-7*H*-pyrrolo[2,3-*d*]pyrimidin-7-yl)-3,4-dihydroxytetrahydrofuran-2-yl)methyl)acrylamide (**13b**)

25 mg, 18%; white solid; ^1^H NMR (400 MHz, DMSO-*d*_6_) δ 8.35 (t, *J* = 5.7 Hz, 1H), 8.17 (s, 1H), 7.88 (s, 1H), 6.99 (s, 1H), 6.95 (s, 1H), 6.82 (s, 1H), 6.28 (dd, *J* = 16.9, 10.1 Hz, 1H), 6.11 (dd, *J* = 17.2, 2.1 Hz, 1H), 6.05 (d, *J* = 5.9 Hz, 1H), 5.60 (dd, *J* = 10.1, 2.3 Hz, 1H), 5.42 (d, *J* = 6.4 Hz, 1H), 5.26 (d, *J* = 5.0 Hz, 1H), 4.44 (q, *J* = 5.8 Hz, 1H), 4.03 (q, *J* = 4.4 Hz, 1H), 3.94–3.90 (m, 1H), 3.77 (s, 3H), 3.56–3.52 (m, 1H), 3.39 (q, *J* = 6.9 Hz, 1H), 2.30 (s, 3H); ^13^C NMR (100 MHz, DMSO-*d*_6_) δ 164.8, 159.2, 157.6, 152.9, 150.1, 139.6, 131.6, 127.5, 125.5, 124.2, 123.3, 115.6, 113.2, 102.2, 95.0, 91.3, 87.0, 82.8, 82.5, 73.3, 71.3, 55.2, 41.3, 20.9; HRMS (ESI) found 464.1924 [calcd for C_24_H_26_N_5_O_5_^+^ (M + H)^+^ 464.1928]; HPLC purity: 99.84%.

##### *N*-(((2*R*,3*S*,4*R*,5*R*)-5-(5-([1,1′-Biphenyl]-4-yl)-4-amino-7*H*-pyrrolo[2,3-*d*]pyrimidin-7-yl)-3,4-dihydroxytetrahydrofuran-2-yl)methyl)acrylamide (**13c**)

21 mg, (21%); white solid; ^1^H NMR (400 MHz, CD_3_OD) δ 8.18 (s, 1H), 7.75 (d, *J* = 8.3 Hz, 2H), 7.68 (d, *J* = 7.4 Hz, 2H), 7.58 (d, *J* = 8.3 Hz, 2H), 7.46 (t, *J* = 7.6 Hz, 2H), 7.37–7.34 (m, 2H), 6.32–6.20 (m, 2H), 6.15 (d, *J* = 6.0 Hz, 1H), 5.65 (dd, *J* = 9.2, 2.8 Hz, 1H), 4.62 (t, *J* = 5.5 Hz, 1H), 4.21–4.13 (m, 2H), 3.66 (ddd, *J* = 32.9, 14.2, 4.8 Hz, 2H); ^13^C NMR (100 MHz, CD_3_OD) δ 168.4, 158.9, 152.6, 151.9, 141.7, 141.5, 134.6, 131.9, 130.4, 130.0, 128.6, 128.6, 127.9, 127.2, 122.8, 118.7, 102.8, 90.2, 84.3, 75.1, 72.9, 42.5; HRMS (ESI) found 472.1966 [calcd for C_26_H_26_N_5_O_4_^+^ (M + H)^+^ 472.1979]; HPLC purity: 96.90%.

##### *N*-(((2*R*,3*S*,4*R*,5*R*)-5-(4-Amino-5-(1*H*-indol-2-yl)-7*H*-pyrrolo[2,3-*d*]pyrimidin-7-yl)-3,4-dihydroxytetrahydrofuran-2-yl)methyl)acrylamide (**13d**)

30 mg (23%); white solid; ^1^H NMR (400 MHz, CD_3_OD) δ 8.19 (s, 1H), 7.57 (d, *J* = 7.8 Hz, 1H), 7.50 (s, 1H), 7.41 (d, *J* = 7.8 Hz, 1H), 7.14 (t, *J* = 6.9 Hz, 1H), 7.05 (t, *J* = 7.1 Hz, 1H), 6.58 (s, 1H), 6.32–6.20 (m, 2H), 6.15 (d, *J* = 5.5 Hz, 1H), 5.63 (dd, *J* = 8.9, 3.0 Hz, 1H), 4.62 (t, *J* = 5.5 Hz, 1H), 4.21–4.14 (m, 2H), 3.67 (ddd, *J* = 39.6, 14.2, 4.8 Hz, 2H); ^13^C NMR (100 MHz, DMSO-*d*_6_) δ 164.8, 157.5, 152.2, 150.9, 136.7, 132.2, 131.6, 128.8, 125.5, 121.6, 121.3, 119.8, 119.4, 111.2, 108.4, 100.5, 100.4, 87.0, 82.5, 73.4, 71.4, 41.4; HRMS (ESI) found 435.1771 [calcd for C_22_H_23_N_6_O_4_^+^ (M + H)^+^ 435.1775]; HPLC purity: 99.90%.

##### *N*-(((2*R*,3*S*,4*R*,5*R*)-5-(4-Amino-5-(3,5-dimethoxyphenyl)-7*H*-pyrrolo[2,3-*d*]pyrimidin-7-yl)-3,4-dihydroxytetrahydrofuran-2-yl)methyl)acrylamide (**13e**)

15 mg (13%); white solid; ^1^H NMR (400 MHz, DMSO-*d*_6_) δ 8.34 (t, *J* = 5.7 Hz, 1H), 8.16 (s, 1H), 7.53 (s, 1H), 6.64 (d, *J* = 2.3 Hz, 2H), 6.51 (t, *J* = 2.1 Hz, 1H), 6.27 (dd, *J* = 17.2, 10.3 Hz, 1H), 6.12–6.07 (m, 2H), 5.57 (dd, *J* = 10.1, 2.3 Hz, 1H), 5.38 (d, *J* = 6.4 Hz, 1H), 5.23 (d, *J* = 5.1 Hz, 1H), 4.47 (q, *J* = 5.8 Hz, 1H), 4.05 (q, *J* = 4.7 Hz, 1H), 3.92 (dd, *J* = 10.3, 4.4 Hz, 1H), 3.80 (s, 6H), 3.54 (dt, *J* = 13.9, 5.3 Hz, 1H), 3.39 (q, *J* = 6.7 Hz, 1H); ^13^C NMR (100 MHz, DMSO-*d*_6_) δ 164.8, 160.8, 157.2, 151.9, 151.0, 136.3, 131.6, 125.4, 121.2, 116.6, 106.5, 100.3, 98.9, 86.8, 82.5, 73.2, 71.3, 55.3, 41.3; HRMS (ESI) found 456.1886 [calcd for C_22_H_26_N_5_O_6_^+^ (M + H)^+^ 456.1878].

##### *N*-(((2*R*,3*S*,4*R*,5*R*)-5-(4-Amino-5-(7-methoxy-5-methylbenzo[*b*]thiophen-2-yl)-7*H*-pyrrolo[2,3-*d*]pyrimidin-7-yl)-3,4-dihydroxytetrahydrofuran-2-yl)methyl)acrylamide (**13f**)

32 mg (21%); white solid; ^1^H NMR (400 MHz, DMSO-*d*_6_) δ 8.36 (s, 1H), 8.20 (s, 1H), 7.71 (s, 1H), 7.36 (s, 1H), 7.29 (s, 1H), 6.81 (s, 1H), 6.52 (s, 1H), 6.28 (dd, *J* = 17.0, 10.1 Hz, 1H), 6.13–6.08 (m, 2H), 5.58 (d, *J* = 10.1 Hz, 1H), 5.42 (d, *J* = 6.0 Hz, 1H), 5.25 (d, *J* = 4.6 Hz, 1H), 4.51 (d, *J* = 5.5 Hz, 1H), 4.05 (d, *J* = 3.7 Hz, 1H), 3.96 (s, 4H), 3.58–3.55 (m, 1H), 3.39 (d, *J* = 6.9 Hz, 1H), 2.44 (s, 3H); ^13^C NMR (100 MHz, DMSO-*d*_6_) δ 164.8, 157.3, 153.4, 152.3, 151.1, 141.9, 136.2, 135.9, 131.6, 125.5, 124.4, 122.9, 122.7, 116.1, 109.2, 106.5, 100.5, 86.9, 82.8, 73.1, 71.3, 55.7, 41.2, 21.6; HRMS (ESI) found 496.1638 [calcd for C_24_H_26_N_5_O_5_S^+^ (M + H)^+^ 496.1649]; HPLC purity: 96.91%.

##### *N*-(((2*S*,3*R*,4*S*,5*S*)-5-(4-Amino-5-(7-methoxy-5-methylbenzo[*b*]thiophen-2-yl)-7*H*-pyrrolo[2,3-*d*]pyrimidin-7-yl)-3,4-dihydroxytetrahydrofuran-2-yl)methyl)acrylamide (ent-**13f**)

20 mg (17%); white solid; ^1^H NMR (400 MHz, DMSO-*d*_6_) δ 8.36 (t, *J* = 5.7 Hz, 1H), 8.20 (s, 1H), 7.71 (s, 1H), 7.35 (s, 1H), 7.29 (s, 1H), 6.81 (s, 1H), 6.51 (s, 1H), 6.27 (dd, *J* = 17.4, 10.1 Hz, 1H), 6.12–6.07 (m, 2H), 5.58 (dd, *J* = 10.1, 2.3 Hz, 1H), 5.43 (d, *J* = 6.4 Hz, 1H), 5.26 (d, *J* = 4.6 Hz, 1H), 4.50 (q, *J* = 5.8 Hz, 1H), 4.05 (q, *J* = 4.4 Hz, 1H), 3.95 (s, 3H), 3.93–3.91 (m, 1H), 3.40–3.35 (m, 1H), 3.39 (t, *J* = 6.6 Hz, 1H), 2.44 (s, 3H); ^13^C NMR (100 MHz, DMSO-*d*_6_) δ 164.8, 157.3, 153.4, 152.3, 151.1, 141.9, 136.2, 135.9, 131.6, 125.5, 124.4, 122.9, 122.6, 116.1, 109.2, 106.5, 100.5, 86.9, 82.7, 73.1, 71.3, 55.7, 41.2, 21.6; MS (ESI) *m*/*z* 496.1777 [M + H]^+^.

#### 3.1.9. Synthesis of 5′-Acrylamide-7-substituted-7-deaza-4′-thioadenosine Derivatives (**19a**–**e**)

##### ((3a*S*,4*R*,6*R*,6a*R*)-6-(4-Amino-5-iodo-7*H*-pyrrolo[2,3-*d*]pyrimidin-7-yl)-2,2-dimethyltetrahydrothieno[3,4-*d*][1,3]dioxol-4-yl)methanol (**15**)

To a solution of **14** (4.6 g, 6.7 mmol) in THF (100 mL) was added a solution of 1.0 M TBAF in THF (10.1 mL, 10.1 mmol) at room temperature under N_2_. After being stirred for 40 min at room temperature, the reaction mixture was quenched with aqueous ammonium chloride solution (10 mL). Then the mixture was evaporated under reduced pressure and extracted with EtOAc thrice, and the organic phase was washed with brine, and the combined organic layers were dried over anhydrous MgSO_4_, filtered, and evaporated under reduced pressure. The residue was purified by column chromatography on silica to give **15** as a white solid (2.8 g, 95%). ^1^H NMR (400 MHz, CDCl_3_) δ 8.25 (s, 1H), 7.17 (s, 1H), 5.98 (t, *J* = 4.0 Hz, 1H), 5.91 (d, *J* = 16.5 Hz, 2H), 5.27 (q, *J* = 4.3 Hz, 1H), 4.95 (d, *J* = 5.0 Hz, 1H), 4.01 (dd, *J* = 12.3, 4.1 Hz, 1H), 3.87 (d, *J* = 8.2 Hz, 2H), 1.62 (s, 3H), 1.33 (s, 3H); ^13^C NMR (100 MHz, CDCl_3_) δ 157.4, 151.8, 149.1, 129.3, 112.1, 105.6, 88.7, 85.7, 71.4, 65.1, 54.9, 50.1, 28.1, 25.5;

##### 7-((3a*R*,4*R*,6*R*,6a*S*)-6-(Aminomethyl)-2,2-dimethyltetrahydrothieno[3,4-*d*][1,3]dioxol-4-yl)-5-iodo-7*H*-pyrrolo[2,3-*d*]pyrimidin-4-amine (**16**)

To the dry THF (150 mL) at 0 °C was added at PPh_3_ (4.1 g, 15.6 mmol) under N_2_. The solution was allowed to stir for 10 min, and DIAD (3.1 mL, 15.6 mmol) was added dropwise. The resulting mixture was stirred for an additional time until it became a white suspension. The ice bath was removed, and to the warming solution was added DPPA (3.4 mL, 15.6 mmol) over a period of 5 min, followed by the addition of **15** (2.8 g, 6.25 mmol), which was dissolved in dry THF (45 mL). The reaction mixture was stirred for 17 h at room temperature. Then the mixture was quenched with H_2_O (10 mL) and extracted with EtOAc thrice. The organic phase was washed with brine, and the combined organic layers were dried over anhydrous MgSO_4_, filtered, and evaporated under reduced pressure to give crude products, and were used in the next step without further purification. To a solution of crude products in dry THF (100 mL) was added PPh_3_ (4.1 g, 15.6 mmol), and stirred for 17 h at room temperature under N_2_. Then the reaction mixture was added ammonium hydroxide (2 mL) and stirred for 2 h at 65 °C. After being cooled to room temperature, the mixture was evaporated under reduced pressure. The residue was purified by column chromatography to give **16** as a yellow solid (1.3 g, 47% for 2 steps). ^1^H NMR (400 MHz, CDCl_3_) δ 8.18 (d, *J* = 5.5 Hz, 1H), 7.30 (s, 1H), 6.48 (s, 2H), 6.22 (d, *J* = 3.2 Hz, 1H), 5.25 (s, 2H), 5.10 (q, *J* = 3.0 Hz, 1H), 4.89 (q, *J* = 3.0 Hz, 1H), 3.73–3.69 (m, 1H), 3.13 (d, *J* = 22.4 Hz, 2H), 1.59 (s, 3H), 1.30 (s, 3H); ^13^C NMR (100 MHz, CDCl_3_) δ 157.2, 151.4, 149.8, 127.5, 113.2, 104.5, 88.8, 85.3, 55.7, 51.3, 44.5, 27.6, 25.4;

#### 3.1.10. Procedure for Sonogashira Coupling for the Preparation of **17**

To a microwave vial equipped with a septum, containing **16**, CuI (25 mol%), and PdCl_2_(PPh_3_)_2_ (10 mol %) was added a degassed mixture of DMF/Et_3_N (4:1). The resulting mixture was degassed with nitrogen for 5 min before adding corresponding alkynes (2.0 equiv) and heated in microwave for 1 h at 50 °C. Then the mixture was extracted with EtOAc thrice, and the organic phase was washed with brine, and the combined organic layers were dried over anhydrous MgSO_4_, filtered, and evaporated under reduced pressure. The residue is used in the next step without further purification.

#### 3.1.11. General Procedure for Suzuki Coupling for the Preparation of **18a**–**d**

In a microwave vial, starting material (1 equiv), corresponding boronic ester (1.2 equiv), PdCl_2_(PPh_3_)_2_ (6 mol %), and sodium carbonate (2 equiv) were taken, and the vial was sealed with a septum. To this mixture was added degassed DMF/H_2_O (0.14 M/0.36 M), and the reaction mixture was heated in a microwave at 70 °C. The reaction was quenched after 1 h with water and extracted with EtOAc thrice. The organic phase was washed with brine, and the combined organic layers were dried over anhydrous MgSO_4_, filtered, and evaporated under reduced pressure. The residue is used in the next step without further purification.

#### 3.1.12. General Procedure for EDC Coupling and Acetonide Deprotection for the Preparation of **19a**–**e**

To a solution of the starting material in anhydrous DMF was added the corresponding acrylic acid derivative (1.8 eq), EDC·HCl (2.0 eq), and DMAP (0.3 eq) sequentially at 0 °C. The reaction mixture was stirred at the same temperature for 2 h. Upon completion, the reaction was quenched with saturated aqueous ammonium chloride solution and diluted with ethyl acetate. The organic layer was extracted with ethyl acetate 3 times, washed with brine, dried over MgSO_4_, and concentrated under reduced pressure to afford the crude product, which was used in the next step without further purification. To a solution of starting material in THF was added 50% aqueous formic acid solution, and the resulting mixture was stirred for 2 days at room temperature. Acidic solution was basified using a weakly basic anion-exchange resin (Dowex^®^ 66 free base) and stirred for an additional 1 h, filtered, and concentrated under reduced pressure. The residue was purified by silica gel column chromatography to give **19a**–**19e**.

##### *N*-(((2*R*,3*S*,4*R*,5*R*)-5-(4-Amino-5-((3,5-dimethoxyphenyl)ethynyl)-7*H*-pyrrolo[2,3-*d*]pyrimidin-7-yl)-3,4-dihydroxytetrahydrothiophen-2-yl)methyl)acrylamide (**19a**)

24 mg (25%); white solid; ^1^H NMR (400 MHz, DMSO-*d*_6_) δ 8.49 (t, *J* = 5.7 Hz, 1H), 8.16 (s, 1H), 8.03 (s, 1H), 6.77 (d, *J* = 1.8 Hz, 2H), 6.55 (t, *J* = 2.1 Hz, 1H), 6.28 (dd, *J* = 17.0, 10.1 Hz, 1H), 6.14–6.10 (m, 2H), 5.62 (dd, *J* = 10.1, 1.8 Hz, 1H), 5.53 (d, *J* = 6.4 Hz, 1H), 5.43 (d, *J* = 4.1 Hz, 1H), 4.55 (td, *J* = 6.8, 3.4 Hz, 1H), 4.11 (d, *J* = 2.8 Hz, 1H), 3.78 (s, 6H), 3.71–3.59 (m, 1H), 3.48–3.42 (m, 1H); ^13^C NMR (100 MHz, DMSO-*d*_6_) δ 164.9, 160.4, 157.5, 152.9, 150.4, 131.6, 127.7, 125.7, 124.1, 108.9, 101.9, 101.4, 94.9, 91.3, 82.8, 77.0, 74.1, 61.3, 55.4, 50.7, 43.0; HRMS (ESI) found 496.1659 [calcd for C_24_H_26_N_5_O_5_S^+^ (M + H)^+^ 496.1649].

##### *N*-(((2*R*,3*S*,4*R*,5*R*)-5-(4-Amino-5-(4-(1,1-dioxidothiomorpholino)phenyl)-7*H*-pyrrolo[2,3-*d*]pyrimidin-7-yl)-3,4-dihydroxytetrahydrothiophen-2-yl)methyl)acrylamide (**19b**)

35mg (25%); white solid; ^1^H NMR (400 MHz, DMSO-*d*_6_) δ 8.49 (t, *J* = 5.7 Hz, 1H), 8.14 (s, 1H), 7.53 (s, 1H), 7.37 (d, *J* = 8.2 Hz, 2H), 7.15 (d, *J* = 8.7 Hz, 2H), 6.30–6.08 (m, 4H), 5.60 (dd, *J* = 10.3, 2.1 Hz, 1H), 5.49 (d, *J* = 6.4 Hz, 1H), 5.39 (d, *J* = 4.6 Hz, 1H), 4.54 (td, *J* = 6.7, 3.5 Hz, 1H), 4.12 (q, *J* = 3.5 Hz, 1H), 3.85 (s, 4H), 3.68–3.61 (m, 1H), 3.46–3.39 (m, 1H), 3.15 (s, 4H); ^13^C NMR (100 MHz, DMSO-*d*_6_) δ 164.9, 157.3, 151.7, 151.2, 146.3, 131.6, 129.5, 125.6, 125.1, 120.1, 116.5, 116.0, 100.4, 77.0, 74.2, 61.1, 50.5, 49.7, 46.6, 42.7; HRMS (FAB) found 545.1633 [calcd for C_24_H_29_N_6_O_5_S2^+^ (M + H)^+^ 545.1641].

##### *N*-(((2*R*,3*S*,4*R*,5*R*)-5-(4-Amino-5-(furan-2-yl)-7*H*-pyrrolo[2,3-*d*]pyrimidin-7-yl)-3,4-dihydroxytetrahydrothiophen-2-yl)methyl)acrylamide (**19c**)

25 mg (21%); white solid; ^1^H NMR (400 MHz, DMSO-*d*_6_) δ 8.50 (t, *J* = 5.7 Hz, 1H), 8.14 (s, 1H), 7.94 (s, 1H), 7.79 (d, *J* = 0.9 Hz, 1H), 6.92 (s, 2H), 6.75 (d, *J* = 3.2 Hz, 1H), 6.63 (q, *J* = 1.7 Hz, 1H), 6.31–6.09 (m, 3H), 5.62 (dd, *J* = 10.1, 2.3 Hz, 1H), 5.52 (d, *J* = 6.9 Hz, 1H), 5.41 (d, *J* = 4.1 Hz, 1H), 4.53 (td, *J* = 6.8, 3.4 Hz, 1H), 4.12 (q, *J* = 3.5 Hz, 1H), 3.71–3.64 (m, 1H), 3.45 (q, *J* = 6.7 Hz, 1H); ^13^C NMR (100 MHz, DMSO-*d*_6_) δ 164.9, 157.2, 152.2, 151.3, 148.7, 141.9, 131.6, 125.7, 120.3, 112.0, 106.6, 105.3, 99.1, 77.0, 74.1, 61.1, 50.7, 42.9; HRMS (FAB) found 402.1231 [calcd for C_18_H_20_N_5_O_4_S^+^ (M + H)^+^ 402.1236].

##### *N*-(((2*R*,3*S*,4*R*,5*R*)-5-(4-Amino-5-(3,5-dimethoxyphenyl)-7*H*-pyrrolo[2,3-*d*]pyrimidin-7-yl)-3,4-dihydroxytetrahydrothiophen-2-yl)methyl)acrylamide (**19d**)

22 mg (18%); white solid; ^1^H NMR (400 MHz, DMSO-*d*_6_) δ 8.47 (t, *J* = 6.0 Hz, 1H), 8.15 (s, 1H), 7.67 (s, 1H), 6.63 (d, *J* = 2.3 Hz, 2H), 6.51 (t, *J* = 2.3 Hz, 1H), 6.30–6.20 (m, 2H), 6.10 (dd, *J* = 17.2, 2.1 Hz, 1H), 5.58 (dd, *J* = 10.1, 2.3 Hz, 1H), 5.49 (d, *J* = 6.0 Hz, 1H), 5.39 (d, *J* = 3.7 Hz, 1H), 4.55 (d, *J* = 3.2 Hz, 1H), 4.12 (d, *J* = 3.2 Hz, 1H), 3.81 (s, 6H), 3.68–3.62 (m, 1H), 3.49–3.40 (m, 1H), 3.37–3.30 (1H, overlapped with solvent peak); ^13^C NMR (100 MHz, DMSO-*d*_6_) δ 164.9, 160.8, 157.2, 151.8, 151.3, 136.3, 131.6, 125.6, 121.1, 116.7, 106.4, 100.1, 98.9, 77.0, 74.2, 61.1, 55.3, 50.6, 42.7; HRMS (ESI) found 472.1656 [calcd for C_22_H_26_N_5_O_5_S^+^ (M + H)^+^ 472.1649].

##### *N*-(((2*R*,3*S*,4*R*,5*R*)-5-(4-Amino-5-(7-methoxy-5-methylbenzo[*b*]thiophen-2-yl)-7*H*-pyrrolo[2,3-*d*]pyrimidin-7-yl)-3,4-dihydroxytetrahydrothiophen-2-yl)methyl)acrylamide (**19e**)

32 mg (23%); white solid; ^1^H NMR (400 MHz, DMSO-*d*_6_) δ 8.50 (t, *J* = 5.7 Hz, 1H), 8.19 (s, 1H), 7.84 (s, 1H), 7.35 (s, 1H), 7.29 (s, 1H), 6.81 (s, 1H), 6.51 (s, 2H), 6.31–6.21 (m, 2H), 6.11 (dd, *J* = 16.9, 1.8 Hz, 1H), 5.59 (dd, *J* = 10.1, 1.4 Hz, 1H), 5.53 (d, *J* = 6.4 Hz, 1H), 5.42 (d, *J* = 4.1 Hz, 1H), 4.59 (dd, *J* = 10.1, 6.9 Hz, 1H), 4.12 (d, *J* = 2.7 Hz, 1H), 3.96 (s, 3H), 3.67 (q, *J* = 6.6 Hz, 1H), 3.48–3.41 (m, 1H), 2.44 (s, 3H); ^13^C NMR (100 MHz, DMSO-*d*_6_) δ 164.9, 157.3, 153.4, 152.3, 151.4, 141.9, 136.3, 135.8, 131.6, 125.6, 124.4, 122.7, 122.5, 116.1, 109.3, 106.5, 100.2, 76.9, 74.1, 61.2, 55.7, 50.7, 42.8, 21.6; HRMS (ESI) found 512.1387 [calcd for C_24_H_26_N_5_O_4_S_2_^+^ (M + H)^+^ 512.1421]; HPLC purity: 97.14%.

#### 3.1.13. Synthesis of *N*-Methyl-5′-acrylamide-7-substituted-7-deaza Adenosine **21**

##### 7-((3a*R*,4*R*,6*R*,6a*R*)-2,2-Dimethyl-6-((methylamino)methyl)tetrahydrofuro[3,4-*d*][1,3]dioxol-4-yl)-5-(7-methoxy-5-methylbenzo[*b*]thiophen-2-yl)-7*H*-pyrrolo[2,3-*d*]pyrimidin-4-amine (**20**)

To a stirred solution of **9** (376 mg, 0.871 mmol) in CH_2_Cl_2_ (6 mL) at 0 °C was added Et_3_N (0.364 mL, 2.61 mmol), followed by methanesulfonyl chloride (0.101 mL, 1.31 mmol) dropwise. The solution was then allowed to warm to room temperature and then stirred for 1 h. Then the mixture was quenched with aqueous NaHCO_3_ solution (5 mL) and extracted with EtOAc thrice. The organic phase was washed with brine, and the combined organic layers were dried over anhydrous MgSO_4_, filtered, and evaporated under reduced pressure to give crude products, and were used in the next step without further purification. The crude product was dissolved in THF (3 mL) and transferred to a sealed tube. To this solution was added methylamine 2M in THF (excess). The tube was quickly sealed and heated to 70 °C for 24 h. The sealed tube containing the reaction mixture was cooled to room temperature, and the solvent was evaporated under reduced pressure to give crude products, and used in the next step without further purification. In a microwave vial, starting material (1 equiv), corresponding boronic ester (1.2 equiv), PdCl_2_(PPh_3_)_2_ (6 mol %), and sodium carbonate (2 equiv) were taken, and the vial was sealed with a septum. To this mixture was added degassed DMF/H_2_O (0.14 M/0.36 M), and the reaction mixture was heated in a microwave at 70 °C. The reaction was quenched after 1 h with water and extracted with EtOAc thrice. The organic phase was washed with brine, and the combined organic layers were dried over anhydrous MgSO_4_, filtered, and evaporated under reduced pressure. The residue was purified by silica gel column chromatography to give **20** (198 mg, 46% for 3 steps) as yellow solid; ^1^H NMR (400 MHz, CDCl_3_) δ 8.32 (s, 1H), 7.22 (s, 1H), 7.21 (s, 1H), 7.21 (s, 1H), 6.64 (s, 1H), 6.09 (d, *J* = 3.2 Hz, 1H), 5.55 (s, 2H), 5.32 (t, *J* = 3.4 Hz, 1H), 5.11 (dd, *J* = 5.5, 3.7 Hz, 1H), 4.37 (q, *J* = 4.3 Hz, 1H), 4.00 (s, 3H), 3.10–2.93 (m, 2H), 2.52 (s, 3H), 2.49 (s, 3H), 1.62 (s, 3H), 1.37 (s, 3H); ^13^C NMR (100 MHz, CDCl_3_) δ 157.3, 154.0, 152.6, 150.4, 142.1, 136.6, 136.3, 125.9, 123.3, 123.2, 116.2, 115.0, 110.1, 106.4, 102.6, 91.8, 83.8, 83.7, 81.9, 55.8, 53.2, 36.3, 27.4, 25.5, 22.1;

##### (2*R*,3*R*,4*S*,5*R*)-2-(4-Amino-5-(7-methoxy-5-methylbenzo[*b*]thiophen-2-yl)-7*H*-pyrrolo[2,3-*d*]pyrimidin-7-yl)-5-((methylamino)methyl)tetrahydrofuran-3,4-diol (**21**)

To a solution of **20** (198 mg, 0.400 mmol) in THF was added 50% aqueous formic acid solution, and the resulting mixture was stirred for 2 days at room temperature. Acidic solution was basified using a weakly basic anion-exchange resin (Dowex^®^ 66 free base) and stirred for an additional 1 h, filtered, and concentrated under reduced pressure. The residue was purified by silica gel column chromatography to give **21** (44.8 mg, 22%) as a white solid. ^1^H NMR (400 MHz, DMSO-*d*_6_) δ 8.19 (s, 1H), 7.78 (d, *J* = 5.0 Hz, 1H), 7.35 (d, *J* = 2.7 Hz, 1H), 7.29 (d, *J* = 3.2 Hz, 1H), 6.86–6.71 (m, 2H), 6.54 (s, 2H), 6.16–6.00 (m, 2H), 5.68–5.28 (m, 3H), 4.57 (td, *J* = 10.7, 5.2 Hz, 1H), 4.09–4.00 (m, 2H), 3.95 (s, 3H), 3.84–3.51 (m, 2H), 3.05–2.89 (s each, 3H, -CH_3_ in amide), 2.44 (s, 3H); ^13^C NMR (100 MHz, DMSO-*d*_6_) δ 165.5, 157.2, 153.4, 152.2, 151.2, 151.2, 141.9, 136.2, 135.9, 128.6, 128.5, 127.4, 126.7, 125.8, 124.4, 124.4, 122.8, 122.7, 122.7, 116.2, 109.4, 106.5, 100.4, 100.3, 86.9, 86.6, 81.9, 81.8, 73.1, 72.9, 71.6, 71.1, 55.7, 51.4, 49.9, 40.5, 40.4, 40.2, 38.9, 36.4, 34.2, 21.6; HRMS (ESI) found 510.1821 [calcd for C_25_H_28_N_5_O_5_S^+^ (M + H)^+^ 510.1806]. Note: The NMR spectra exhibit signals corresponding to both cis-, trans- amide isomers, resulting in overlapping peaks. Attempts to resolve the individual isomer were unsuccessful due to rapid interconversion in solution.

#### 3.1.14. General Procedure for EDC Coupling and Acetonide Deprotection for the Preparation of **22a**–**e**

To a solution of the **12d** in anhydrous DMF was added the corresponding acrylic acid derivative (1.8 eq), EDC·HCl (2.0 eq), and DMAP (0.3 eq) sequentially at 0 °C. The reaction mixture was stirred at the same temperature for 2 h. Upon completion, the reaction was quenched with saturated aqueous ammonium chloride solution and diluted with ethyl acetate. The organic layer was extracted with ethyl acetate 3 times, washed with brine, dried over MgSO_4_, and concentrated under reduced pressure to afford the crude product, which was used in the next step without further purification. To a solution of starting material in THF was added 50% aqueous formic acid solution, and the resulting mixture was stirred for 2 days at room temperature. Acidic solution was basified using a weakly basic anion-exchange resin (Dowex^®^ 66 free base) and stirred for an additional 1 h, filtered, and concentrated under reduced pressure. The residue was purified by silica gel column chromatography to give **22a**–**22e**.

##### *N*-(((2*R*,3*S*,4*R*,5*R*)-5-(4-Amino-5-(7-methoxy-5-methylbenzo[*b*]thiophen-2-yl)-7*H*-pyrrolo[2,3-*d*]pyrimidin-7-yl)-3,4-dihydroxytetrahydrofuran-2-yl)methyl)propionamide (**22a**)

45 mg (50%); white solid; ^1^H NMR (400 MHz, DMSO-*d*_6_) δ 8.19 (s, 1H), 8.04 (t, *J* = 5.7 Hz, 1H), 7.70 (s, 1H), 7.35 (s, 1H), 7.29 (s, 1H), 6.81 (s, 1H), 6.51 (s, 2H), 6.08 (d, *J* = 6.4 Hz, 1H), 5.40 (d, *J* = 6.4 Hz, 1H), 5.21 (d, *J* = 5.0 Hz, 1H), 4.50 (q, *J* = 6.1 Hz, 1H), 4.01 (q, *J* = 4.6 Hz, 1H), 3.95 (s, 3H), 3.88 (dd, *J* = 9.1, 5.5 Hz, 1H), 2.44 (s, 3H), 2.10 (q, *J* = 7.9 Hz, 2H), 0.98 (t, *J* = 7.8 Hz, 3H); ^13^C NMR (100 MHz, DMSO-*d*_6_) δ 173.2, 157.4, 153.4, 152.3, 151.1, 141.9, 136.2, 135.9, 124.4, 123.0, 122.7, 116.1, 109.1, 106.5, 100.5, 87.0, 82.9, 73.0, 71.2, 55.7, 41.0, 40.1, 39.9, 39.7, 39.5, 39.3, 39.1, 38.9, 28.4, 21.6, 10.0; HRMS (ESI) found 498.1814 [calcd for C_24_H_28_N_5_O_5_S^+^ (M + H)^+^ 498.1806]; HPLC purity: 99.38%.

##### *N*-(((2*R*,3*S*,4*R*,5*R*)-5-(4-Amino-5-(7-methoxy-5-methylbenzo[*b*]thiophen-2-yl)-7*H*-pyrrolo[2,3-*d*]pyrimidin-7-yl)-3,4-dihydroxytetrahydrofuran-2-yl)methyl)methacrylamide (**22b**)

40 mg (24%); white solid; ^1^H NMR (400 MHz, DMSO-*d*_6_) δ 8.19 (s, 1H), 8.15 (t, *J* = 5.7 Hz, 1H), 7.71 (s, 1H), 7.35 (s, 1H), 7.29 (s, 1H), 6.81 (s, 1H), 6.57 (s, 2H), 6.09 (d, *J* = 6.4 Hz, 1H), 5.68 (s, 1H), 5.41 (d, *J* = 4.1 Hz, 1H), 5.33 (s, 1H), 5.23 (s, 1H), 4.51 (d, *J* = 3.2 Hz, 1H), 4.08 (s, 1H), 3.96 (d, *J* = 5.9 Hz, 4H), 3.51–3.45 (m, 1H), 3.37 (1H, overlapped with solvent peak), 2.44 (s, 3H), 1.85 (s, 3H); ^13^C NMR (100 MHz, DMSO-*d*_6_) δ 167.9, 157.1, 153.4, 152.0, 151.0, 141.9, 139.9, 136.1, 135.9, 124.5, 123.0, 122.7, 119.3, 116.2, 109.2, 106.5, 100.5, 86.9, 82.7, 73.1, 71.3, 55.7, 41.5, 40.2, 39.9, 21.6, 18.8; HRMS (ESI) found 510.1793 [calcd for C_25_H_28_N_5_O_5_S^+^ (M + H)^+^ 510.1806], found 510.1793.

##### *N*-(((2*R*,3*S*,4*R*,5*R*)-5-(4-Amino-5-(7-methoxy-5-methylbenzo[*b*]thiophen-2-yl)-7*H*-pyrrolo[2,3-*d*]pyrimidin-7-yl)-3,4-dihydroxytetrahydrofuran-2-yl)methyl)-2-fluoroacrylamide (**22c**)

30 mg (25%); white solid; ^1^H NMR (400 MHz, DMSO-*d*_6_) δ 8.75 (s, 1H), 8.19 (s, 1H), 7.72 (s, 1H), 7.35 (s, 1H), 7.29 (s, 1H), 6.81 (s, 1H), 6.54 (s, 2H), 6.09 (d, *J* = 6.4 Hz, 1H), 5.55 (dd, *J* = 48.0, 2.7 Hz, 1H), 5.44 (d, *J* = 6.4 Hz, 1H), 5.28–5.24 (m, 2H), 4.53 (d, *J* = 5.0 Hz, 1H), 4.07 (d, *J* = 2.3 Hz, 1H), 3.99–3.95 (m, 4H), 3.54–3.42 (m, 2H), 2.44 (s, 3H); ^13^C NMR (100 MHz, DMSO-*d*_6_) δ 159.2 (d, J_C,F_ = 31.7 Hz, C=O), 157.3, 156.4 (d, J_C,F_ = 268.6 Hz, CF), 153.4, 152.2, 151.1, 142.0, 136.2, 135.9, 124.5, 123.0, 122.7, 116.2, 109.2, 106.5, 100.5, 98.8, 87.0, 82.3, 73.0, 71.3, 55.7, 41.5, 40.1, 21.6; 19F-NMR (375 MHz, DMSO-*d*_6_) δ -117.4 (dd, *J* = 49 Hz, *J* = 17 Hz); HRMS (ESI) found 514.1548 [calcd for C_24_H_25_FN_5_O_5_S^+^ (M + H)^+^ 514.1555].

##### *N*-(((2*R*,3*S*,4*R*,5*R*)-5-(4-Amino-5-(7-methoxy-5-methylbenzo[*b*]thiophen-2-yl)-7*H*-pyrrolo[2,3-*d*]pyrimidin-7-yl)-3,4-dihydroxytetrahydrofuran-2-yl)methyl)but-2-ynamide (**22d**)

15 mg (18%); white solid; ^1^H NMR (400 MHz, DMSO-*d*_6_) δ 8.89 (t, *J* = 4.8 Hz, 1H), 8.21 (s, 1H), 7.71 (s, 1H), 7.36 (s, 1H), 7.29 (s, 1H), 6.81 (s, 1H), 6.55 (s, 2H), 6.07 (d, *J* = 6.4 Hz, 1H), 5.43 (d, *J* = 6.4 Hz, 1H), 5.27 (d, *J* = 4.6 Hz, 1H), 4.52 (q, *J* = 5.8 Hz, 1H), 4.01 (s, 1H), 3.95 (s, 4H), 3.40 (s, 2H), 2.43 (s, 3H), 1.93 (s, 3H); ^13^C NMR (100 MHz, DMSO-*d*_6_) δ 157.4, 153.4, 152.9, 152.2, 150.9, 142.0, 136.2, 135.9, 124.5, 123.2, 122.6, 116.2, 109.1, 106.5, 100.6, 87.3, 83.0, 82.7, 75.6, 73.0, 71.3, 55.7, 41.3, 40.2, 39.9, 21.6, 3.1; HRMS (ESI) found 508.1659 [calcd for C_25_H_26_N_5_O_5_S^+^ (M + H)^+^ 508.1649].

##### *N*-(((2*R*,3*S*,4*R*,5*R*)-5-(4-Amino-5-(7-methoxy-5-methylbenzo[*b*]thiophen-2-yl)-7*H*-pyrrolo[2,3-*d*]pyrimidin-7-yl)-3,4-dihydroxytetrahydrofuran-2-yl)methyl)-2-chloroacetamide (**22e**)

16 mg (19%); white solid; ^1^H NMR (400 MHz, DMSO-*d*_6_) δ 8.48 (t, *J* = 5.5 Hz, 1H), 8.21 (s, 1H), 7.74 (s, 1H), 7.36 (s, 1H), 7.29 (s, 1H), 6.81 (s, 1H), 6.59 (s, 2H), 6.11 (d, *J* = 6.4 Hz, 1H), 5.45 (d, *J* = 5.0 Hz, 1H), 5.29 (s, 1H), 4.51 (d, *J* = 5.0 Hz, 1H), 4.08 (s, 2H), 4.04 (s, 1H), 3.95 (s, 3H), 3.92 (s, 1H), 3.54–3.48 (m, 1H), 3.36 (1H, overlapped with solvent peak), 2.44 (s, 3H); ^13^C NMR (100 MHz, DMSO-*d*_6_) δ 166.3, 157.2, 153.4, 152.1, 151.1, 141.9, 136.1, 135.9, 124.5, 123.0, 122.8, 116.2, 109.3, 106.5, 100.4, 86.9, 82.6, 73.1, 71.2, 55.7, 42.7, 40.2, 21.6; HRMS (ESI) found 518.1265 [calcd for C_23_H_25_ClN_5_O_5_S^+^ (M + H)^+^ 518.1259].

#### 3.1.15. *N*-(((2*R*,3*S*,4*R*,5*R*)-5-(4-Amino-5-(7-methoxy-5-methylbenzo[*b*]thiophen-2-yl)-7*H*-pyrrolo[2,3-*d*]pyrimidin-7-yl)-3,4-dihydroxytetrahydrofuran-2-yl)methyl)ethenesulfonamide (**22f**)

To a solution of **12d** (42 mg, 0.084 mmol) in CH_2_Cl_2_ (5 mL) was added Et_3_N (0.02 mL, 0.252 mmol) at 0 °C and stirred for 10 min. Then the reaction mixture was added 2-chloroethanesulfonyl chloride (0.01 mL, 0.092 mmol) dropwise and stirred from 0 °C to room temperature for 17 h. Then the mixture was quenched with aqueous NaHCO_3_ (5 mL) and extracted with CH_2_Cl_2_ thrice. The organic phase was washed with brine, and the combined organic layers were dried over anhydrous MgSO_4_, filtered, and evaporated under reduced pressure to afford the crude product, which was used in the next step without further purification. To a solution of starting material in THF was added 50% aqueous formic acid solution, and the resulting mixture was stirred for 2 days at room temperature. Acidic solution was basified using a weakly basic anion-exchange resin (Dowex^®^ 66 free base) and stirred for an additional 1 h, filtered, and concentrated under reduced pressure. The residue was purified by silica gel column chromatography to give **22f** (7 mg, 24%); ^1^H NMR (400 MHz, DMSO-d_6_) δ 8.18 (s, 1H), 7.96 (t, *J* = 5.9 Hz, 1H), 7.72 (s, 1H), 7.35 (s, 1H), 7.28 (s, 1H), 6.81 (s, 1H), 6.72 (dd, *J* = 16.5, 10.1 Hz, 1H), 6.54 (s, 2H), 6.06 (s, 1H), 6.03 (d, *J* = 8.2 Hz, 1H), 5.94 (d, *J* = 10.1 Hz, 1H), 5.44 (d, *J* = 6.4 Hz, 1H), 5.27 (d, *J* = 4.6 Hz, 1H), 4.58 (q, *J* = 6.3 Hz, 1H), 4.09 (dd, *J* = 8.0, 4.8 Hz, 1H), 3.99–3.98 (m, 1H), 3.95 (s, 3H), 3.23–3.16 (m, 1H), 3.12–3.05 (m, 1H), 2.43 (s, 3H); ^13^C NMR (100 MHz, DMSO-d_6_) δ 157.4, 153.4, 152.1, 150.7, 141.9, 136.8, 136.1, 135.9, 125.5, 124.4, 123.5, 122.7, 116.2, 108.9, 106.5, 100.7, 87.7, 83.1, 72.7, 71.1, 55.7, 44.7, 21.6; HRMS (ESI) found 532.1312 [calcd for C_23_H_26_N_5_O_6_S_2_^+^ (M + H)^+^ 532.1319].

### 3.2. Biological and Computational Methods

#### 3.2.1. Kinase Inhibition Assay for FGFR1, FGFR2, FGFR3, and FGFR4

Kinase inhibition assays for FGFR1, FGFR2, FGFR3, and FGFR4 were conducted by Eurofins Cerep using their KinaseProfiler™ platform. Each kinase was assayed under optimized radiometric conditions specific to each isoform. All reactions were initiated by the addition of [γ-^33^P]-ATP at a concentration within 15 µM of the apparent K_M_ for ATP for each kinase, as summarized below: FGFR1 (200 µM), FGFR2 (90 µM), FGFR3 (15 µM), and FGFR4 (155 µM). Kinases were incubated in a buffer containing 20 mM MOPS (pH 7.0), 1 mM EDTA, 0.01% Brij-35, 5% glycerol, 0.1% β-mercaptoethanol, and 1 mg/mL BSA. Reactions were carried out in the presence of 10 mM magnesium acetate and peptide substrates specific to each kinase (proprietary sequences). The compounds were prepared in 100% DMSO and diluted to a final assay concentration with 2% DMSO in the reaction mixture. No pre-incubation step was included prior to reaction initiation. After incubation at room temperature for 40 min, reactions were terminated by the addition of phosphoric acid to a final concentration of 0.5%. An aliquot of each reaction was spotted onto phosphocellulose filters, washed four times with 0.425% phosphoric acid and once with ethanol, then dried prior to scintillation counting to determine radioactivity incorporation. All assays were performed in duplicate and included positive control wells (with DMSO but no test compound) and blank wells (containing a known reference inhibitor) to determine 0% kinase activity. Percent inhibition was calculated relative to these controls.

#### 3.2.2. Kinase Selectivity Profiling Assay

Kinase selectivity profiling was performed by Eurofins DiscoverX (San Diego, CA, USA) using the scanMAX™ platform, which screened the test compound against a panel of 468 human kinases, including wild-type and mutant variants. The assay was carried out at a final compound concentration of 100 μM. Kinases were expressed either as T7 phage display constructs in E. coli or as tagged proteins in HEK-293 cells. Streptavidin-coated magnetic beads were conjugated with biotinylated affinity ligands, and blocked with excess biotin and SeaBlock buffer. Binding reactions were assembled in 384-well polypropylene plates by combining kinases, ligand-coated beads, and test compounds in binding buffer (20% SeaBlock, 0.17× PBS, 0.05% Tween-20, 6 mM DTT), in a final volume of 20 μL. After 1 h incubation at room temperature with shaking, the beads were washed and eluted using a competitive ligand. The amount of kinase bound was quantified by real-time PCR. Assay results are reported as percent of control (%Ctrl), representing the level of kinase binding in the presence of compound relative to DMSO control (100% binding). Lower %Ctrl values indicate stronger kinase binding.

#### 3.2.3. FGFR1 Kinase Cloning and Protein Expression

The kinase domain of human FGFR1 (residues 458–765) was amplified from a cDNA clone (hMU003398) and subcloned into the pET-28a expression vector with an N-terminal hexahistidine (His_6_) tag and a thrombin cleavage site (LVPRGS). Full-length human PTP1B (residues 1–435, cDNA clone hMU000649) was cloned into the pGEX-4T-1 vector (GE Healthcare, Chicago, IL, USA) to facilitate protein purification. The resulting recombinant plasmids were co-transformed into *Escherichia coli* BL21-CodonPlus (DE3) RIPL cells. Cultures were grown in LB medium supplemented with 30 µg/mL kanamycin and 50 µg/mL ampicillin at 37 °C until OD_600_ reached 0.7–0.8. Protein expression was induced with 1 mM IPTG at 20 °C for 12 h. Cells were harvested by centrifugation (6000× *g*, 10 min, 4 °C).

#### 3.2.4. FGFR1 Protein Purification

Cell pellets were resuspended in lysis buffer A (20 mM Tris-HCl, 300 mM NaCl, 10 mM imidazole, 2 mM TCEP, pH 7.8) supplemented with PMSF, followed by sonication and centrifugation (35,000× *g*, 30 min, 4 °C). The clarified supernatant was filtered (0.45 µm) and loaded onto a Ni^2+^-charged HiTrap Chelating HP column (GE Healthcare). Bound proteins were eluted with a linear imidazole gradient using buffer B (20 mM Tris-HCl, 300 mM NaCl, 300 mM imidazole, 2 mM TCEP, pH 7.8). Fractions containing FGFR1 were buffer-exchanged into buffer C (20 mM Tris-HCl, 20 mM NaCl, 2 mM TCEP, pH 7.8) and incubated with thrombin to remove the His_6_ tag, followed by re-purification on the same Ni^2+^ column. Final polishing was performed by size-exclusion chromatography in buffer C. The purified FGFR1 kinase domain was concentrated to ~10 mg/mL, quantified using its extinction coefficient, and stored at −80 °C until use.

#### 3.2.5. FGFR1 Crystallization and Soaking Experiments

Purified FGFR1 was crystallized by the sitting-drop vapor diffusion method at 22 °C. Crystallization was performed using a reservoir solution containing L-proline, PEG 3350, and HEPES buffer. Protein crystals were soaked with compound **6h** (protein:ligand molar ratio of 1:10) for 3 days to obtain the co-crystal structure.

#### 3.2.6. Cell Culture

RT4 (from Korean Cell Line Bank, Seoul, Korea) and HCT116 (from ATCC, Manassas, VA, USA) cell lines were maintained in RPMI-1640 medium supplemented with 10% fetal bovine serum (FBS; Gibco, Thermo Fisher Scientific, Waltham, MA, USA) and 1% penicillin–streptomycin (Gibco, Thermo Fisher Scientific, Waltham, MA, USA). Cells were cultured at 37 °C in a humidified incubator ventilated with 5% CO_2_. Cells were subcultured upon reaching ~80% confluence.

#### 3.2.7. Cell Proliferation Assay Using IncuCyte Live-Cell Imaging

RT4 cells were seeded at a density of 5 × 10^4^ cells/mL, and HCT116 cells were seeded at 1 × 10^4^ cells/mL in 96-well plates (100 μL per well). After overnight incubation in incubator (37 °C, 5% CO_2_), cells were treated with the indicated compounds. IncuCyte ZOOM Live-Cell Imaging system (Essen BioScience, Ann Arbor, MI, USA) was used to monitor cell confluency in real time for up to 72 h.

#### 3.2.8. Cell Culture (SRB Assay)

Human liver cancer (SK-HEP-1), breast cancer (MDA-MB-231), lung cancer (A549), colorectal cancer (HCT116), and stomach cancer (SNU-638) were obtained from the American Type Culture Collection (ATCC, Manassas, VA, USA). Cells were cultured in medium (DMEM medium for SK-HEP-1 and MDA-MB-231; RPMI 1640 for A549, HCT116, and SNU-638 cells) supplemented with 10% FBS, 100 units/mL penicillin, 100 μg/mL streptomycin, and 0.25 μg/mL amphotericin B. Cells were incubated at 37 °C with 5% CO_2_ in a humidified atmosphere.

#### 3.2.9. Cell Proliferation Assay (SRB Assay)

Cell proliferation was assessed using the sulforhodamine B (SRB) assay. Cells were seeded in 96-well plates. For day-zero controls, cells were fixed 30 min after seeding. Treatment groups were exposed to test compounds at various concentrations for 72 h. After treatment, cells were fixed with cold 10% trichloroacetic acid (TCA) for 30 min at 4 °C, rinsed with distilled water, and air-dried. The fixed cells were stained with 0.4% (*w*/*v*) SRB dissolved in 1% (*v*/*v*) acetic acid for 2 h at room temperature. Unbound dye was removed by washing with 1% acetic acid, and the plates were again dried. The protein-bound dye was solubilized with 10 mM Tris base (pH 10.0) for 30 min, and absorbance was measured at 515 nm. Cell proliferation was determined as follows: cell proliferation (%) = (average absorbance_compound_ − average absorbance_day zero_)/(average absorbance_control_ − average absorbance_day zero_) × 100. IC_50_ values were calculated by nonlinear regression analysis using TableCurve 2D v5.01 (Systat Software Inc., Richmond, CA, USA).

#### 3.2.10. Intact Mass Analysis of FGFR1-Ligand Covalent Complexes

For intact mass analysis, 20 μL of FGFR1 protein solution (18 mg/mL) was incubated with the test compound at a 10-fold molar excess in 1 μL DMSO at 4 °C for 16 h. After incubation, the sample was centrifuged to remove any precipitates. Buffer exchange was performed using a Zeba™ Spin Desalting Column (7K MWCO, Thermo Scientific, Waltham, MA, USA) pre-equilibrated with 0.1% formic acid. The desalted protein solution was collected by centrifugation and analyzed using a Q-TOF 5600 mass spectrometer (AB Sciex, Framingham, MA, USA) to evaluate covalent adduct formation.

#### 3.2.11. Western Blot

Cells were lysed in cell lysis buffer (CLB) and incubated on ice for 1 h. The lysates were then centrifuged at 16,000× *g* for 15 min at 4 °C to remove cellular debris. Protein concentrations were determined using the Bradford assay, and all samples were normalized to equal protein concentrations by adding CLB. Proteins were denatured with 6X SDS Protein Loading Buffer at 100 °C and loaded to SDS–PAGE gel, followed by transfer into nitrocellulose (NC) membranes. Membranes were blocked with 5% skim milk in PBST (Phosphate-Buffered Saline with Tween 20) for 1 h at room temperature, washed three times with PBST, and incubated overnight at 4 °C with the appropriate primary antibodies. After three washes with PBST, membranes were incubated with horseradish peroxidase (HRP)-conjugated secondary antibodies for 1 h at room temperature. Protein bands were visualized using enhanced chemiluminescence (ECL) detection reagents.

#### 3.2.12. Molecular Dynamics Simulation

Molecular dynamics (MD) simulations were performed using the Desmond module within Schrödinger (ver. 14.2)’s Small Molecule Drug Discovery Suite (version 2024-4), executed on a Rocky Linux system equipped with an NVIDIA RTX A4000 GPU (NVIDIA, Santa Clara, CA, USA). The simulation was based on the docking model of compound **22f** bound to fibroblast growth factor receptor 4 (FGFR4, PDB ID: 4R6V). System preparation was carried out using the Desmond System Builder in Maestro. An orthorhombic simulation box was constructed with a 10 Å buffer in all directions around the protein–ligand complex. The system was solvated with the simple point charge (SPC) water model, and appropriate counter ions were added to neutralize the net charge. The OPLS4 force field was applied, and the simulation was conducted under an NPT ensemble, maintaining a temperature of 300.0 K and a pressure of 1.01325 bar. Prior to the production run, the system was relaxed using Desmond’s default relaxation protocol. Initial velocities were assigned randomly using a defined random seed. A 100-nanosecond MD simulation was then performed, with trajectory frames recorded every 100.0 ps, resulting in approximately 1000 frames. Post-simulation analysis was conducted using the Simulation Interactions Diagram (SID) module in Maestro to evaluate protein–ligand interactions and complex stability.

#### 3.2.13. CYP Inhibition Assay

All incubations were conducted at 37 °C in a water bath. Incubation mixtures were prepared in 100 mM potassium phosphate buffer and contained microsomes (0.253 mg/mL, 158 μL), test compound (at eight concentrations, 2.00 μL), a selective inhibitor (at 300 μM or 100 μM, 2.00 μL), a solvent (DMSO: MeOH (1:1, *v*:*v*) and DMSO: MeOH (1:9, *v*:*v*) were used as solvents for test compound and positive control inhibitors, respectively, 2.00 μL), and a cocktail of five probe substrates (20.0 μL). After a 10 min warm-up, cofactor (20.0 μL) was added to initiate the reaction. After 10 min of incubation, reactions were terminated by adding 400 µL of stop solution containing the internal standard. Samples were centrifuged at 3220× *g* for 20 min. The resulting supernatant was taken and diluted with purified water (HPLC grade) to lower the concentration of organic solvent prior to analysis. Sample plates were shaken at 800 rpm for 10 min to make the mixture homogeneous. Samples were injected for LC-MS/MS analysis for the metabolite of each substrate.

#### 3.2.14. In Vitro Microsomal Stability Assay

Human, mouse, and rat liver microsomes (0.5 mg/mL) diluted by 0.1 M potassium phosphate buffer (PH 7.4), three positive controls (testosterone 1 µM, diclofenac 1 µM, propafenone 1 µM), and test compound (1 µM concentration) were pre-incubated with liver microsomes for 10 min at 37 °C. NADPH was added and incubated for 0, 5, 15, 30, 45, 60 min at 37 °C. In order to terminate the reaction, acetonitrile containing internal standards (tolbutamide and labetalol) was added and centrifuged for 20 min (3220× *g*, 4 °C). The supernatant mixed with water was injected into the LC-MS/MS system to analyze the test compound. The disappearance of the test compound was analyzed using Shimadzu LC (Shimadzu, Kyoto, Japan) and SCIEX Triple Quad 6500+ (AB Sciex, Framingham, MA, USA). SPE Cartridge (1.5 × 5 mm, Optimize Technologies, Oregon City, OR, USA) was used. The mobile phase used contained 0.1% formic acid in water (A) and 0.1% formic acid and 2 mM ammonium acetate in water/acetonitrile (*v*:*v*, 10:90) (B). Test compound was quantified using MRM (multiple reaction monitoring). Experimental data were acquired using Analyst software (version 1.7.3) and processed using Discovery Quant software (version 3.0.1).

#### 3.2.15. In Vitro Plasma Metabolic Stability Assay

Frozen plasma was thawed under cold water for 10 to 20 min, and then centrifuged at 3220× *g* for 5 min. Mouse, rat, and human plasma, control compounds (propantheline bromide 2 μM, enalapril maleate salt 2 μM), and test compound (2 μM concentration) were incubated at 37.0 °C in a water bath. At corresponding time points (0, 10, 30, 60, 120 min), acetonitrile solution containing internal standards (tolbutamide and labetalol) was added to terminate the reaction, and centrifuged for 20 min (3220× *g*, 4 °C). The supernatant was injected into the LC-MS/MS system to analyze the test compound and control compounds. Within each well recording, the percent of control values was calculated for each test compound concentration current response based on peak current in the presence of reference control (current response/ peak current) ×100%. The dose–response curves were fit to the standard Hill equation as shown below:

Ipost cpd/Ipre cpd = Bottom + (Top − Bottom)/(1 + 10^((LogIC50 − X) × HillSlope)^) (1)

where X is the logarithm of concentration, Ipost cpd/Ipre cpd is the normalized peak current amplitude, Top is 1, and Bottom is equal to 0. Curve-fitting and IC50 calculations were performed by GraphPad Prism 5.0. If the inhibition obtained at the lowest concentration tested was over 50%, or at the highest concentration tested was less than 50%, we reported the IC50 as less than the lowest concentration, or higher than the highest concentration, respectively.

#### 3.2.16. In Vitro hERG Assay

CHO cells stably expressing hERG potassium channels from Sophion Biosciences (Ballerup, Denmark) were used for this test. The cells were cultured in a humidified and air-controlled (5% CO_2_) incubator at 37 °C. The CHO cells, which were at least two days after plating and more than 75% confluent, would be used for experiments. Before testing, cells were harvested using TrypLE and resuspended in the physiological solution at room temperature. The physiological solution and external solution were prepared at least one month ago. The intracellular solution was prepared in batches, aliquoted, and stored at 4 °C until used. Test compounds were dissolved in 100% DMSO to obtain stock solutions for different test concentrations. Then the stock solutions were further diluted into the external solution to achieve final concentrations for testing. A visual check for precipitation was conducted before testing. Final DMSO concentration in the extracellular solution was not more than 0.30% for the test compounds. Voltage command protocol: From this holding potential of −80 mV, the voltage was first stepped to −50 mV for 80 ms for leak subtraction, and then stepped to +20 mV for 4800 ms to open hERG channels. After that, the voltage was stepped back down to −50 mV for 5000 ms, causing a “rebound” or tail current, which was measured and collected for data analysis. Finally, the voltage was stepped back to the holding potential (−80 mV, 1000 ms). This voltage command protocol was repeated every 20,000 ms. This command protocol was performed continuously during the test (vehicle control and test compound). The hERG SyncroPatch assay was conducted at room temperature. The Setup, Prime Chip, Catch and Seal Cells, Amplifier Settings, Voltage, and Application Protocols were established with Biomek Software (V5.0; Nanion, Munich, Germany). One addition of 40 μL of the vehicle was applied, followed by 300 s for a baseline period. Then the doses of the compounds were added with 40 μL. The exposure of the test compound at each concentration was no less than 300s. The recording for the whole process had to pass the quality control, or the well was abandoned and the compound was retested, all automatically set by PatchControl. Five concentrations (0.30 μM, 1.00 μM, 3.00 μM, 10.00 μM, and 30.00 μM) were tested for each compound. Data analysis was carried out using DataControl, Excel 2013 (Microsoft, Redmond, WA, USA), and GraphPad Prism 5.0.

## 4. Conclusions

Fibroblast growth factor receptors are frequently dysregulated across cancers and remain clinically important therapeutic targets. In this study, we successfully designed and synthesized a novel series of nucleoside-based irreversible pan-FGFR inhibitors. Through comprehensive SAR investigations, guided by X-ray co-crystal structure (PDB: 9WFK) and MD simulations, we identified the 7-methoxy-5-methylbenzo[*b*]thiophene moiety and ribose hydroxyl groups as critical determinants for potent inhibition across FGFR1–4. Among the structural determinants elucidated, the ribose hydroxyl groups emerged as a critical driving element for potency by forming stable water-mediated hydrogen-bond networks that reinforce ligand anchoring within the FGFR active site. Kinome-wide profiling of compound **13f** validated high FGFR selectivity with minimal off-target effects. Covalent bond with FGFR1 was directly confirmed by LC/MS analysis and Western blot, with compound **22f** having an ethenesulfonamide warhead, exhibiting effective and sustained covalent engagement with FGFR1. Additionally, microsomal stability studies revealed that the electron-withdrawing sp^3^-hybridized sulfonamide contributed to metabolic stability by reducing CYP-mediated degradation. Although futibatinib exhibits superior microsomal stability, the nucleoside scaffold still offers clear potential for further metabolic optimization as a differentiated scaffold. Importantly, representative compounds, including **13f**, **19e**, and **22f**, exhibited strong inhibition across FGFR1–4 and robust antiproliferative efficacy in both FGFR-driven and wild-type cancer models. Notably, **22f** showed dose-dependent inhibition of the AKT and ERK signaling pathways in FGFR1-amplified cells, supporting its on-target mechanism of action. Collectively, these results support nucleoside analogues as a privileged scaffold for covalent pan-FGFR inhibition.

## Figures and Tables

**Table 1:** Structures and biochemical potencies of compounds **5a**–**d**, **6a**–**h**. 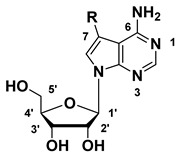

Comp.	R	Inhibition Rate (%) at 1 μM *^a^*/IC_50_ (nM)			
		FGFR1	FGFR2	FGFR3	FGFR4
**5a**	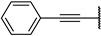	82 ± 4%	61 ± 4%	50 ± 1%	−4 ± 2%
**5b**	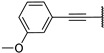	98 ± 2%	97 ± 1%	95 ± 4%	25 ± 3%
**5c**	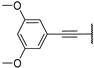	99 ± 2%	96 ± 0%	98 ± 0%	25 ± 9%
**5d**	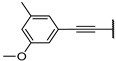	99 ± 2%	99 ± 1%	98 ± 1%	22 ± 1%
**6a**	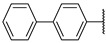	37 ± 0%	17 ± 8%	20 ± 1%	−11 ± 4%
**6b**	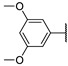	102 ± 0%	100 ± 1%	101 ± 1%	88 ± 2%
**6c**	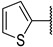	99 ± 0%	90 ± 1%	92 ± 3%	28 ± 5%
**6d**	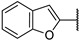	80 ± 2%	29 ± 2%	26 ± 4%	5 ± 6%
**6e**	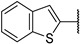	98 ± 1%	100 ± 0%	98 ± 1%	83 ± 1%
**6f**	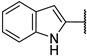	82 ± 5%	82 ± 5%	57 ± 5%	15 ± 5%
**6g**	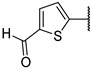	75 ± 3%	75 ± 3%	63 ± 3%	4 ± 2%
**6h**	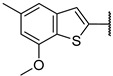	101 ± 1% 2	102 ± 1% 4	100 ± 0% 6	73 ± 2% 299

*^a^* Data are shown as mean ± SD (n = 2).

**Table 2:** Structures, biochemical potencies and antiproliferation efficacy of compounds **13a**–**f**, ent-**13f**, **19a**–**e** *^a^*. 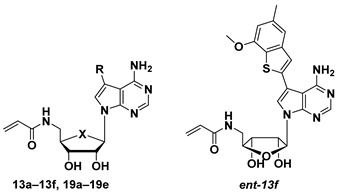

Comp.	X	R	Inhibition Rate (%) at 0.1 μM *^b^*/IC_50_ (nM) *^c^*				IC_50_ (μM) *^d^*	
			FGFR1	FGFR2	FGFR3	FGFR4	HCT116 (FGFR1 Amplification)	RT4 (FGFR3-TACC3
**13a**	O	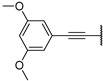	49 ± 2% 81	53 ± 2%	64 ± 2% 57	−5 ± 4%	>1000	19.05
**13b**	O	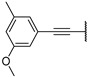	84 ± 0% 25	67 ± 5%	66 ± 2% 57	−7 ± 5%	7.33	21.69
**13c**	O	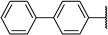	2 ± 10%	−2 ± 9%	−2 ± 1% >1000	−1 ± 1%	17.39	8.37
**13d**	O	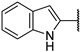	30 ± 6%	9 ± 2%	10 ± 2% >1000	−7 ± 3%	8.81	32.87
**13e**	O	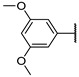	20 ± 6%	27 ± 2%	17 ± 3%	0 ± 6%	>1000	N.D.
**13f**	O	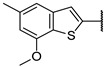	100 ± 0% 2	97 ± 0% 4	97 ± 1% 3	45 ± 5% 102	1.89	10.59
**ent-13f**	O	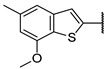	88 ± 0% 19	71 ± 3% 51	84 ± 0% 17	8 ± 5% >1000	3.51	N.D.
**19a**	S	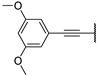	88 ± 1% 20	75 ± 3%	−5 ± 1%	−5 ± 3%	12.03	16.42
**19b**	S	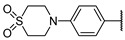	−14 ± 6% >1000	5 ± 5%	8 ± 8% >1000	1 ± 2%	>1000	>50
**19c**	S	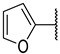	16 ± 2% 285	14 ± 1%	−7 ± 5% >1000	13 ± 4%	>1000	>50
**19d**	S	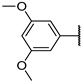	59 ± 4% 76	50 ± 1%	24 ± 5% 284	7 ± 4%	19.88	>50
**19e**	S	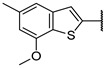	100 ± 1% 0.9	98 ± 1% 4	100 ± 0% 3	64 ± 3% 61	0.52	1.52
Futibatinib			6	61	12	>1000	15.05	3.90
Fexagratinib			0.7	4	2	82	4.10	15.05

*^a^* N.D.: Not Determined. *^b^* Data are shown as mean ± SD (n = 2). *^c^* All IC_50_ values are shown as mean ± SEM (n = 2), and were obtained using the same biochemical assay platform. Values of reference compounds are provided for comparison but may differ due to assay condition. *^d^* IC_50_ values against cancer cell lines were calculated from three independent experiments.

**Table 3:** In vitro half-life (T_1/2_) and microsomal intrinsic clearance (CL_int_) of compounds **6h**, **13f**, and **19e** and control drugs across human, rat, and mouse liver microsomes *^a^*.

Comp.	Human		Rat		Mouse	
	T_1/2_ (min)	CL_int_ (mic) (μL/min/mg)	T_1/2_ (min)	CL_int_ (mic) (μL/min/mg)	T_1/2_ (min)	CL_int_ (mic) (μL/min/mg)
**6h**	67.7	20.5	64.9	21.4	69.2	20.0
**13f**	9.4	148.1	26.3	52.6	19.1	72.6
**19e**	2.3	606.1	8.3	167.6	5.1	273.3
Testosterone	17.4 ± 2.3	80.4 ± 11.5	1.6 ± 0.2	827.2 ± 93.2	6.3 ± 1.5	233.4 ± 31.2
Diclofenac	5.6 ± 0.6	258.3 ± 27.9	16.8 ± 3.7	98.7 ± 23.5	41.3 ± 5.6	32.4 ± 4.2
Propafenone	7.0 ± 1.5	202.6 ± 38.9	1.5 ± 0.2	946.6 ± 97.5	2.9 ± 0.7	550.3 ± 163.2

*^a^* The control values are mean ± SD across batches.

**Table 4:** Structures, biochemical potencies, and antiproliferation efficacy of compounds **21** and **22a**–**f**. 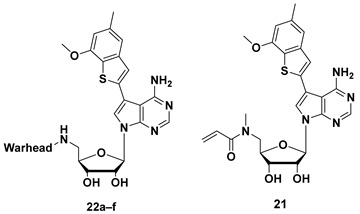

Comp.	Warhead	Inhibition Rate (%) at 0.1 μM ^*a*^/IC_50_ ± SEM (nM) *^b^*				IC_50_ (μM) *^c^*	
		FGFR1	FGFR2	FGFR3	FGFR4	HCT116 (FGFR1 Amplification)	RT4 (FGFR3-TACC3
**13f**	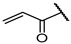	100 ± 0% 2	97 ± 0% 4	97 ± 1% 3	45 ± 5% 102	1.89	10.59
**21**		98 ± 0% 3	89 ± 1% 16	95 ± 0% 8	40 ± 2% 151	5.03	6.30
**22a**	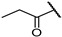	98 ± 1% 3	91 ± 1% 8	94 ± 3% 8	43 ± 0% 137	2.08	10.87
**22b**	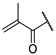	99 ± 1%	91 ± 2%	101%	92 ± 4%	2.73	1.22
**22c**	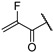	99 ± 0%	94 ± 0%	97 ± 2%	77 ± 1%	1.68	1.43
**22d**	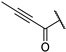	99 ± 1%	95 ± 2%	96 ± 1%	35 ± 2%	1.63	0.07
**22e**	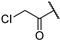	100 ± 0%	97 ± 1%	97 ± 0%	78 ± 1%	0.65	10.33
**22f**	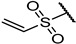	98 ± 1% 1	95 ± 0% 8	97 ± 0% 5	64 ± 5% 53	0.58	0.67
Futibatinib		6	61	12	>1000	15.05	3.90
Fexagratinib		0.7	4	2	82	4.10	15.05

*^a^* Data are shown as mean ± SD (n = 2). *^b^* All IC_50_ values are shown as mean ± SEM (n = 2), and were obtained using the same biochemical assay platform. Values of reference compounds are provided for comparison but may differ due to assay condition. *^c^* IC_50_ values against cancer cell lines were calculated from three independent experiments.

**Table 5:** In vitro half-life (T_1/2_) and microsomal intrinsic clearance (CL_int_) of compounds **21**, **22a**, **22f**, and futibatinib and control drugs across human, rat, and mouse liver microsomes *^a^*.

Comp.	Human		Rat		Mouse	
	T_1/2_ (min)	CL_int_ (mic) (μL/min/mg)	T_1/2_ (min)	CL_int_ (mic) (μL/min/mg)	T_1/2_ (min)	CL_int_ (mic) (μL/min/mg)
**21**	12.8	108.5	11.8	117.1	12.1	114.4
**22a**	12.6	109.9	23.8	58.2	24.7	56.0
**22f**	29.7	46.7	18.2	76.0	27.7	50.0
**Futibatinib**	57.3	24.2	40.8	34.0	60.5	22.9
Testosterone	17.4 ± 2.3	80.4 ± 11.5	1.6 ± 0.2	827.2 ± 93.2	6.3 ± 1.5	233.4 ± 31.2
Diclofenac	5.6 ± 0.6	258.3 ± 27.9	16.8 ± 3.7	98.7 ± 23.5	41.3 ± 5.6	32.4 ± 4.2
Propafenone	7.0 ± 1.5	202.6 ± 38.9	1.5 ± 0.2	946.6 ± 97.5	2.9 ± 0.7	550.3 ± 163.2

*^a^* The control values are mean ± SD across batches.

**Table 6:** Anticancer Activity of the **13a**–**f**, **19b**–**e**, **21**, and **22a**–**f** against 5 representative human cancer cell lines.

Comp.			IC_50_ (μM) *^a^*		
	SK-HEP-1	MDA-MB-231	A549	HCT116	SNU-638
**13a**	9.60	>50	48.83	>50	29.13
**13b**	3.58	7.05	12.69	37.88	13.29
**13f**	0.26	0.61	0.69	1.12	0.82
**19b**	>50	>50	>50	>50	>50
**19c**	26.10	>50	>50	33.50	>50
**19d**	>50	>50	>50	>50	>50
**19e**	0.55	0.62	0.82	0.93	0.75
**21**	0.60	0.81	1.21	1.96	1.42
**22a**	1.24	2.36	3.77	4.88	5.00
**22b**	0.98	3.33	2.68	4.63	2.85
**22c**	0.50	1.25	1.15	1.68	1.49
**22d**	0.51	0.78	1.61	1.43	0.85
**22e**	0.36	0.91	0.88	0.92	0.75
**22f**	0.57	0.65	0.64	0.92	0.41
Etoposide	0.69	7.75	0.17	0.96	0.21

*^a^* Measured using SRB assay. IC_50_ values against cancer cell lines were calculated from three independent experiments.

**Table 7:** Summary of ADMET profile of compound **22f**.

Parameters	Results
CYP Inhibition *^a^*: IC_50_ (μM)	>50/>50/20.7/10.8/>50
Metabolic Stability in Plasma *^b^*: T_1/2_ (min)	47.3/32.6/37.3
hERG inhibition: IC_50_ (μM)	>30
Human oral absorption *^c^* (%)	41.1
Serum albumin binding *^c^* (logK_HSA_)	−0.177

*^a^* CYP inhibition IC_50_ values were determined in human liver microsomes for CYP3A4/5, 2D6, 2C19, 2C9, and 1A2, respectively. *^b^* Plasma stability is shown as half-life (T_1/2_, min) in mouse, rat, and human plasma, respectively. *^c^* predicted parameters using QikProp (Schrödinger 14.2).

## Data Availability

The original contributions presented in this study are included in the article and supplementary material. Further inquiries can be directed to the corresponding author.

## Supplementary Materials

The following supporting information can be downloaded at: https://www.mdpi.com/article/10.3390/ph18111745/s1, File S1. 468 kinase selectivity of **13f**. Figure S1. ORTEP diagram of compound **6h** showing thermal ellipsoids at 50% probability. Table S1. Crystal Data and Structure Refinement for **6h**. Figure S2. ORTEP diagram of compound ent-**7** showing thermal ellipsoids at 50% probability. Table S2. Crystal Data and Structure Refinement for ent-**7**. Table S3. Data collection and refinement (PDB ID: 9WFK). Table S4. Dissociation constant of **19d** and **19e** at FGFR1–4. Figure S3. Binding curves obtained from kinase binding assays. Figure S4. Replicated Western blot experiments confirming the inhibitory effect of **22f** (LJ5442) on FGFR1 signaling pathway. Figure S5. Molecular dynamics (MD) simulation analysis of the **22f** at FGFR4 (PDB ID: 4R6V) over a 100 ns trajectory performed three times. Table S5. hERG inhibition profile of **22f**. Figures S6–S55. ^1^H, ^13^C NMR of the final compounds, HPLC data of the representative compounds, and IC_50_ curves of compounds. Figures S56–S86. MS spectra of compounds. References [37,38,39] are cited in supplementary materials.

## Abbreviations

The following abbreviations are used in this manuscript:

FGFR	Fibroblast growth factor receptor
FGF	Fibroblast Growth Factor
TKI	Tyrosine Kinase Inhibitor
SAR	Structure–Activity Relationship
IC_50_	Half maximal inhibitory concentration
Kd	Dissociation constant
MD	Molecular Dynamics
ERK	Extracellular Signal-Regulated Kinase
MEK	Mitogen-Activated Protein Kinase Kinase
MAPK	Mitogen-Activated Protein Kinase
PI3K	Phosphoinositide 3-kinase
AKT	Protein Kinase B
TACC3	Transforming Acidic Coiled-Coil containing protein 3
TNBC	Triple-Negative Breast Cancer
NSCLC	Non-Small Cell Lung Cancer
HCT116	Human colorectal carcinoma cell line
RT4	Human bladder cancer cell line
SRB	Sulforhodamine B
ADME	Absorption, Distribution, Metabolism, and Excretion
CYP	Cytochrome P450
hERG	Human Ether-à-go-go-Related Gene (potassium channel)
MS	Mass Spectrometry
HRMS	High-Performance Liquid Chromatography
UV	Ultraviolet
ESI	Electrospray Ionization
FAB	Fast Atom Bombardment
CL_int_	Intrinsic Clearance
SEM	Standard Error of the Mean
SD	Standard Deviation
EDC	1-Ethyl-3-(3-dimethylaminopropyl)carbodiimmide
DMAP	4-Dimethylaminopyridine
DIAD	Diisopropyl azodicarboxylate
DBU	1,8-Diazabicyclo[5.4.0]undec-7-ene
PEt_3_	Triethylphosphine
TBSCl	tert-Butyldimethylsilyl chloride
TBAF	Tetra-n-butylammonium fluoride
TBDPS	tert-Butyldiphenylsilyl
DPPA	Diphenylphosphoryl azide
TCDI	1,1′-Thiocarbonyldiimidazole
DMSO	Dimethyl sulfoxide
ACN	Acetonitrile
EtOAc	Ethyl acetate
THF	Tetrahydrofuran
DMF	Dimethylformamide
MeOH	Methanol
