# Supplementary material for: Design, Synthesis, and Biological Evaluation of 5′,7-Disubstituted 7-Deaza-adenosine Analogues as Irreversible Pan-FGFR Inhibitors
Source: Pharmaceuticals (Basel). 2025 Nov 17;18(11):1745. doi: 10.3390/ph18111745 (PMC12655421; doi:10.3390/ph18111745)
Supplement: Supplementary file 1 [file pharmaceuticals-18-01745-s001.zip › pharmaceuticals-3979700-supplementary/Supporting Informations_Pharmaceuticals_ver.1.pdf]

Supporting Information

Design, Synthesis, and Biological Evaluation of 5′,7-Disubstituted 7-Deaza-adenosine Analogues as Irreversible Pan-FGFR Inhibitors

Jung Hoon Park, Phuong Thao Tran, Hye Lin Ko, Seonghee Mun, Sung Chul Jang, Dong Hyun Moon, Jaeho Han, Jieun Kim, Gibae Kim, Hongseok Choi, Seung Woo Kim, Minjae Kim, Sang Kook Lee, Byung Woo Han, Keon Wook Kang, and Lak Shin Jeong*

***Corresponding authors: lakjeong@snu.ac.kr**

Table of Contents

1. Single-Crystal X-ray crystallography data of compounds 6h and *ent*-7 ……………………………….S2

2. Data collection and refinement (PDB ID : 9WFK) ……………………………………………………S6

3. Dissociation constant of 19d and 19e at FGFR1–4 ………………………………………………….S7

4. Replicated western blot experiments ………………………………………………………………S8

5. Supplementary images of Molecular Dynamic Simulation…………………………………………S9

6. hERG inhibition profile of 22f ……………………………………………………………………S10

7. ^{1}H and ^{13}C NMR of the final compounds……………………………………………………………S11

8. HPLC data of the representative compounds…………………………………………………………S43

9. IC_{50} curves of compounds……………………………………………………………………………S44

10. MS spectra of componds……………………………………………………………………………S52

Single-Crystal X-ray crystallography of compound 6h

Compound **6h** (10mg) was dissolved in dichloromethane (1.0 mL) in a glass vial. After staying at room temperature, the single crystals of **6h** were obtained. X-ray diffraction data of **6h** (CCDC No: 2472736) were obtained on a SuperNova, Dual, Cu at zero, AtlasS2 diffractometer. Using Olex2 [1], the structure was solved with the ShelXT [2] structure solution program using Direct Methods and refined with the ShelXL [3] refinement package using Least Squares minimization.

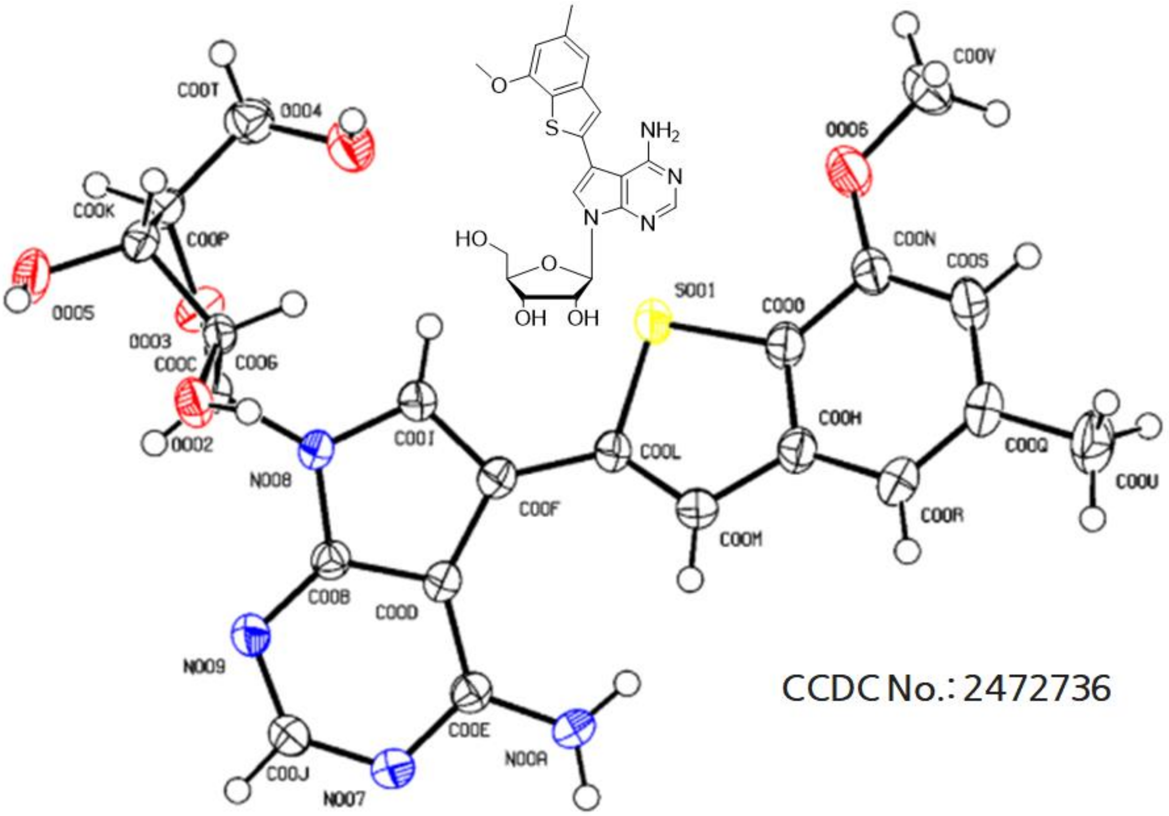

Figure S1. ORTEP diagram of compound **6h** showing thermal ellipsoids at 50% probability.

Table S1. Crystal Data and Structure Refinement for **6h.**

Empirical formula	$C_{21}H_{22}N_4O_5S$
Formula weight	442.48
Temperature/K	294.1(6)
Crystal system	orthorhombic
Space group	$P2_12_12_1$
a/Å	5.00521(17)
b/Å	15.7834(4)
c/Å	25.7378(6)
α/°	90
β/°	90
γ/°	90
Volume/Å^3	2033.27(10)
Z	4
ρ_{calc}g/cm^3	1.445
μ/mm^{-1}	1.787
F(000)	928.0
Crystal size/mm^3	0.2 × 0.15 × 0.1
Radiation	CuKα (λ = 1.54184)
2Θ range for data collection/°	6.57 to 147.43
Index ranges	$-3 \leq h \leq 5$, $-13 \leq k \leq 19$, $-31 \leq l \leq 26$
Reflections collected	4791
Independent reflections	3348 [R_{int} = 0.0203, R_{sigma} = 0.0320]
Data/restraints/parameters	3348/0/285
Goodness-of-fit on F^2	1.084
Final R indexes [I>=2σ (I)]	R_1 = 0.0314, wR_2 = 0.0940
Final R indexes [all data]	R_1 = 0.0358, wR_2 = 0.0988
Largest diff. peak/hole / e Å^{-3}	0.17/-0.17
Flack parameter	-0.02(2)

Single-Crystal X-ray crystallography of compound *ent*-7

Compound ***ent*-7** (10mg) was dissolved in dichloromethane (1.0 mL) in a glass vial. After staying at room temperature, the single crystals of ***ent*-7** were obtained. X-ray diffraction data of ***ent*-7** (CCDC No: 2474352) were obtained on a SuperNova, Dual, Cu at zero, AtlasS2 diffractometer. Using Olex2 [1], the structure was solved with the ShelXT [2] structure solution program using Direct Methods and refined with the ShelXL [3] refinement package using Least Squares minimization.

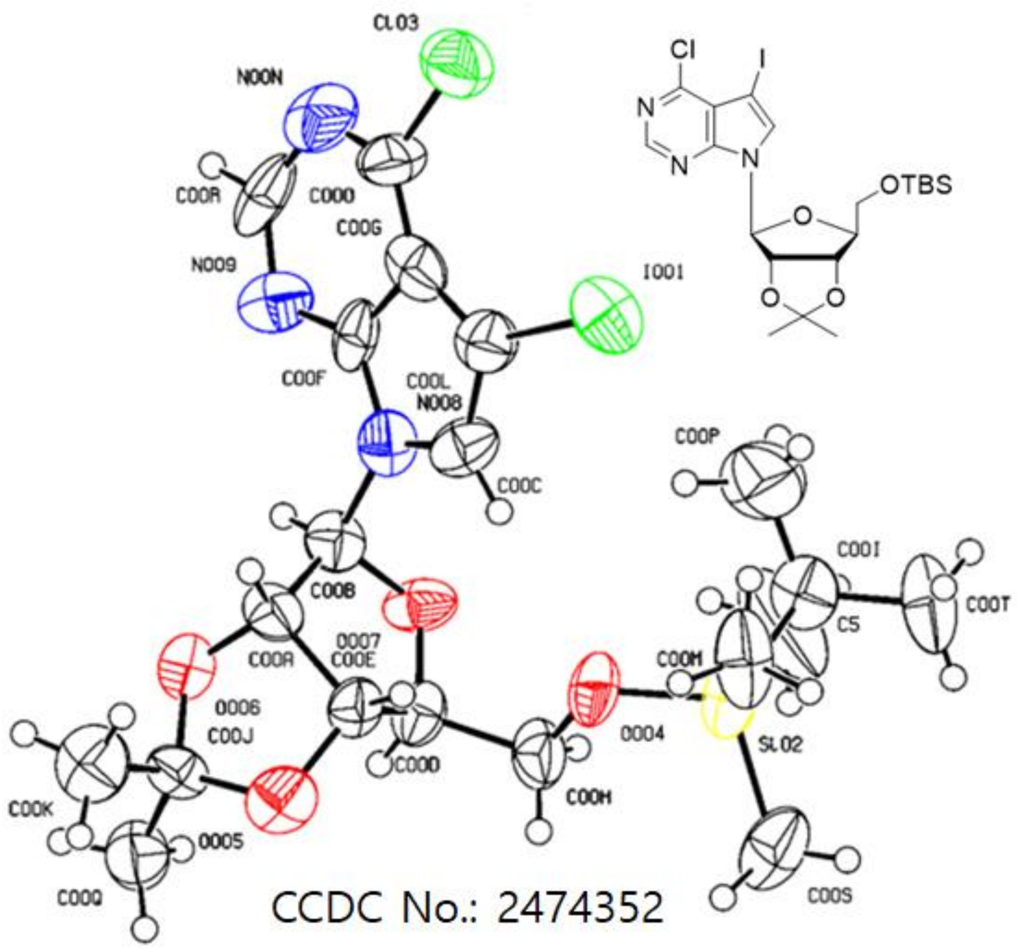

Figure S2. ORTEP diagram of compound ***ent*-7** showing thermal ellipsoids at 50% probability.

Table S2. Crystal Data and Structure Refinement for ***ent*-7.**

Empirical formula	$C_{20}H_{29}ClIN_3O_4Si$
Formula weight	565.90
Temperature/K	291.88(10)
Crystal system	orthorhombic
Space group	$P2_12_12_1$
a/Å	7.4458(6)
b/Å	16.2403(19)
c/Å	20.229(2)
α/°	90
β/°	90
γ/°	90
Volume/Å^3	2446.1(4)
Z	4
ρ_{calc}g/cm^3	1.537
μ/mm^{-1}	1.497
F(000)	1144.0
Crystal size/mm^3	0.2 × 0.15 × 0.1
Radiation	MoKα (λ = 0.71073)
2Θ range for data collection/°	4.026 to 59.31
Index ranges	$-8 \le h \le 9$, $-14 \le k \le 20$, $-23 \le l \le 27$
Reflections collected	8880
Independent reflections	5452 [R_{int} = 0.1230, R_{sigma} = 0.1697]
Data/restraints/parameters	5452/0/274
Goodness-of-fit on F^2	1.159
Final R indexes [I>=2σ (I)]	R_1 = 0.1482, wR_2 = 0.3227
Final R indexes [all data]	R_1 = 0.1914, wR_2 = 0.3884
Largest diff. peak/hole / e Å^{-3}	2.23/-2.67
Flack parameter	-0.08(9)

Table S3. Data collection and refinement (PDB ID : 9WFK)

Data collection	9WFK
**Ligand**	**6h**
**Diffraction source**	Beamline 5C, Pohang Accelerator Laboratory
**Wavelength (A)**	1.00003
**Space group**	C2
**a, b, c (Å)**	212.002, 50.823, 65.665
**α, β, γ (°)**	90.000, 107.046, 90.000
**Resolution range (Å)**	48.50 - 1.89
**No. of unique reflections**	230,405 / 52,418
**Completeness (%)**	89.18
**Multiplicity**	4.4 (3.4)
<I/σ(I)>	20.9 (2.8)
**$CC_{1/2}$**	0.992 (0.725)
**R_{merge}**	0.083 (0.684)
**$R_{p.i.m.}$**	0.038 (0.334)

Data Collection and Refinement. X-ray diffraction data for FGFR1 crystals complexed with **6h** is collected at 100 K on the BL-5C beamline at Pohang Light Source. Crystals yielded 52,418 unique reflections (redundancy 4.4, completeness 89.18%, resolution 1.89 Å, R_{merge} 8.3%). Crystal forms belonged to space group C2 with similar unit cell parameters.

Table S4. Dissociation constant of **19d and 19e** at FGFR1–4.

	Kd (nM)			
Comp.	FGFR1	FGFR2	FGFR3	FGFR4
19d	30	22	91	12
19e	0.57	0.64	1.2	1.1

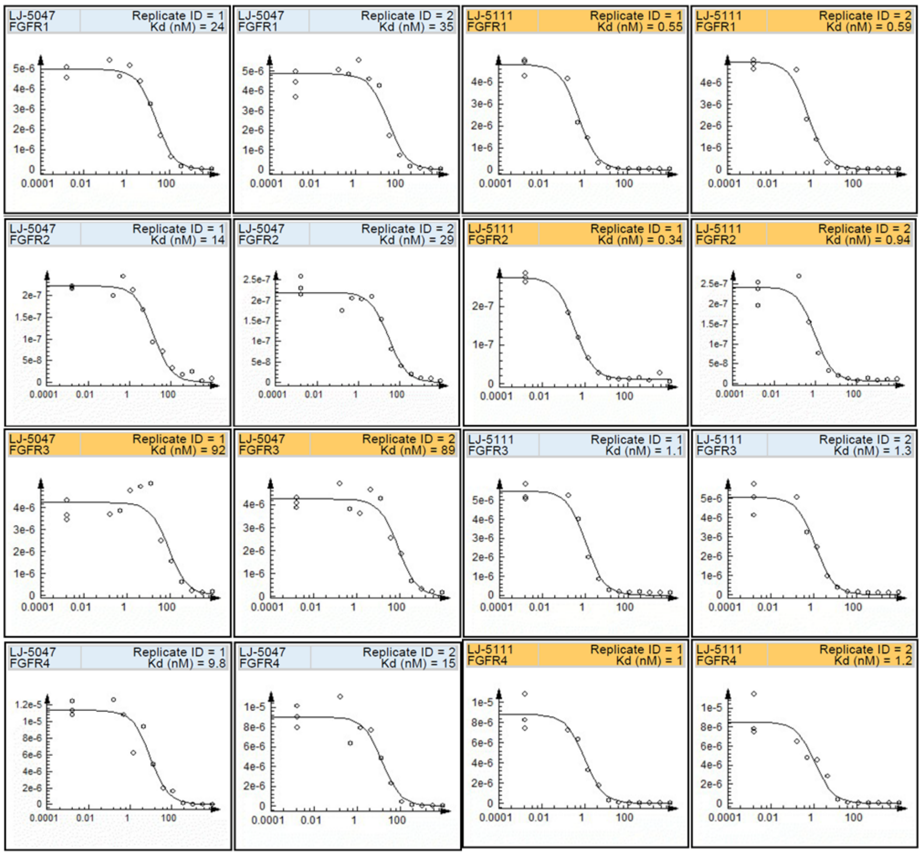

Figure S3. Binding curves obtained from kinase binding assays. Curves were used to determine the dissociation constants (Kd) of compounds **19d** and **19e** against FGFR1–4.

Binding Affinity Measurement Using KdELECT Assay. The dissociation constant (Kd) values of test compounds against selected kinases were determined using the KdELECT assay platform (KINOMEscan™, Eurofins DiscoverX). This competitive binding assay quantifies the ability of compounds to compete with an immobilized, active-site-directed ligand for binding to DNA-tagged kinase constructs. T7 phage-displayed kinases tagged with unique DNA barcodes were expressed in E. coli (BL21) or HEK-293 cells. The kinases were incubated with streptavidin-coated magnetic beads pre-loaded with biotinylated small-molecule ligands. After blocking and washing to minimize non-specific binding, binding reactions were assembled in a final volume of 20 µL in 384-well polypropylene plates. Reactions contained DNA-tagged kinase, ligand-conjugated beads, and test compounds diluted in 1× binding buffer (20% SeaBlock, 0.17× PBS, 0.05% Tween-20, 6 mM DTT) with a final DMSO concentration of 0.9%. Each compound was tested in an 11-point, 3-fold serial dilution series, prepared from a 111× stock solution in 100% DMSO. After 1-hour incubation at room temperature with shaking, the plates were washed with 1× PBS containing 0.05% Tween-20. Bound kinases were eluted using 0.5 µM of non-biotinylated ligand in elution buffer and quantified by quantitative PCR (qPCR) targeting the DNA tag. Kd values were derived by fitting the data to a single-site binding model using the Hill equation with a fixed Hill slope of –1. Curve fitting was performed using the Levenberg–Marquardt algorithm to calculate non-linear least squares regression.

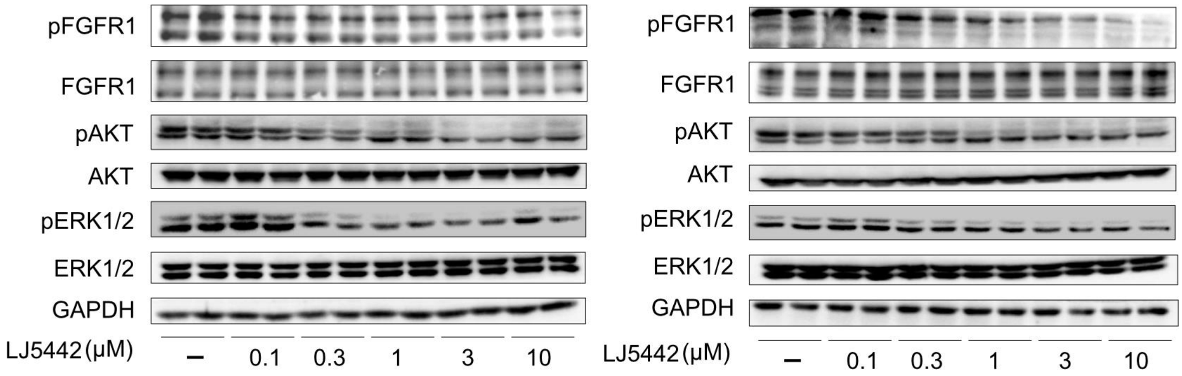

Figure S4. Replicated western blot experiments confirming the inhibitory effect of **22f (LJ5442)** on FGFR1 signaling pathway.

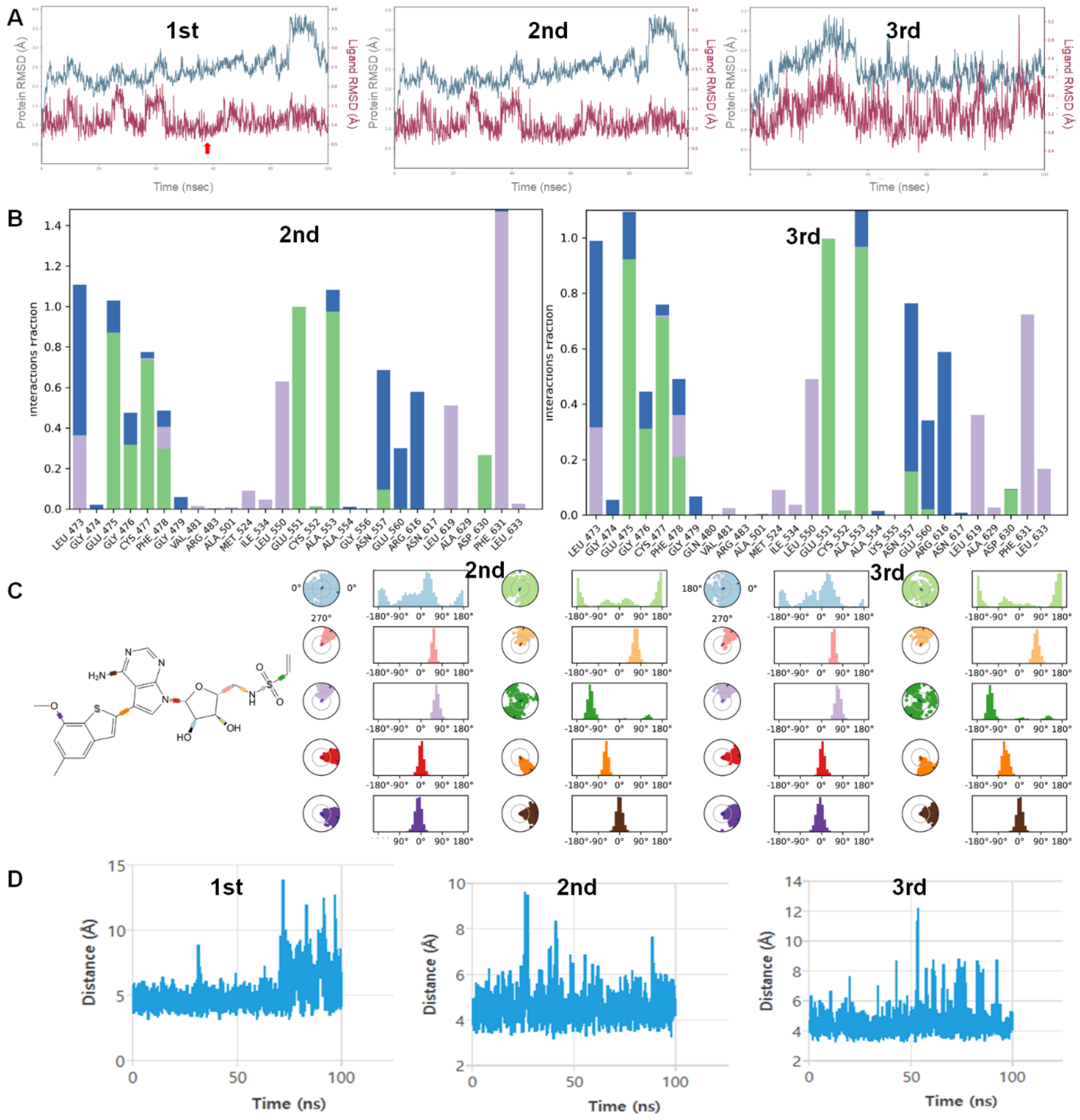

Figure S5. Molecular dynamics (MD) simulation analysis of the **22f** at FGFR4 (PDB ID: 4R6V) over a 100 ns trajectory performed three times. (A) Time-dependent RMSD plots of the protein (blue) and ligand (red) for the all three simulations. (B) Protein–ligand interaction fractions showing key residue contacts observed during 2nd and 3rd simulations. (C) Torsion profiles of the ligand for the 2nd and 3rd simulations, with color-coded rotatable bonds mapped to radial and histogram plots, highlighting the conformational rigidity or flexibility of each torsion angle. (D) Time-dependent distance between the β-carbon of the ethenesulfonamide group and the thiol group of Cys477 monitored during all three simulations.

Table S5. hERG inhibition profile of **22f**

Compound	Conc (uM)	% Inhibition	Std Deviation	DR Curve
**22f**	0.30	6.15	1.30	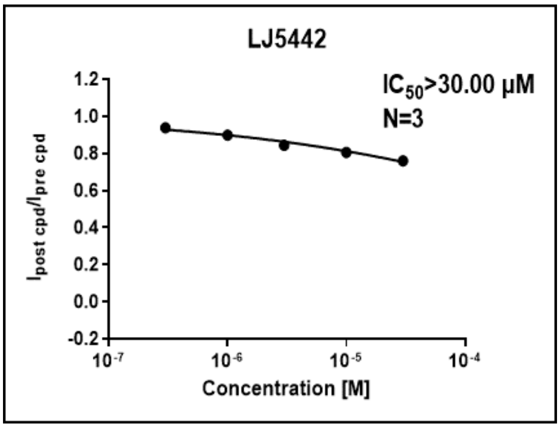
	1.00	10.17	3.21	
	3.00	15.67	2.42	
	10.00	19.46	3.54	
	30.00	24.07	3.03	

References

[1] Dolomanov, O. V.; Bourhis, L. J.; Gildea, R. J; Howard, J. A. K.; Puschmann, H. OLEX2: A Complete Structure Solution, Refinement and Analysis Program. J. Appl. Cryst. 2009, 42, 339-341.

[2] Sheldrick, G. M. SHELXT – Integrated space-group and crystal-structure determination Acta Cryst. 2015, A71, 3-8.

[3] Sheldrick, G. M. A Short History of SHELX. Acta Crystallographica. Acta Cryst. 2008, A64, 112-122

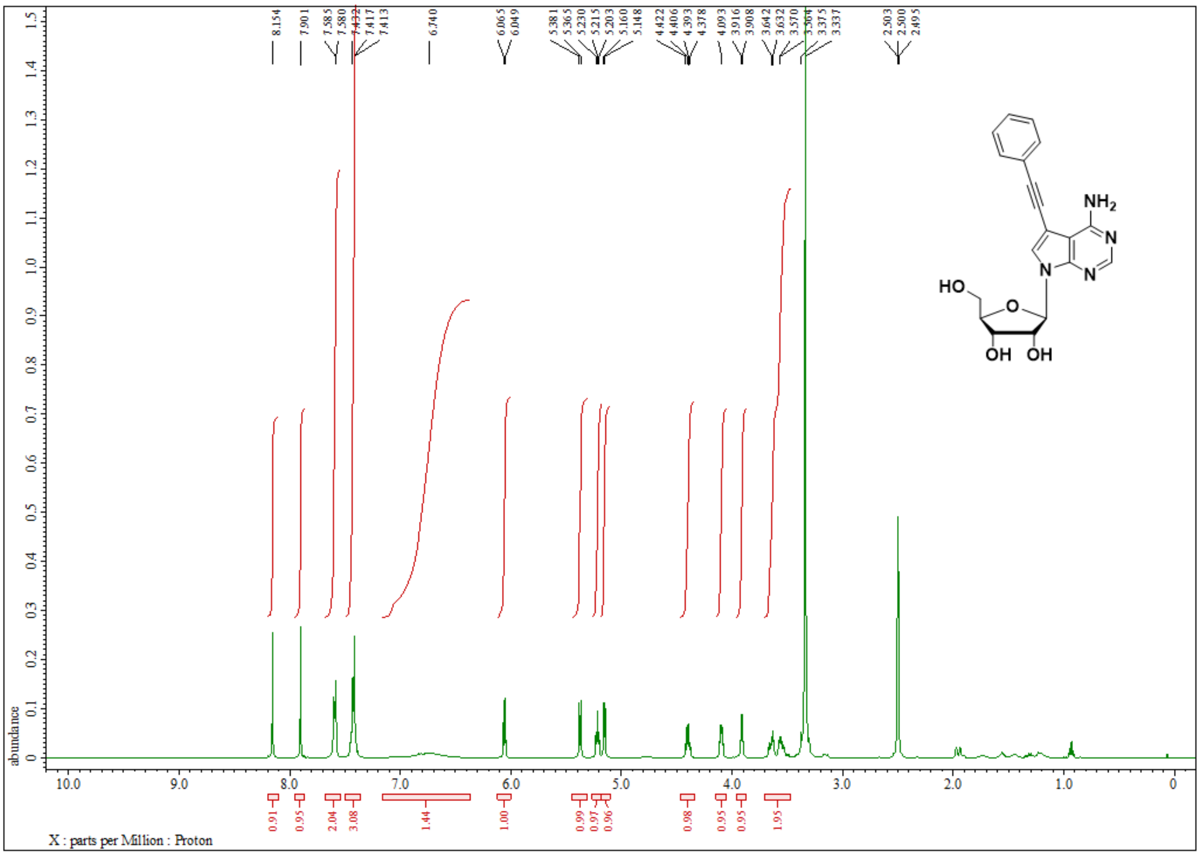

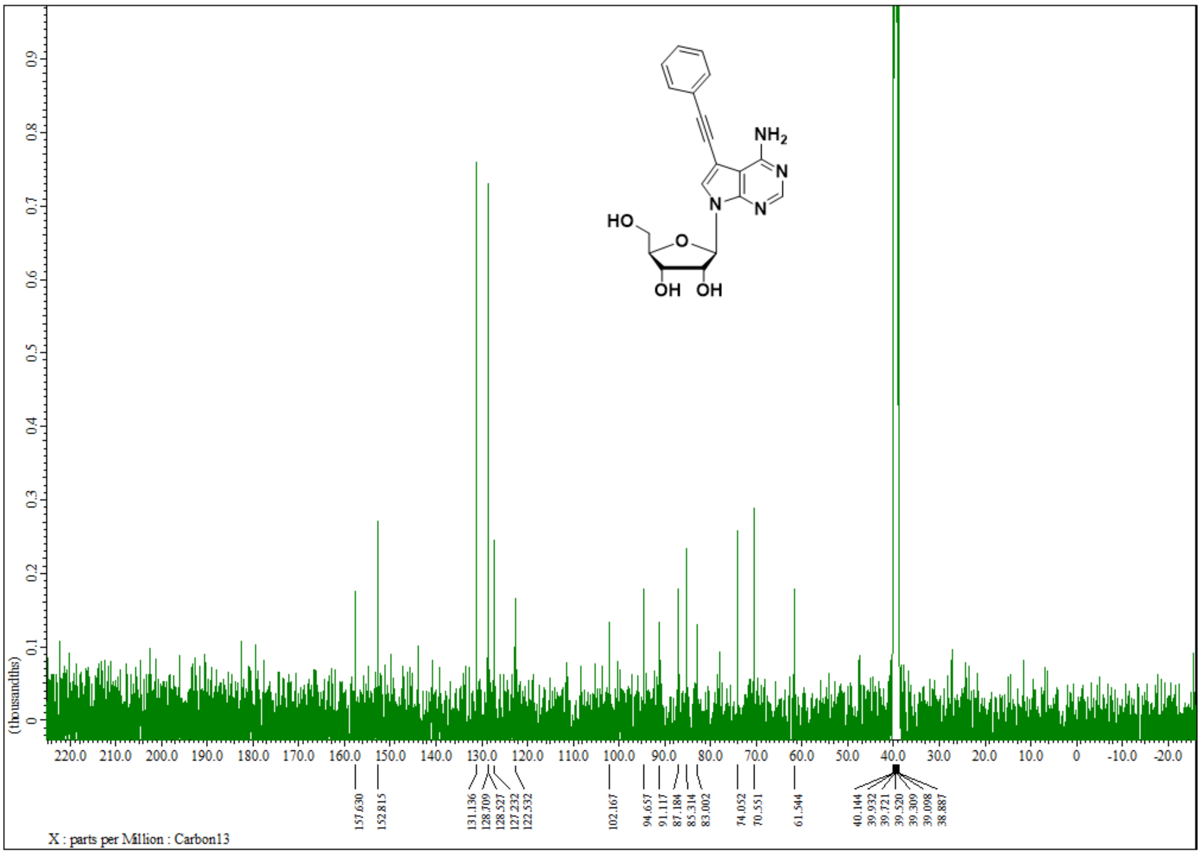

Figure. S6. ^{1}H-NMR (DMSO-d_6, 400 MHz) and ^{13}C-NMR (DMSO-d_6, 100 MHz) spectra of compound **5a**

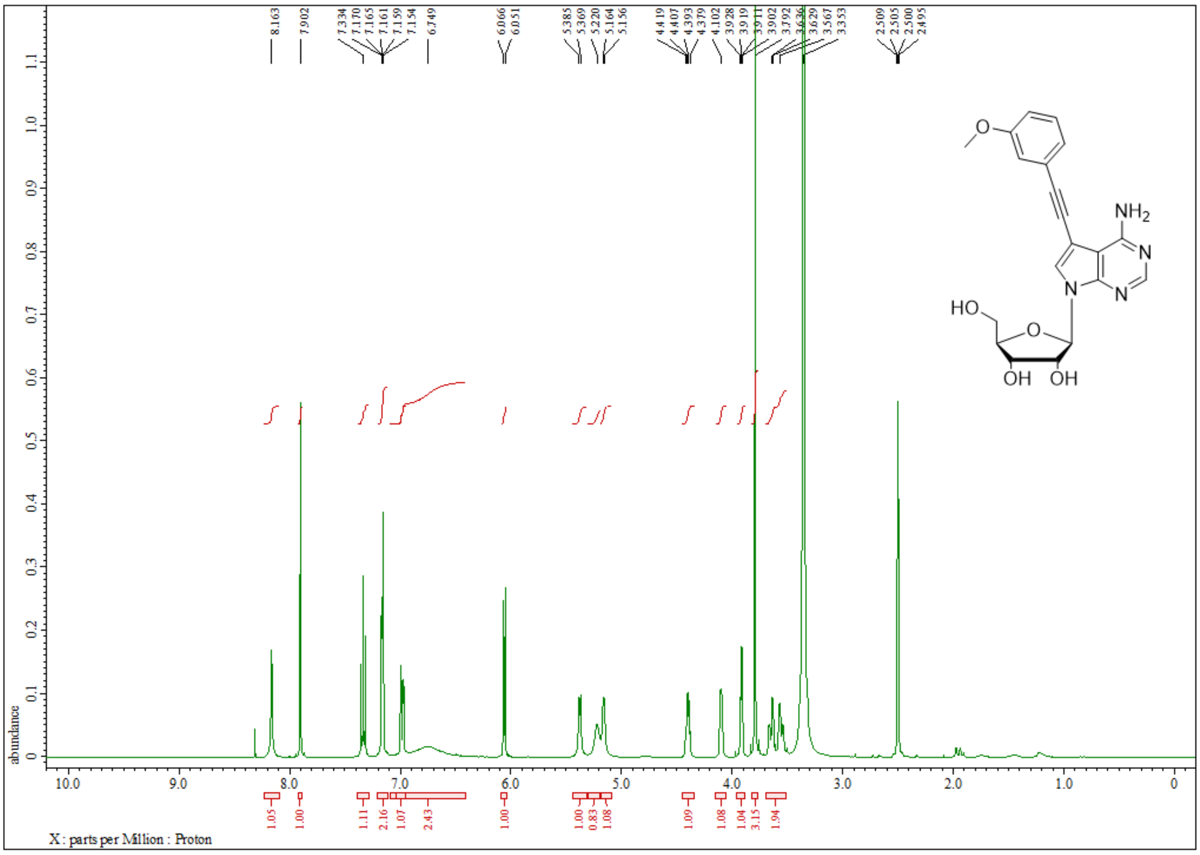

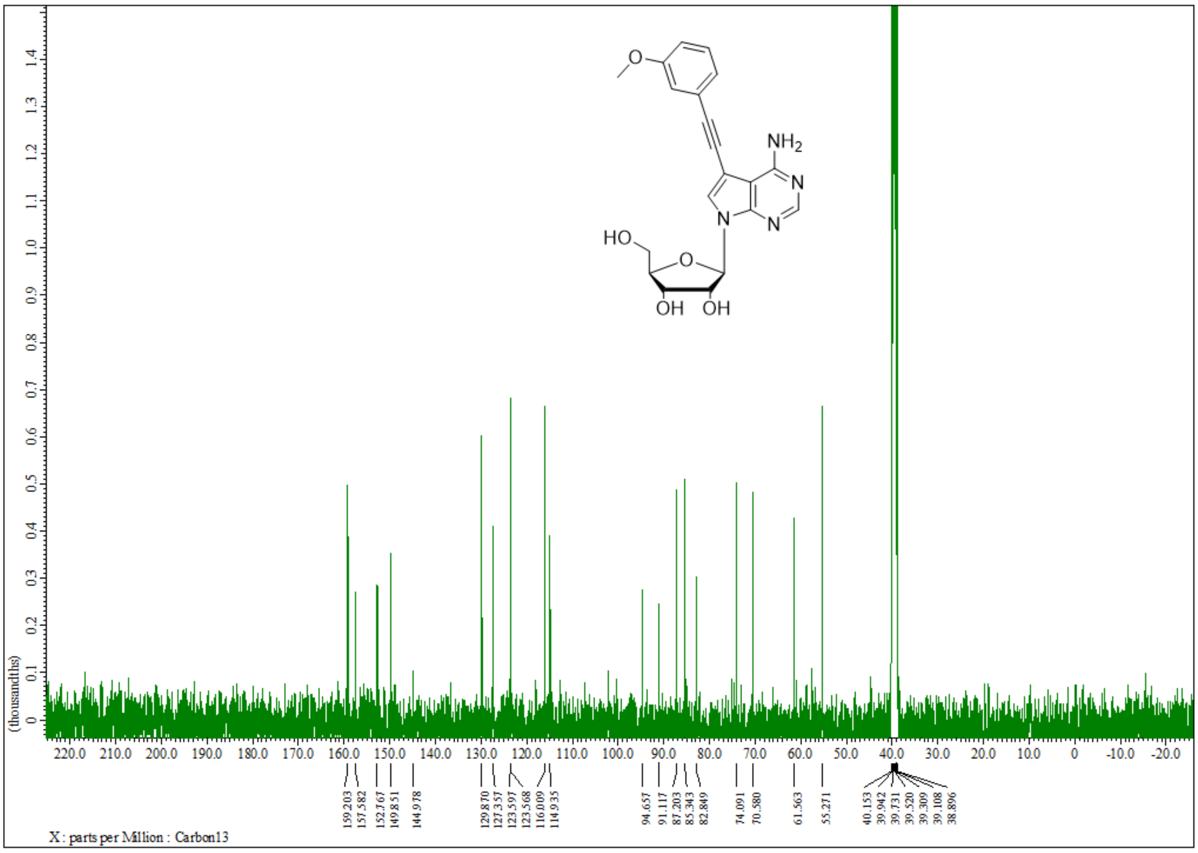

Figure. S7. ^{1}H-NMR (DMSO-d_6, 400 MHz) and ^{13}C-NMR (DMSO-d_6, 100 MHz) spectra of compound **5b**

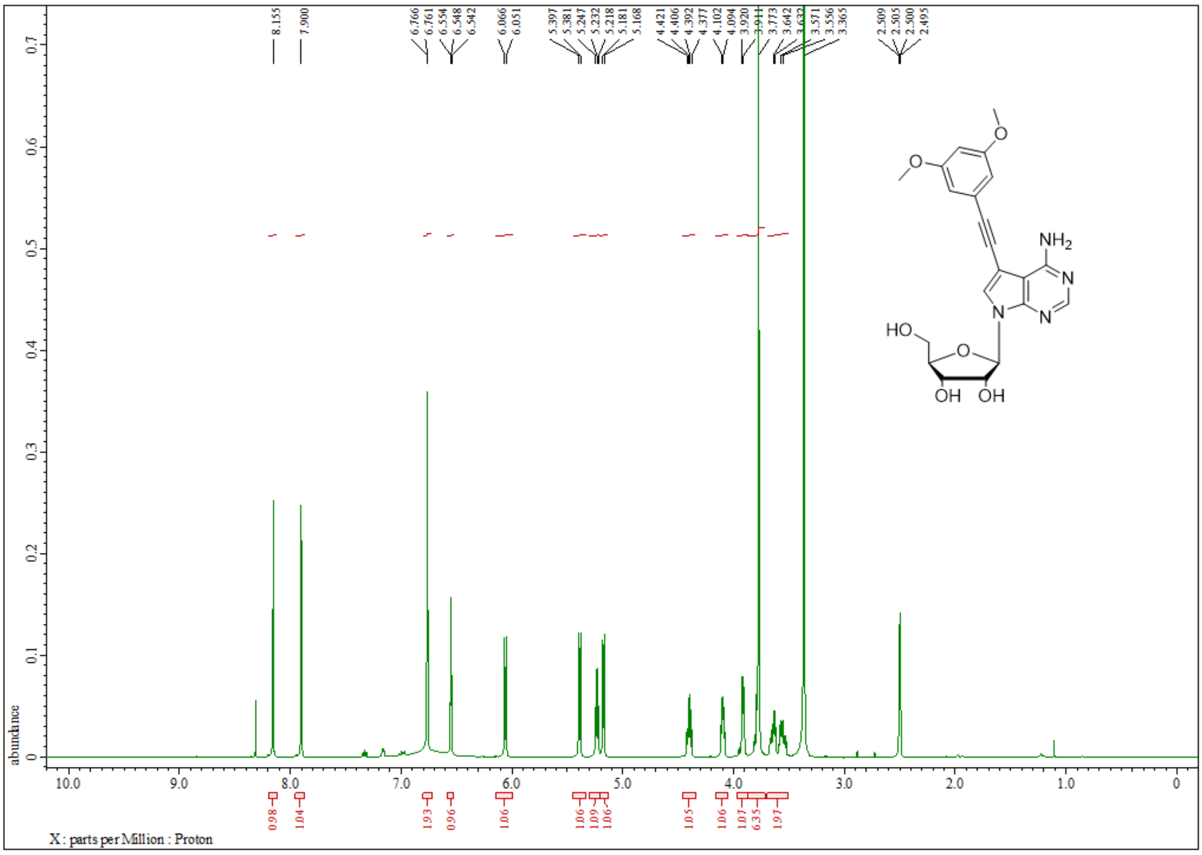

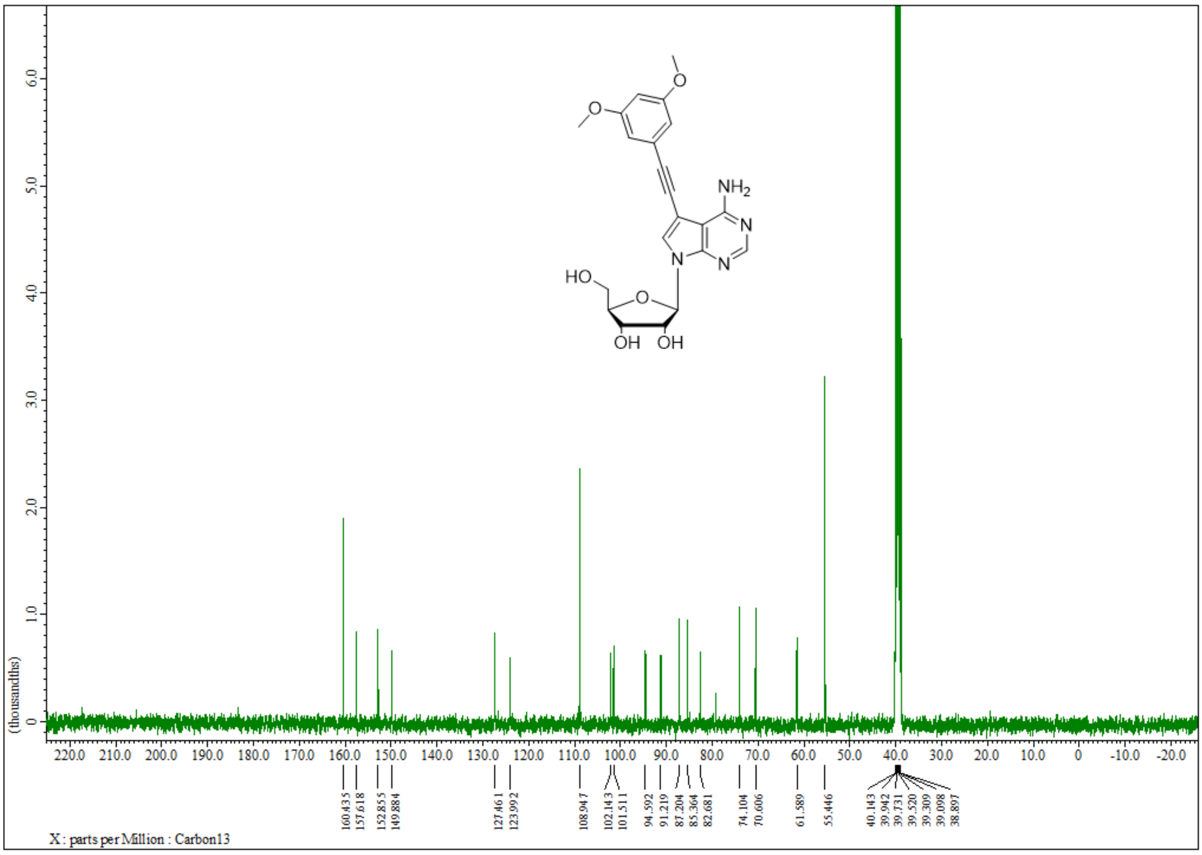

Figure. S8. ^{1}H-NMR (DMSO-d_6, 400 MHz) and ^{13}C-NMR (DMSO-d_6, 100 MHz) spectra of compound **5c**

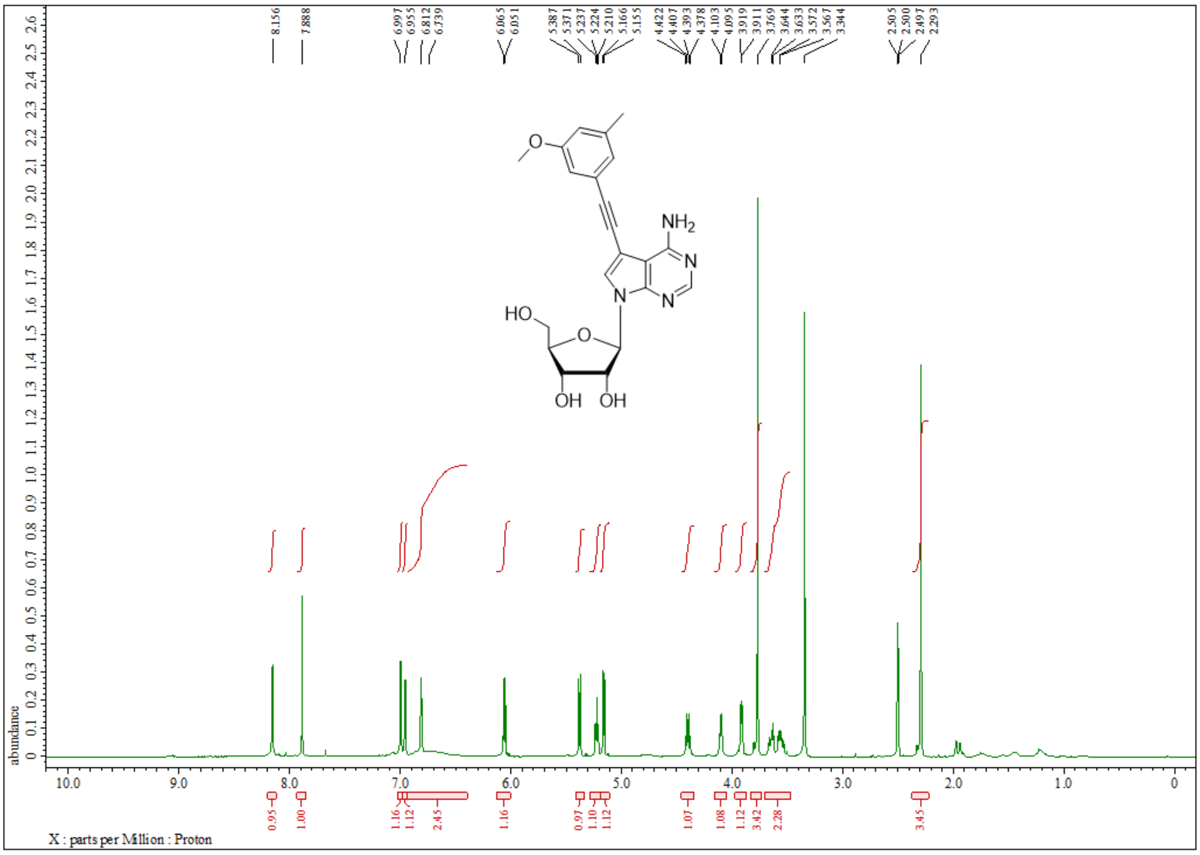

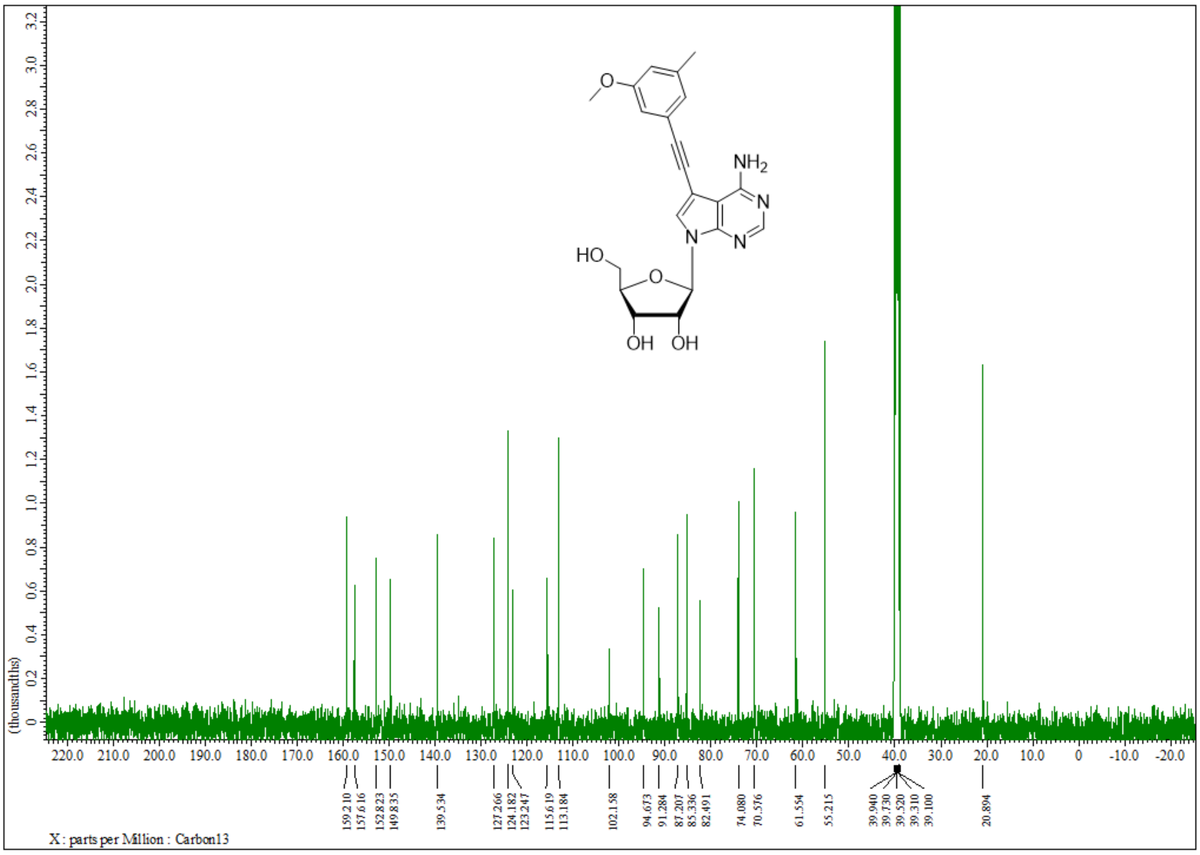

Figure. S9. ^{1}H-NMR (DMSO-d_6, 400 MHz) and ^{13}C-NMR (DMSO-d_6, 100 MHz) spectra of compound **5d**

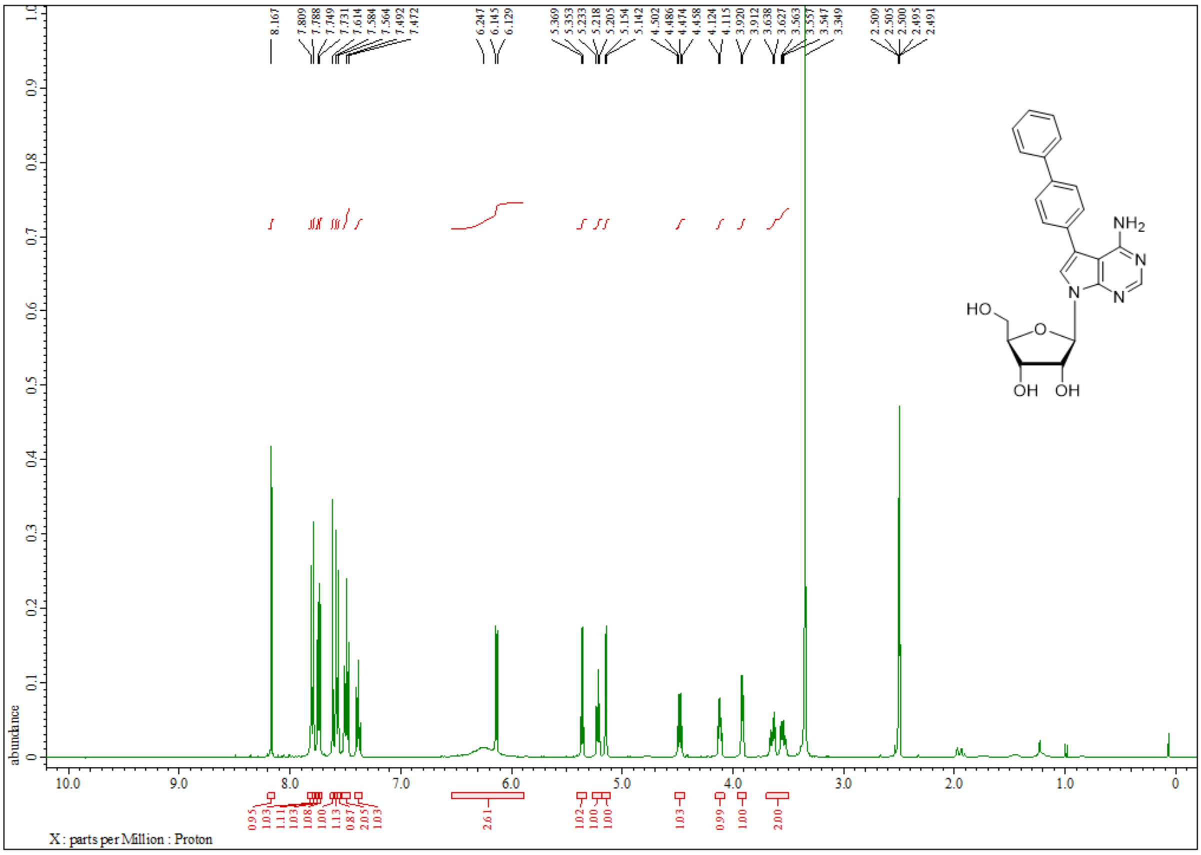

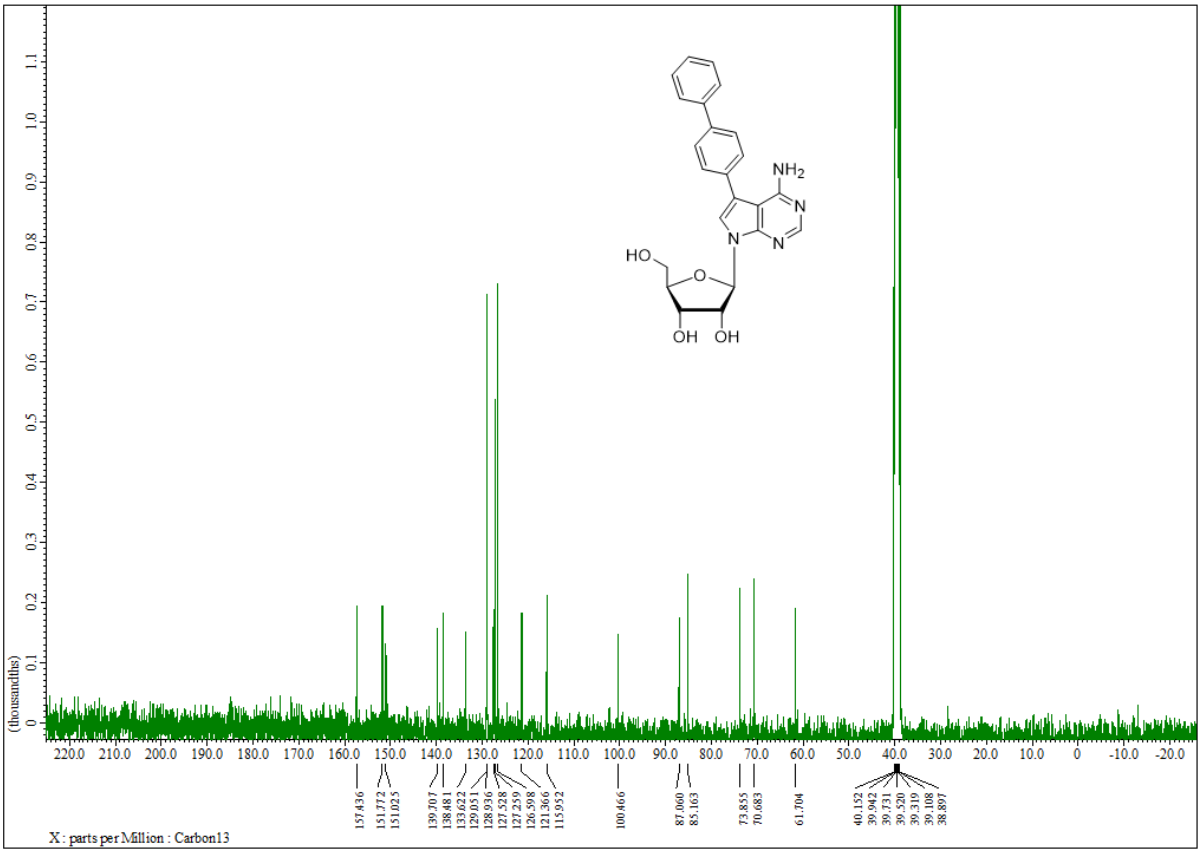

Figure. S10. ^{1}H-NMR (DMSO-d_6, 400 MHz) and ^{13}C-NMR (DMSO-d_6, 100 MHz) spectra of compound **6a**

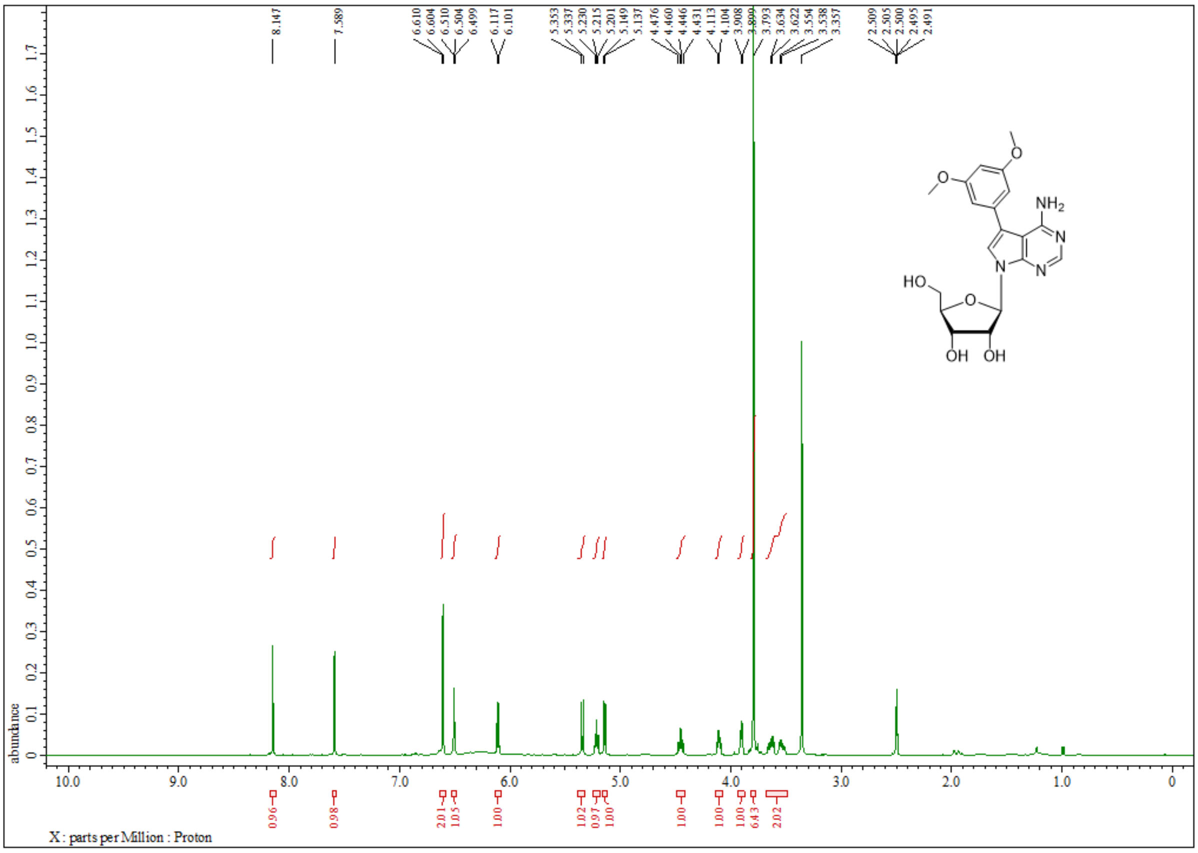

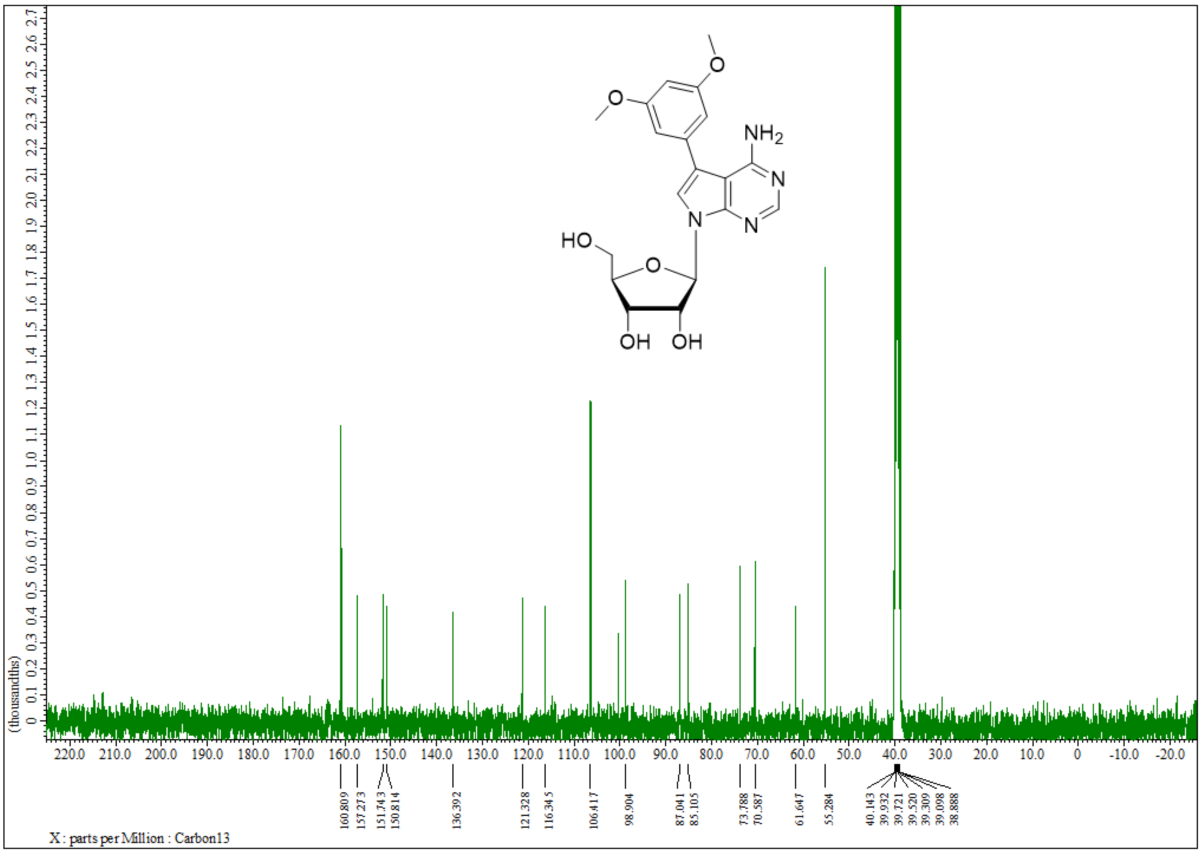

Figure. S11. ^{1}H-NMR (DMSO-d_6, 400 MHz) and ^{13}C-NMR (DMSO-d_6, 100 MHz) spectra of compound **6b**

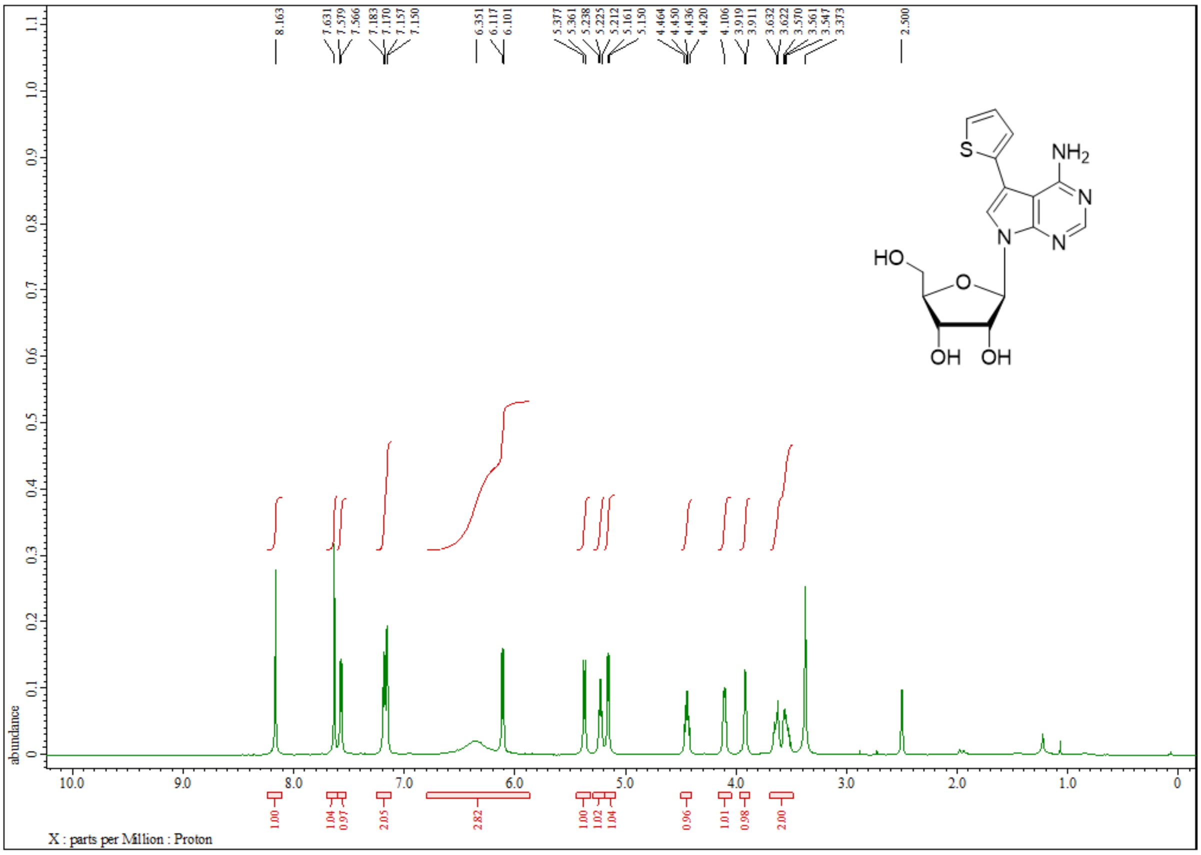

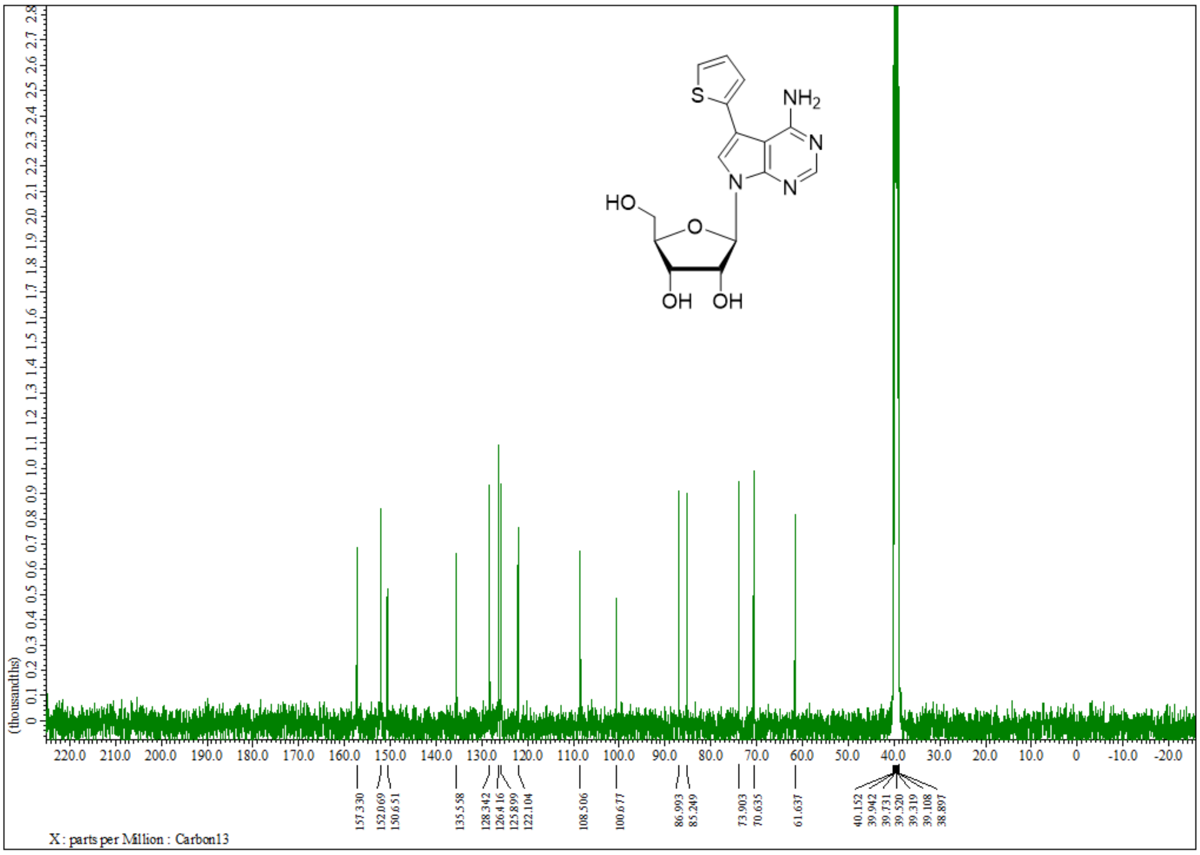

Figure. S12. ^{1}H-NMR (DMSO-d_6, 400 MHz) and ^{13}C-NMR (DMSO-d_6, 100 MHz) spectra of compound **6c**

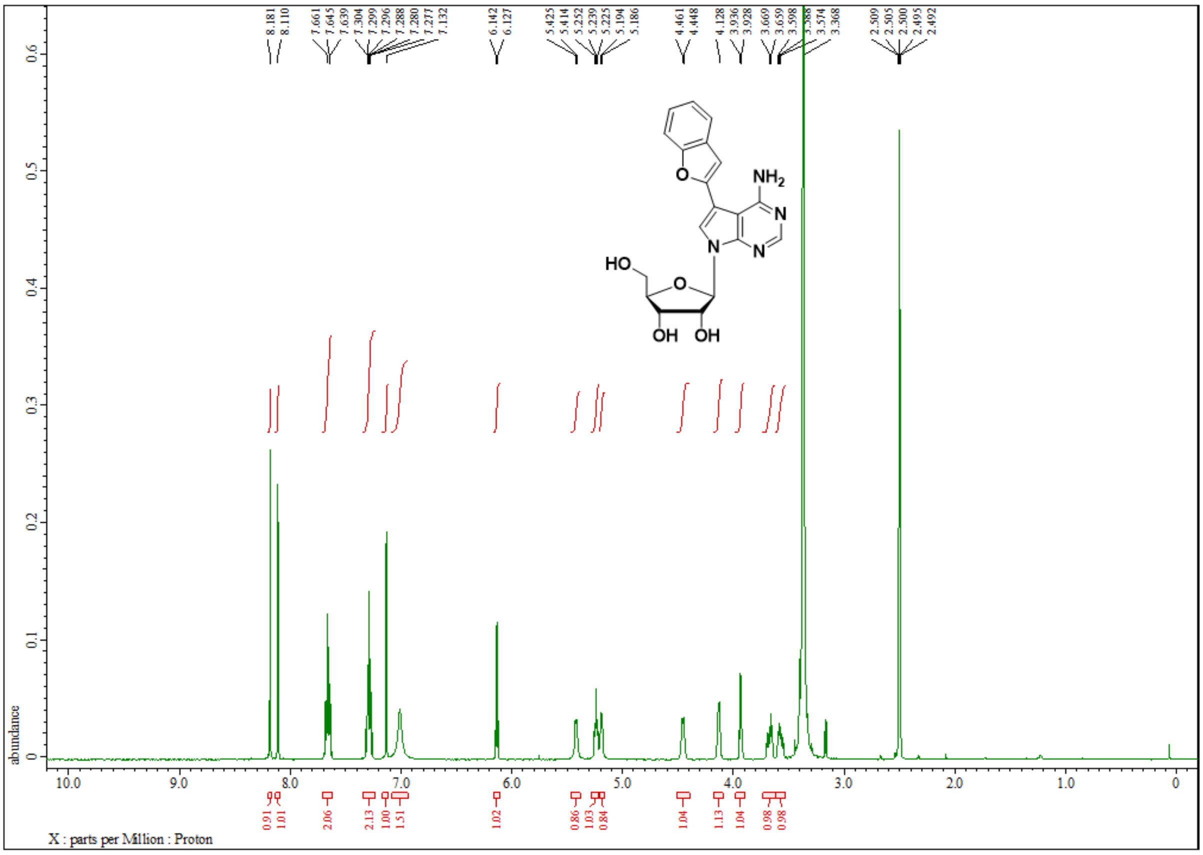

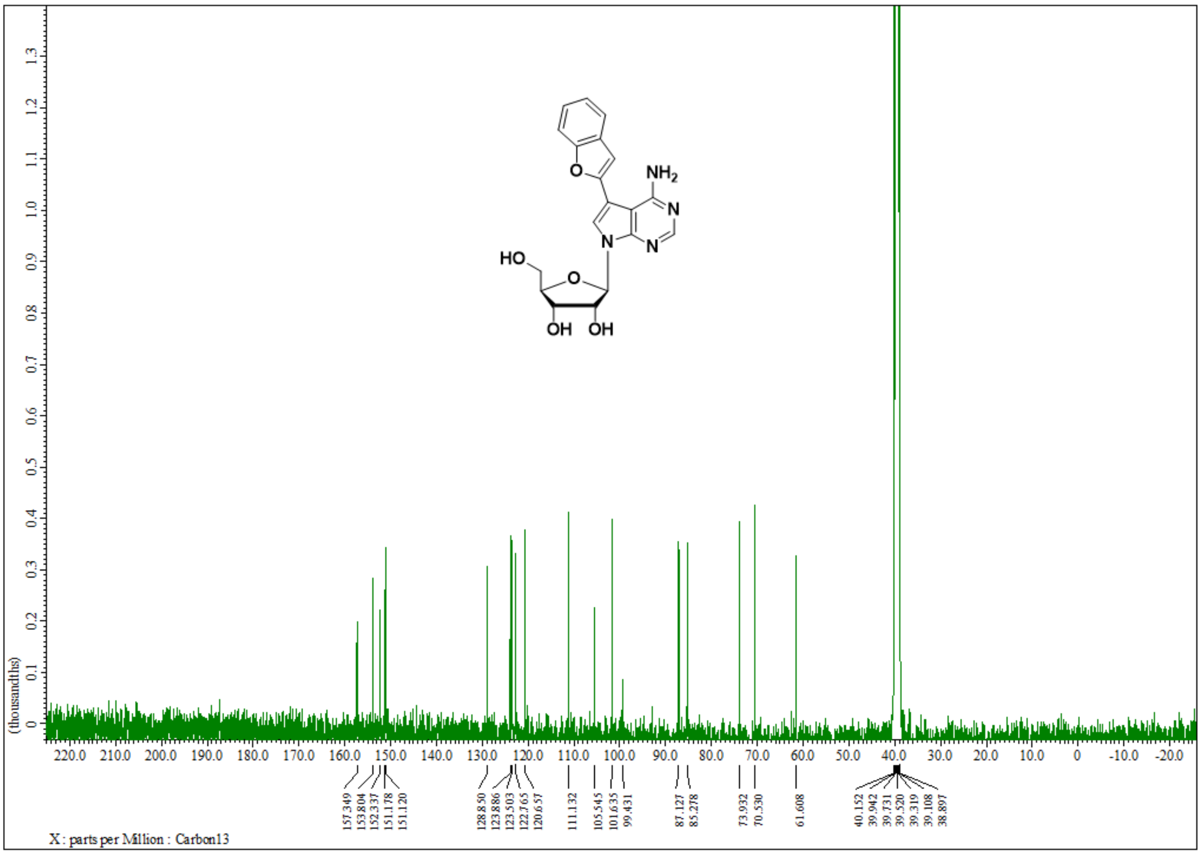

Figure S13. ^{1}H-NMR (DMSO-d_6, 400 MHz) and ^{13}C-NMR (DMSO-d_6, 100 MHz) spectra of compound **6d**

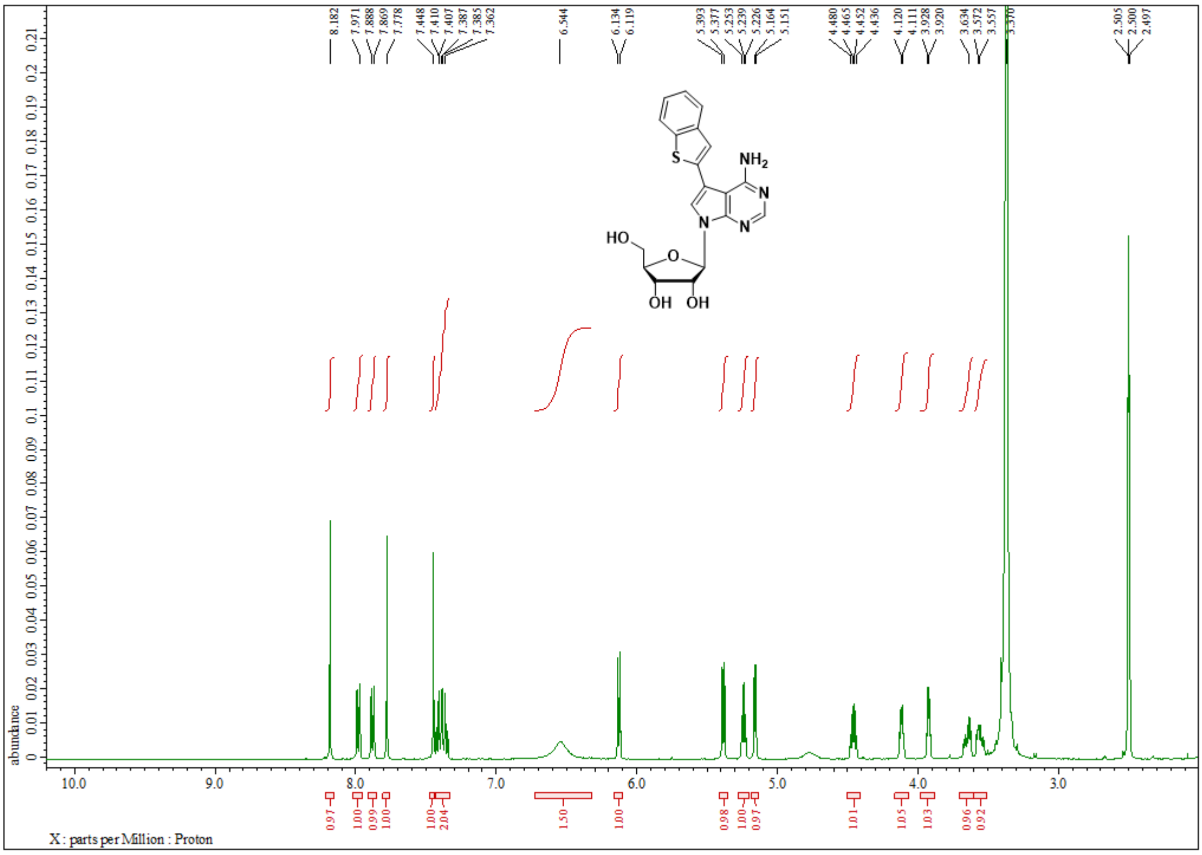

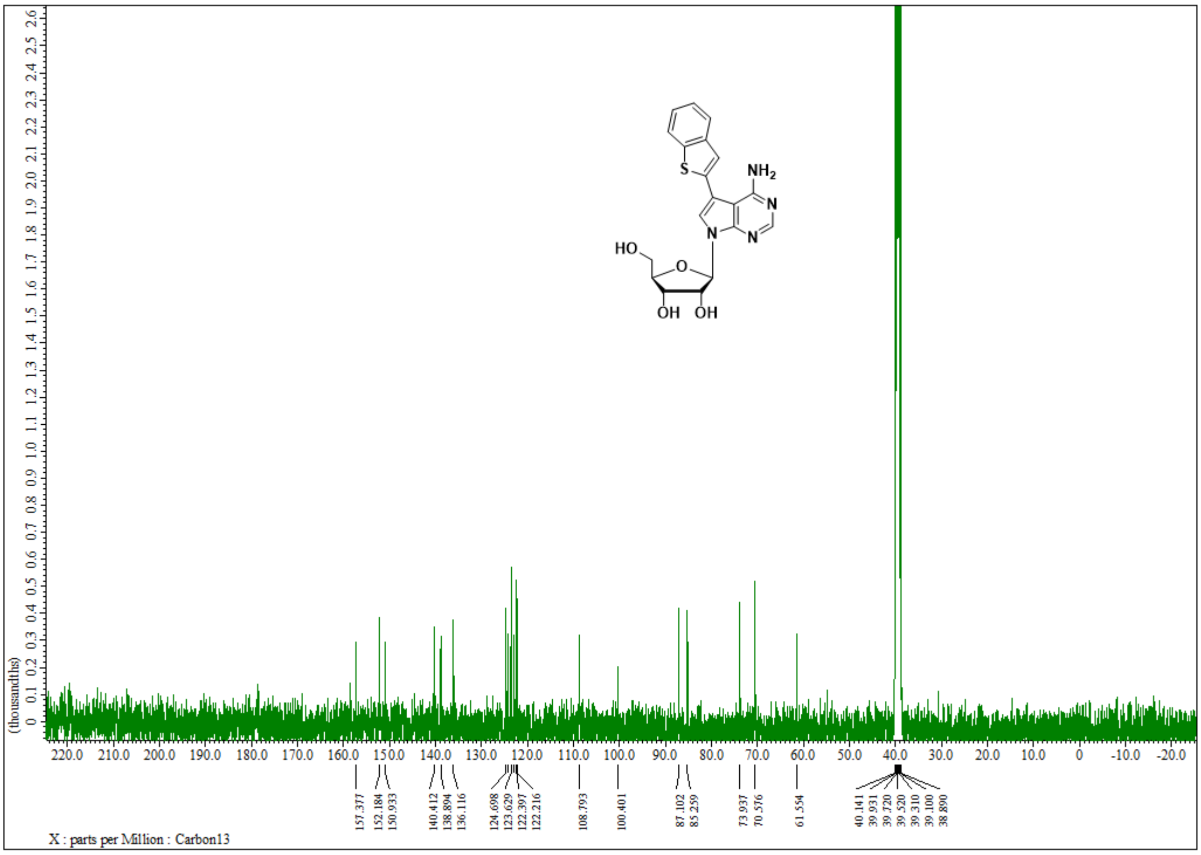

Figure S14. ^{1}H-NMR (DMSO-d_6, 400 MHz) and ^{13}C-NMR (DMSO-d_6, 100 MHz) spectra of compound **6e**

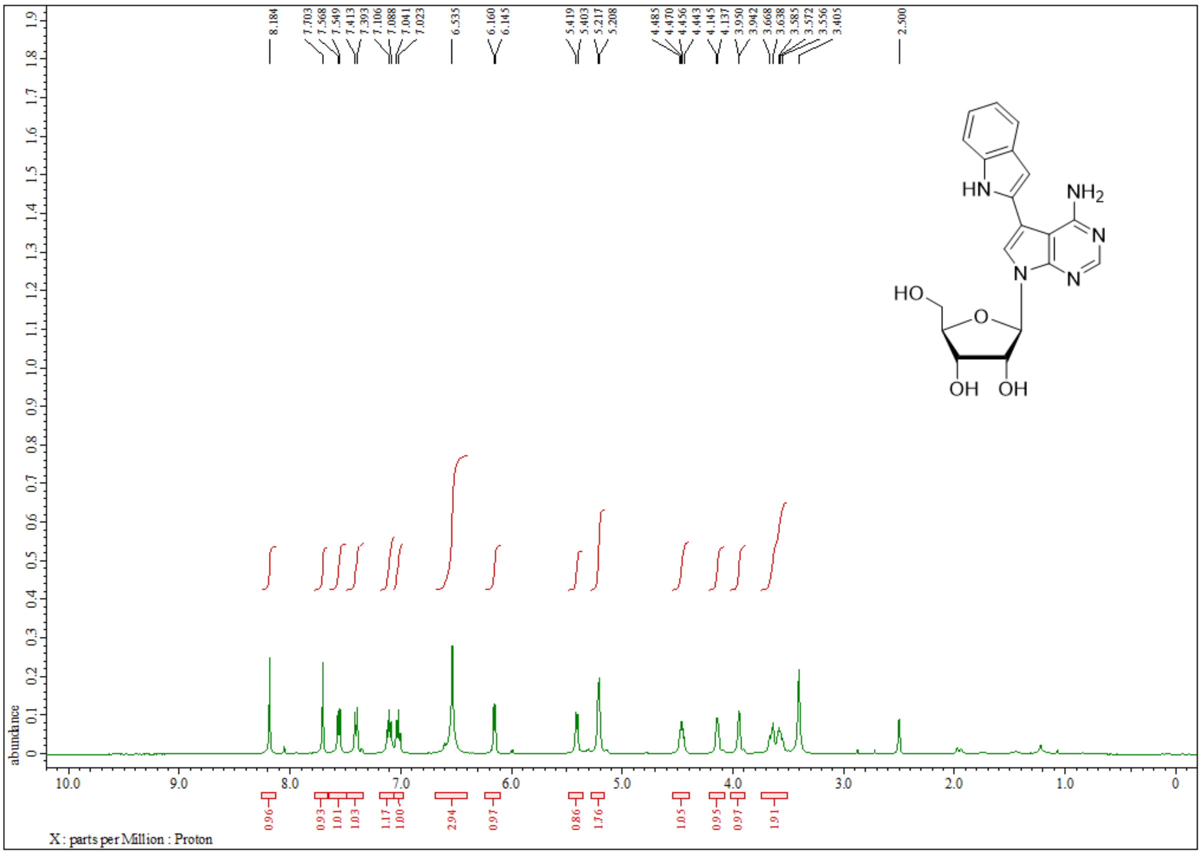

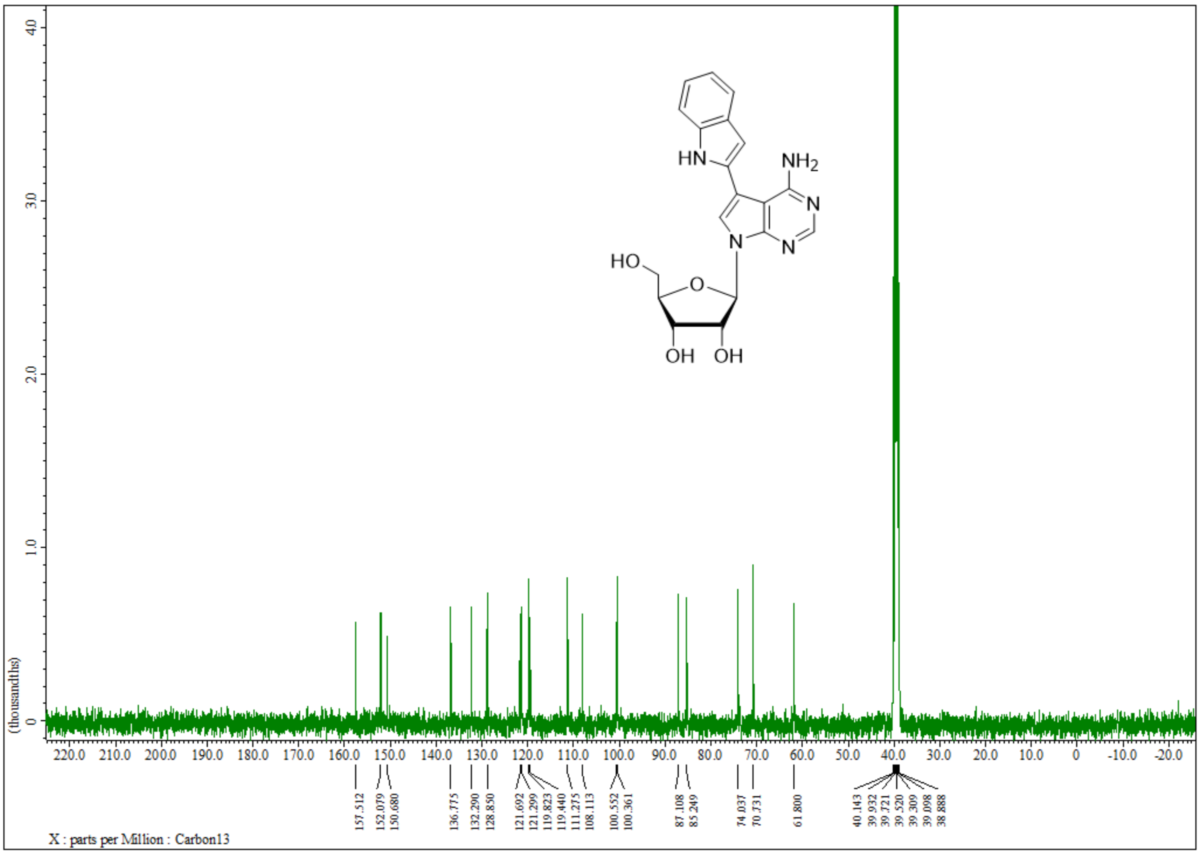

Figure S15. ^{1}H-NMR (DMSO-d_6, 400 MHz) and ^{13}C-NMR (DMSO-d_6, 100 MHz) spectra of compound **6f**

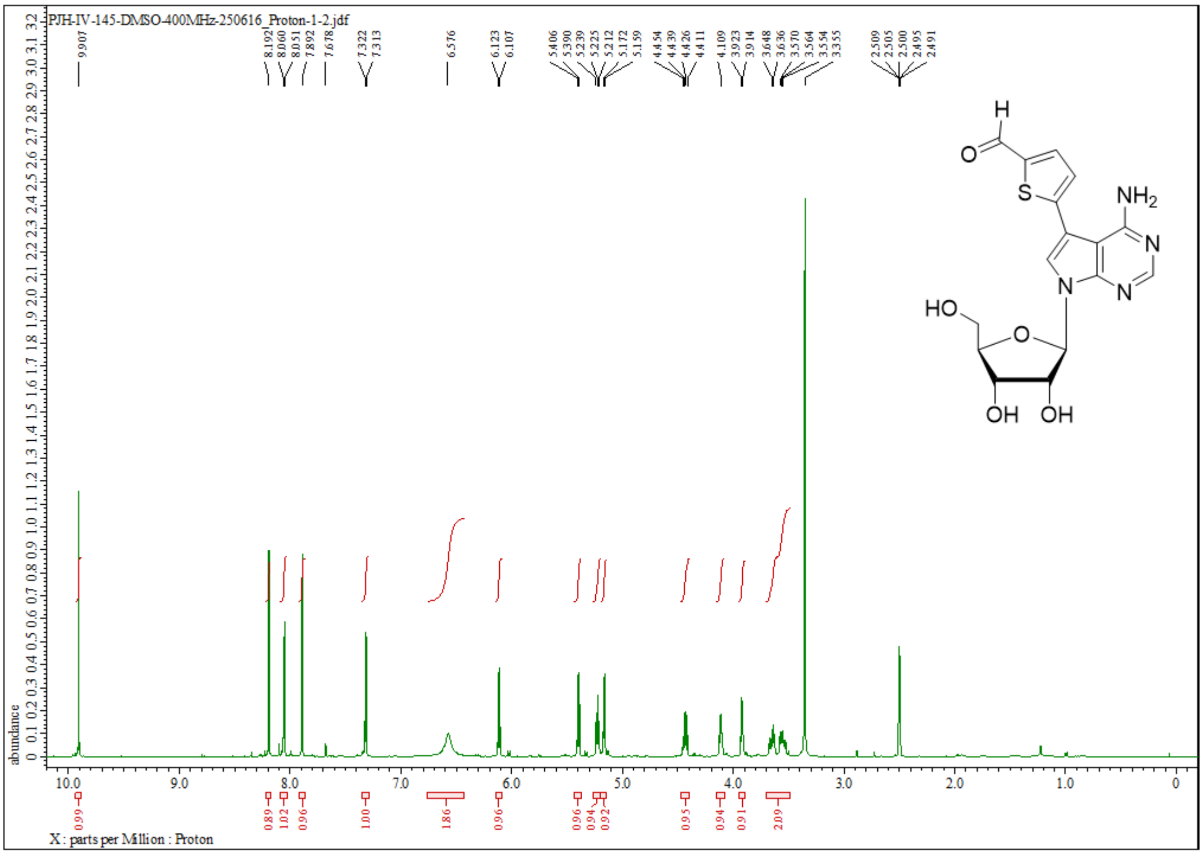

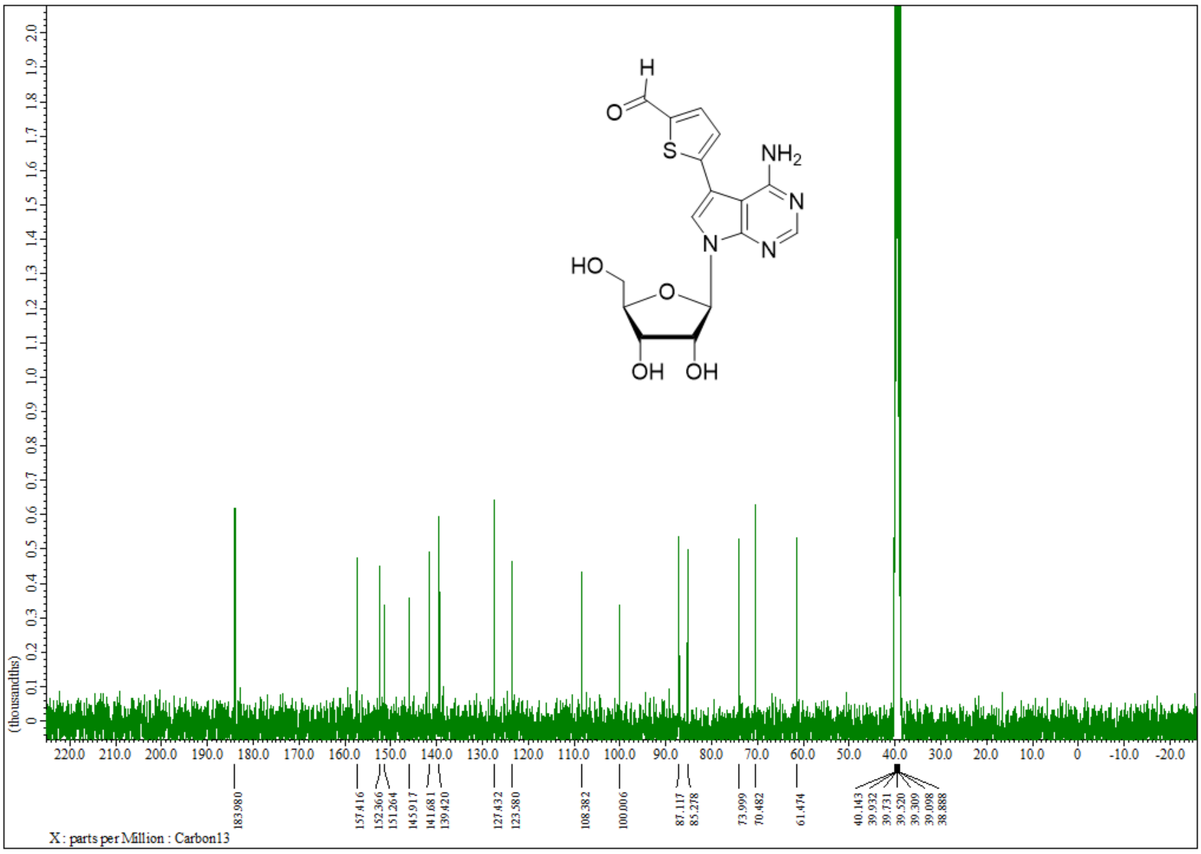

Figure S16. ^{1}H-NMR (DMSO-d_6, 400 MHz) and ^{13}C-NMR (DMSO-d_6, 100 MHz) spectra of compound **6g**

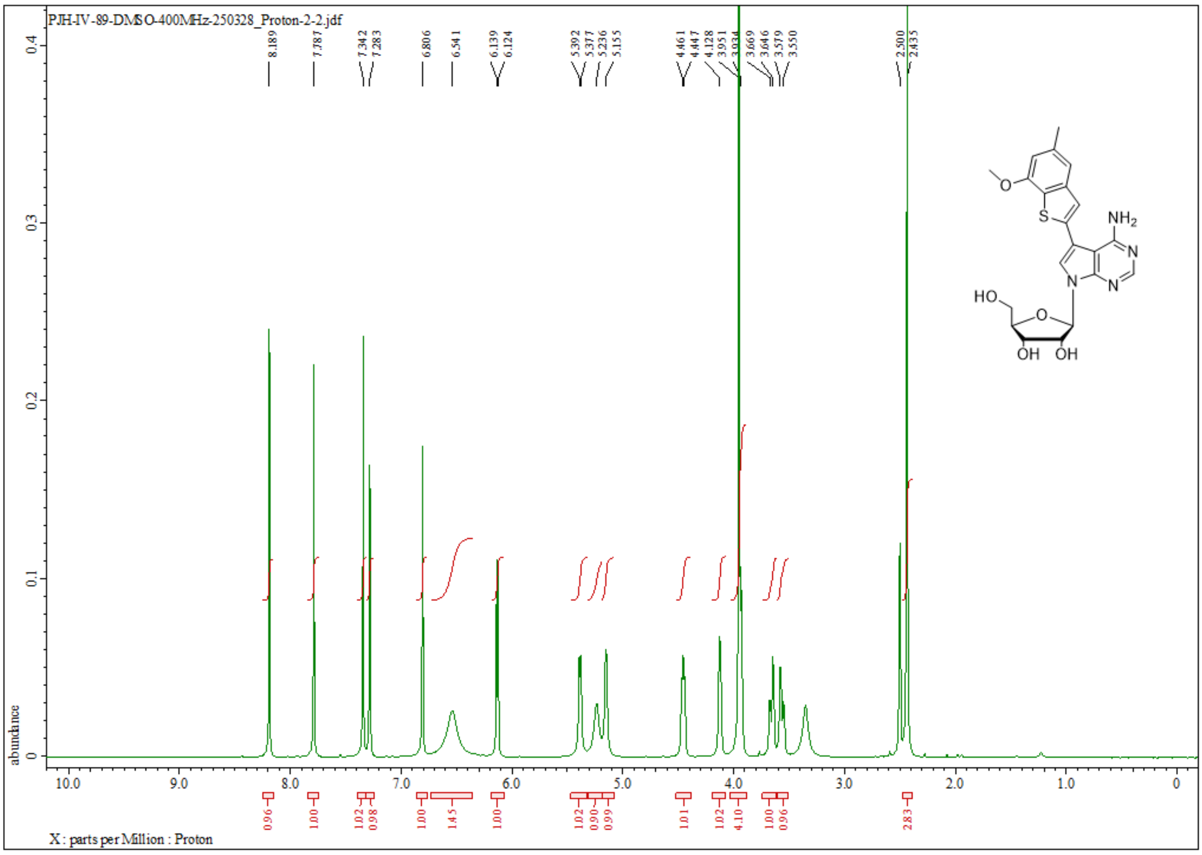

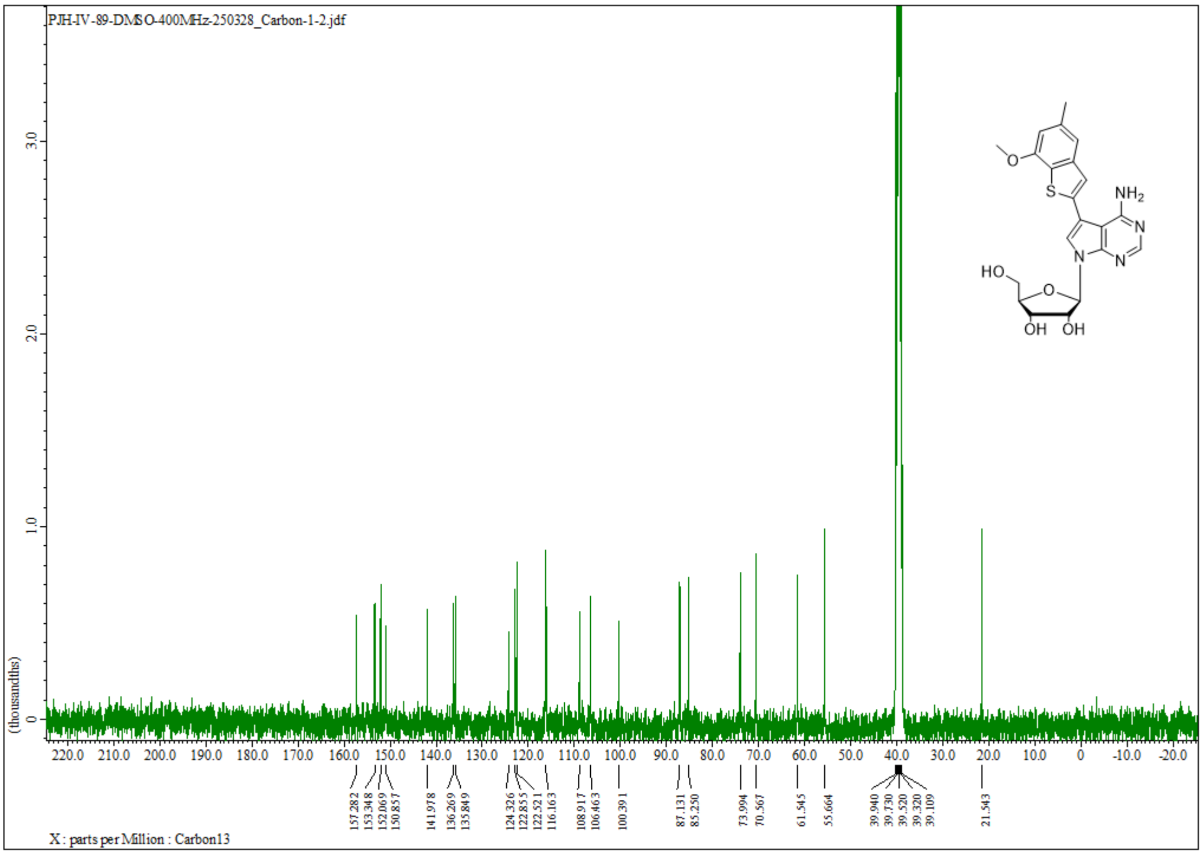

Figure S17. ^{1}H-NMR (DMSO-d_6, 400 MHz) and ^{13}C-NMR (DMSO-d_6, 100 MHz) spectra of compound **6h**

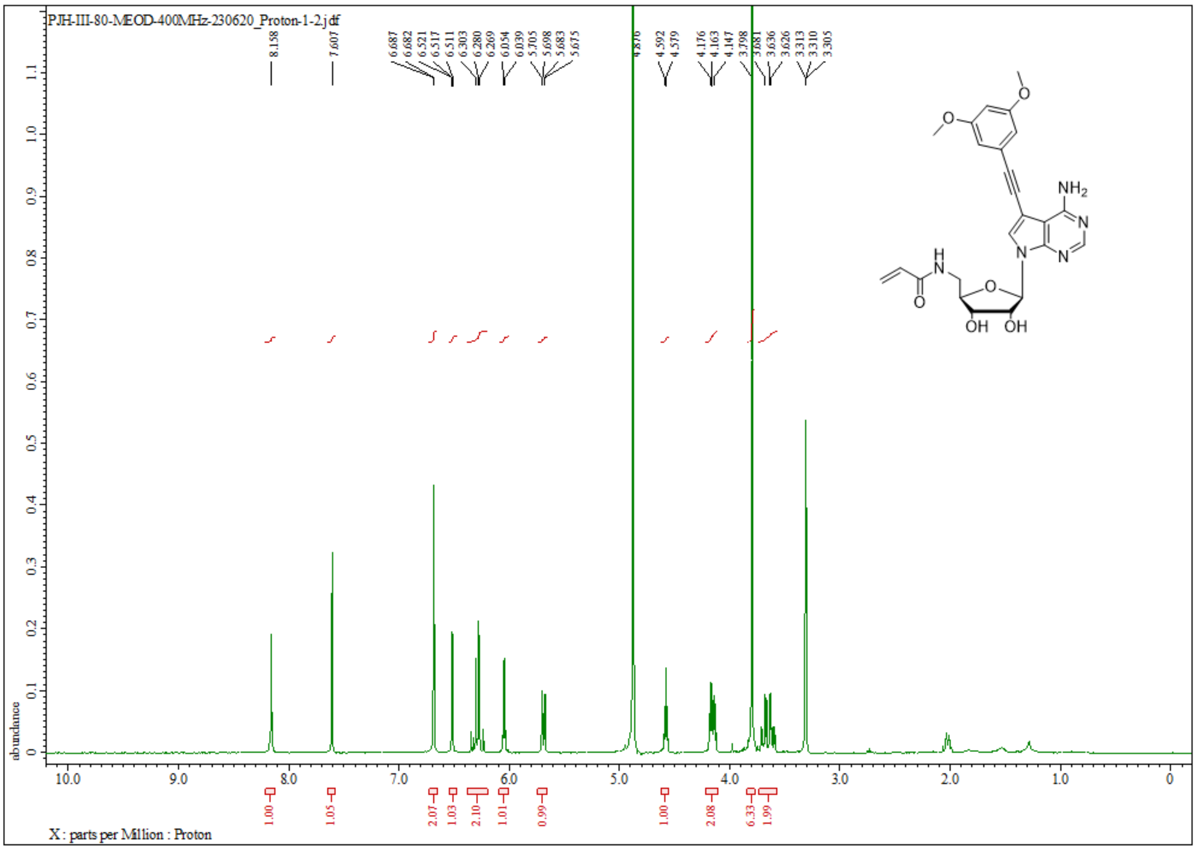

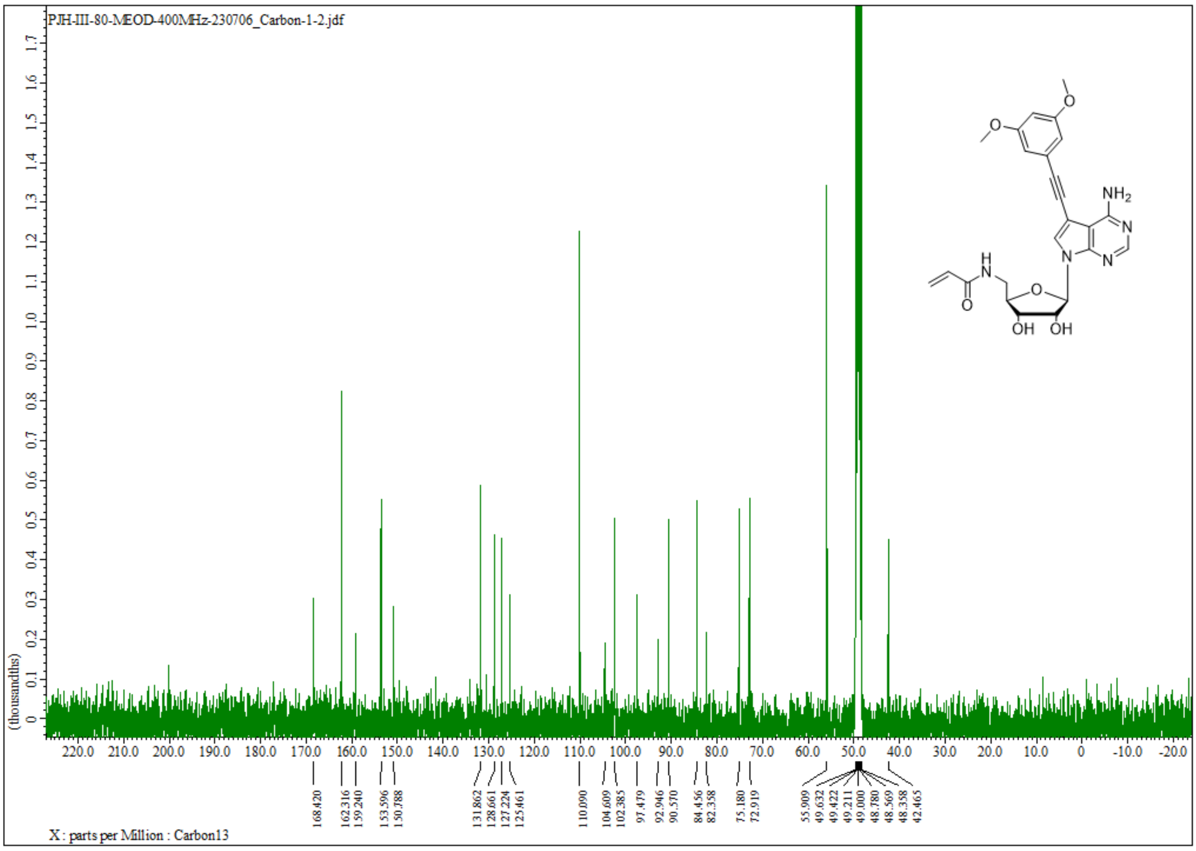

Figure S18. ^{1}H-NMR (MeOD, 400 MHz) and ^{13}C-NMR (MeOD, 100 MHz) spectra of compound **13a**

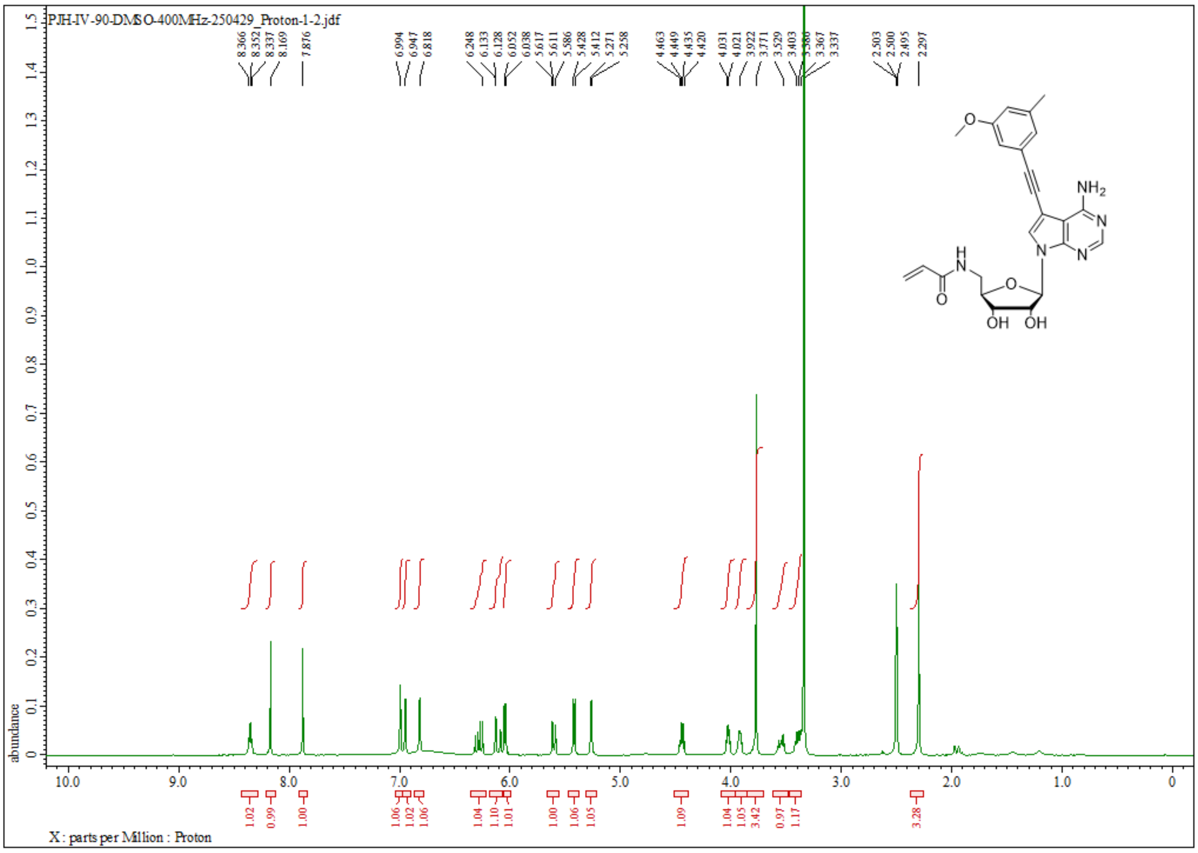

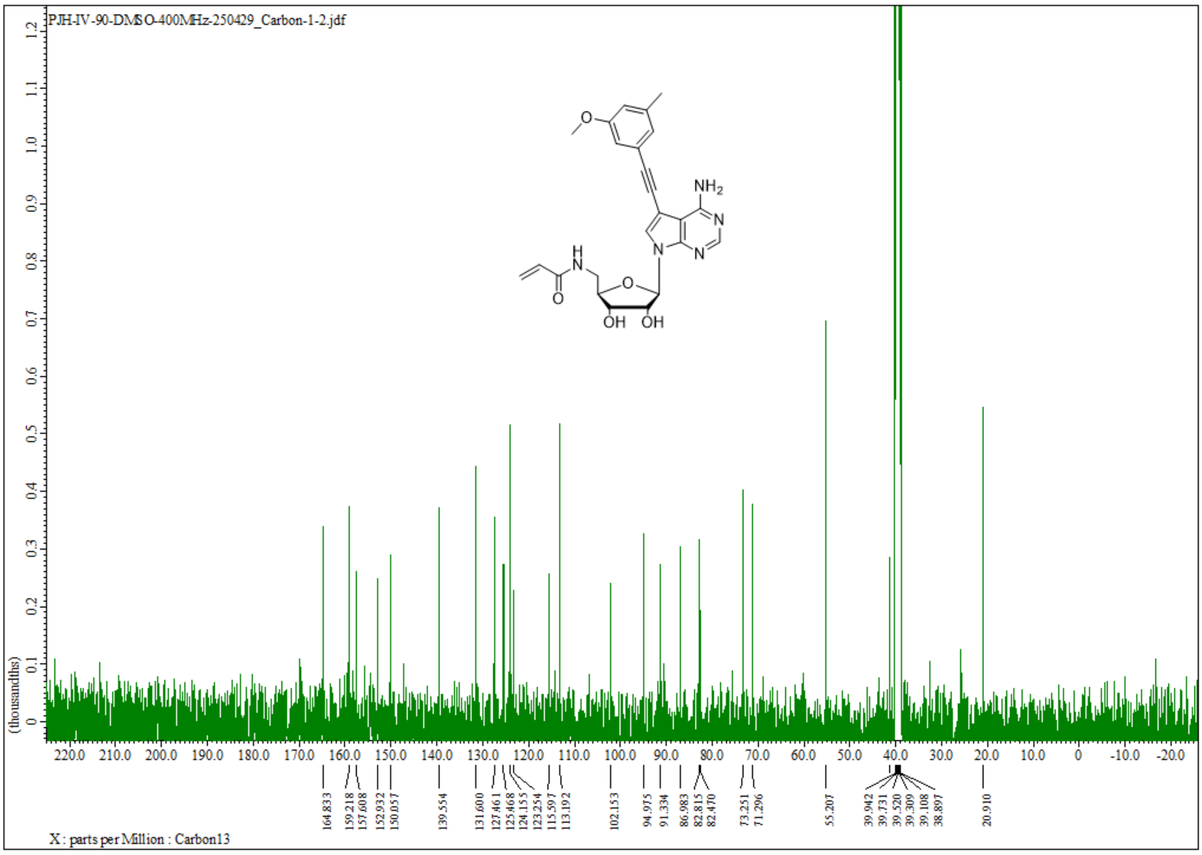

Figure S19. ^{1}H-NMR (DMSO-d_6, 400 MHz) and ^{13}C-NMR (DMSO-d_6, 100 MHz) spectra of compound **13b**

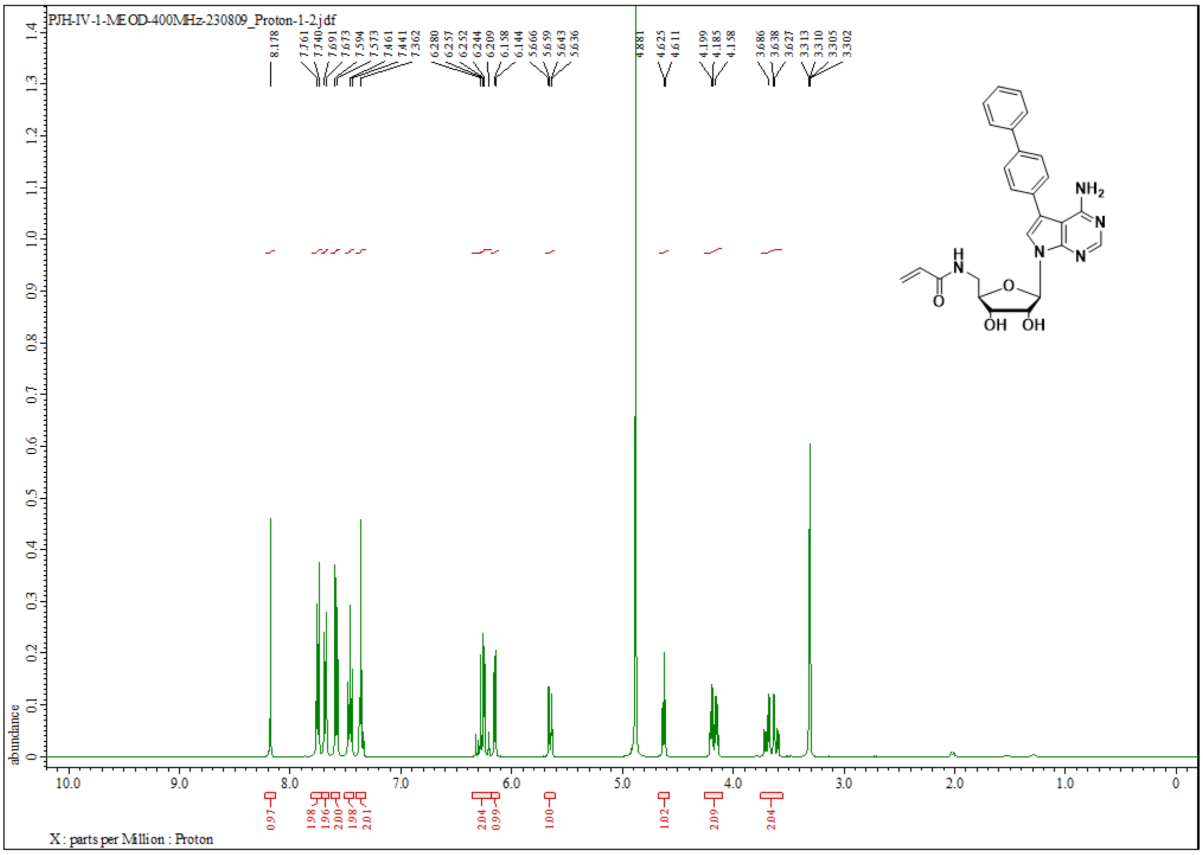

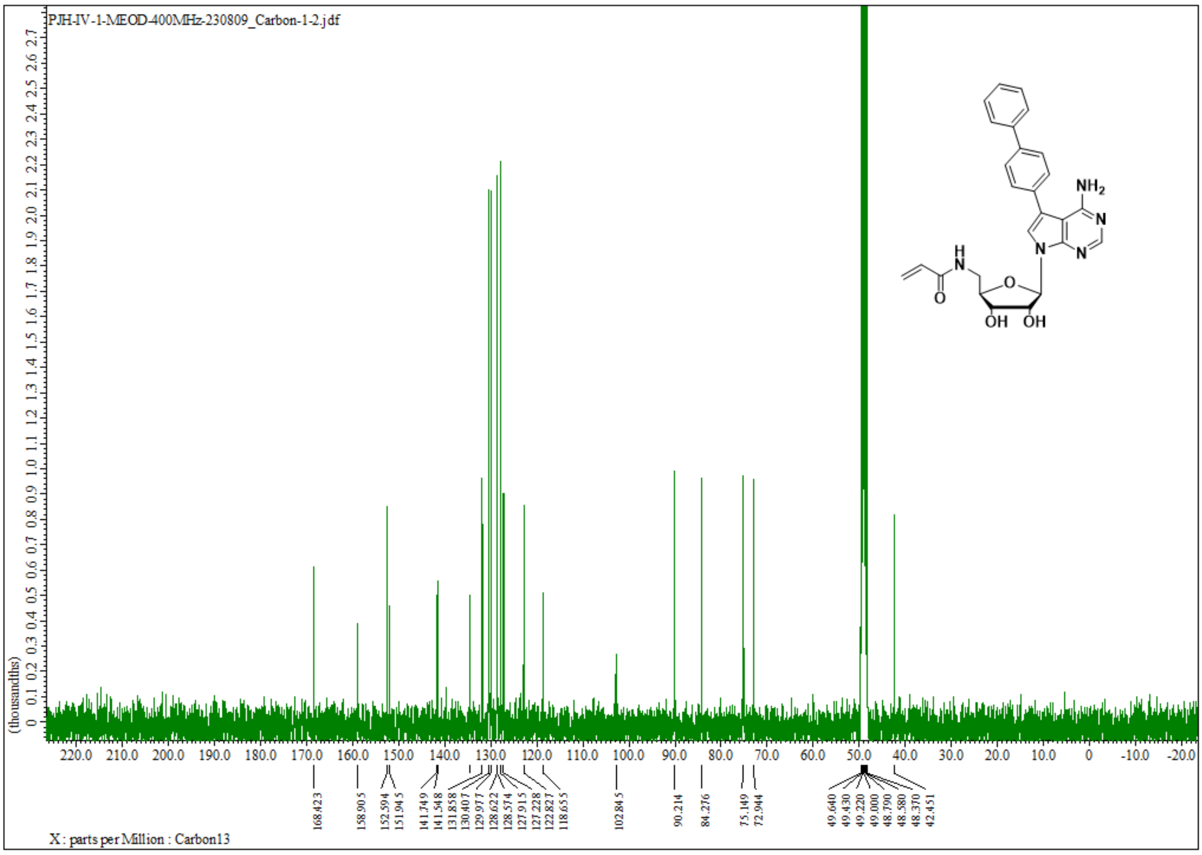

Figure S20. ^{1}H-NMR (MeOD, 400 MHz) and ^{13}C-NMR (MeOD, 100 MHz) spectra of compound **13c**

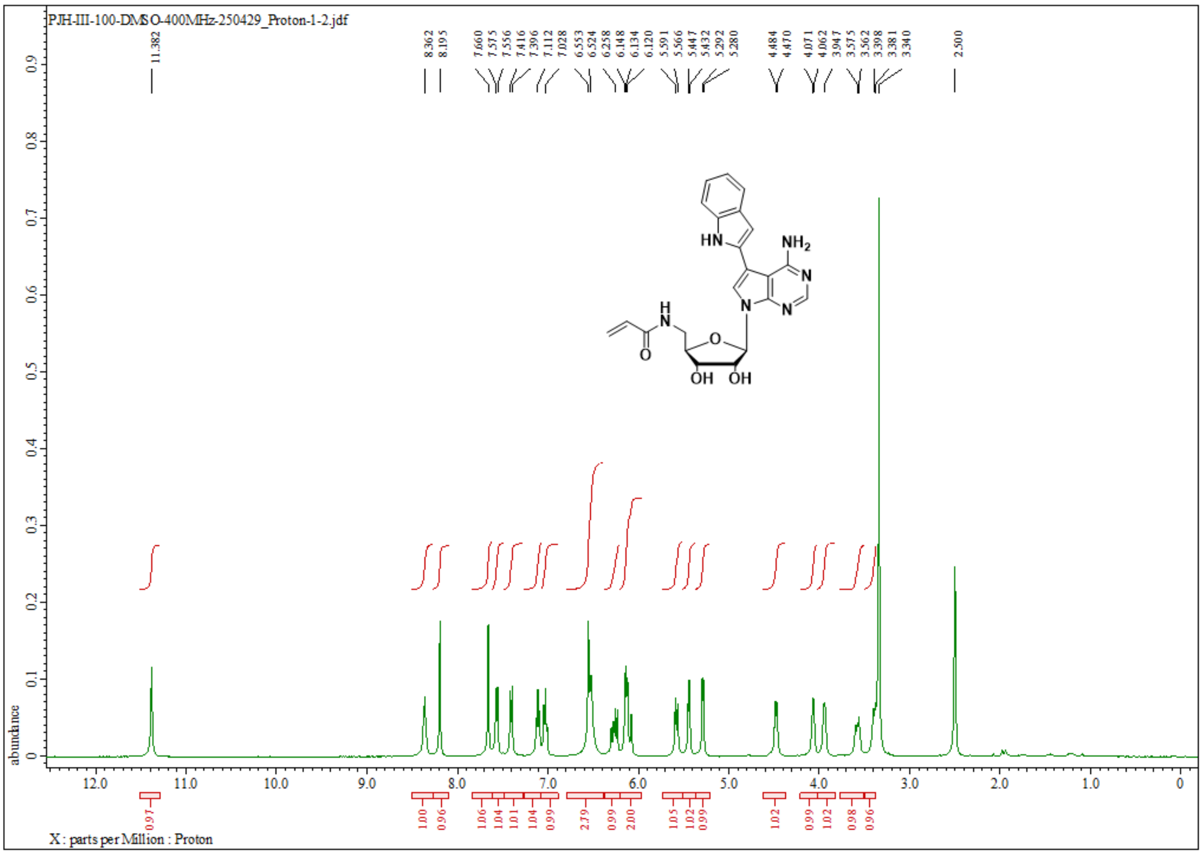

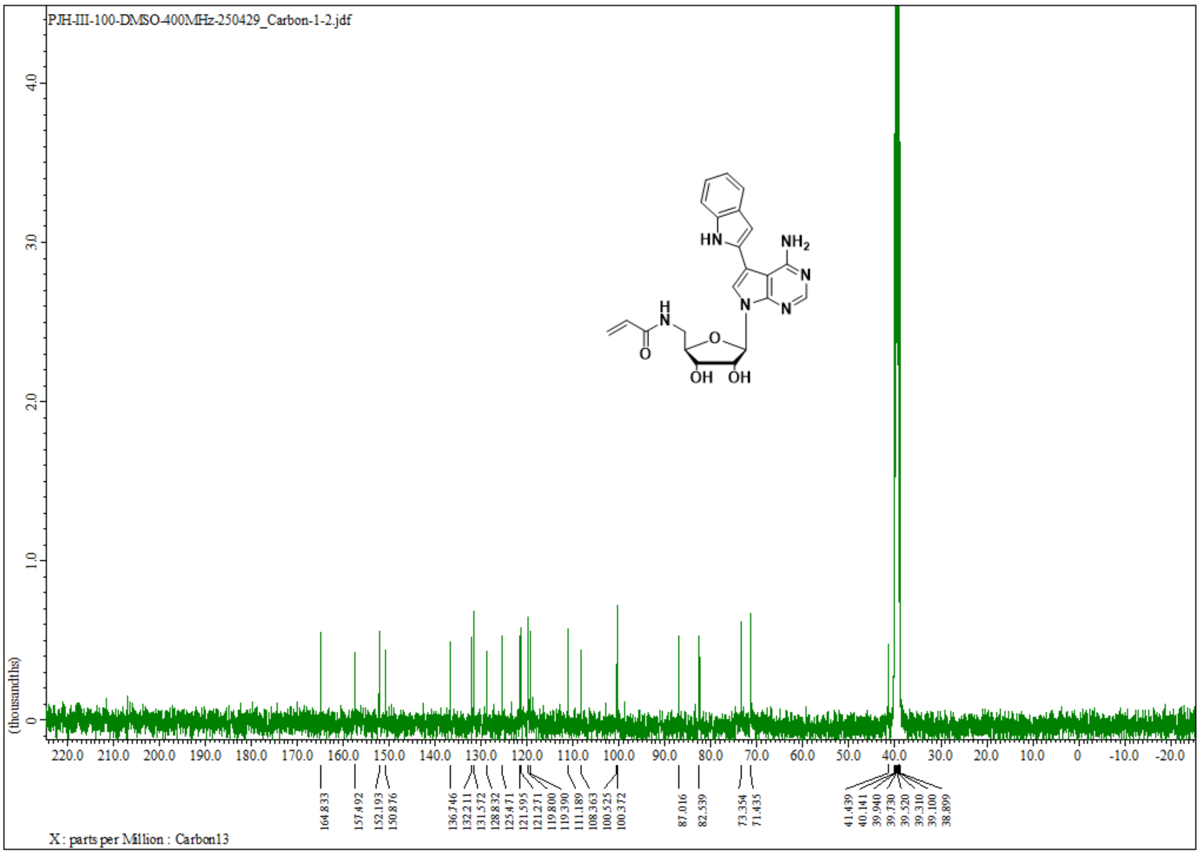

Figure S21. ^{1}H-NMR (DMSO-d_6, 400 MHz) and ^{13}C-NMR (DMSO-d_6, 100 MHz) spectra of compound **13d**

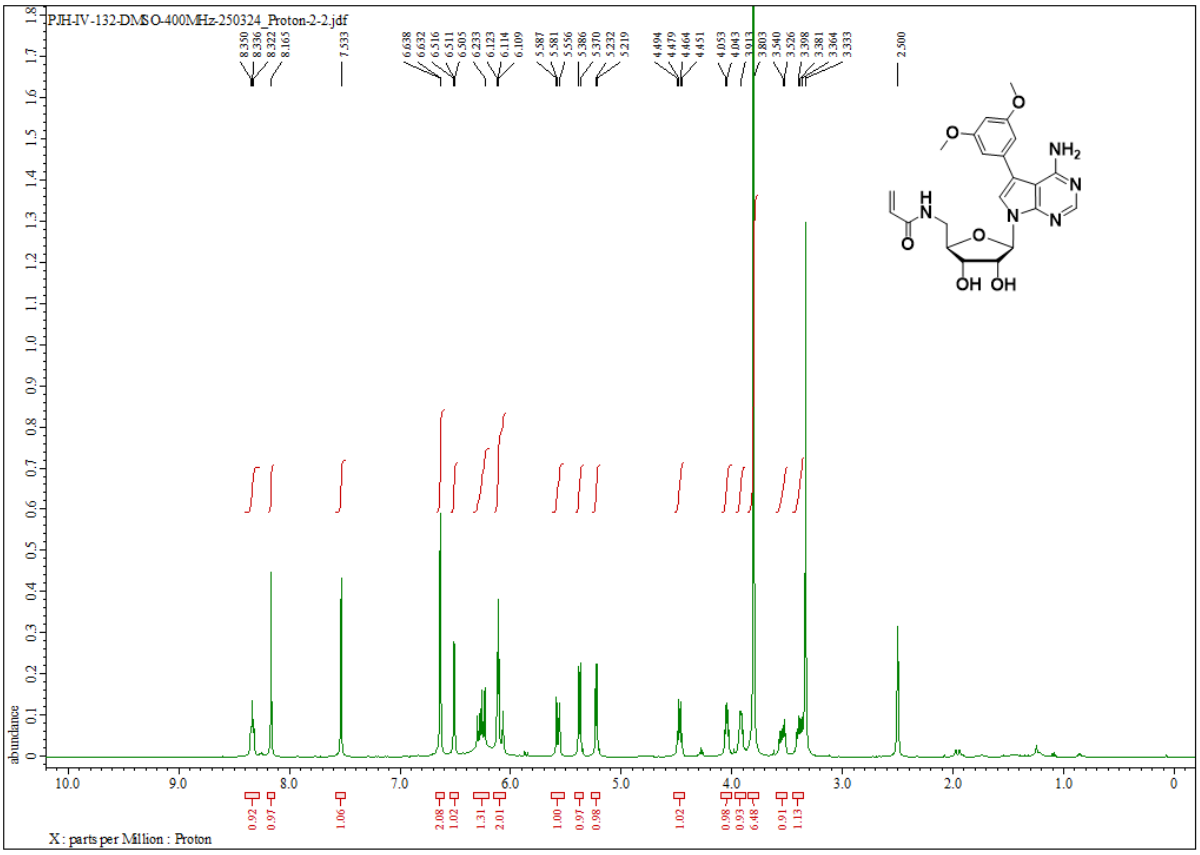

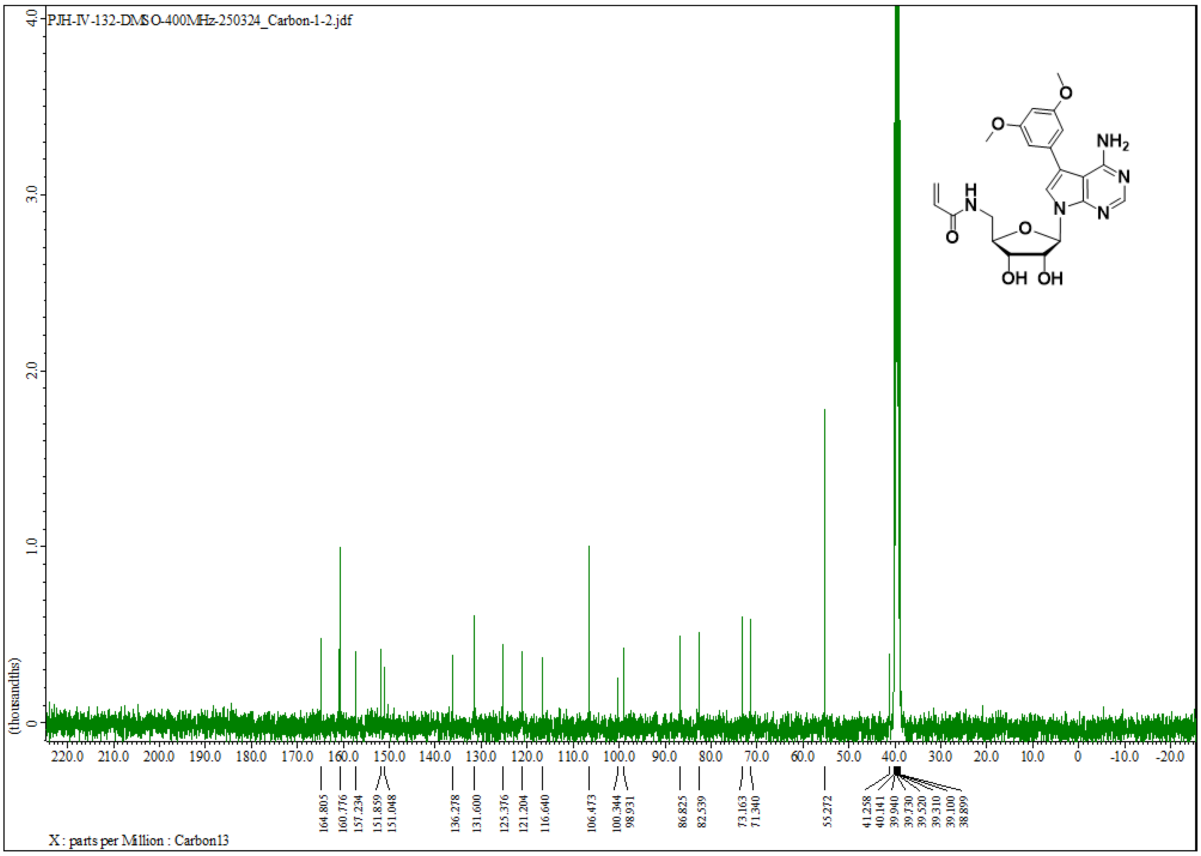

Figure S22. ^{1}H-NMR (DMSO-d_6, 400 MHz) and ^{13}C-NMR (DMSO-d_6, 100 MHz) spectra of compound **13e**

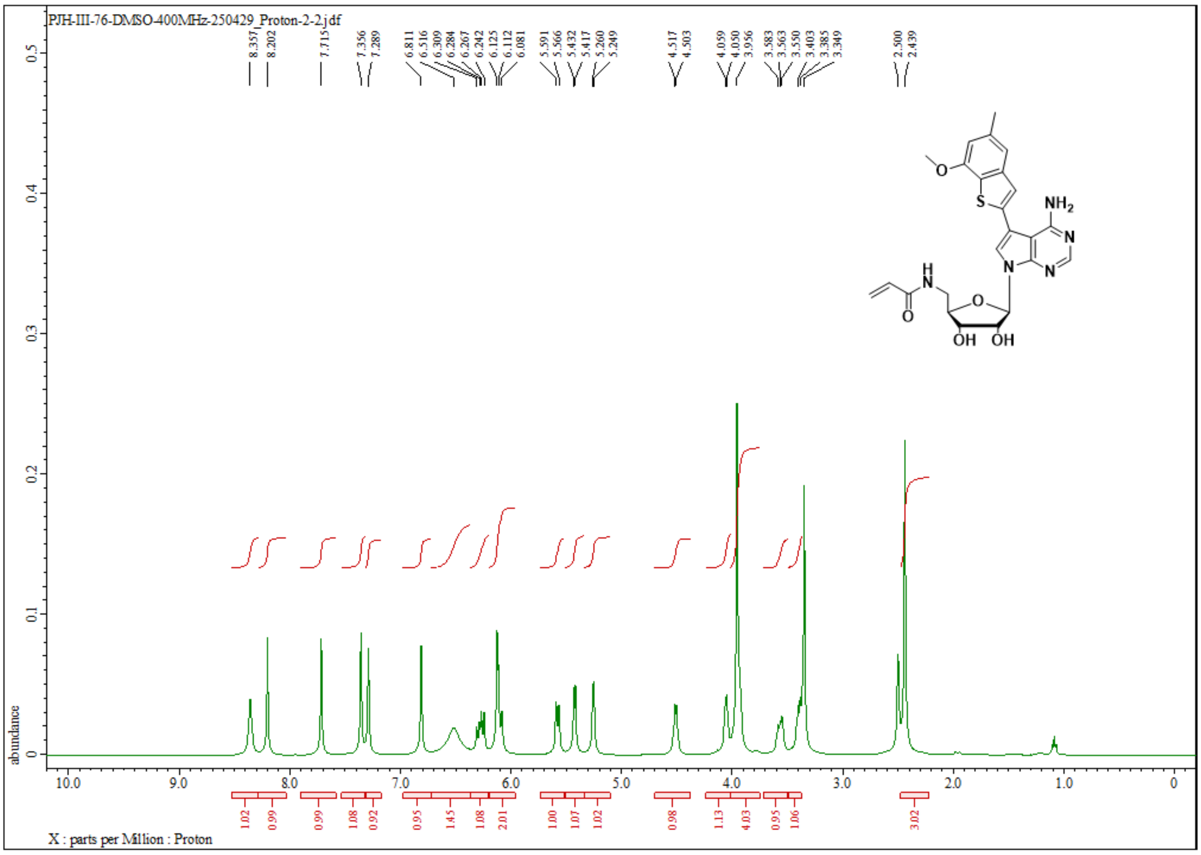

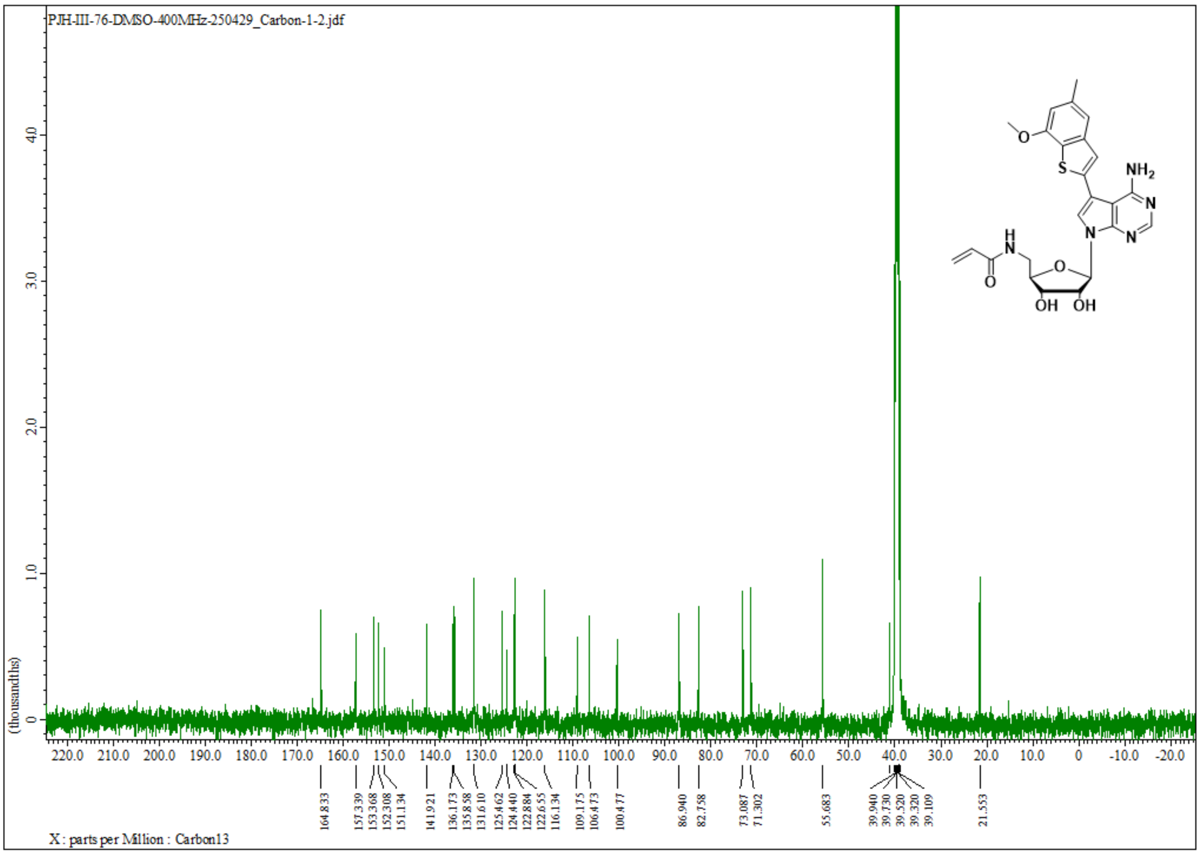

Figure S23. ^{1}H-NMR (DMSO-d_6, 400 MHz) and ^{13}C-NMR (DMSO-d_6, 100 MHz) spectra of compound **13f**

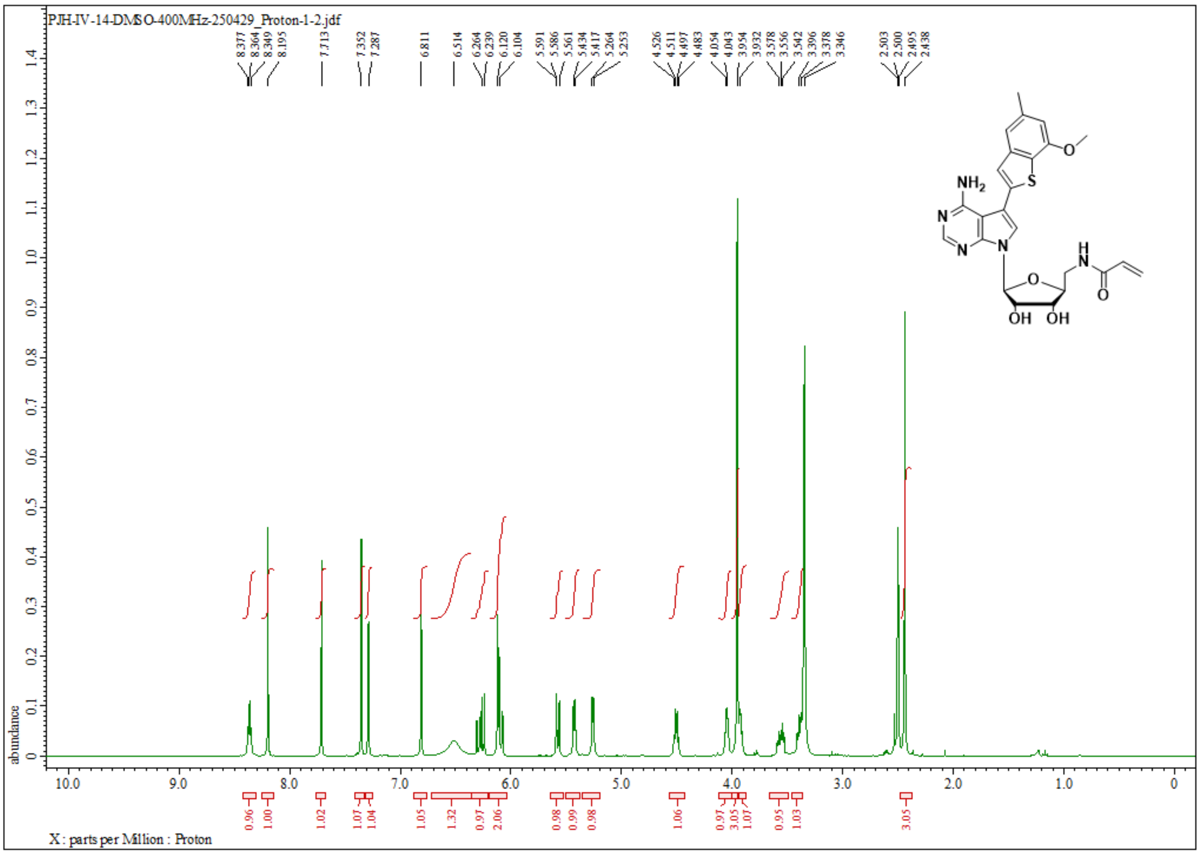

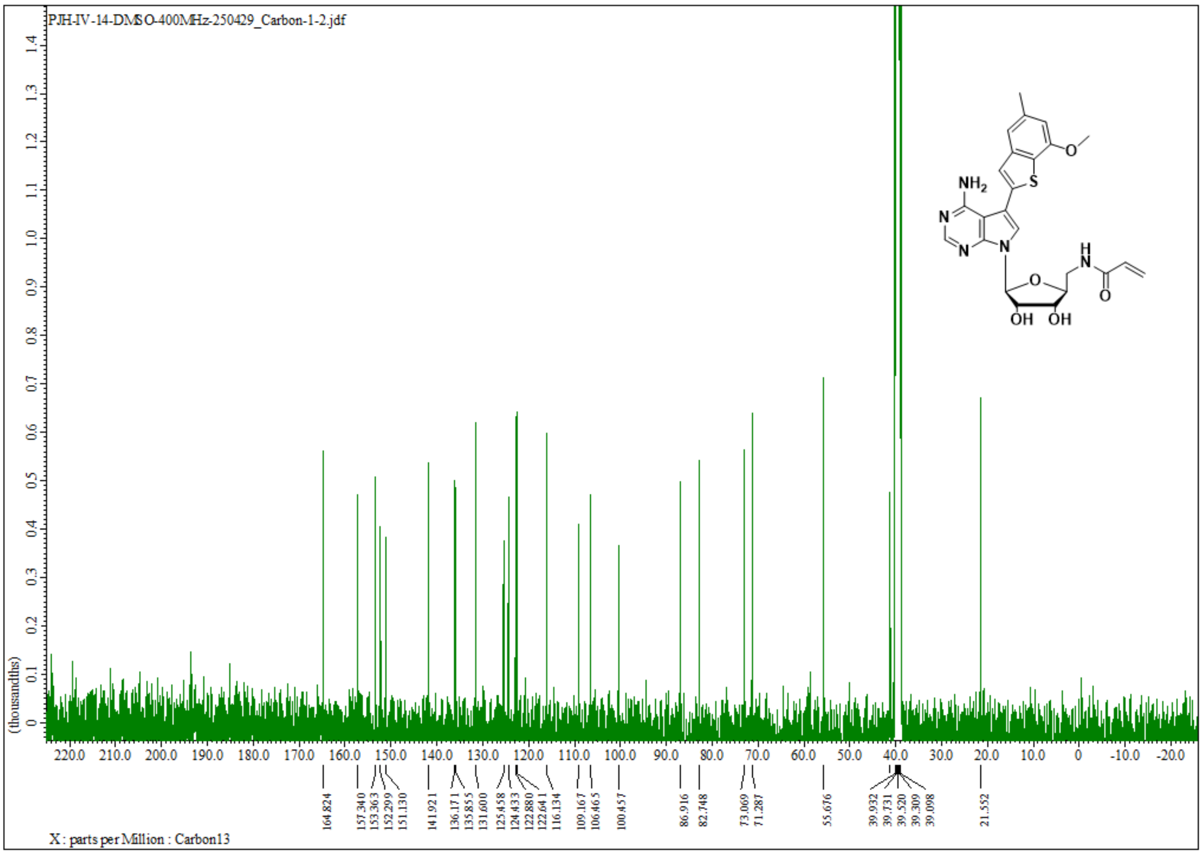

Figure S24. ^{1}H-NMR (DMSO-d_6, 400 MHz) and ^{13}C-NMR (DMSO-d_6, 100 MHz) spectra of compound ***ent*-13f**

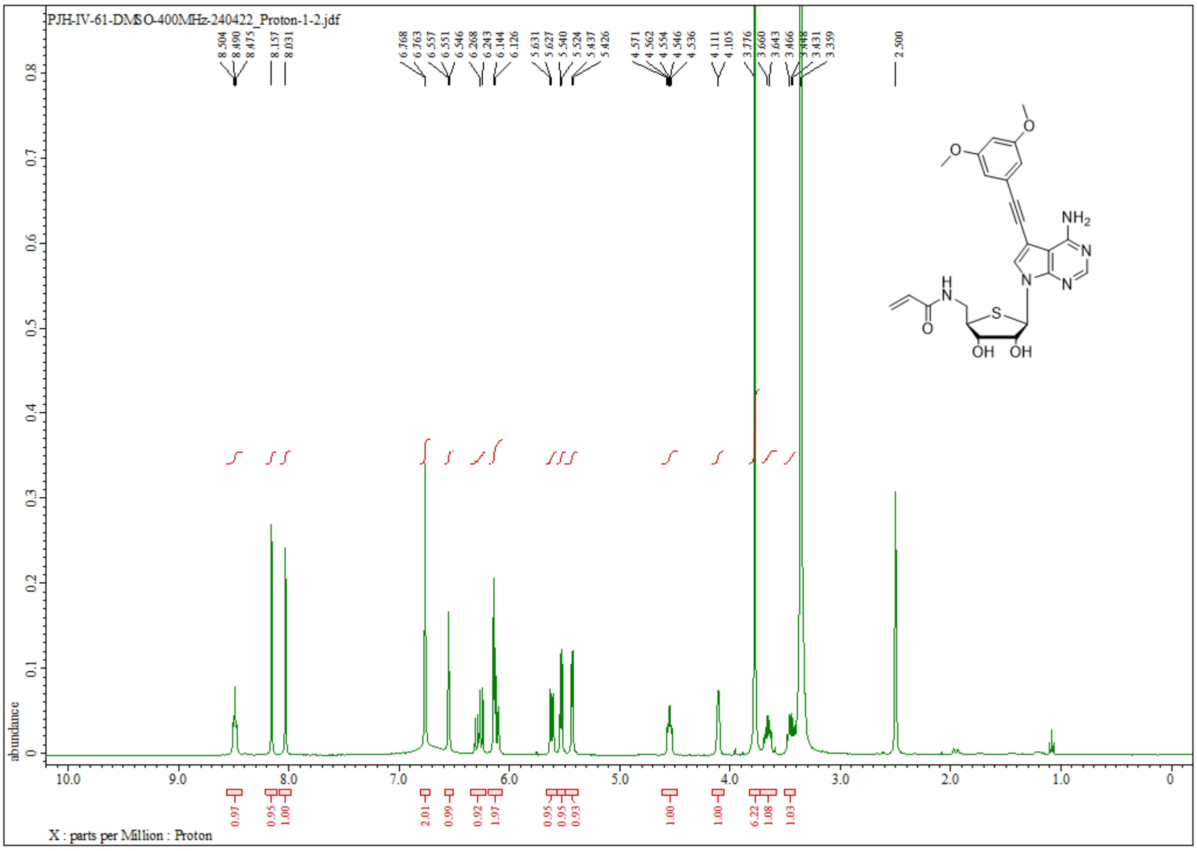

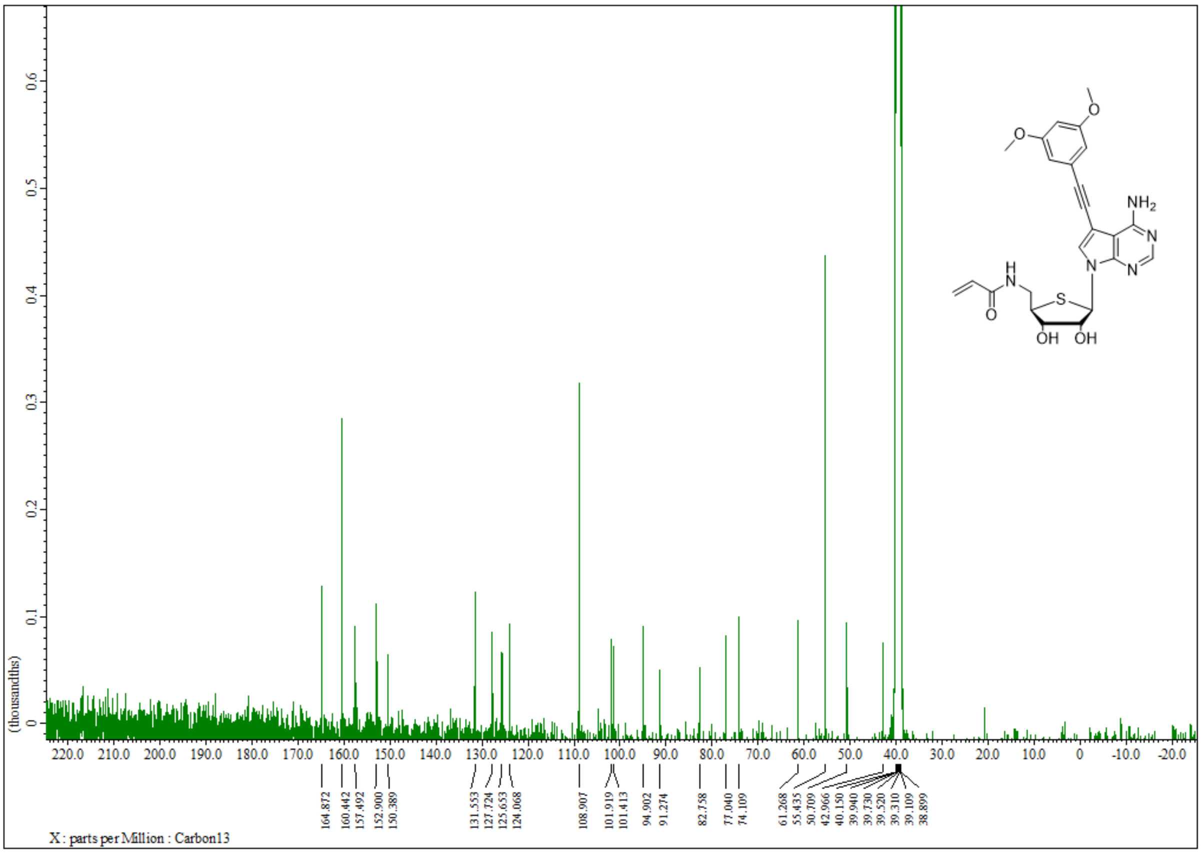

Figure S25. ^{1}H-NMR (DMSO-d_6, 400 MHz) and ^{13}C-NMR (DMSO-d_6, 100 MHz) spectra of compound **19a**

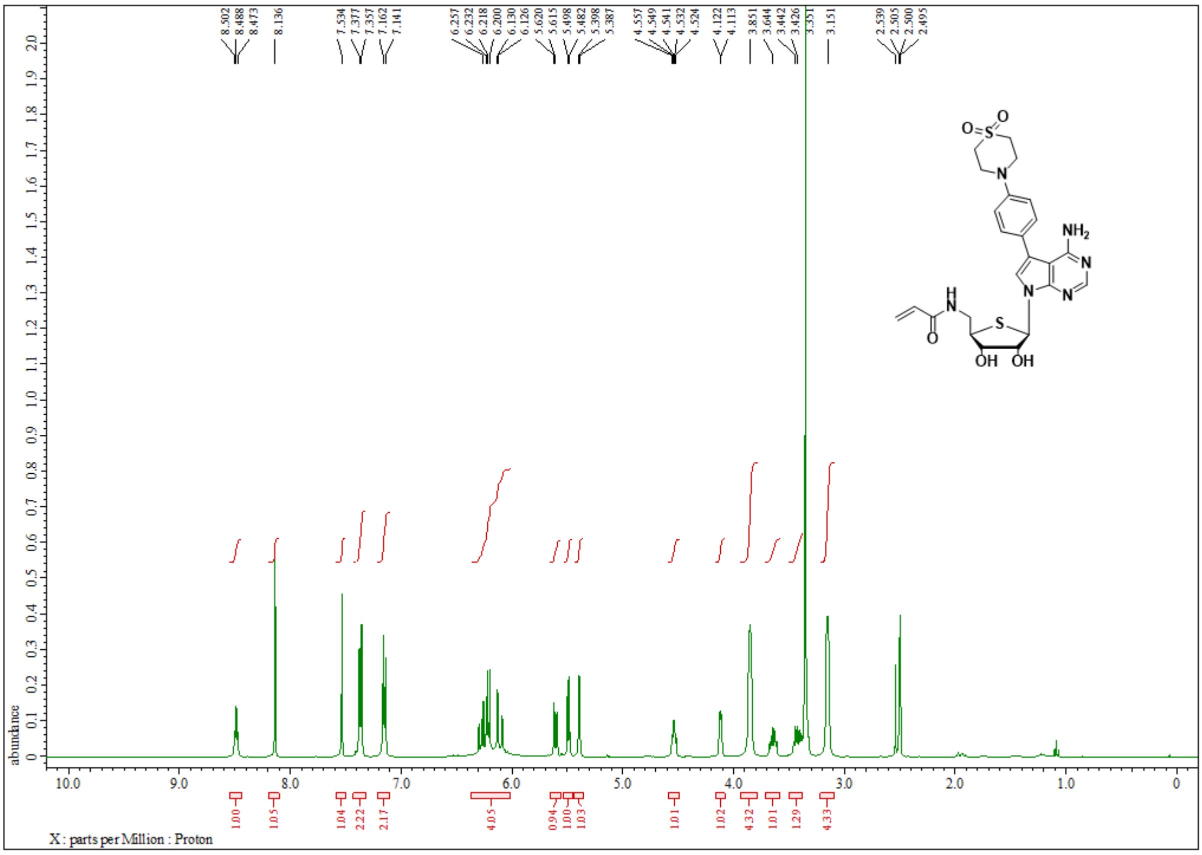

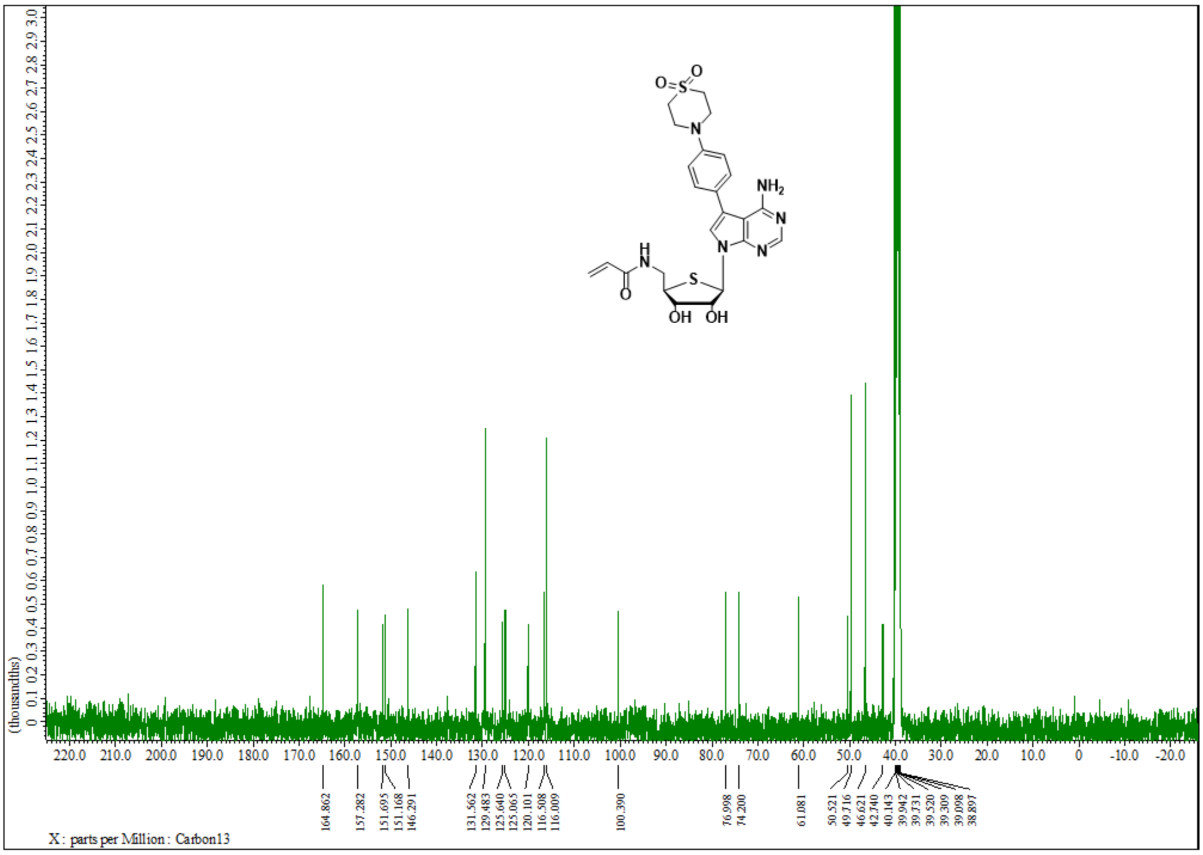

Figure S26. ^{1}H-NMR (DMSO-d_6, 400 MHz) and ^{13}C-NMR (DMSO-d_6, 100 MHz) spectra of compound **19b**

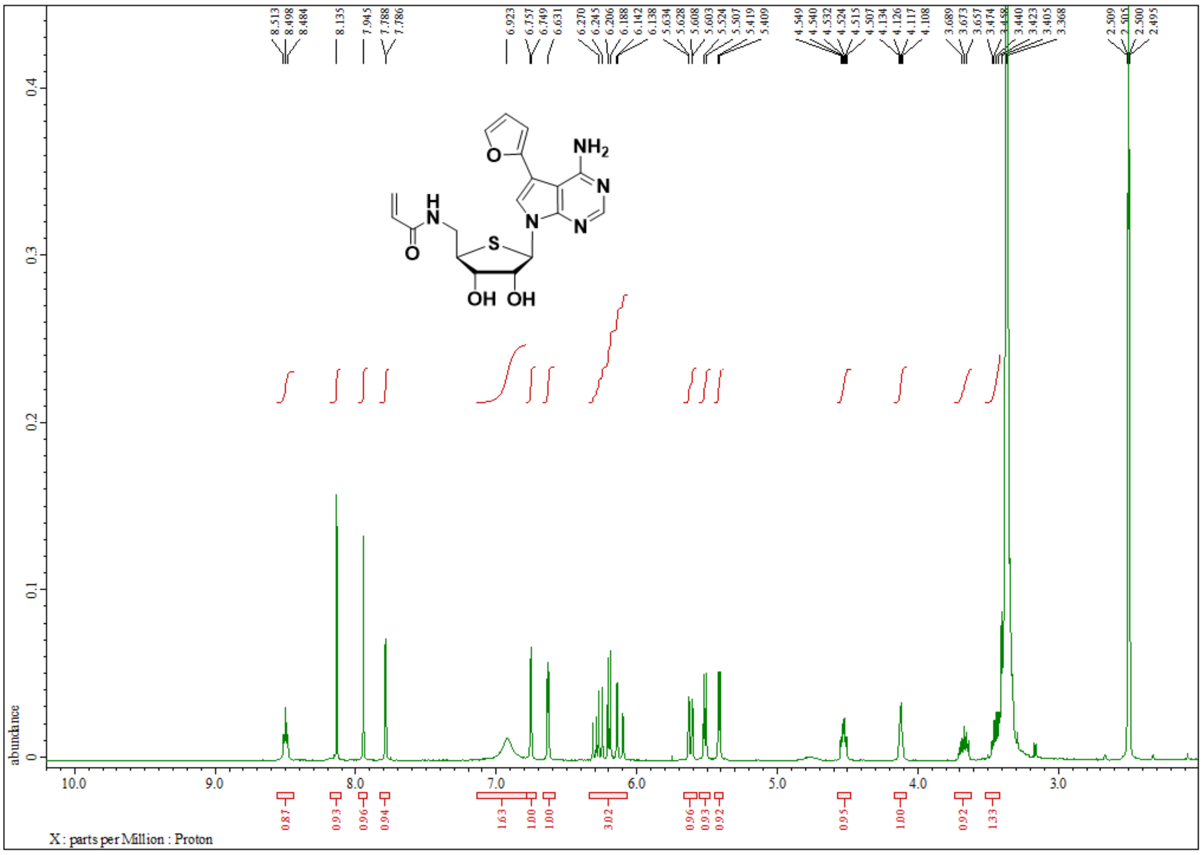

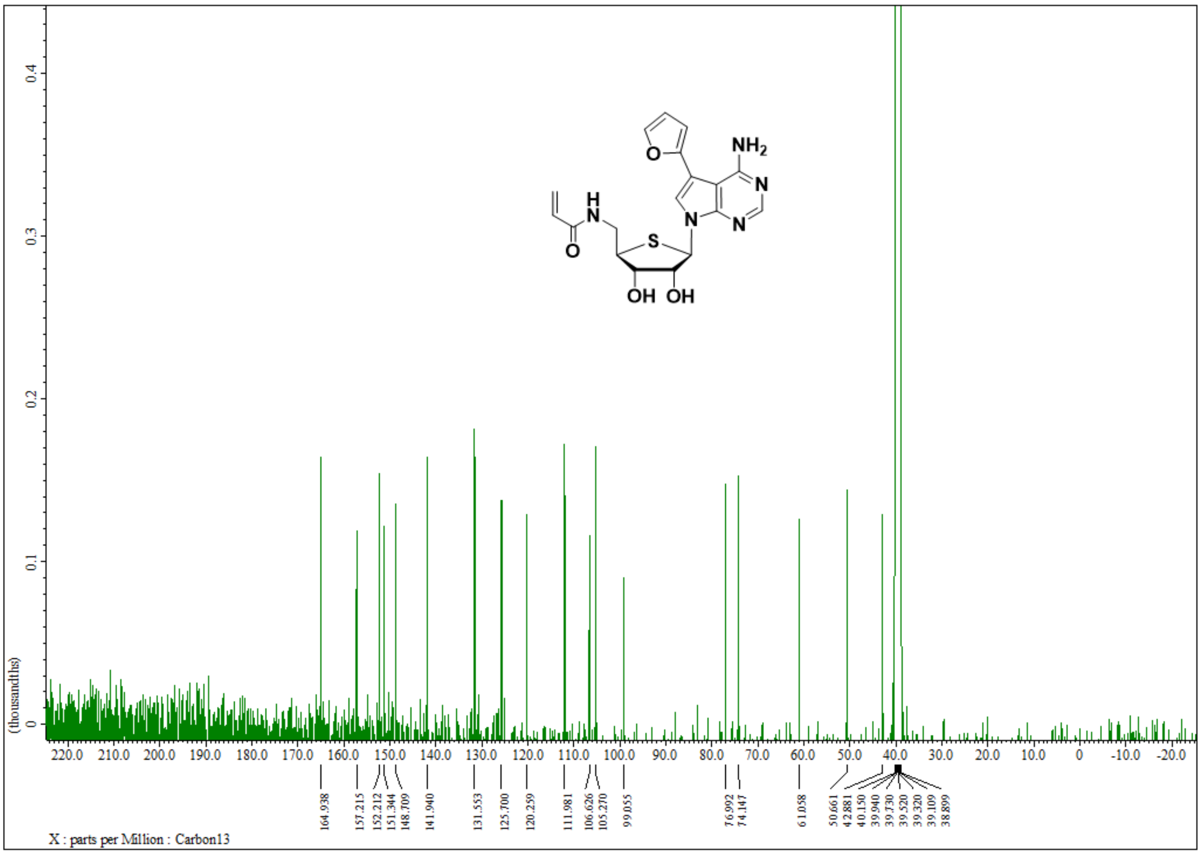

Figure S27. ^{1}H-NMR (DMSO-d_6, 400 MHz) and ^{13}C-NMR (DMSO-d_6, 100 MHz) spectra of compound **19c**

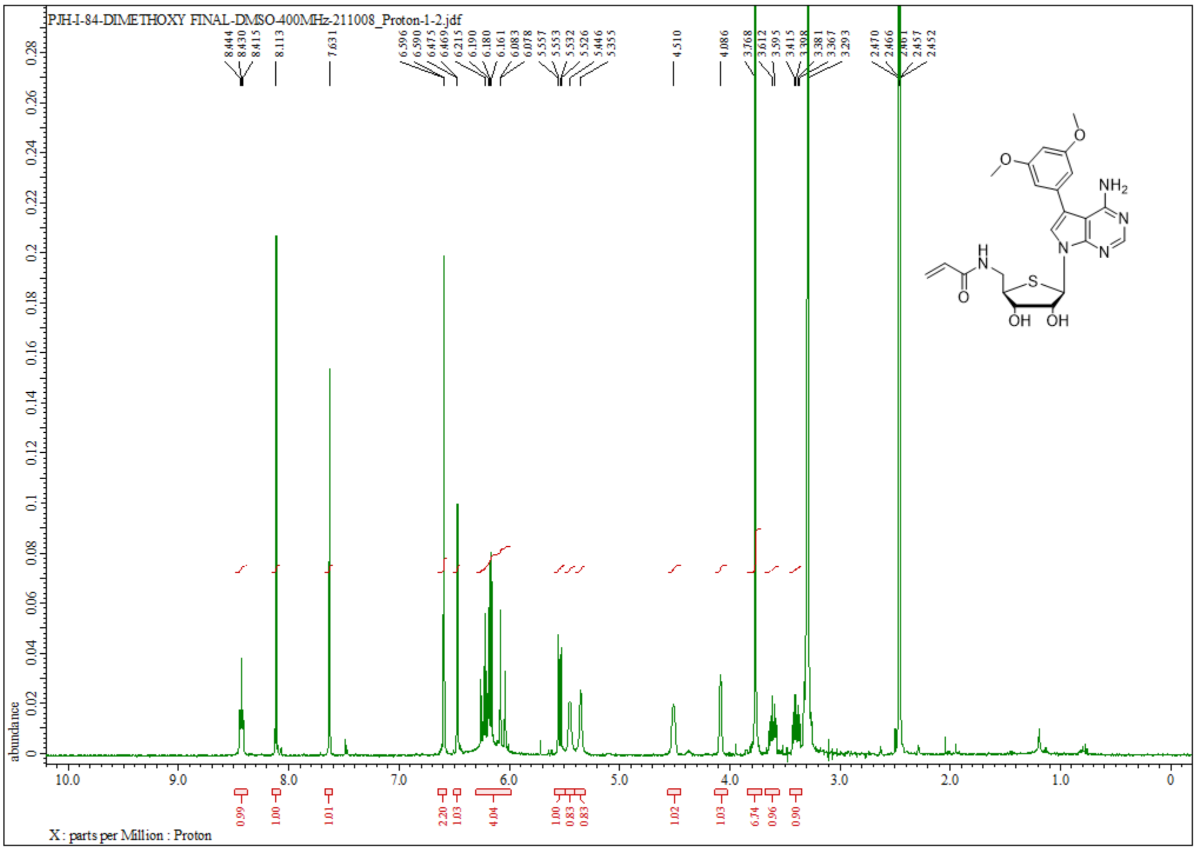

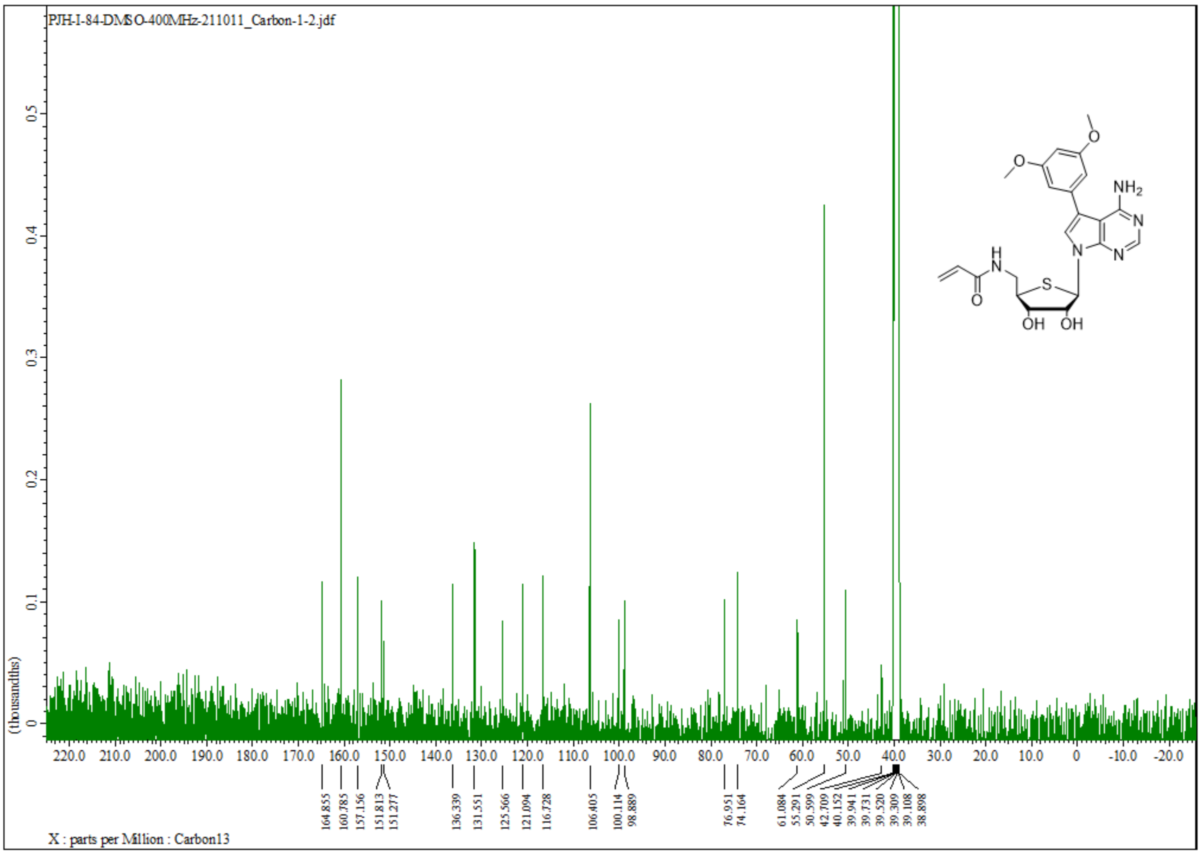

Figure S28. ^{1}H-NMR (DMSO-d_6, 400 MHz) and ^{13}C-NMR (DMSO-d_6, 100 MHz) spectra of compound **19d**

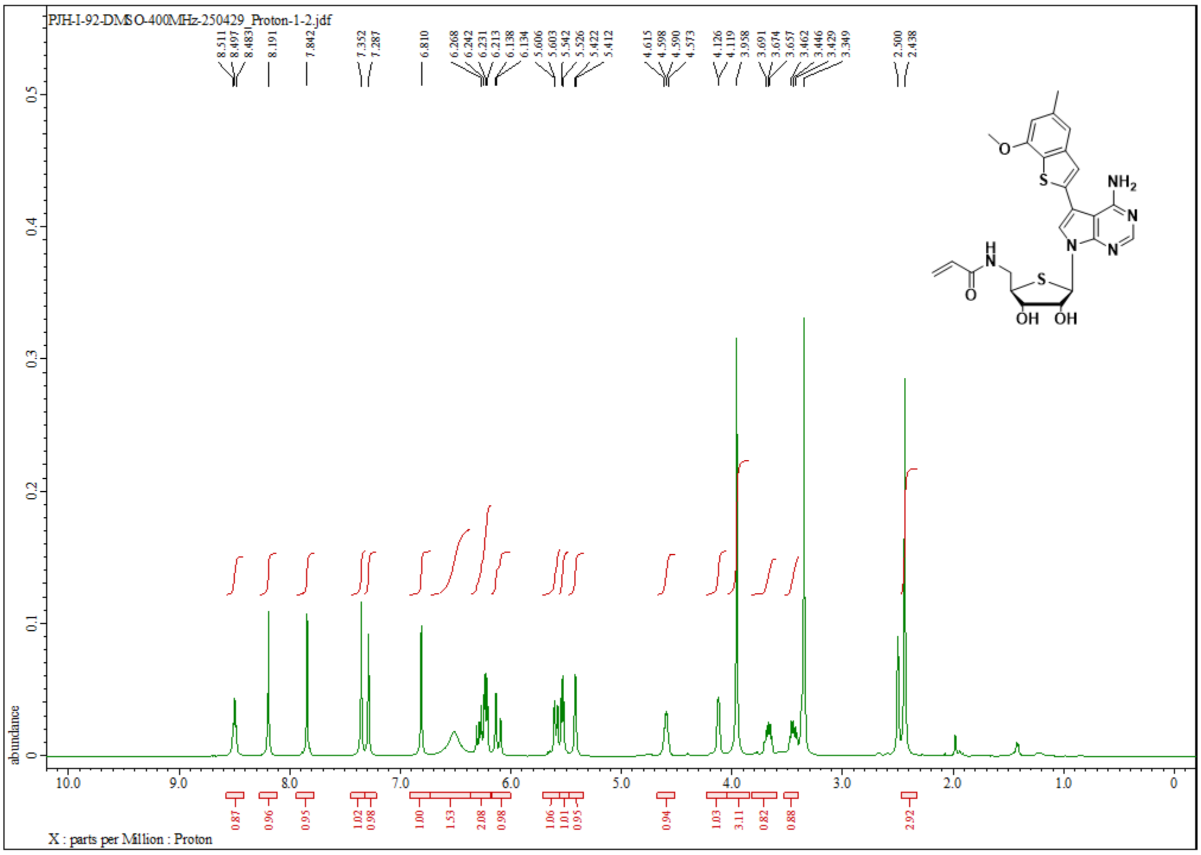

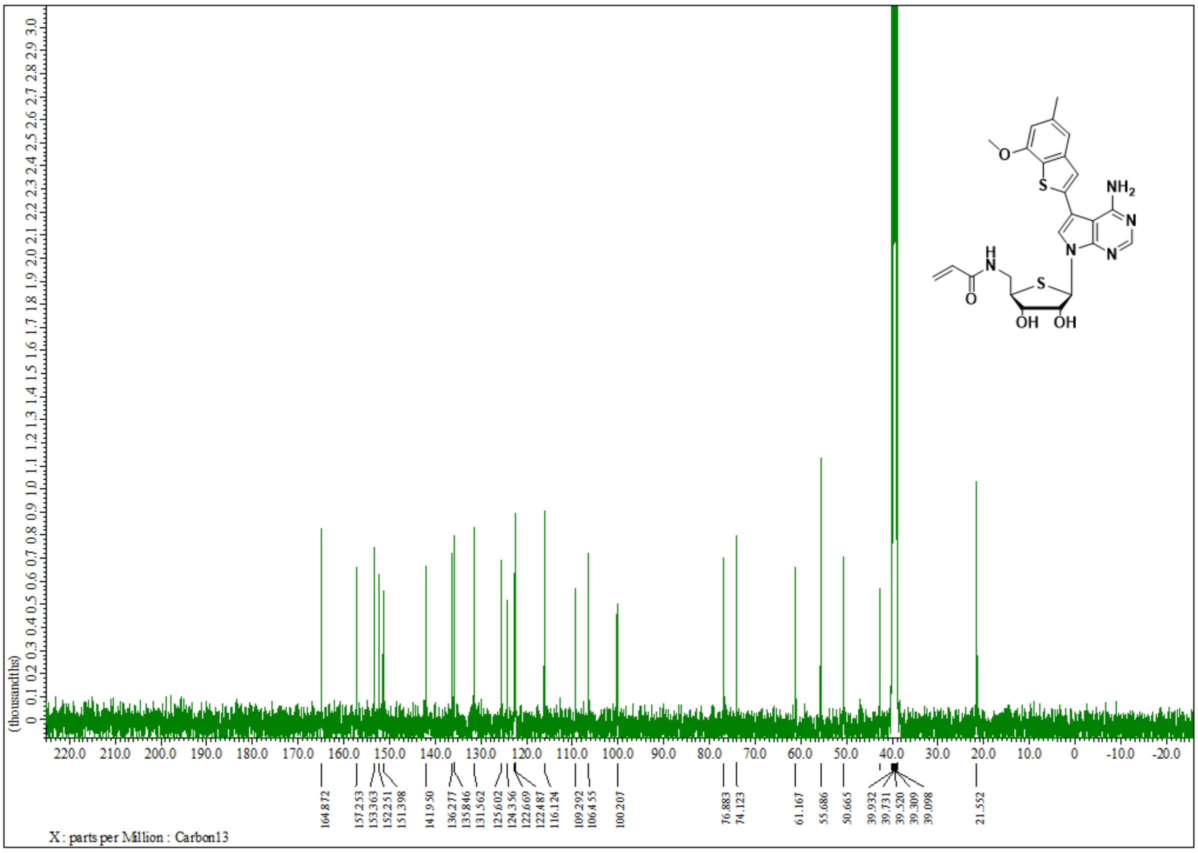

Figure S29. ^{1}H-NMR (DMSO-d_6, 400 MHz) and ^{13}C-NMR (DMSO-d_6, 100 MHz) spectra of compound **19e**

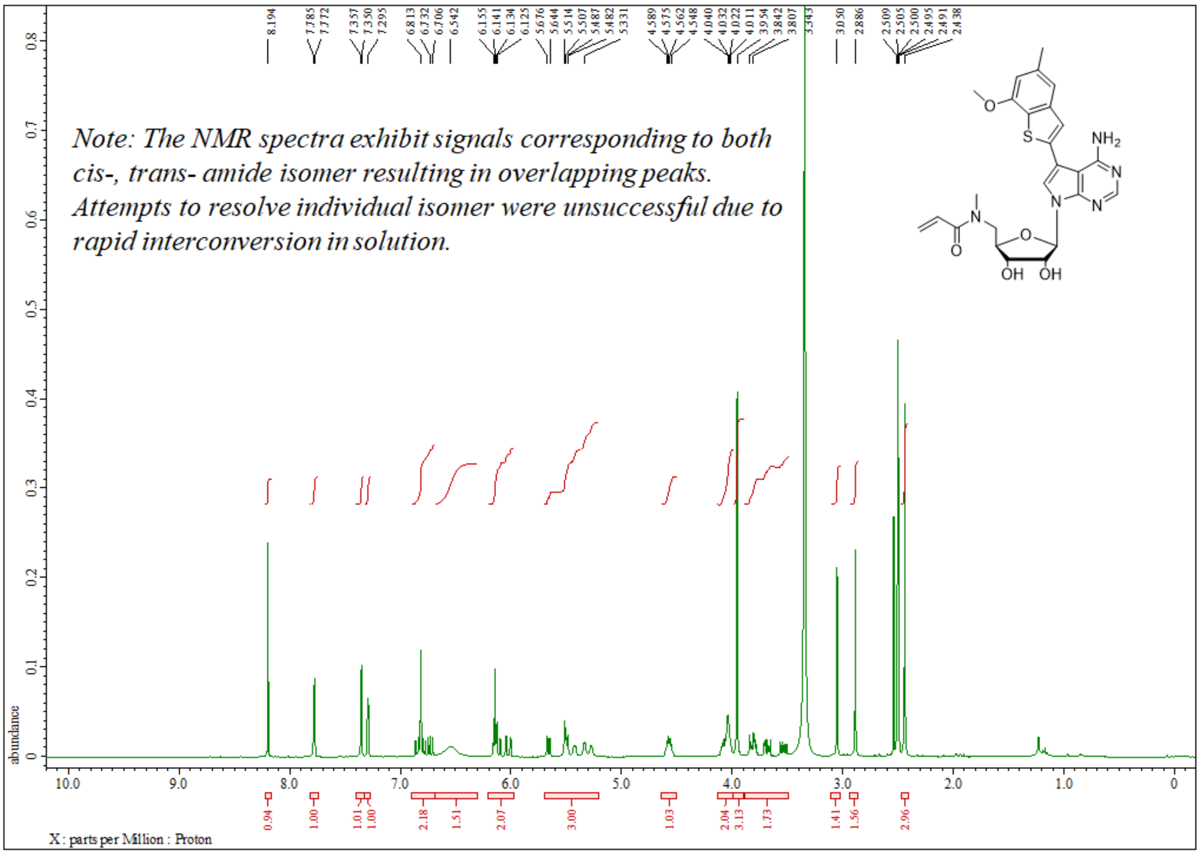

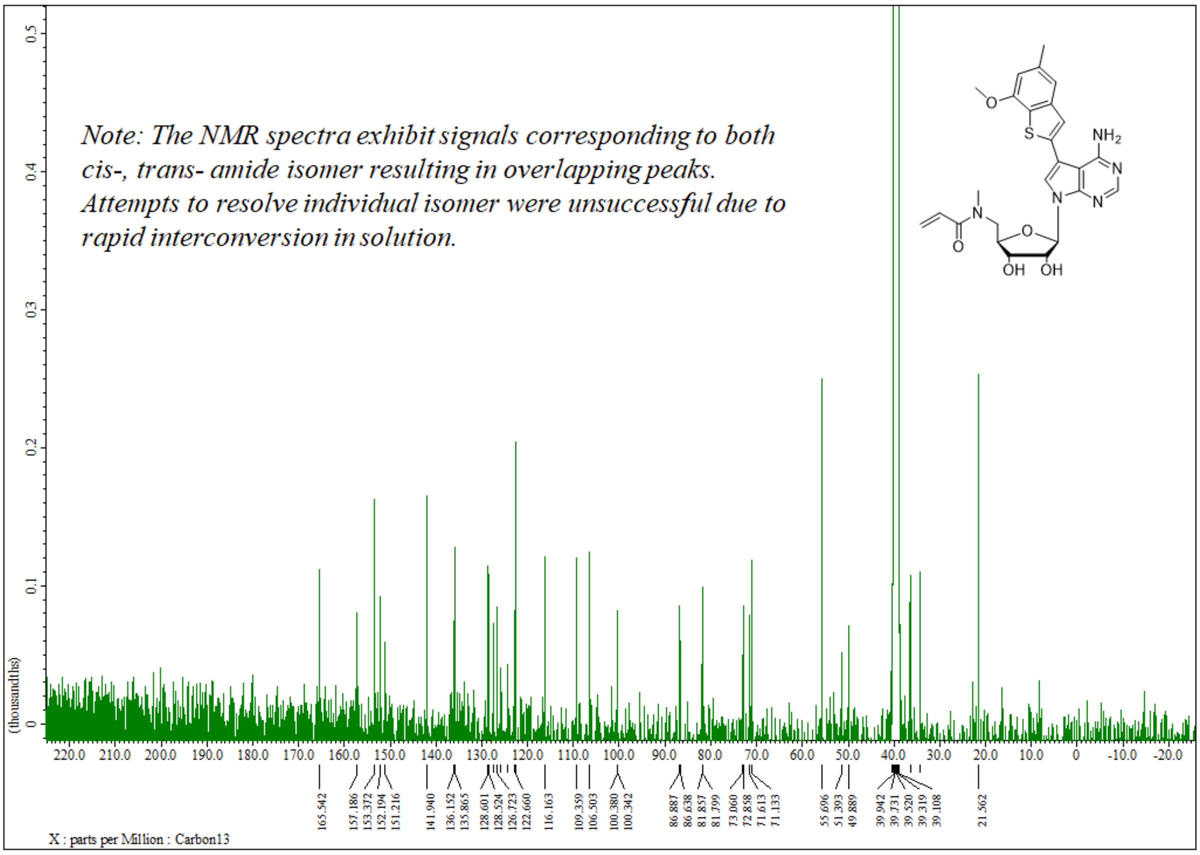

Figure S30. ^{1}H-NMR (DMSO-d_6, 400 MHz) and ^{13}C-NMR (DMSO-d_6, 100 MHz) spectra of compound **21**

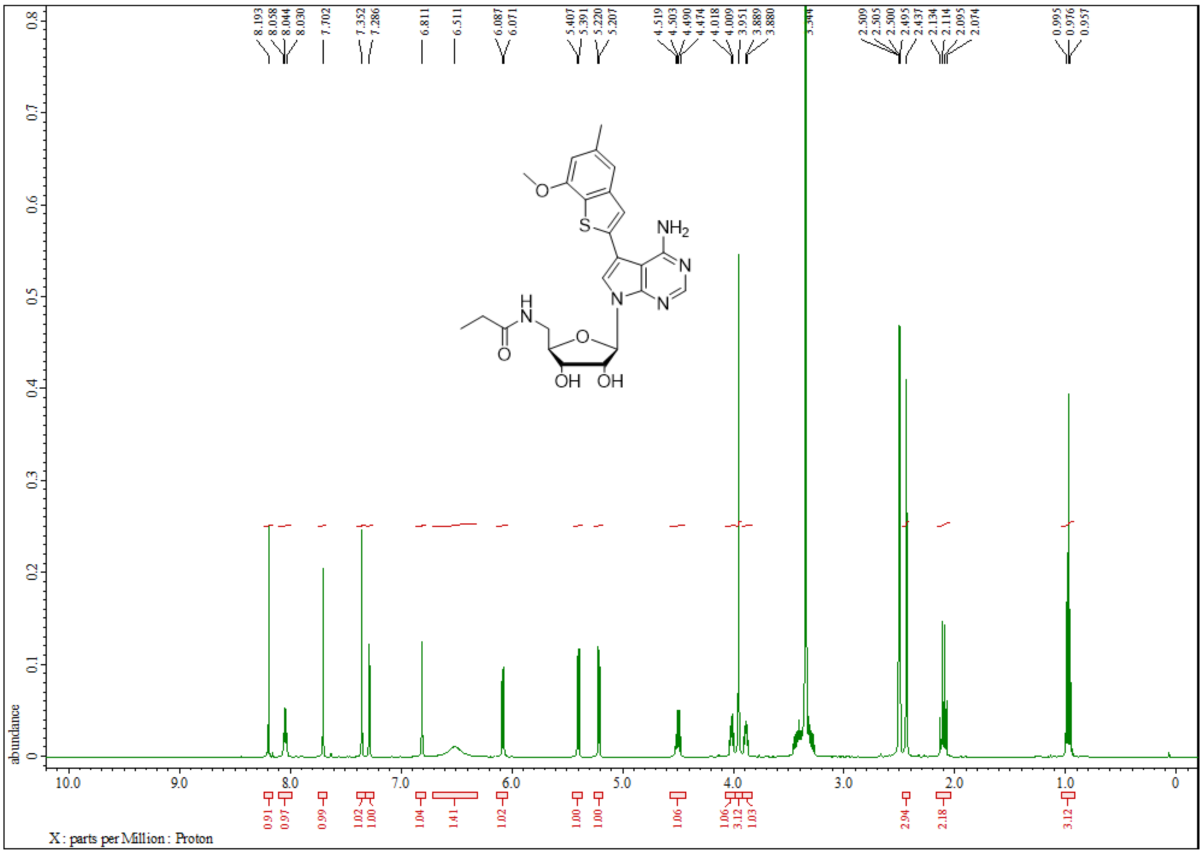

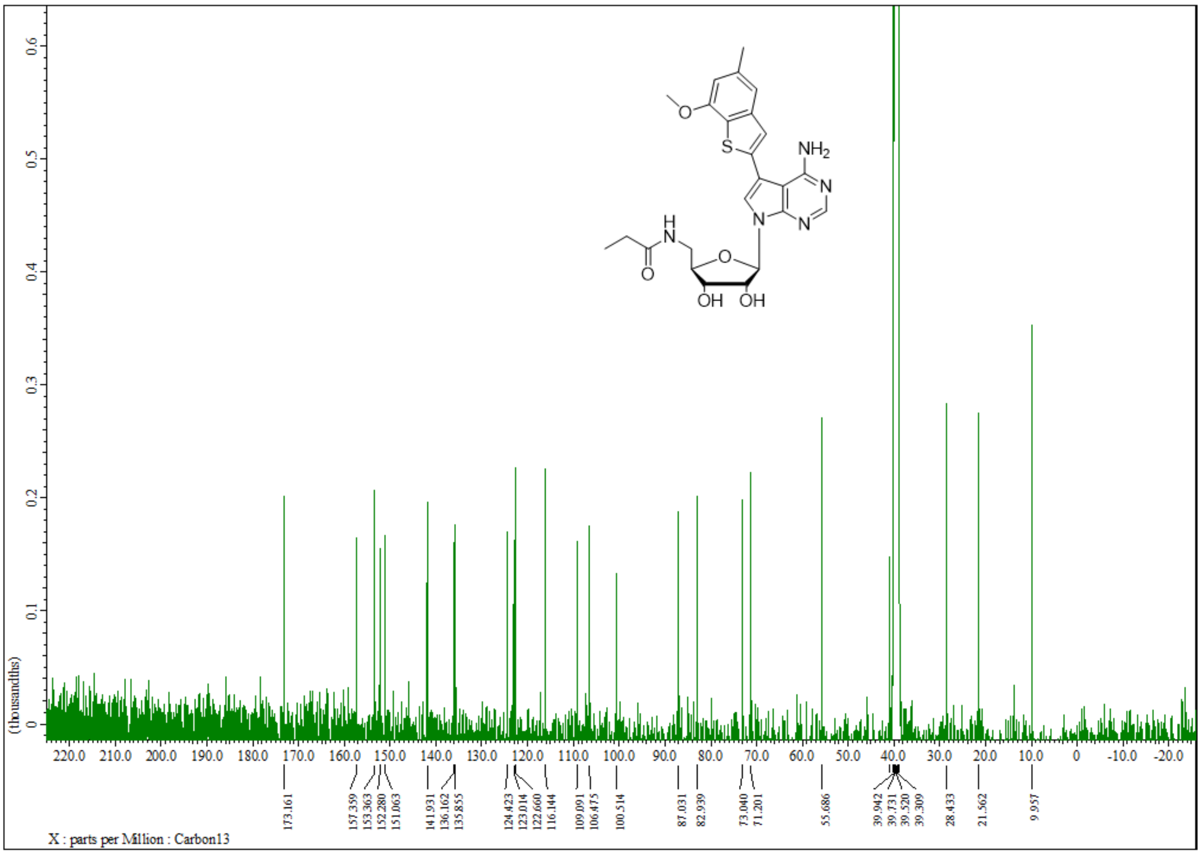

Figure S31. ^{1}H-NMR (DMSO-d_6, 400 MHz) and ^{13}C-NMR (DMSO-d_6, 100 MHz) spectra of compound **22a**

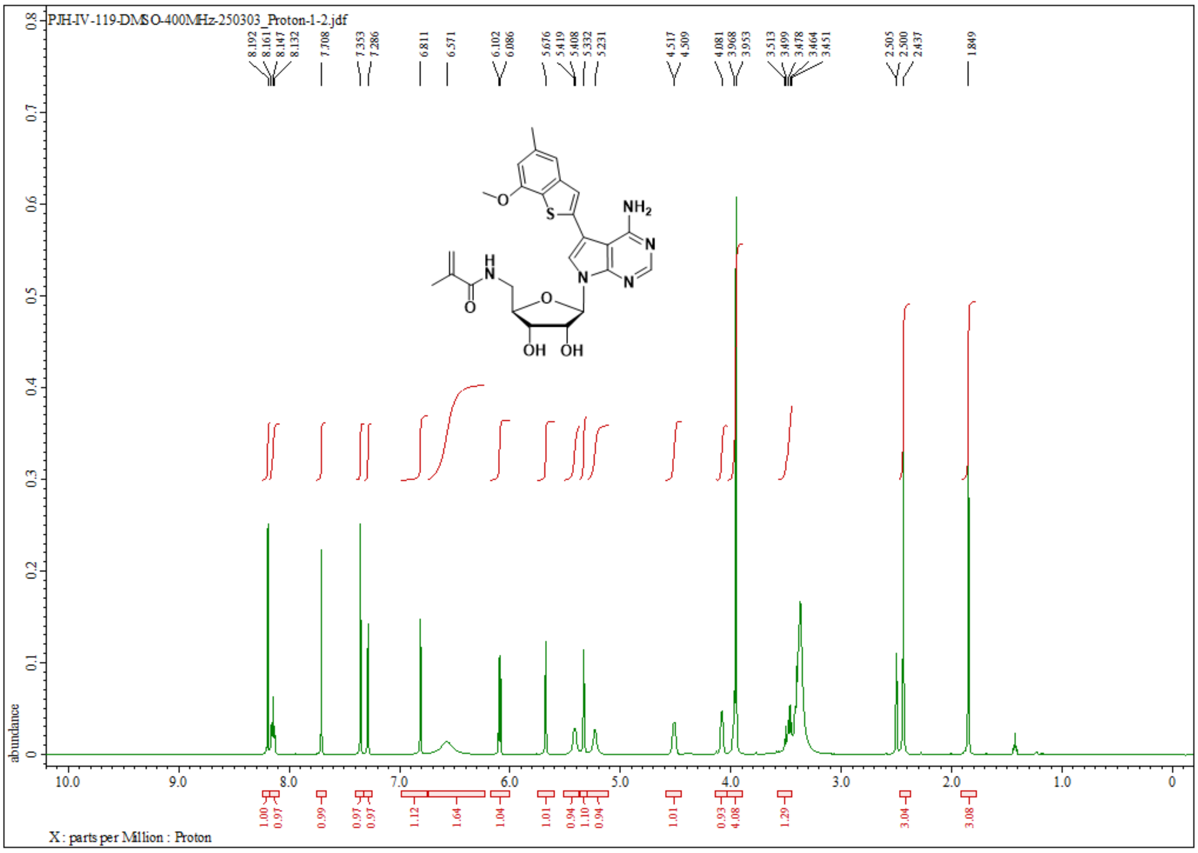
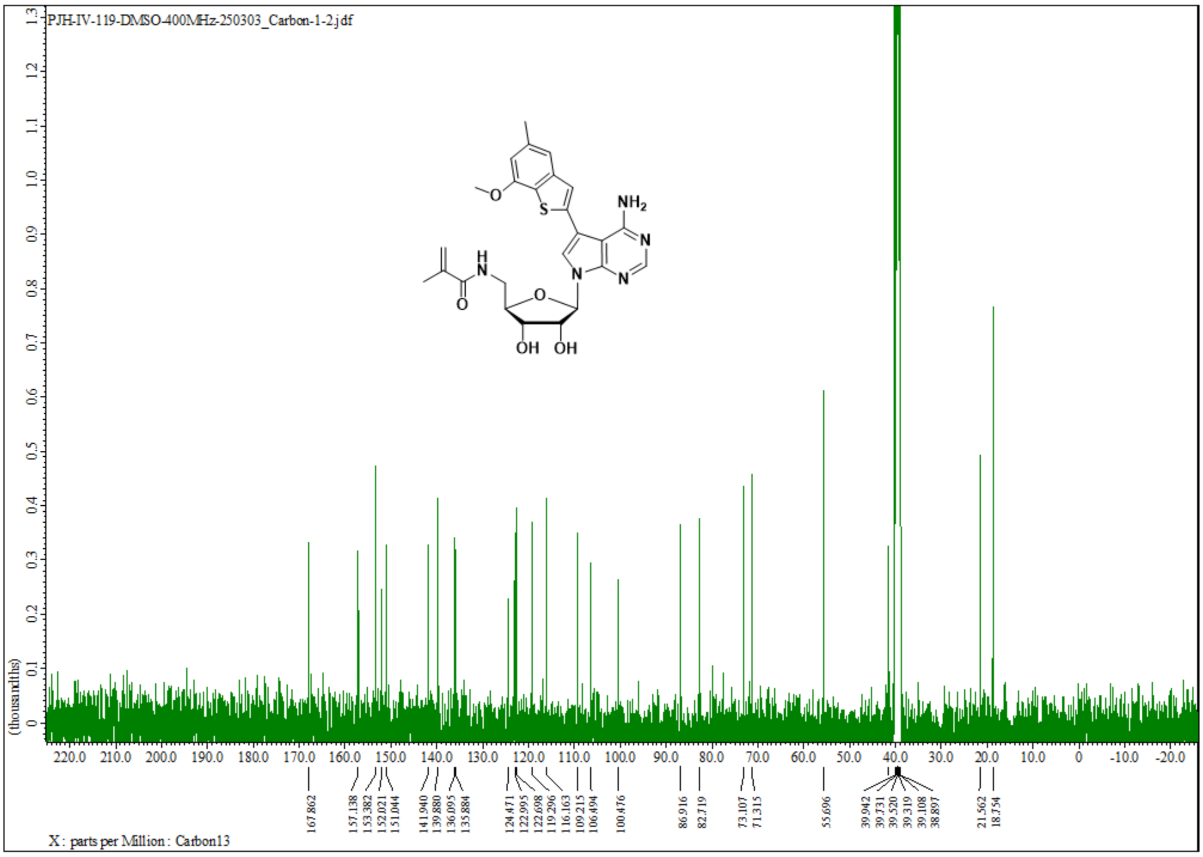

Figure S32. ^{1}H-NMR (DMSO-d_6, 400 MHz) and ^{13}C-NMR (DMSO-d_6, 100 MHz) spectra of compound **22b**

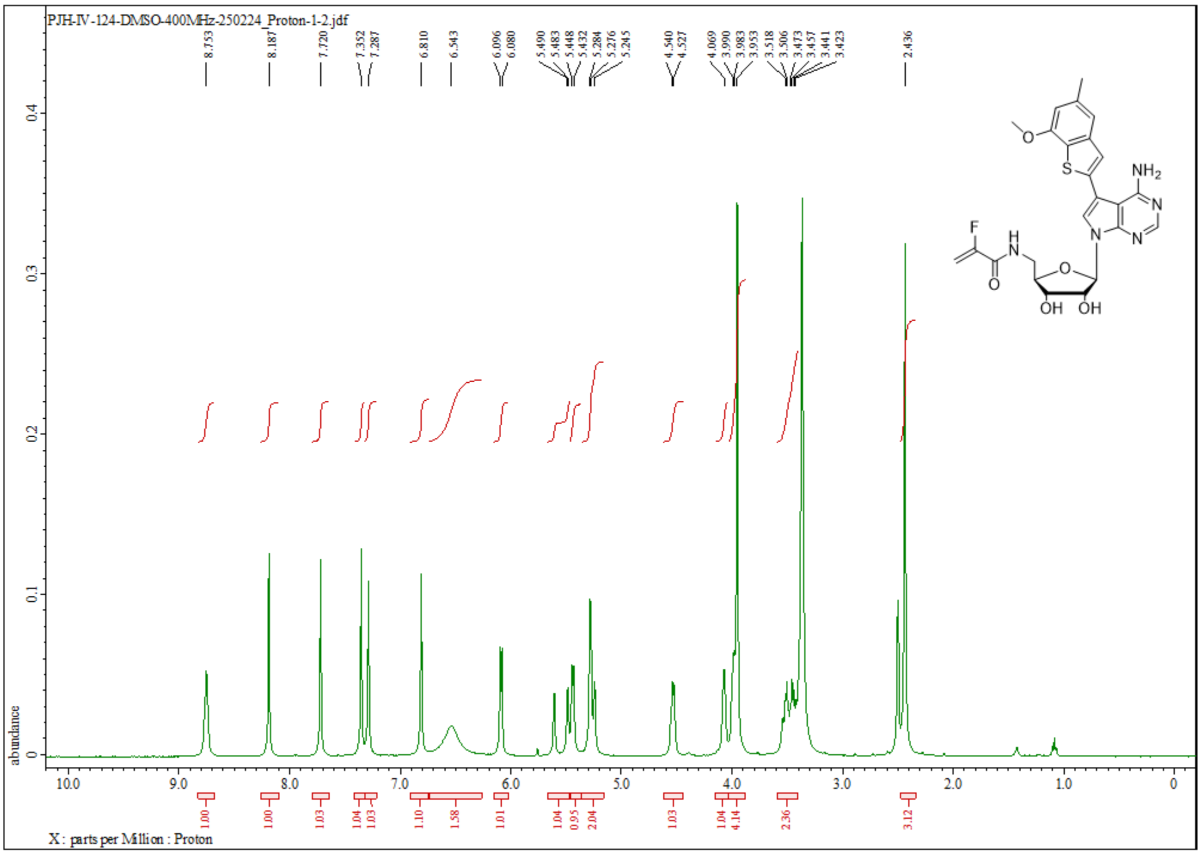
PJH-IV-124-DMSO-400MHz-250224_Proton-1-2.jdf
X : parts per Million : Proton
abundance

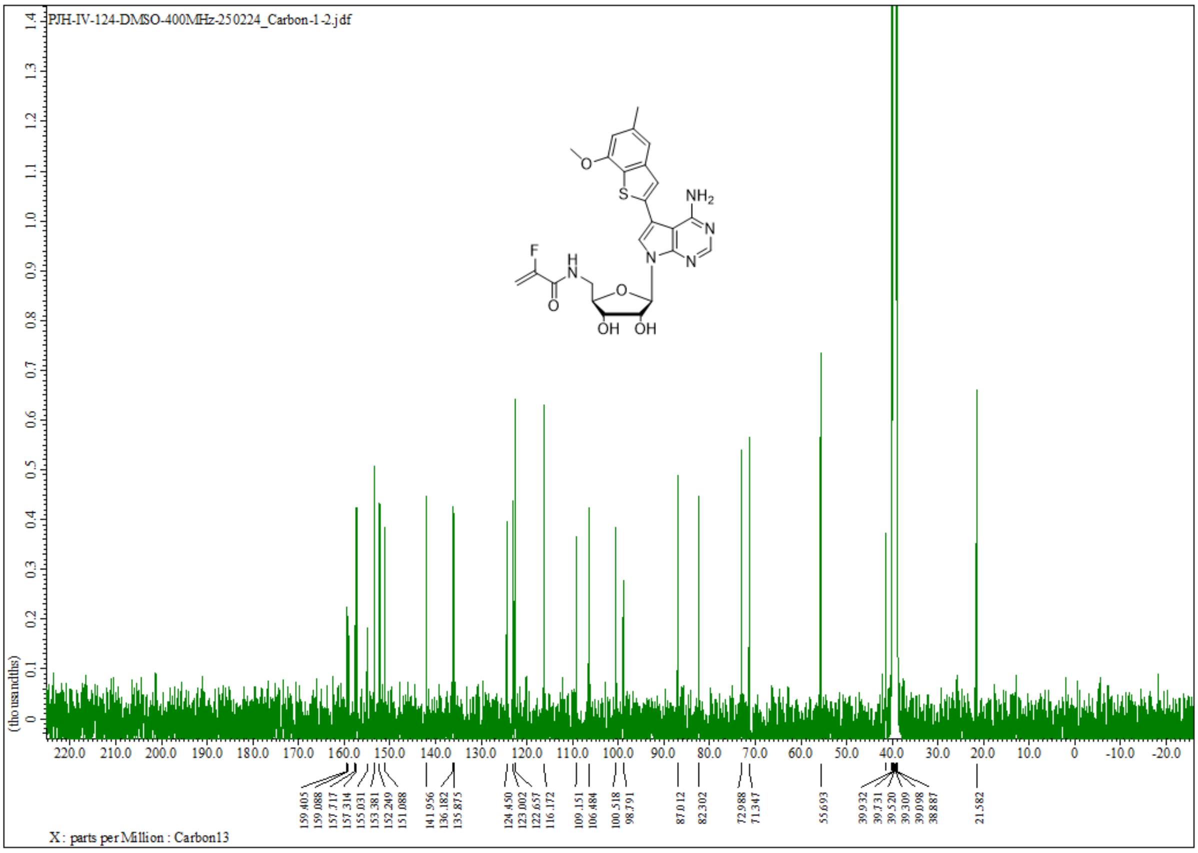
PJH-IV-124-DMSO-400MHz-250224_Carbon-1-2.jdf
X : parts per Million : Carbon13
(thousandths)

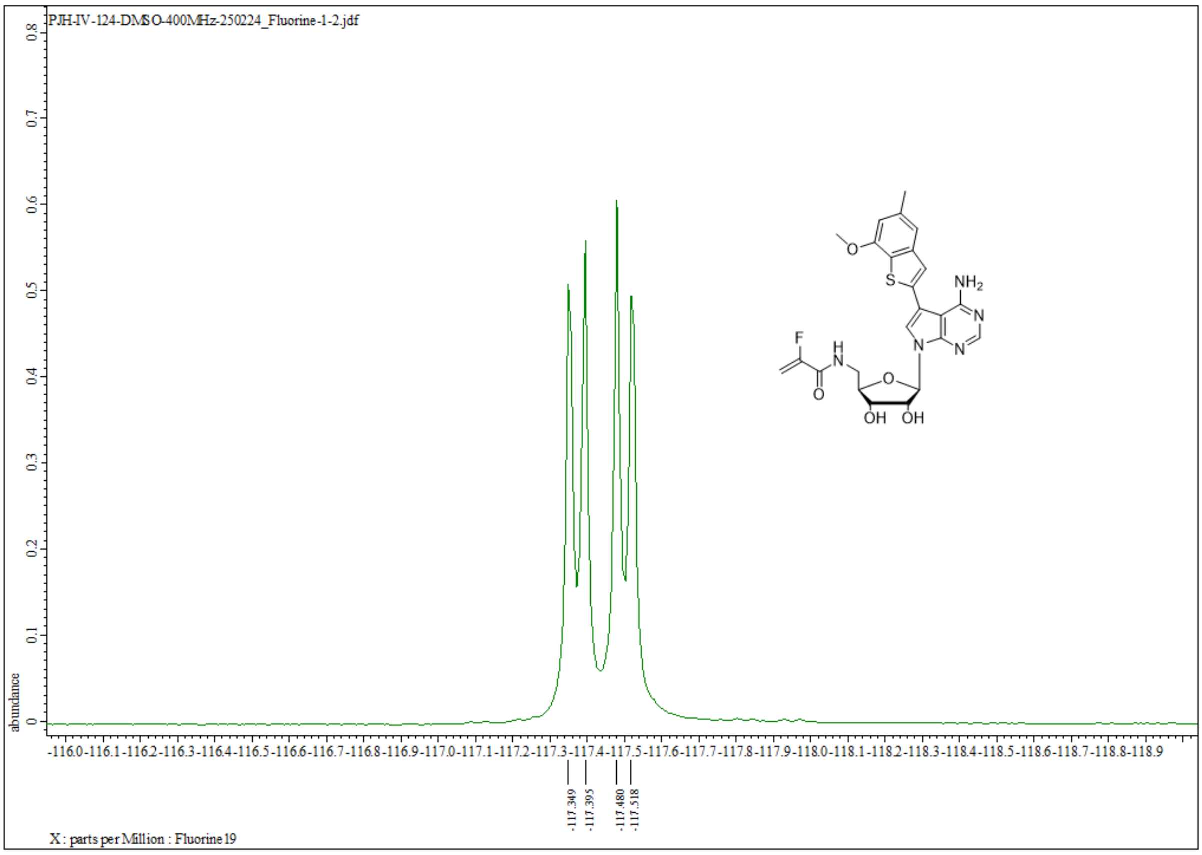

Figure S33. ^{1}H-NMR (DMSO-d_6, 400 MHz), ^{13}C-NMR (DMSO-d_6, 100 MHz) and 19F-NMR (DMSO-d_6, 375 MHz) spectra of compound **22c**

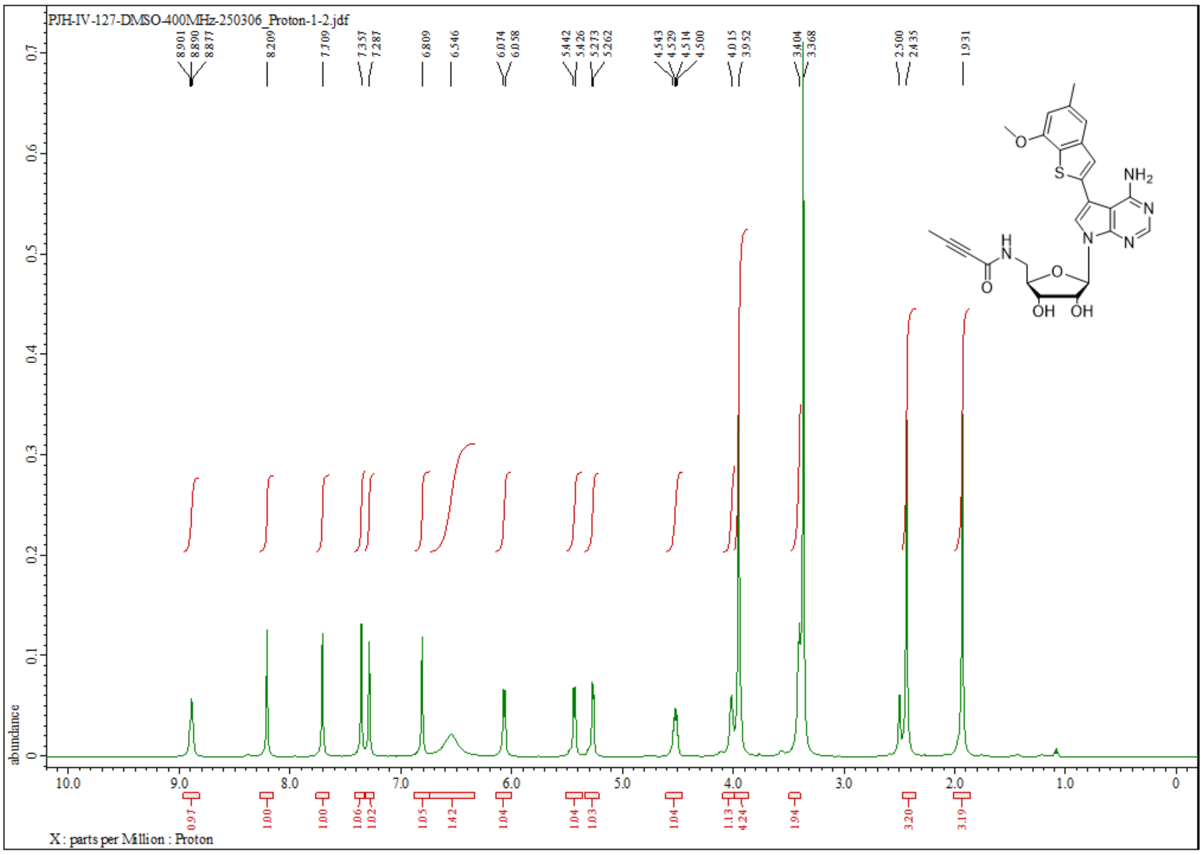

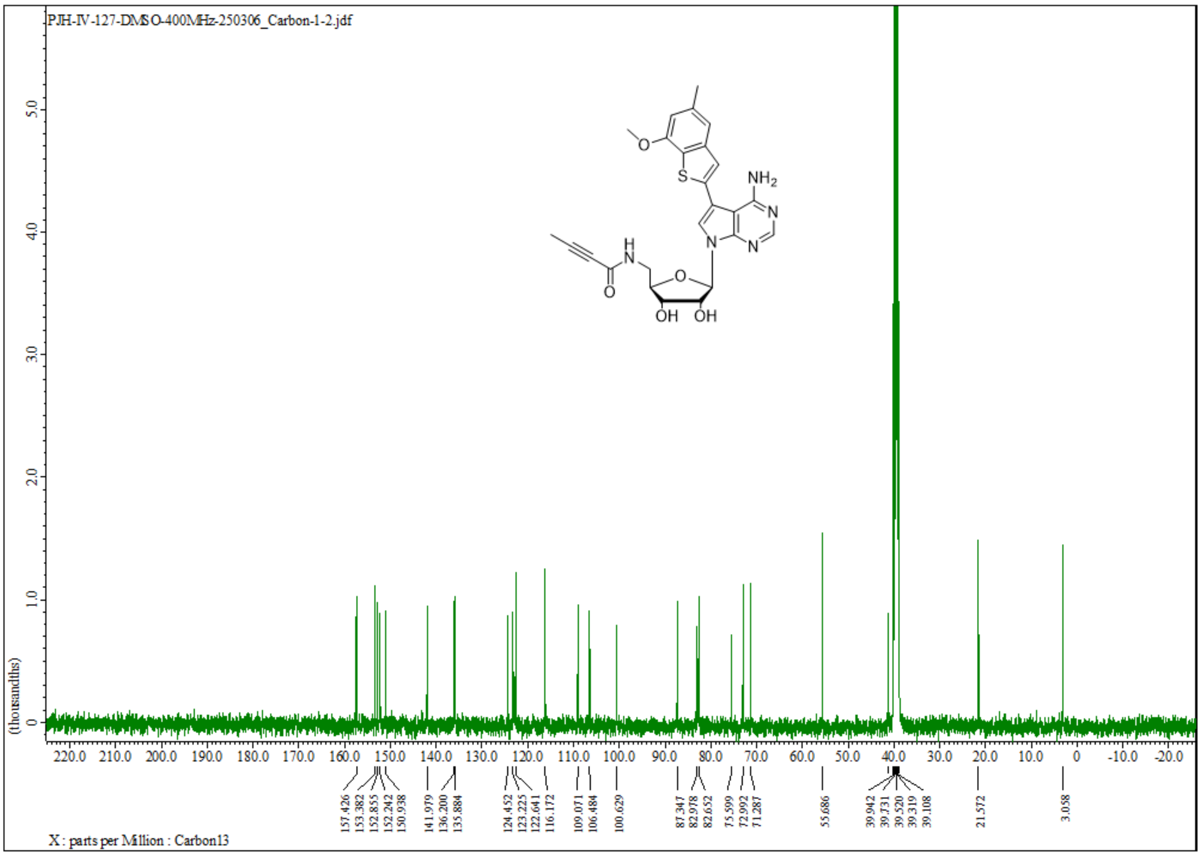

Figure S34. ^{1}H-NMR (DMSO-d_6, 400 MHz) and ^{13}C-NMR (DMSO-d_6, 100 MHz) spectra of compound **22d**

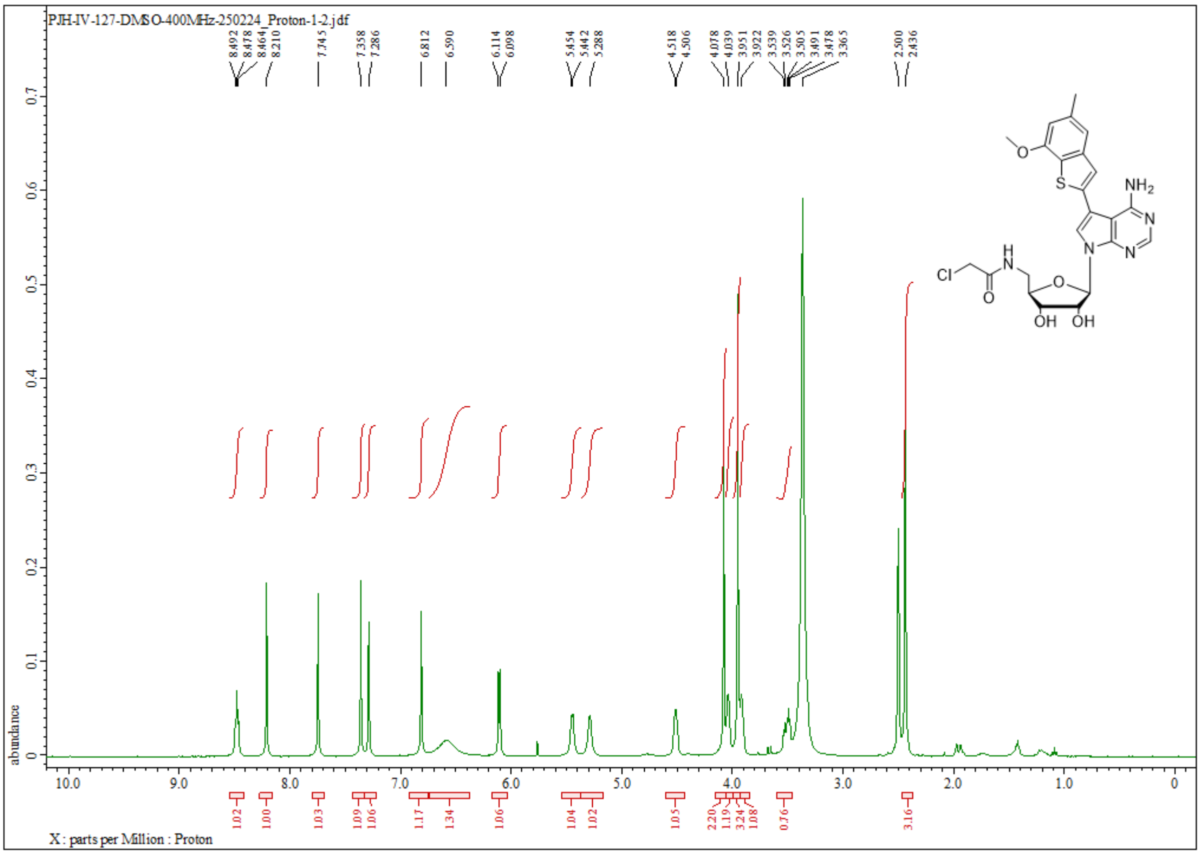

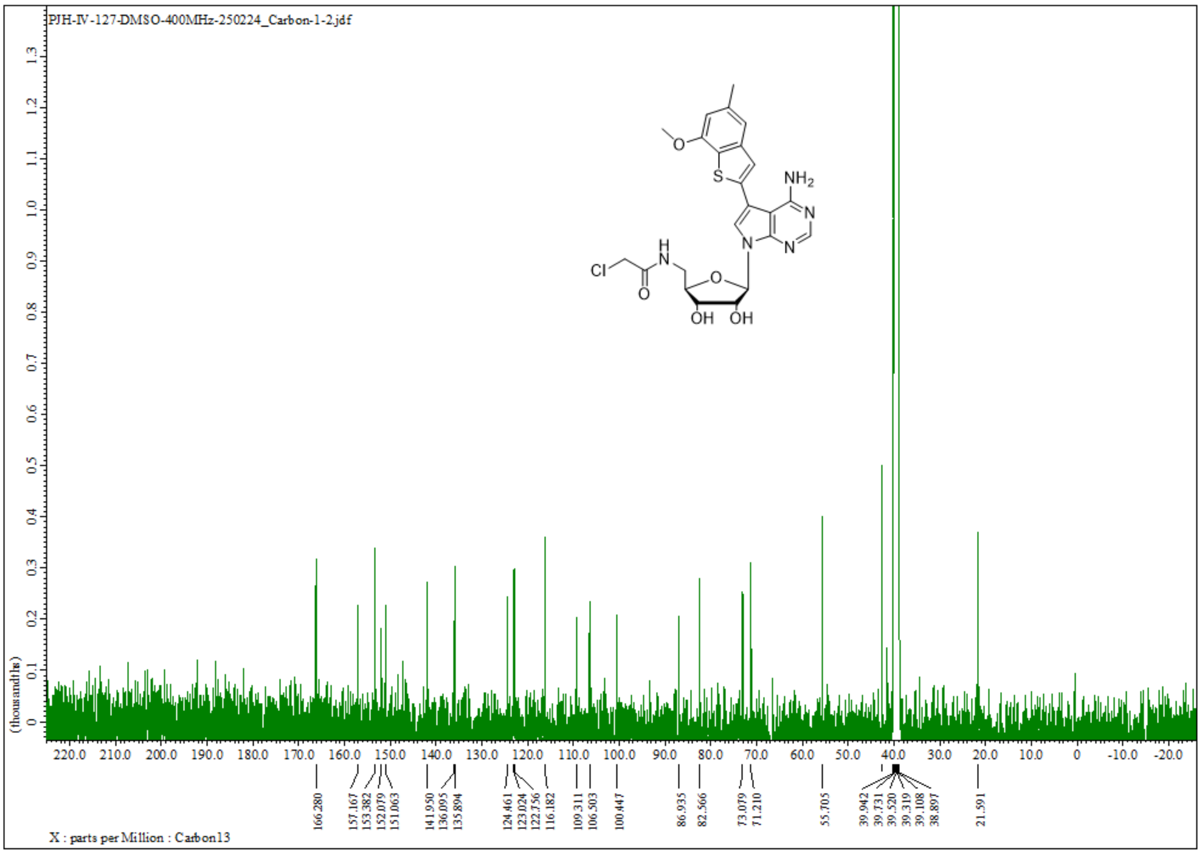

Figure S35. ^{1}H-NMR (DMSO-d_6, 400 MHz) and ^{13}C-NMR (DMSO-d_6, 100 MHz) spectra of compound **22e**

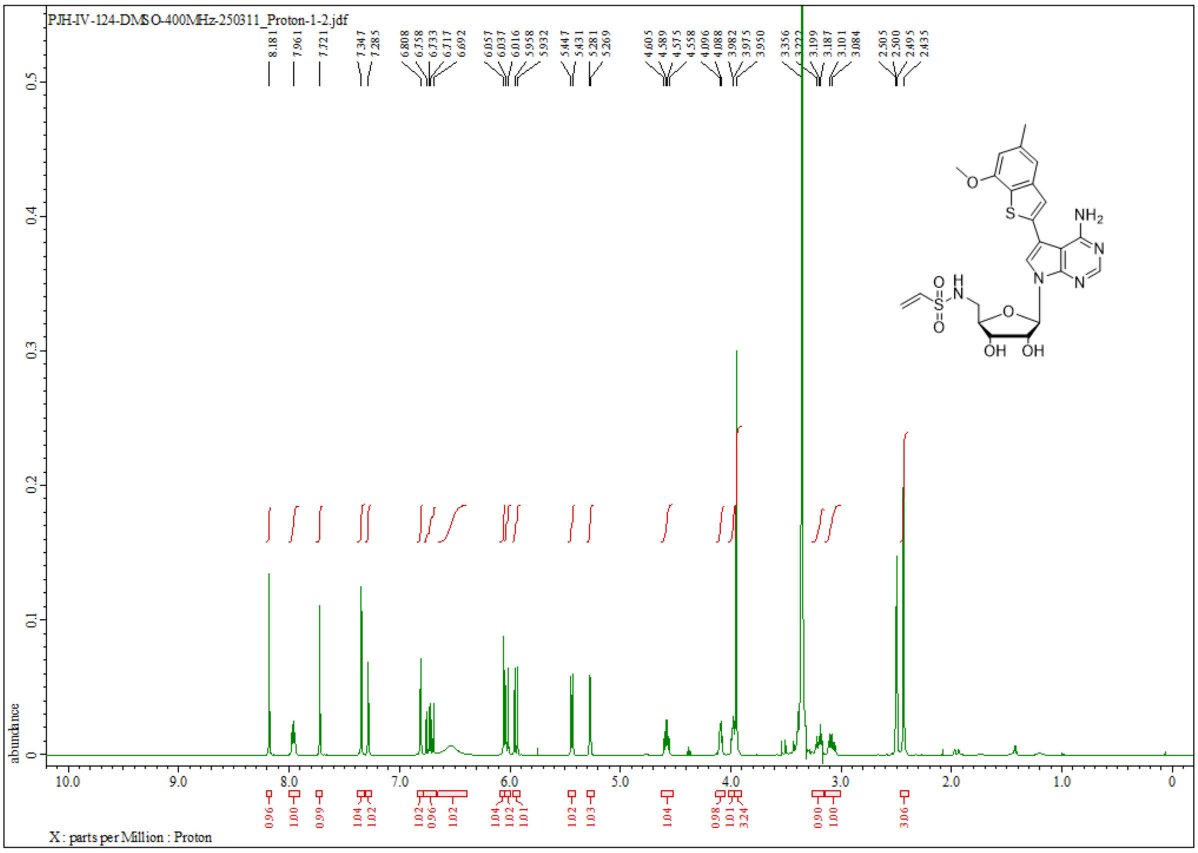

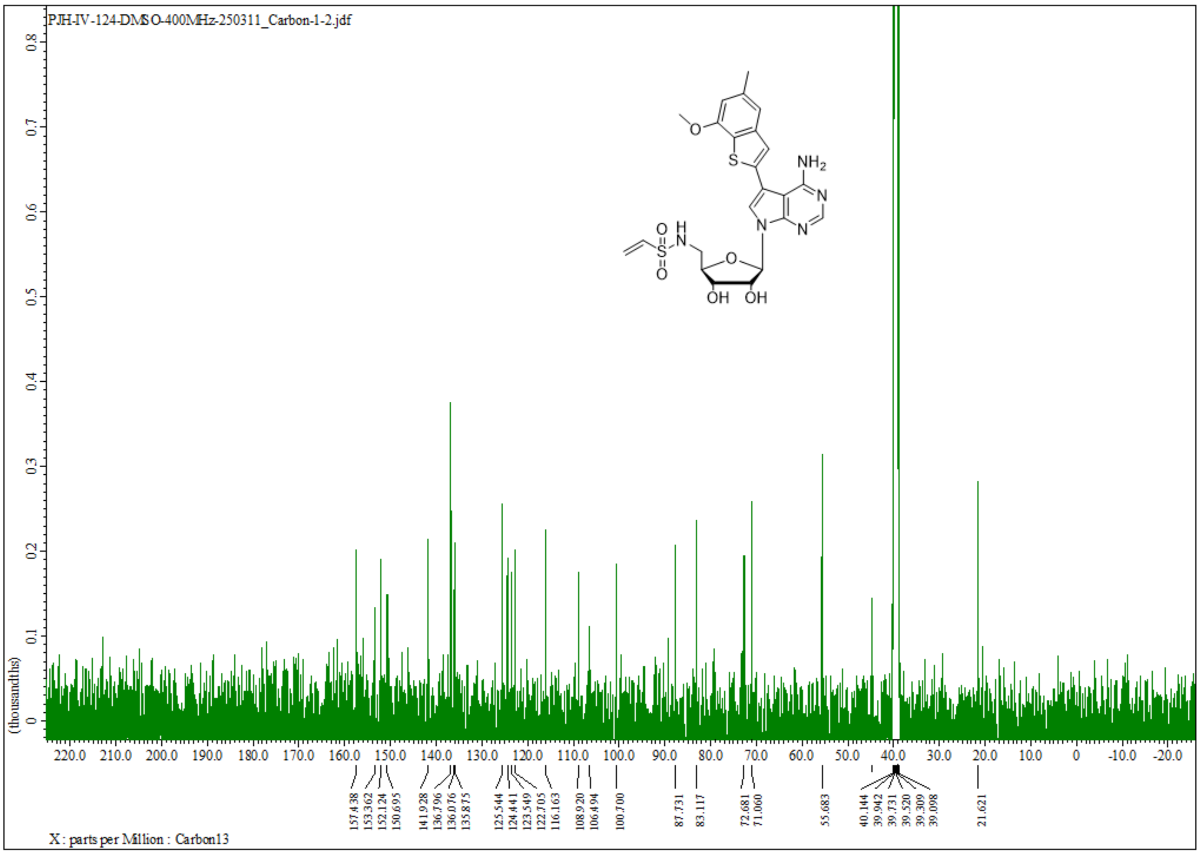

Figure S36. ^{1}H-NMR (DMSO-d_6, 400 MHz) and ^{13}C-NMR (DMSO-d_6, 100 MHz) spectra of compound **22f**

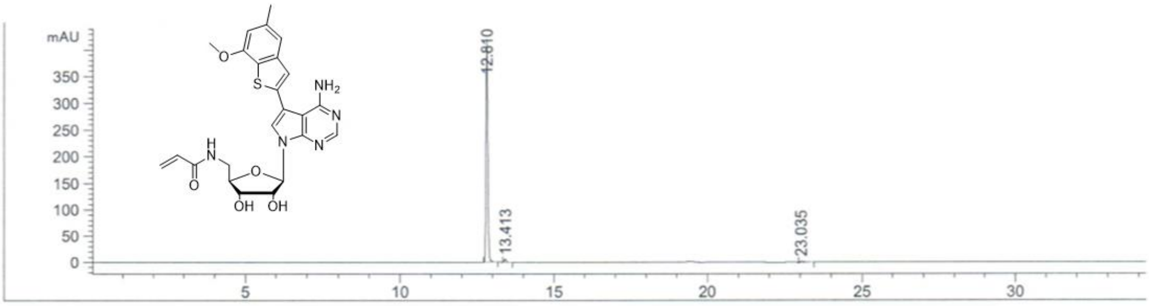

Signal 2: DAD1 B, Sig=297,4 Ref=off

Peak #	RetTime [min]	Type	Width [min]	Area [mAU*s]	Height [mAU]	Area %
1	12.810	BB	0.0644	1749.16431	421.93262	96.9056
2	13.413	BB	0.0608	24.49244	6.11001	1.3569
3	23.035	BB	0.1258	31.36182	3.45945	1.7375
Totals :				1805.01857	431.50207	

Figure S37. HPLC spectra of compound **13f**

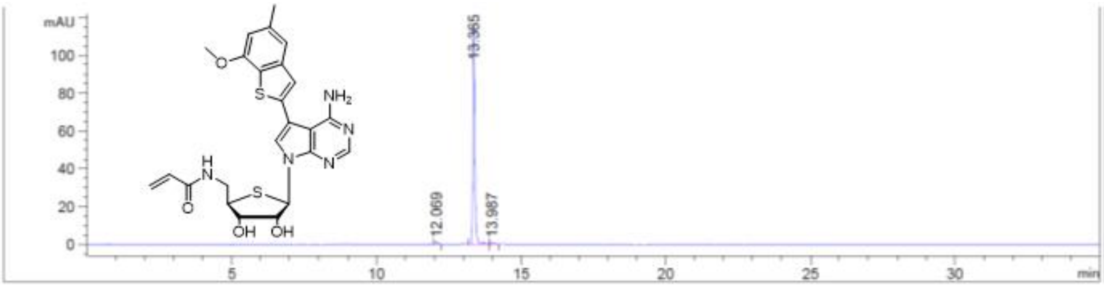

Signal 2: DAD1 B, Sig=297,4 Ref=off

Peak #	RetTime [min]	Type	Width [min]	Area [mAU*s]	Height [mAU]	Area %
1	12.069	BB	0.0770	8.19520	1.56670	1.4448
2	13.365	BV R	0.0734	550.99164	116.07513	97.1382
3	13.987	VB	0.0906	8.03777	1.25697	1.4170
Totals :				567.22461	118.89880	

Figure S38. HPLC spectra of compound **19e**

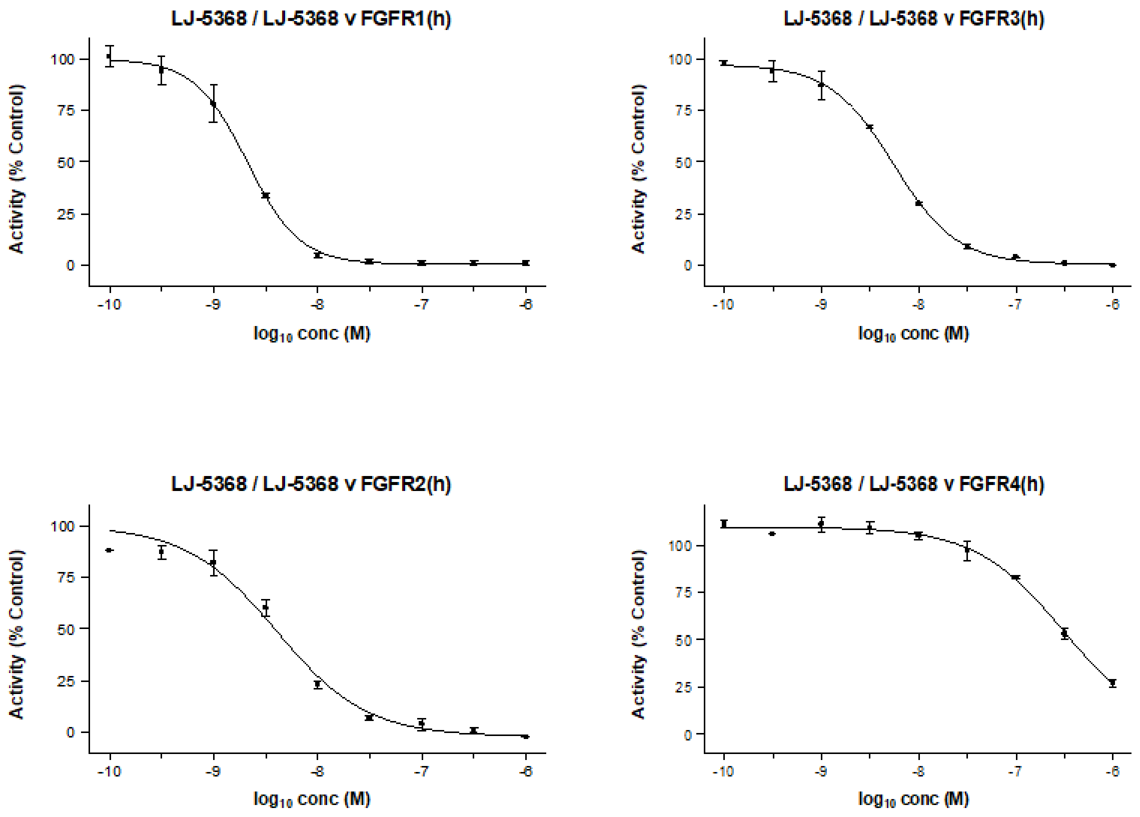

Figure S39. IC_{50} curves of compound **6h**

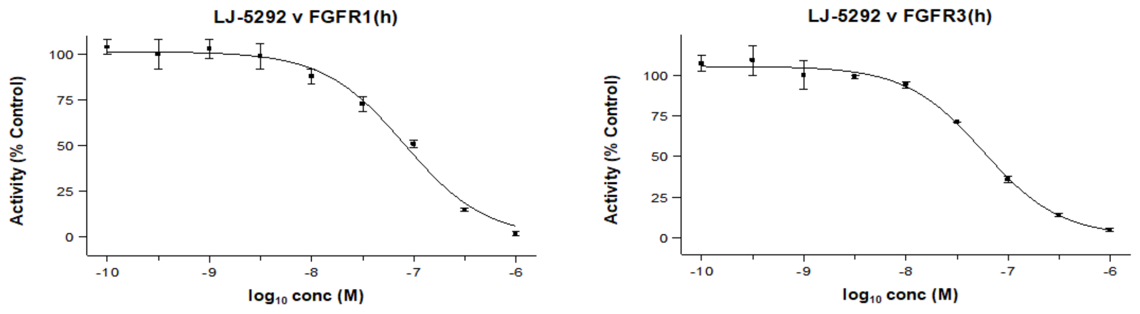

Figure S40. IC_{50} curves of compound **13a**

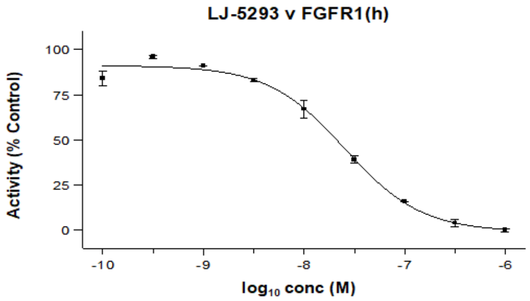

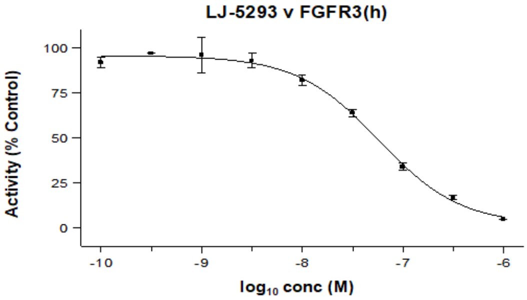

Figure S41. IC_{50} curves of compound **13b**

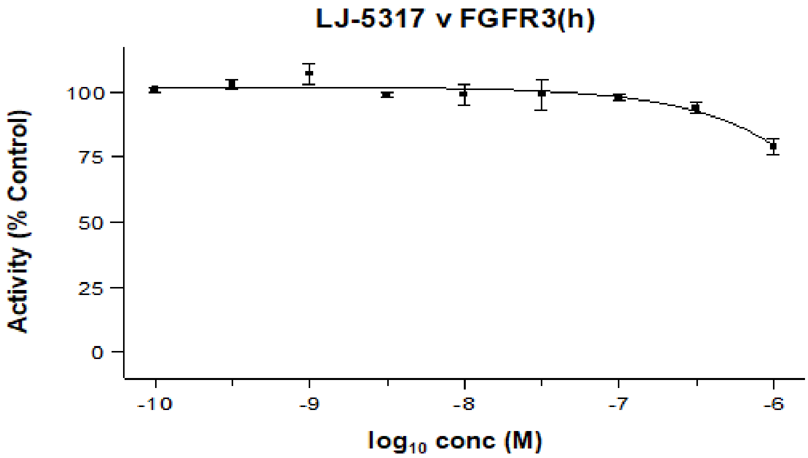

Figure S42. IC_{50} curves of compound **13c**

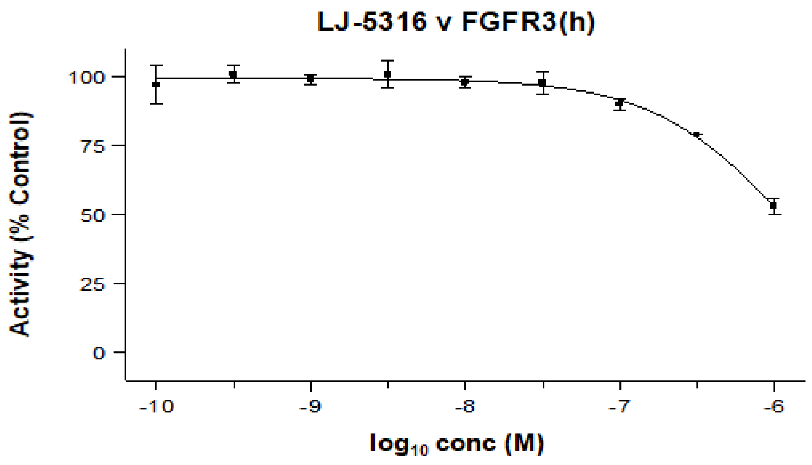

Figure S43. IC_{50} curves of compound **13d**

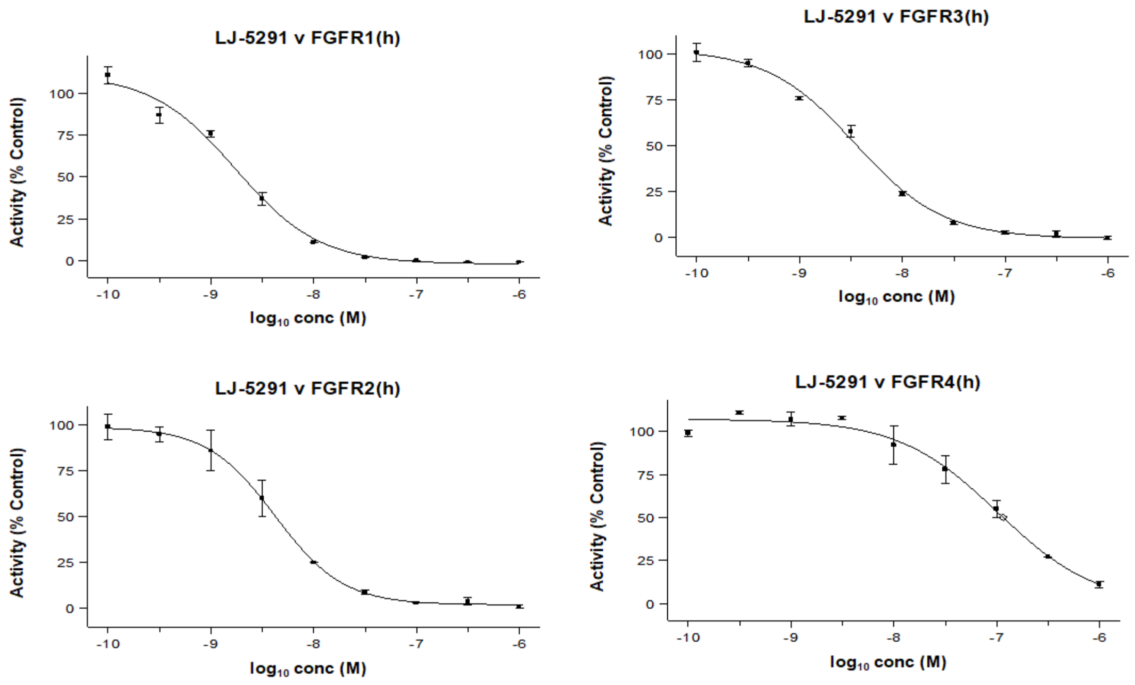

Figure S44. IC_{50} curves of compound **13f**

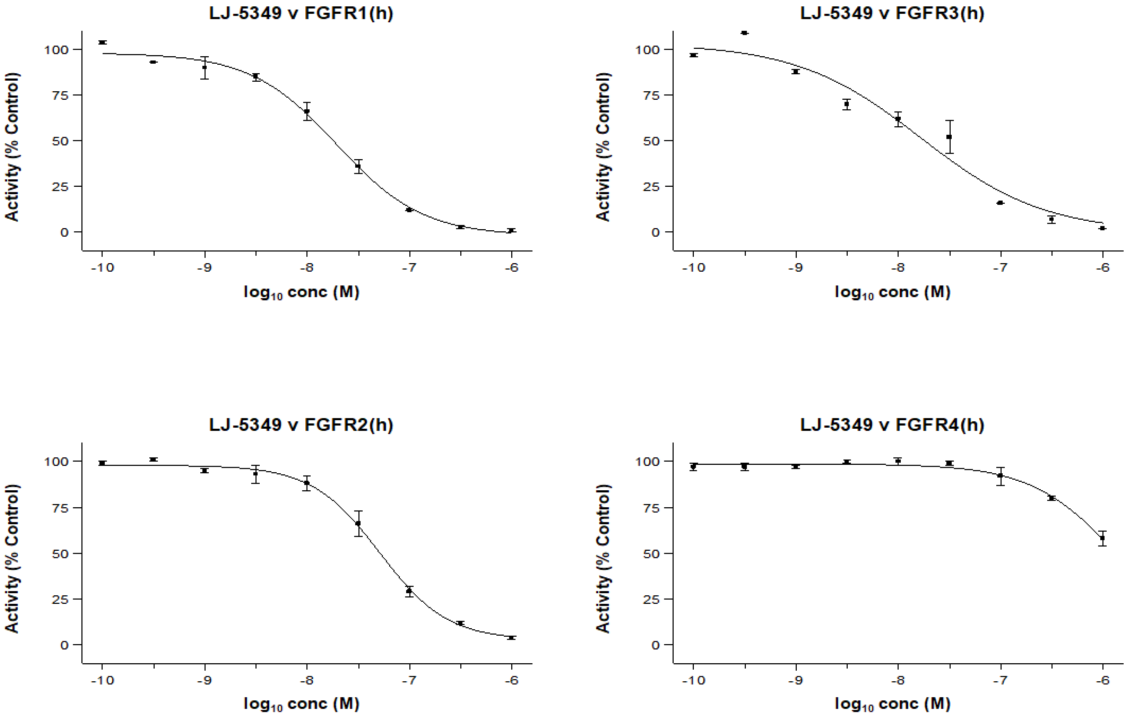

Figure S45. IC_{50} curves of compound ent-**13f**

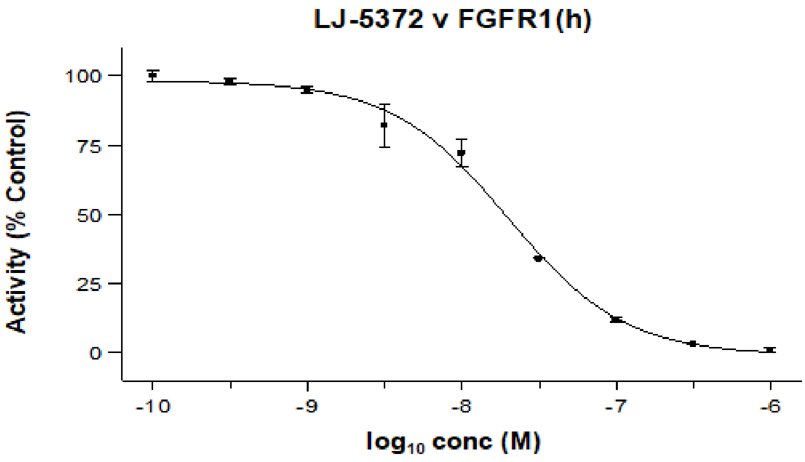

Figure S46. IC_{50} curves of compound **19a**

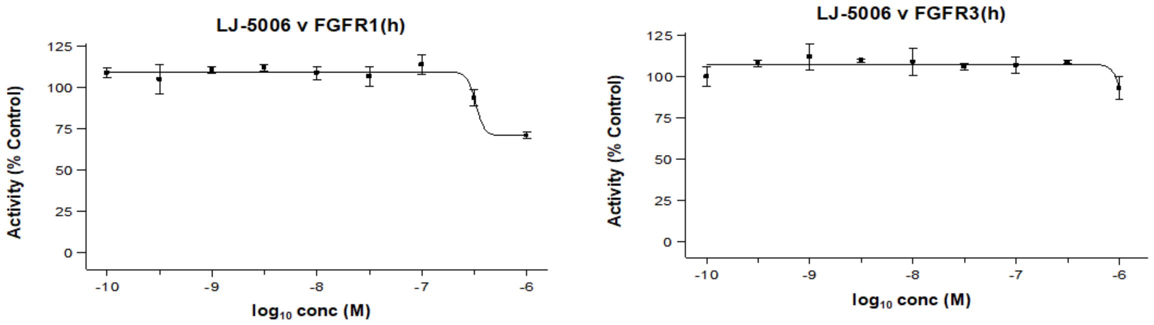

Figure S47. IC_{50} curves of compound **19b**

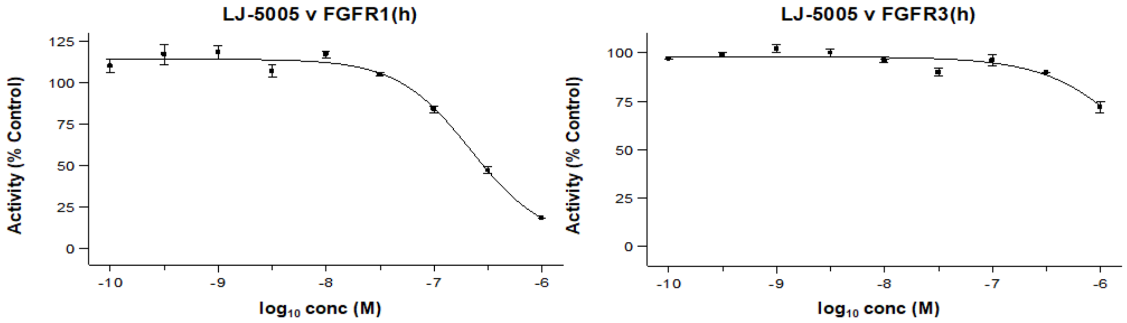

Figure S48. IC_{50} curves of compound **19c**

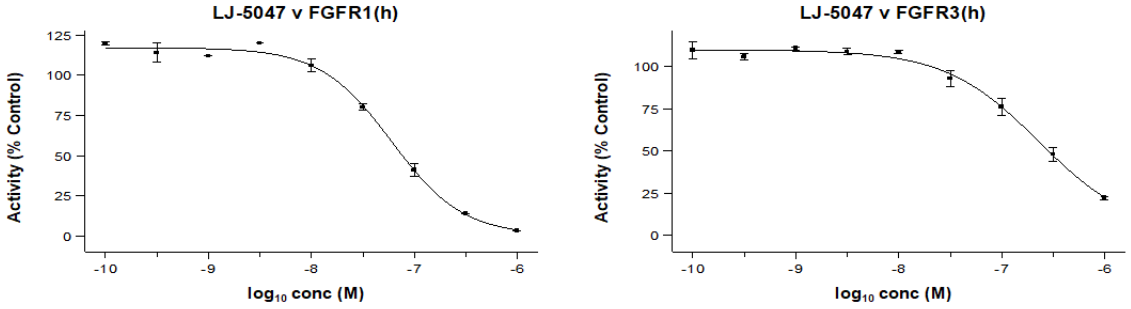

Figure S49. IC_{50} curves of compound **19d**

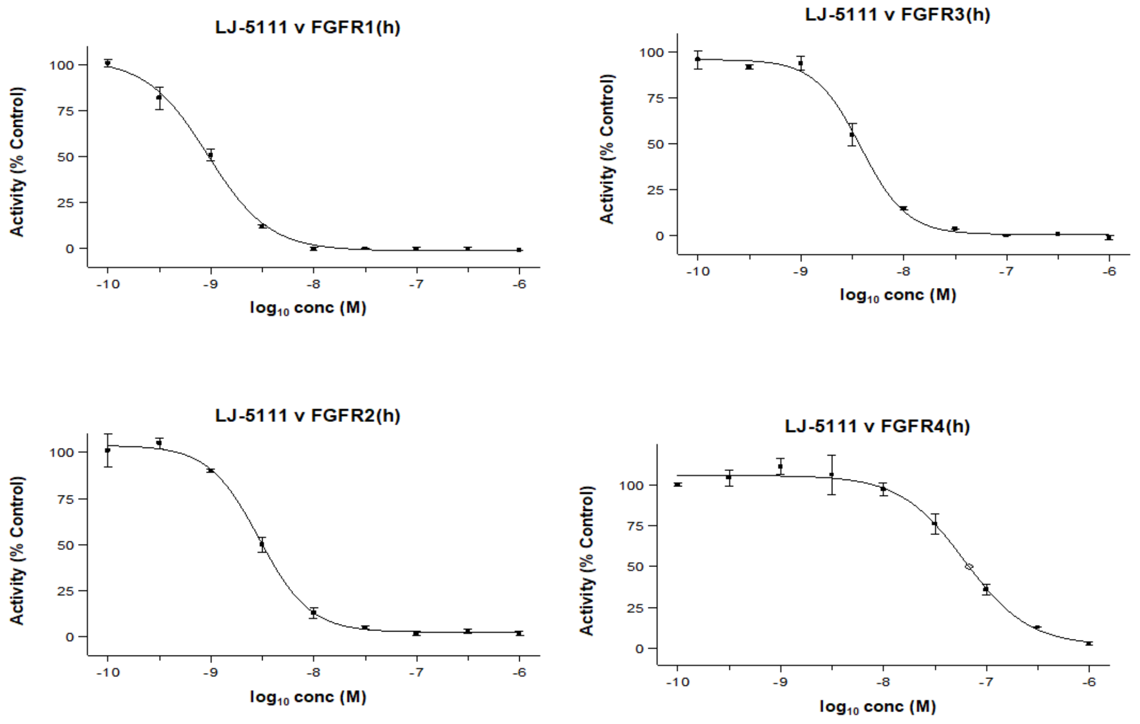

Figure S50. IC_{50} curves of compound **19e**

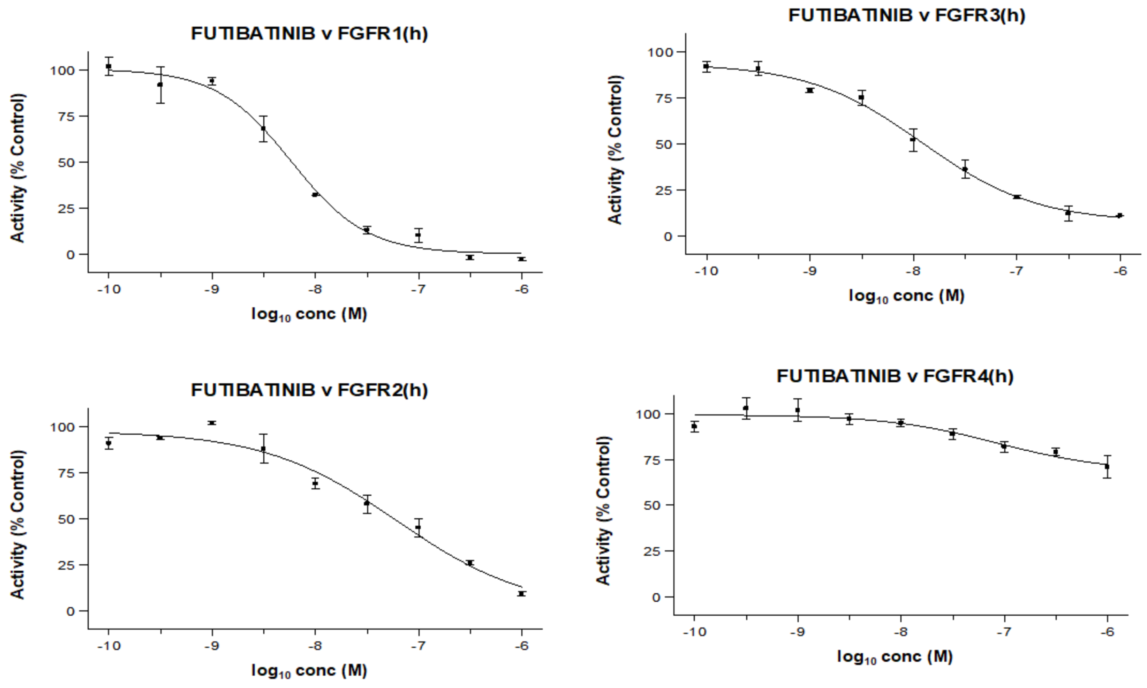

Figure S51. IC_{50} curves of **Futibatinib**

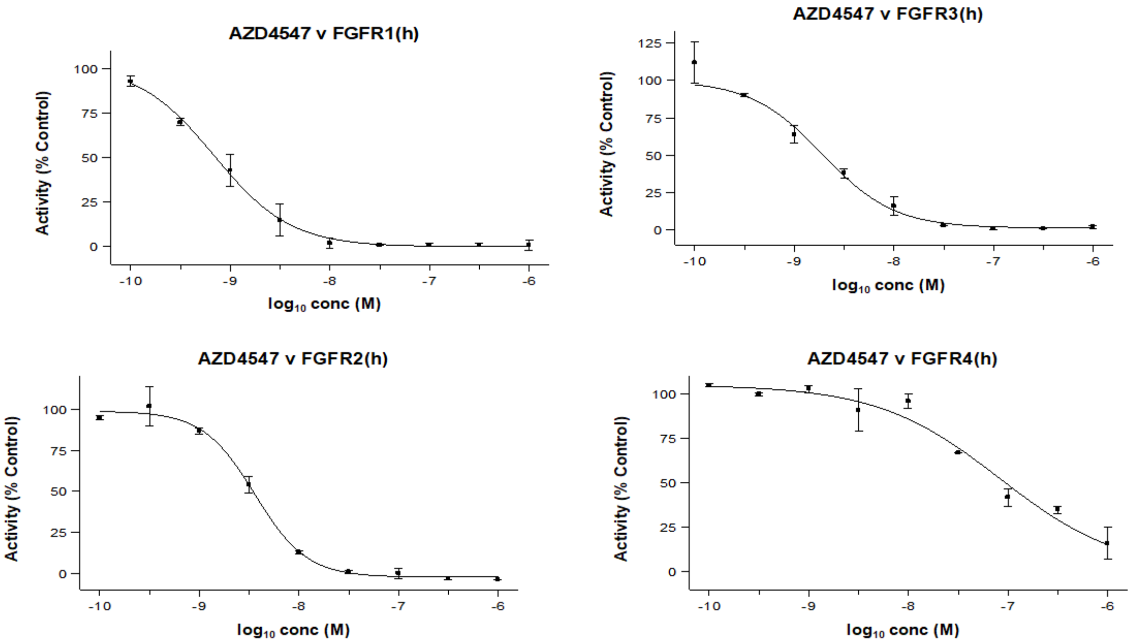

Figure S52. IC_{50} curves of **Fexagratinib**

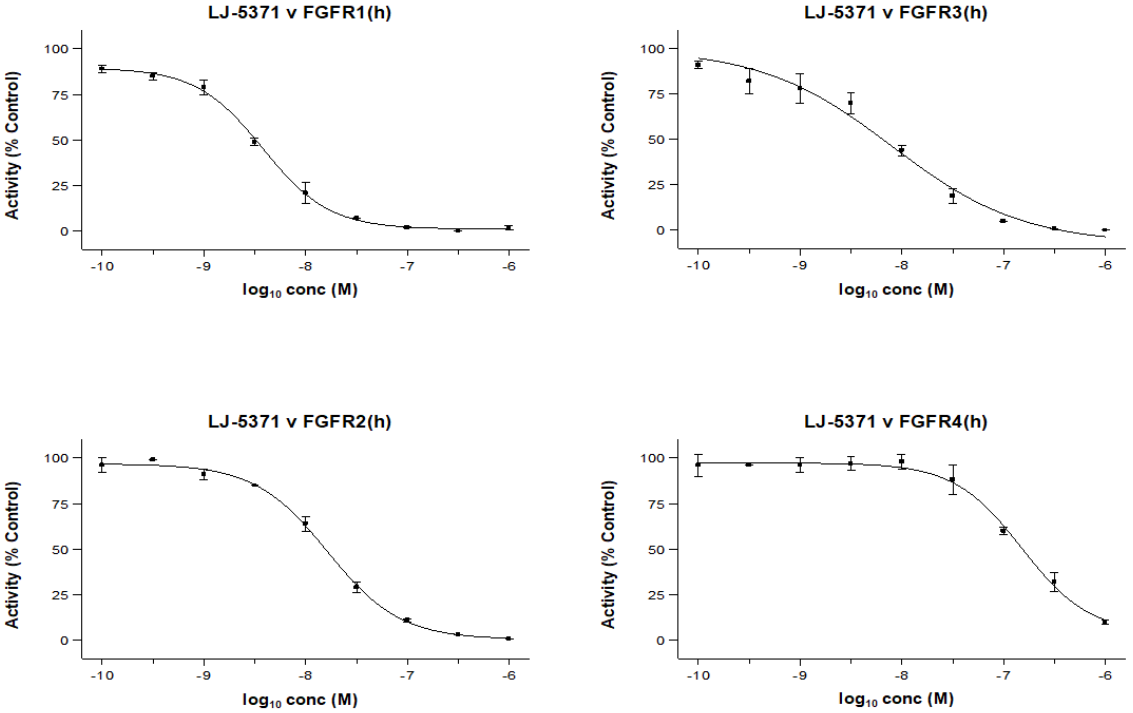

Figure S53. IC_{50} curves of compound **21**

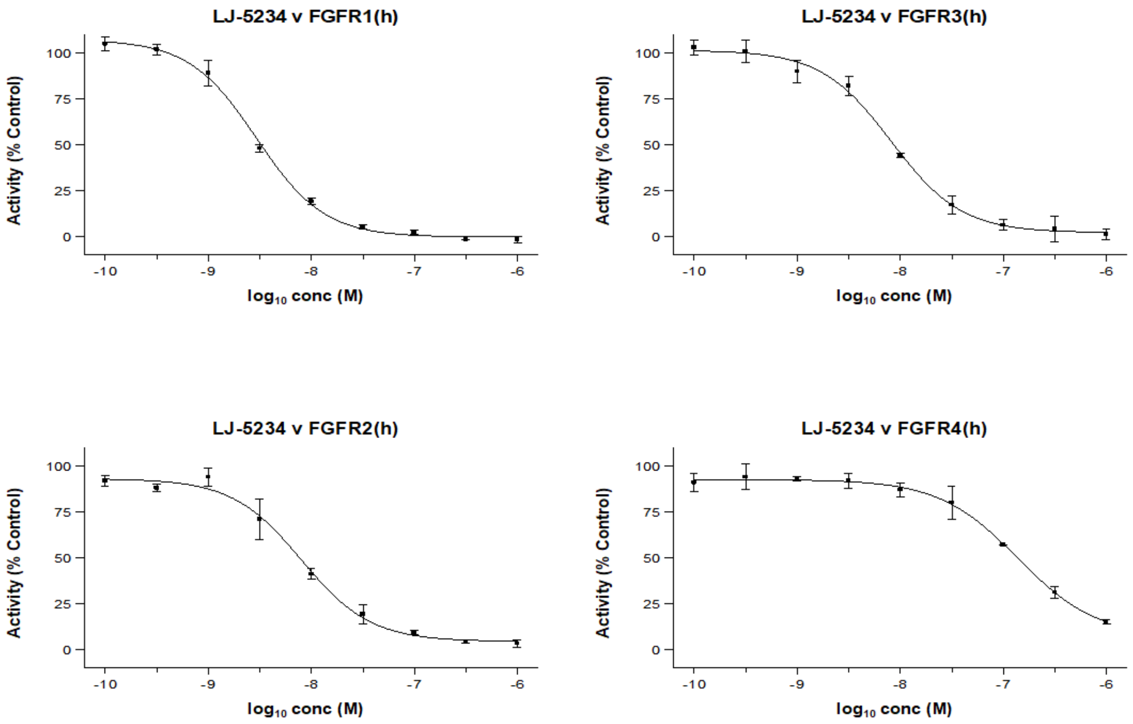

Figure S54. IC_{50} curves of compound **22a**

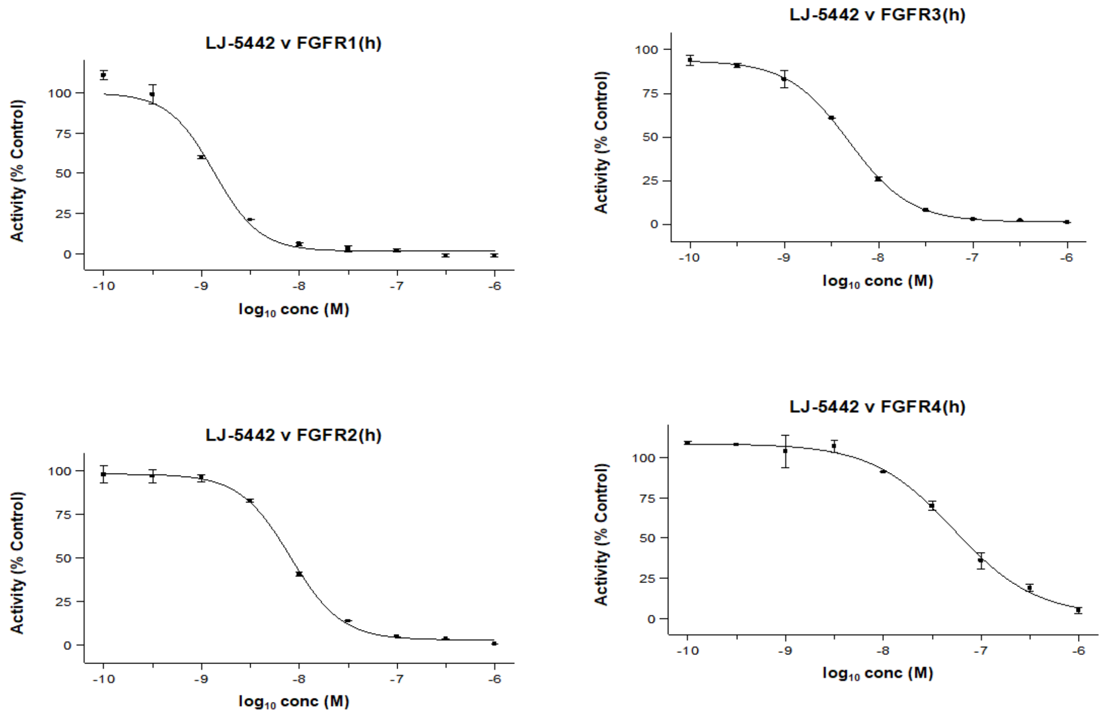

Figure S55. IC_{50} curves of compound **22f**

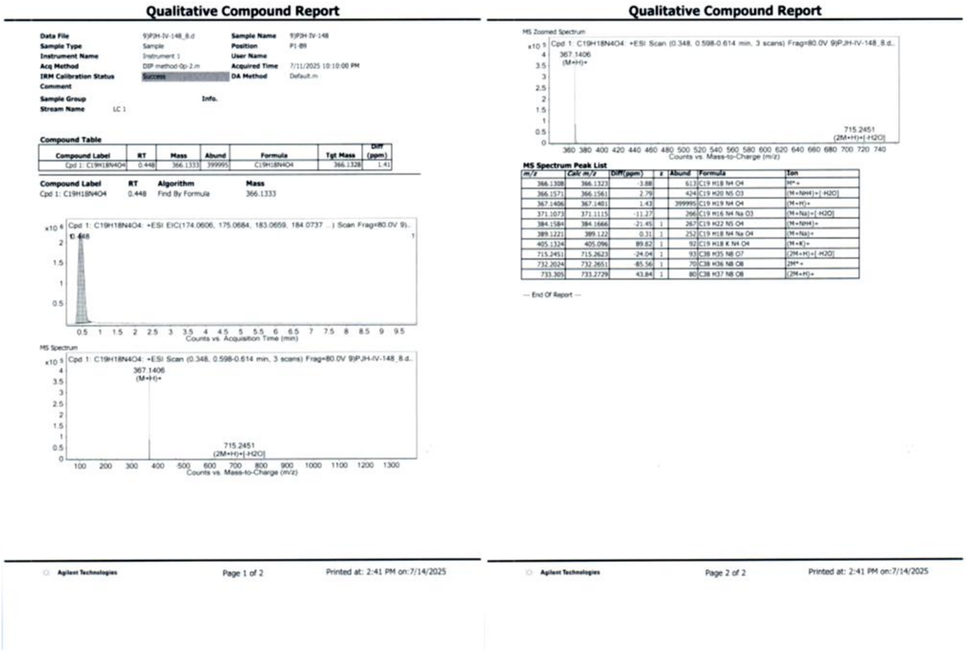

FigureS56. MS spectra of compound **5a**

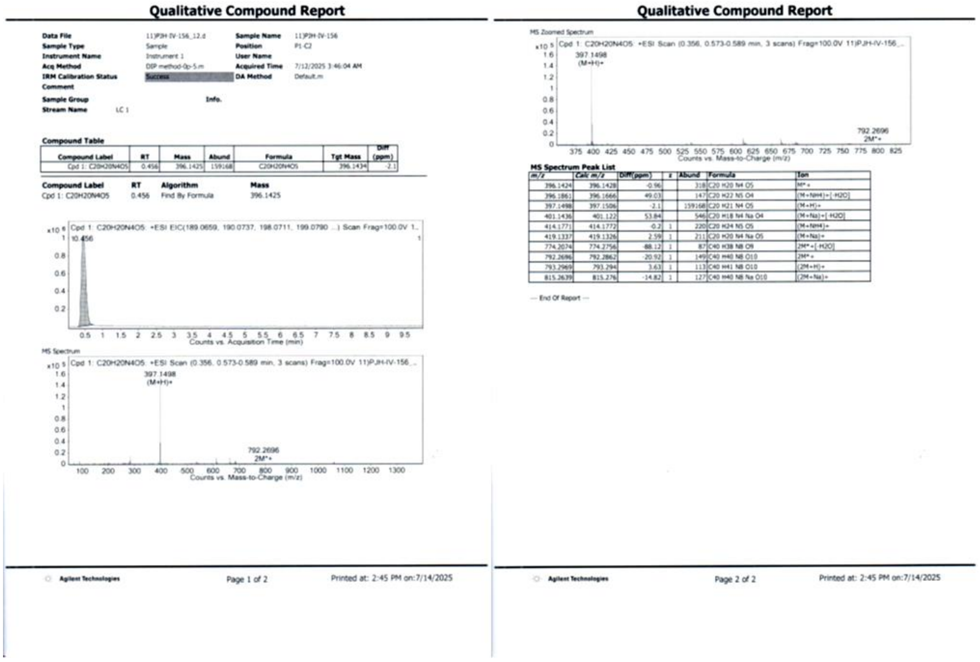

FigureS57. MS spectra of compound **5b**

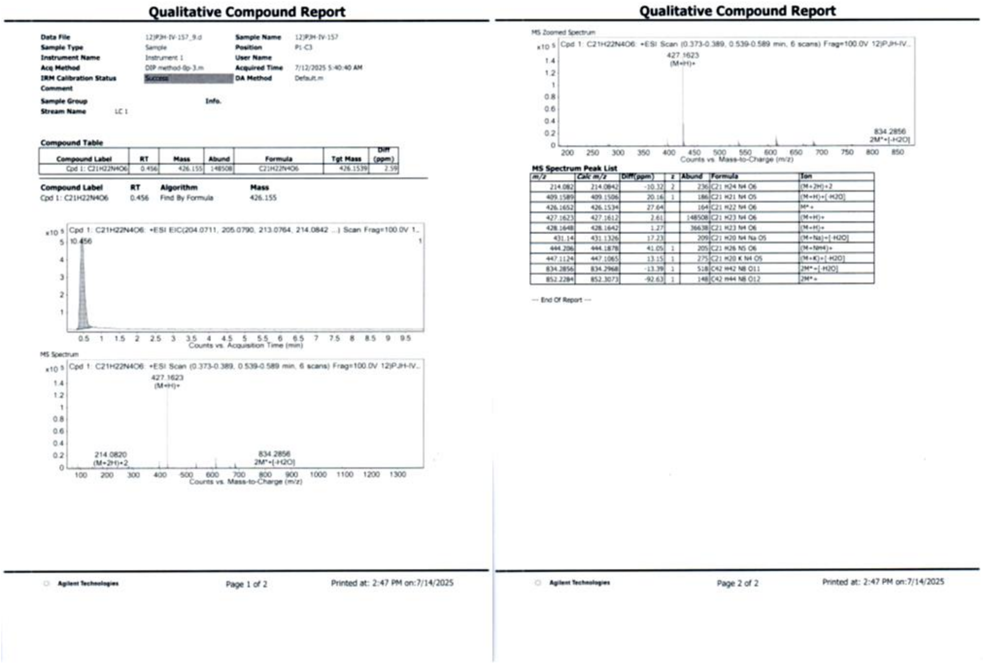

Qualitative Compound Report

Data File	12)PJH-IV-157_9.d	Sample Name	12)PJH-IV-157
Sample Type	Sample	Position	P1-C3
Instrument Name	Instrument 1	User Name	
Acq Method	DIP method-0p-3.m	Acquired Time	7/12/2025 5:40:40 AM
IRM Calibration Status	Success	DA Method	Default.m
Comment			
Sample Group		Info.	
Stream Name	LC 1		

Compound Table

Compound Label	RT	Mass	Abund	Formula	Tgt Mass	Diff (ppm)
Cpd 1: C21H22N4O6	0.456	426.155	148508	C21H22N4O6	426.1539	2.59

Compound Label	RT	Algorithm	Mass
Cpd 1: C21H22N4O6	0.456	Find By Formula	426.155

Agilent Technologies Page 1 of 2 Printed at: 2:47 PM on:7/14/2025

Qualitative Compound Report

MS Spectrum Peak List

m/z	Calc m/z	Diff(ppm)	z	Abund	Formula	Ion
214.082	214.0842	-10.32	2	236	C21 H24 N4 O6	(M+2H)+2
409.1589	409.1506	20.16	1	186	C21 H21 N4 O5	(M+H)+[-H2O]
426.1652	426.1534	27.64		164	C21 H22 N4 O6	M*+
427.1623	427.1612	2.61		148508	C21 H23 N4 O6	(M+H)+
428.1648	428.1642	1.27		36638	C21 H23 N4 O6	(M+H)+
431.14	431.1326	17.23		209	C21 H20 N4 Na O5	(M+Na)+[-H2O]
444.206	444.1878	41.05	1	205	C21 H26 N5 O6	(M+NH4)+
447.1124	447.1065	13.15	1	275	C21 H20 K N4 O5	(M+K)+[-H2O]
834.2856	834.2968	-13.39	1	518	C42 H42 N8 O11	2M*+[-H2O]
852.2284	852.3073	-92.63	1	148	C42 H44 N8 O12	2M*+

--- End Of Report ---

Agilent Technologies Page 2 of 2 Printed at: 2:47 PM on:7/14/2025

FigureS58. MS spectra of compound **5c**

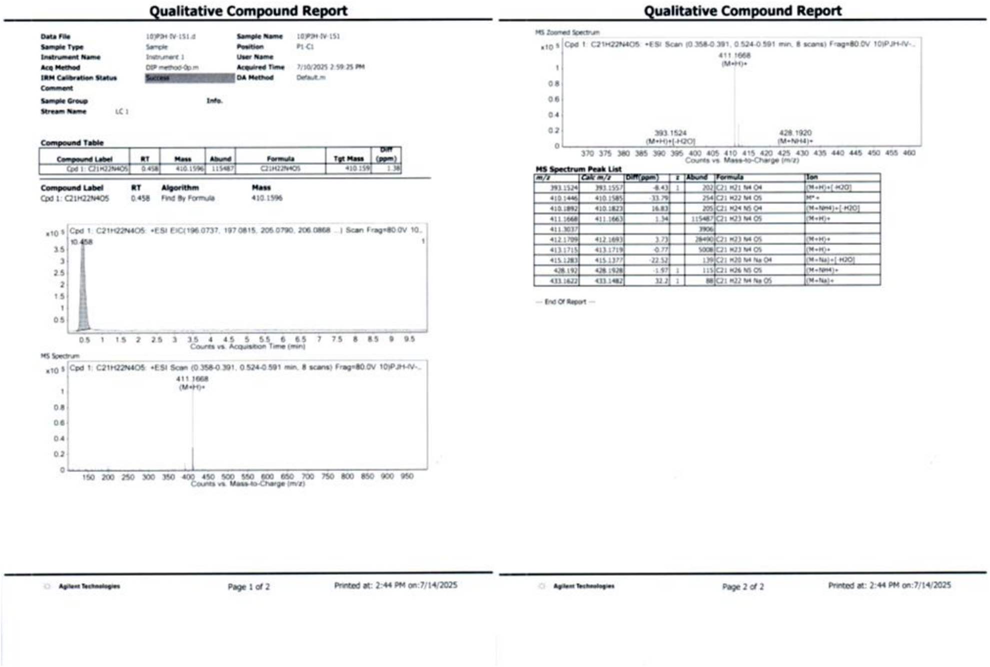

Qualitative Compound Report

Data File	10)PJH-IV-151.d	Sample Name	10)PJH-IV-151
Sample Type	Sample	Position	P1-C1
Instrument Name	Instrument 1	User Name	
Acq Method	DIP method-0p.m	Acquired Time	7/10/2025 2:59:25 PM
IRM Calibration Status	Success	DA Method	Default.m
Comment			
Sample Group		Info.	
Stream Name	LC 1		

Compound Table

Compound Label	RT	Mass	Abund	Formula	Tgt Mass	Diff (ppm)
Cpd 1: C21H22N4O5	0.458	410.1596	115487	C21H22N4O5	410.159	1.38

Compound Label	RT	Algorithm	Mass
Cpd 1: C21H22N4O5	0.458	Find By Formula	410.1596

Agilent Technologies Page 1 of 2 Printed at: 2:44 PM on:7/14/2025

Qualitative Compound Report

MS Spectrum Peak List

m/z	Calc m/z	Diff(ppm)	z	Abund	Formula	Ion
393.1524	393.1557	-8.43	1	202	C21 H21 N4 O4	(M+H)+[-H2O]
410.1446	410.1585	-33.79		254	C21 H22 N4 O5	M*+
410.1892	410.1823	16.83		205	C21 H24 N5 O4	(M+NH4)+[-H2O]
411.1668	411.1663	1.34		115487	C21 H23 N4 O5	(M+H)+
411.3037				3906		
412.1709	412.1693	3.73		28490	C21 H23 N4 O5	(M+H)+
413.1715	413.1719	-0.77		5008	C21 H23 N4 O5	(M+H)+
415.1283	415.1377	-22.52		139	C21 H20 N4 Na O4	(M+Na)+[-H2O]
428.192	428.1928	-1.97	1	115	C21 H26 N5 O5	(M+NH4)+
433.1622	433.1482	32.2	1	88	C21 H22 N4 Na O5	(M+Na)+

--- End Of Report ---

Agilent Technologies Page 2 of 2 Printed at: 2:44 PM on:7/14/2025

FigureS59. MS spectra of compound **5d**

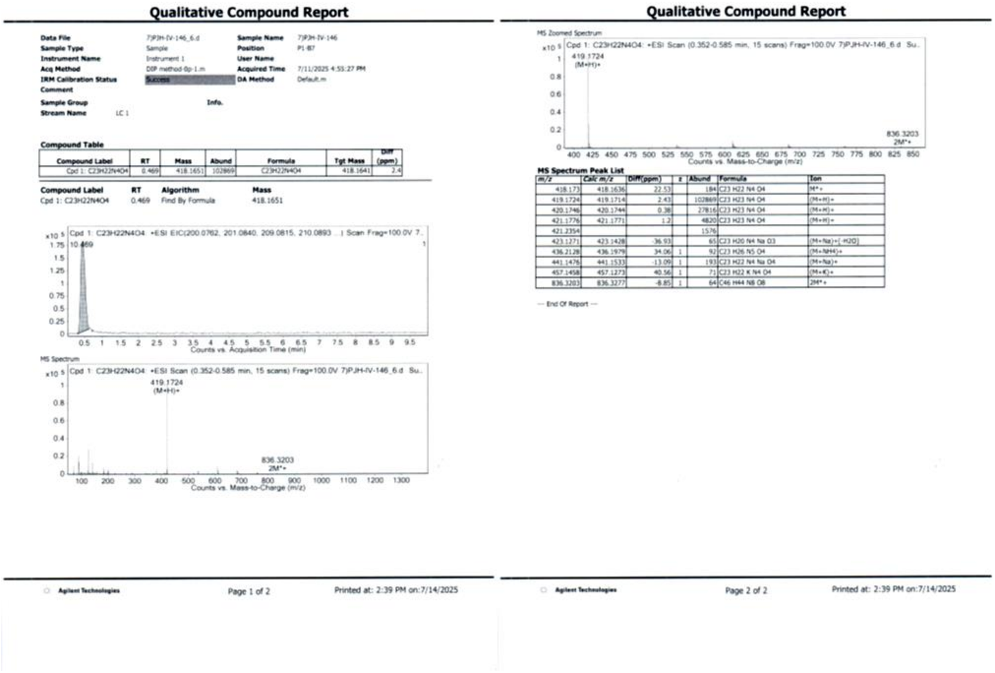

FigureS60. MS spectra of compound **6a**

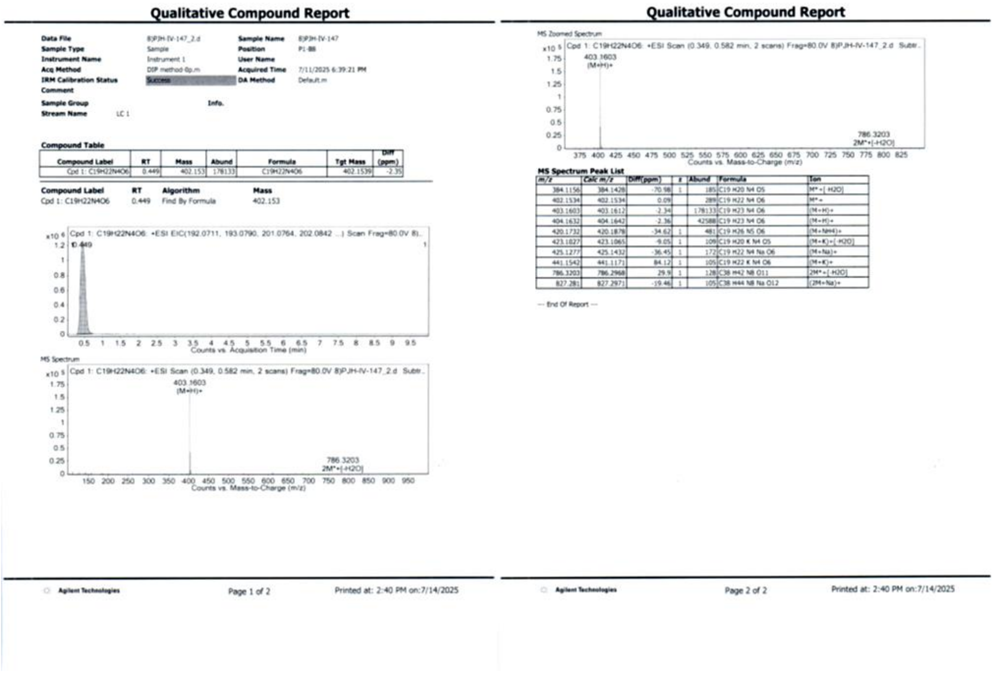

FigureS61. MS spectra of compound **6b**

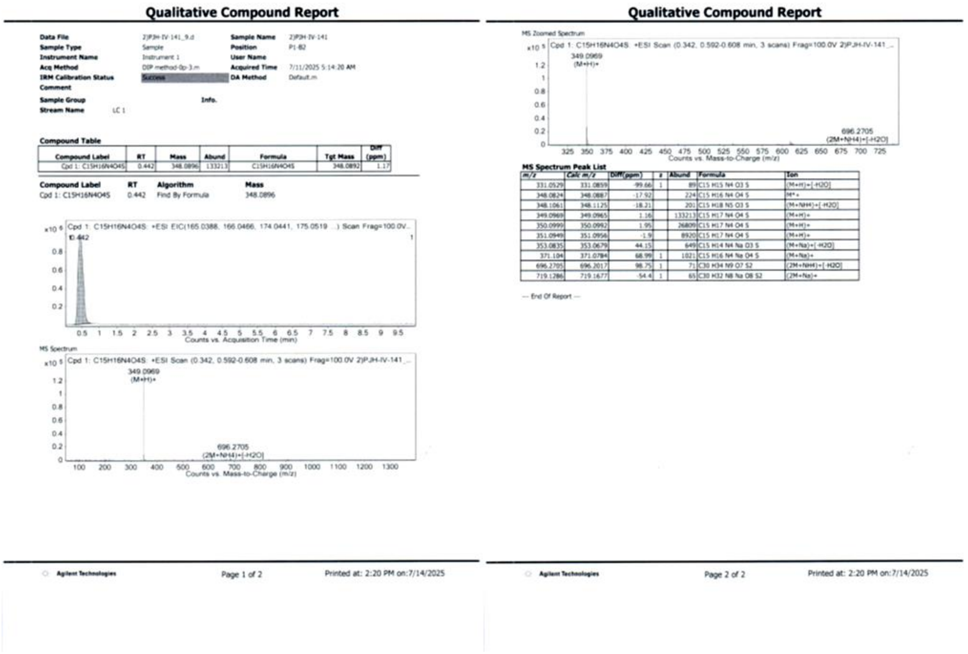

FigureS62. MS spectra of compound **6c**

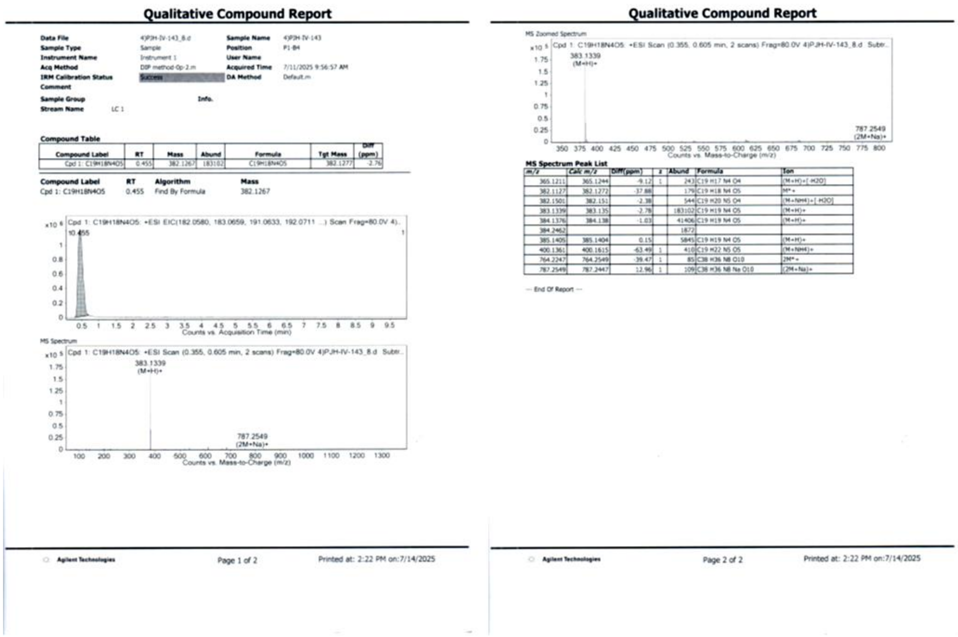

FigureS63. MS spectra of compound **6d**

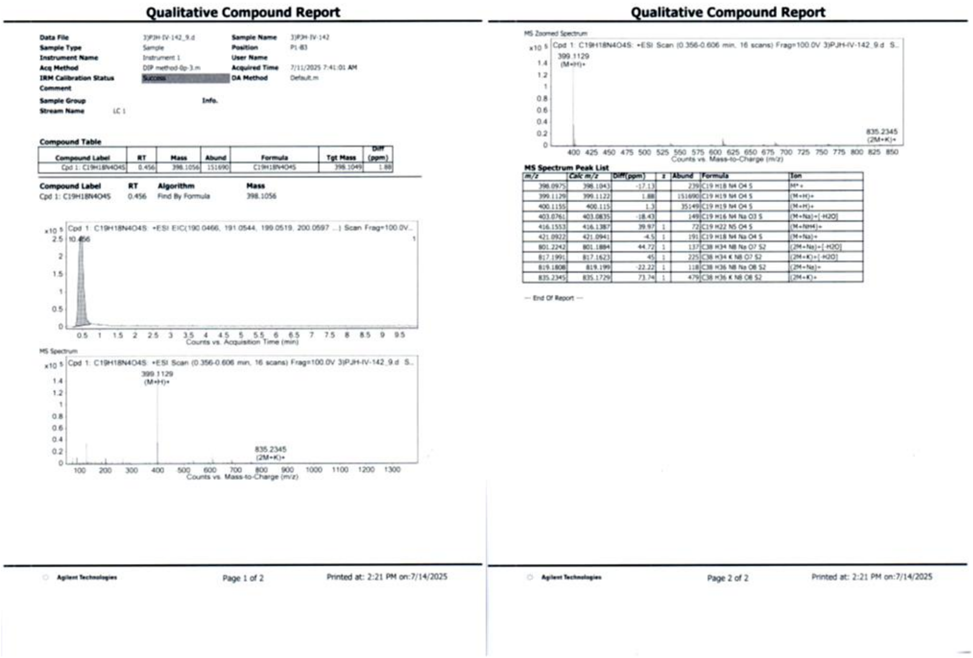

FigureS64. MS spectra of compound **6e**

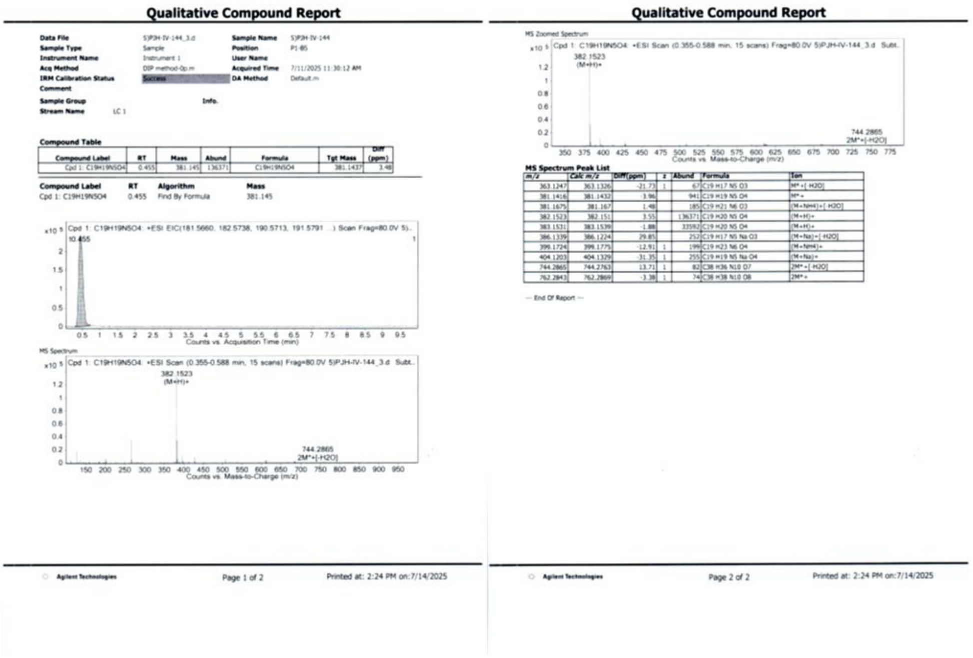

FigureS65. MS spectra of compound **6f**

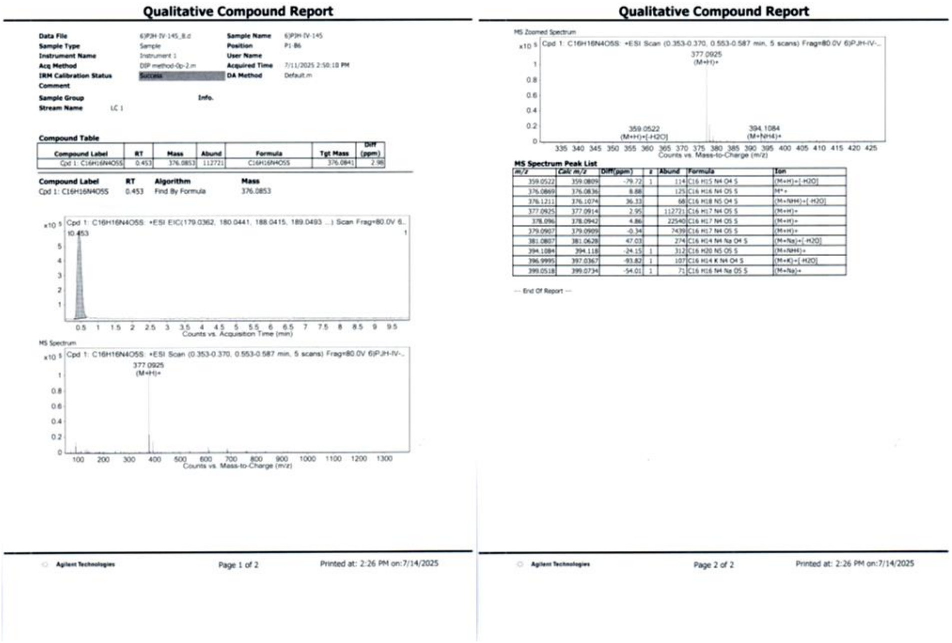

FigureS66. MS spectra of compound **6g**

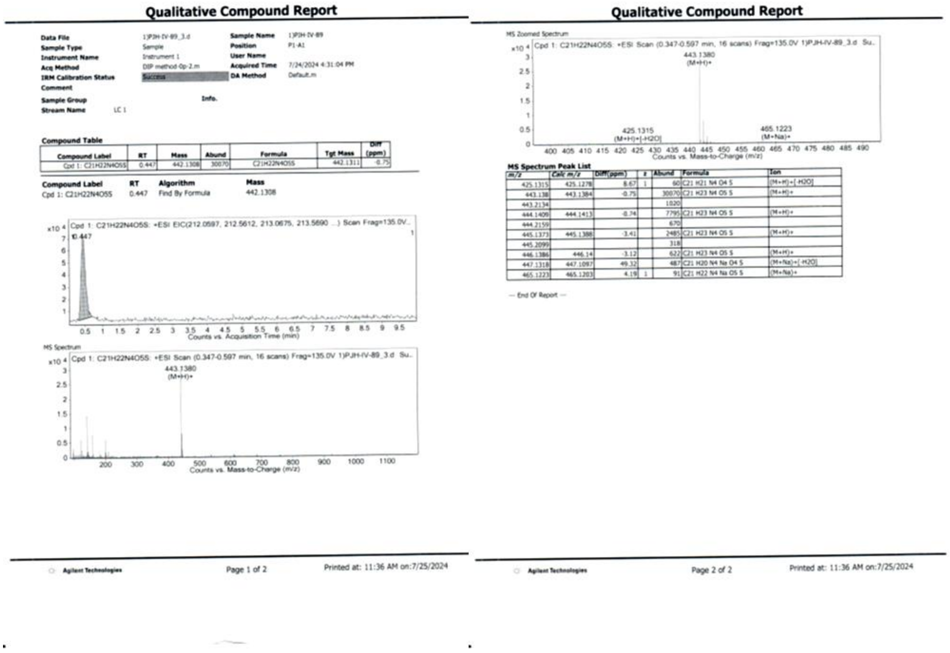

FigureS67. MS spectra of compound **6h**

Qualitative Compound Report

**Data File**	PJH-III-80.d	**Sample Name**	PJH-III-80
**Sample Type**	Sample	**Position**	P1-A1
**Instrument Name**	Instrument 1	**User Name**	
**Acq Method**	DIP method-0p.m	**Acquired Time**	6/12/2023 6:19:00 PM
**IRM Calibration Status**	Success	**DA Method**	Default.m
**Comment**			

Sample Group **Info.**
Stream Name LC 1

Compound Table

Compound Label	RT	Mass	Abund	Formula	Tgt Mass	Diff (ppm)
Cpd 1: C24H25N5O6	0.172	479.1807	165416	C24H25N5O6	479.1805	0.54

Compound Label	RT	Algorithm	Mass
Cpd 1: C24H25N5O6	0.172	Find By Formula	479.1807

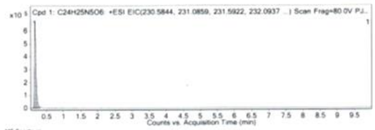

MS Spectrum

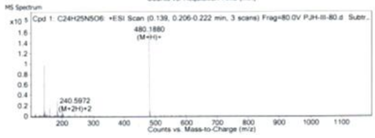

Qualitative Compound Report

MS Zoomed Spectrum

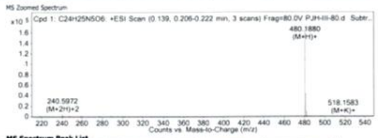

MS Spectrum Peak List

m/z	Calc m/z	Diff(ppm)	z	Abund	Formula	Ion
240.5972	240.5975	-1.16	2	5721	C24 H27 N5 O6	(M+2H)+2
479.1831	479.1799	6.66		191	C24 H25 N5 O6	M*+
479.2227	479.2037	39.52		51	C24 H27 N6 O5	(M+NH4)+[-H2O]
480.188	480.1878	0.52		165416	C24 H26 N5 O6	(M+H)+
481.1913	481.1907	1.09		43913	C24 H26 N5 O6	(M+H)+
482.1941	482.1933	1.71		8583	C24 H26 N5 O6	(M+H)+
484.1975	484.1591	79.29		434	C24 H23 N5 Na O5	(M+Na)+[-H2O]
497.1925	497.2143	-43.78	1	224	C24 H29 N6 O6	(M+NH4)+
502.1818	502.1697	24.09	1	333	C24 H25 N5 Na O6	(M+Na)+
518.1583	518.1436	28.23	1	851	C24 H25 K N5 O6	(M+K)+

--- End Of Report ---

FigureS68. MS spectra of compound **13a**

Qualitative Compound Report

**Data File**	1)PJH-III-90_8.d	**Sample Name**	1)PJH-III-90
**Sample Type**	Sample	**Position**	P1-D1
**Instrument Name**	Instrument 1	**User Name**	
**Acq Method**	DIP method-0p-5.m	**Acquired Time**	7/13/2023 9:07:20 AM
**IRM Calibration Status**	Success	**DA Method**	Default.m
**Comment**			

Sample Group **Info.**
Stream Name LC 1

Compound Table

Compound Label	RT	Mass	Abund	Formula	Tgt Mass	Diff (ppm)
Cpd 1: C24H25N5O5	0.186	463.1851	479454	C24H25N5O5	463.1856	-1.03

Compound Label	RT	Algorithm	Mass
Cpd 1: C24H25N5O5	0.186	Find By Formula	463.1851

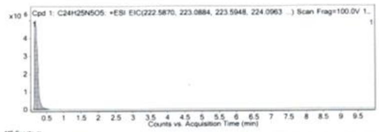

MS Spectrum

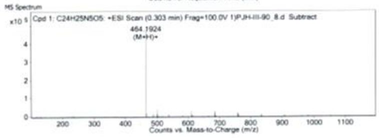

Qualitative Compound Report

MS Zoomed Spectrum

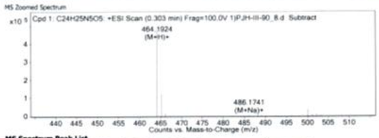

MS Spectrum Peak List

m/z	Calc m/z	Diff(ppm)	z	Abund	Formula	Ion
463.1824				776		
464.1924	464.1928	-1.02		479454	C24 H26 N5 O5	(M+H)+
465.1949	465.1958	-1.91		123845	C24 H26 N5 O5	(M+H)+
466.1981	466.1984	-0.63		18879	C24 H26 N5 O5	(M+H)+
467.1993	467.201	-3.48		2842	C24 H26 N5 O5	(M+H)+
467.258				547		
467.2889				468		
468.1971	468.2035	-13.68		583	C24 H26 N5 O5	(M+H)+
486.1741	486.1748	-1.37	1	5089	C24 H25 N5 Na O5	(M+Na)+
487.1815	487.1778	7.68	1	1123	C24 H25 N5 Na O5	(M+Na)+

--- End Of Report ---

FigureS69. MS spectra of compound **13b**

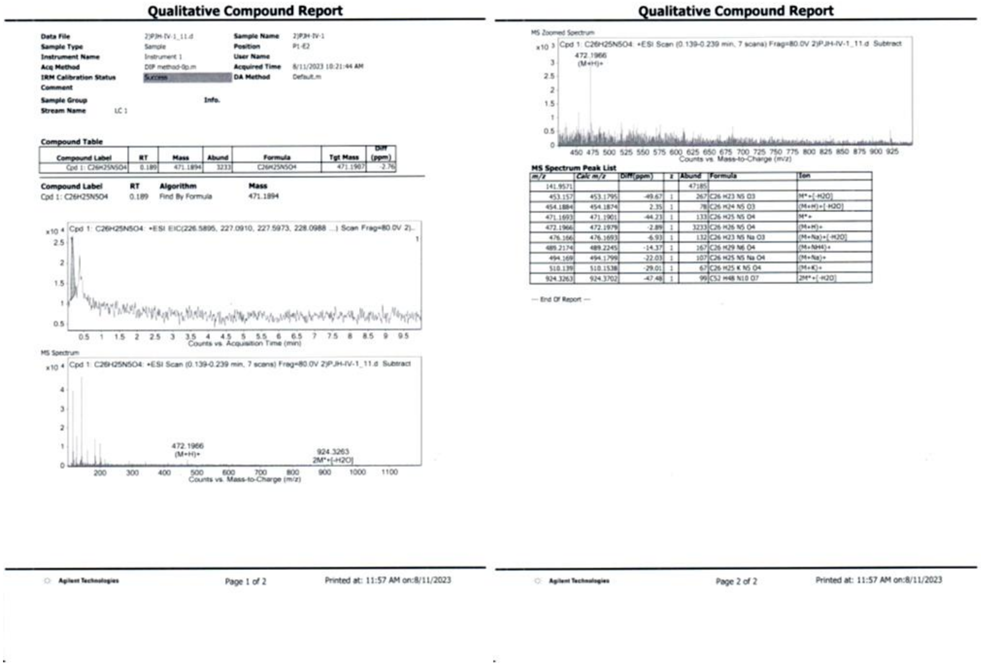

FigureS70. MS spectra of compound **13c**

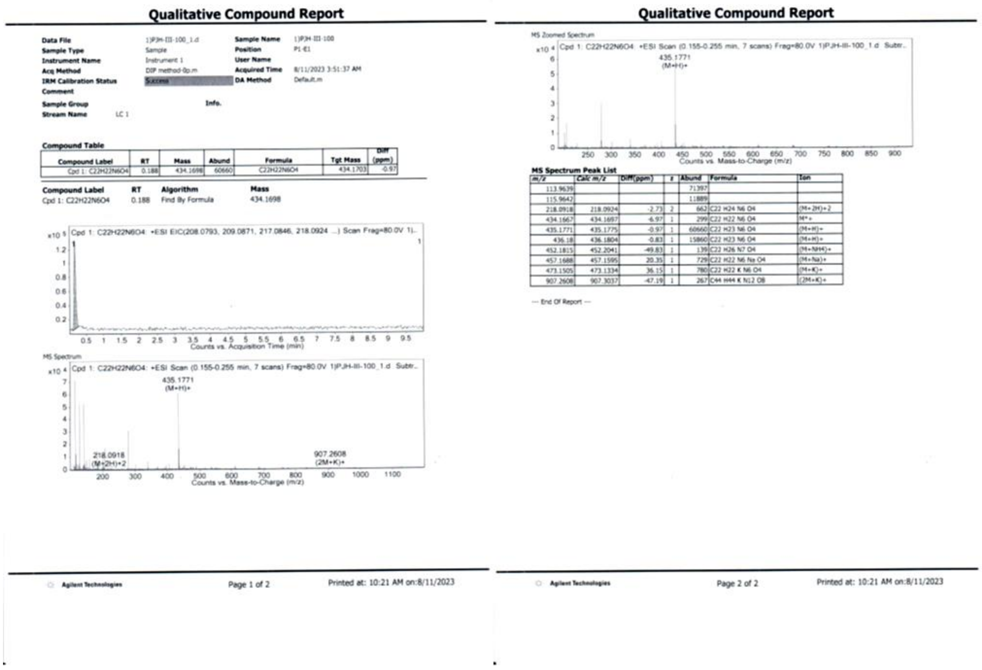

FigureS71. MS spectra of compound **13d**

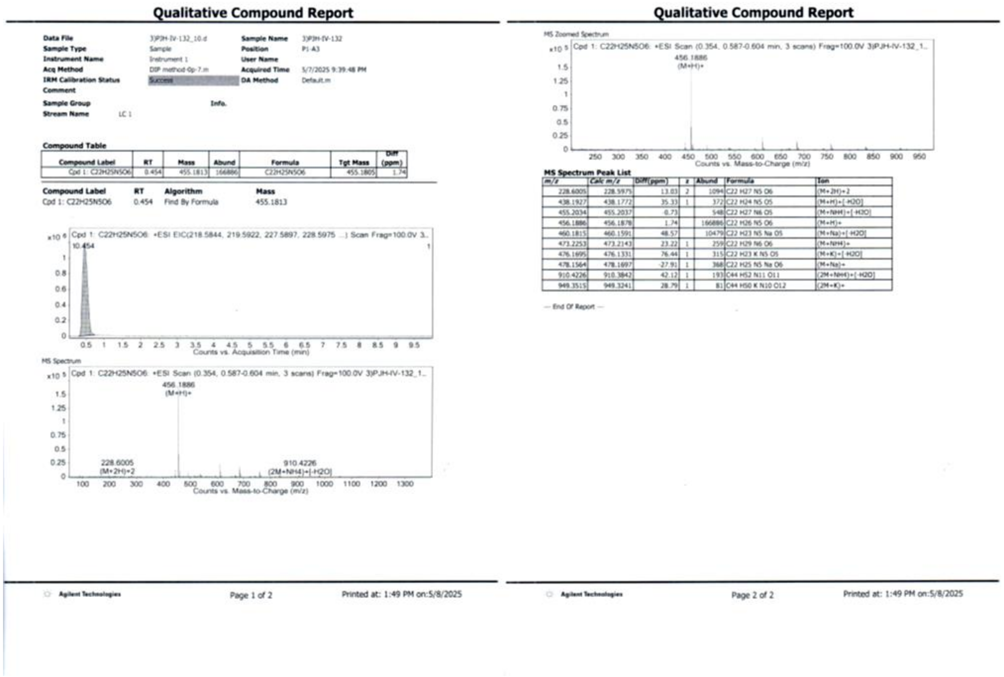

Qualitative Compound Report

**Data File**	3)PJH-IV-132_10.d	**Sample Name**	3)PJH-IV-132
**Sample Type**	Sample	**Position**	P1-A3
**Instrument Name**	Instrument 1	**User Name**	
**Acq Method**	DIP method-0p-7.m	**Acquired Time**	5/7/2025 9:39:48 PM
**IRM Calibration Status**	Success	**DA Method**	Default.m
**Comment**			

Sample Group **Info.**
Stream Name LC 1

Compound Table

Compound Label	RT	Mass	Abund	Formula	Tgt Mass	Diff (ppm)
Cpd 1: C22H25N5O6	0.454	455.1813	166886	C22H25N5O6	455.1805	1.74

Compound Label	RT	Algorithm	Mass
Cpd 1: C22H25N5O6	0.454	Find By Formula	455.1813

MS Zoomed Spectrum

MS Spectrum Peak List

m/z	Calc m/z	Diff(ppm)	z	Abund	Formula	Ion
228.6005	228.5975	13.03	2	1094	C22 H27 N5 O6	(M+2H)+2
438.1927	438.1772	35.33	1	372	C22 H24 N5 O5	(M+H)+[-H2O]
455.2034	455.2037	-0.73		548	C22 H27 N6 O5	(M+NH4)+[-H2O]
456.1886	456.1878	1.74		166886	C22 H26 N5 O6	(M+H)+
460.1815	460.1591	48.57		10479	C22 H23 N5 Na O5	(M+Na)+[-H2O]
473.2253	473.2143	23.22	1	259	C22 H29 N6 O6	(M+NH4)+
476.1695	476.1331	76.44	1	315	C22 H23 K N5 O5	(M+K)+[-H2O]
478.1564	478.1697	-27.91	1	368	C22 H25 N5 Na O6	(M+Na)+
910.4226	910.3842	42.12	1	193	C44 H52 N11 O11	(2M+NH4)+[-H2O]
949.3515	949.3241	28.79	1	81	C44 H50 K N10 O12	(2M+K)+

--- End Of Report ---

Agilent Technologies Page 1 of 2 Printed at: 1:49 PM on:5/8/2025

Agilent Technologies Page 2 of 2 Printed at: 1:49 PM on:5/8/2025

FigureS72. MS spectra of compound **13e**

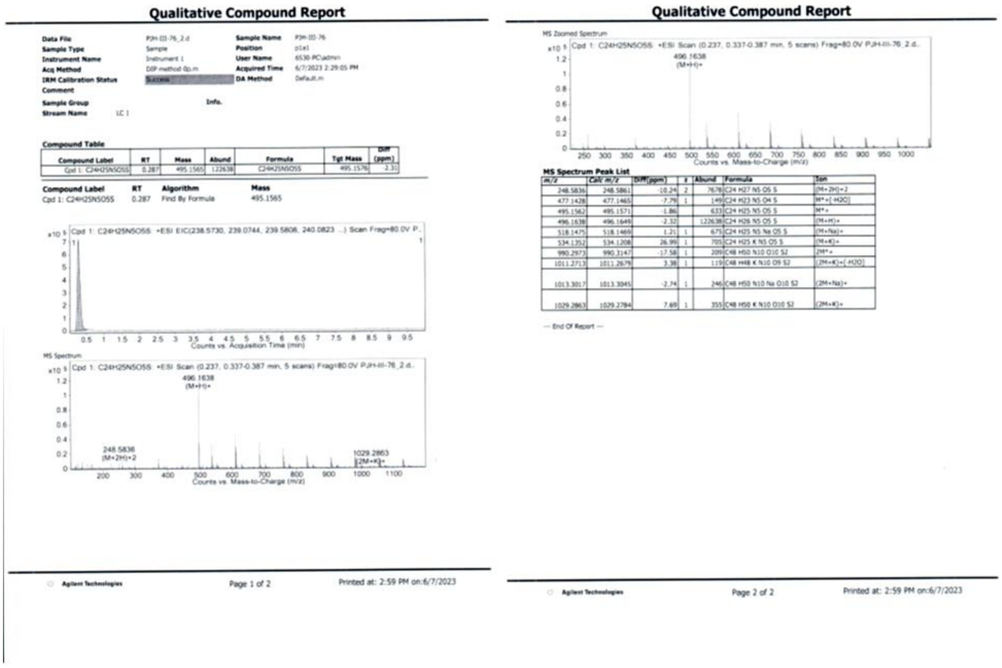

Qualitative Compound Report

**Data File**	PJH-III-76_2.d	**Sample Name**	PJH-III-76
**Sample Type**	Sample	**Position**	p1a1
**Instrument Name**	Instrument 1	**User Name**	6530-PC\admin
**Acq Method**	DIP method-0p.m	**Acquired Time**	6/7/2023 2:29:05 PM
**IRM Calibration Status**	Success	**DA Method**	Default.m
**Comment**			

Sample Group **Info.**
Stream Name LC 1

Compound Table

Compound Label	RT	Mass	Abund	Formula	Tgt Mass	Diff (ppm)
Cpd 1: C24H25N5O5S	0.287	495.1565	122638	C24H25N5O5S	495.1576	-2.31

Compound Label	RT	Algorithm	Mass
Cpd 1: C24H25N5O5S	0.287	Find By Formula	495.1565

MS Zoomed Spectrum

MS Spectrum Peak List

m/z	Calc m/z	Diff(ppm)	z	Abund	Formula	Ion
248.5836	248.5861	-10.24	2	7678	C24 H27 N5 O5 S	(M+2H)+2
477.1428	477.1465	-7.79	1	149	C24 H23 N5 O4 S	M*+[-H2O]
495.1562	495.1571	-1.86		633	C24 H25 N5 O5 S	M*+
496.1638	496.1649	-2.32		122638	C24 H26 N5 O5 S	(M+H)+
518.1475	518.1469	1.21	1	675	C24 H25 N5 Na O5 S	(M+Na)+
534.1352	534.1208	26.99	1	705	C24 H25 K N5 O5 S	(M+K)+
990.2973	990.3147	-17.58	1	209	C48 H50 N10 O10 S2	2M*+
1011.2713	1011.2679	3.38	1	119	C48 H48 K N10 O9 S2	(2M+K)+[-H2O]
1013.3017	1013.3045	-2.74	1	246	C48 H50 N10 Na O10 S2	(2M+Na)+
1029.2863	1029.2784	7.69	1	355	C48 H50 K N10 O10 S2	(2M+K)+

--- End Of Report ---

Agilent Technologies Page 1 of 2 Printed at: 2:59 PM on:6/7/2023

Agilent Technologies Page 2 of 2 Printed at: 2:59 PM on:6/7/2023

FigureS73. MS spectra of compound **13f**

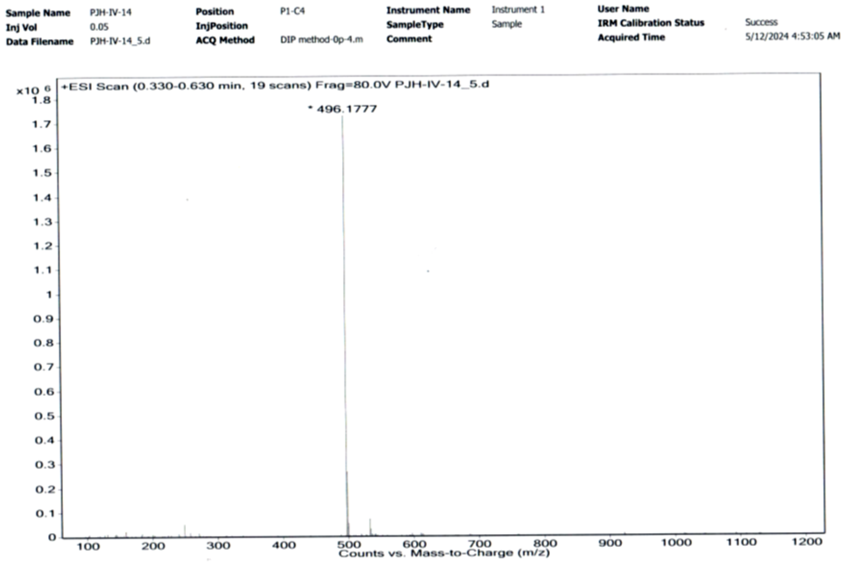

FigureS74. MS spectra of compound **ent-13f**

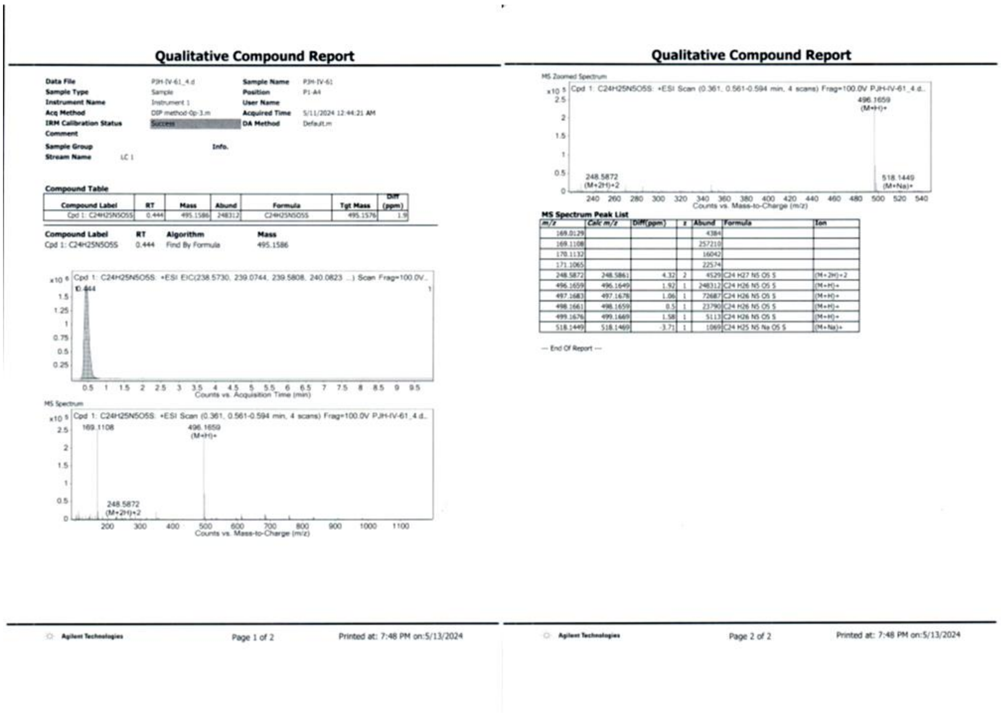

FigureS75. MS spectra of compound **19a**

```
[ Theoretical Ion Distribution ]
Molecular Formula : C24 H29 N6 O5 S2
                    (m/z   545.1641, MW   545.6635, U.S. 15.5)
Base Peak :   545.1641, Averaged MW :   545.6575(a),   545.6587(w)

   m/z       INT.
 545.1641 100.0000**********************************************************
 546.1668  30.6668******************
 547.1635  14.4072********
 548.1646   3.3877**
 549.1634   0.7882
 550.1638   0.1387
 551.1641   0.0212
 552.1649   0.0027
 553.1658   0.0003
```

Data : F38813 Date : 12-Aug-2021 16:47
Instrument : MStation
Sample : PJH-THIO
Note : m-NBA
Inlet : Direct Ion Mode : FAB+
RT : 0.00 min Scan# : (1,169)
Elements : C 50/0, H 100/0, N 8/0, O 7/0, S 2/0
Mass Tolerance : 10ppm, 5mmu if m/z < 500, 10mmu if m/z > 1000
Unsaturation (U.S.) : -0.5 - 20.0

	Observed m/z	Int%	Err[ppm / mmu]	U.S.	Composition
1	545.1633	2.42	-4.8 / -2.6	19.0	C26 H23 N7 O7
2			+4.7 / +2.6	19.5	C27 H25 N6 O5 S
3			+2.3 / +1.2	19.0	C29 H27 N3 O6 S
4			-0.2 / -0.1	18.5	C31 H29 O7 S
5			-6.4 / -3.5	20.0	C27 H27 N7 O2 S2
6			-8.8 / -4.8	19.5	C29 H29 N4 O3 S2
7			-1.4 / -0.8	15.5	C24 H29 N6 O5 S2
8			-3.9 / -2.1	15.0	C26 H31 N3 O6 S2
9			-6.4 / -3.5	14.5	C28 H33 O7 S2
10			+5.9 / +3.2	11.5	C19 H29 N8 O7 S2

FigureS76. MS spectra of compound **19b**

```
[ Theoretical Ion Distribution ]
Molecular Formula : C18 H20 N5 O4 S
                   (m/z   402.1236, MW   402.4539, U.S. 12.5)
Base Peak :   402.1236, Averaged MW :   402.4504(a),   402.4514(w)

   m/z        INT.
 402.1236 100.0000**********************************************************
 403.1263  22.7986*************
 404.1234   7.7136****
 405.1246   1.3292*
 406.1254   0.1895
 407.1268   0.0215
 408.1283   0.0020
 409.1299   0.0002
```

```
Data : F38383      Date : 02-Jun-2021 15:46
Instrument : MStation
Sample : PJH-I-31
Note : m-NBA
Inlet : Direct        Ion Mode : FAB+
RT : 7.99 min        Scan# : (138,205)
Elements : C 50/0, H 100/0, N 7/0, O 10/0, S 1/0
Mass Tolerance      : 10ppm, 5mmu if m/z < 500, 10mmu if m/z > 1000
Unsaturation (U.S.) : -0.5 - 15.0
```

	Observed m/z	Int%	Err[ppm / mmu]	U.S.	Composition
1	402.1231	15.68	+10.5 / +4.2	11.5	C20 H20 N O8
2			-7.5 / -3.0	7.5	C14 H20 N5 O9
3			-10.8 / -4.3	7.0	C16 H22 N2 O10
4			-1.2 / -0.5	12.5	C18 H20 N5 O4 S
5			-4.6 / -1.8	12.0	C20 H22 N2 O5 S
6			+8.8 / +3.5	8.5	C13 H20 N7 O6 S
7			+5.4 / +2.2	8.0	C15 H22 N4 O7 S
8			+2.1 / +0.8	7.5	C17 H24 N O8 S
9			+12.1 / +4.9	3.5	C12 H24 N3 O10 S

FigureS77. MS spectra of compound **19c**

Qualitative Compound Report

Data File	PJH-I-84-up_6.d	Sample Name	PJH-I-84-up
Sample Type	Sample	Position	Vial 2
Instrument Name	Instrument 1	User Name	
Acq Method	DIP method-0p.m	Acquired Time	10/6/2021 10:49:18 AM
IRM Calibration Status	Success	DA Method	Default.m
Comment			
Sample Group		Info.	
Stream Name	LC 1		

Compound Table

Compound Label	RT	Mass	Abund	Formula	Tgt Mass	Diff (ppm)
Cpd 1: C22H25N5O5S	0.194	471.1583	109436	C22H25N5O5S	471.1576	1.45

Compound Label	RT	Algorithm	Mass
Cpd 1: C22H25N5O5S	0.194	Find By Formula	471.1583

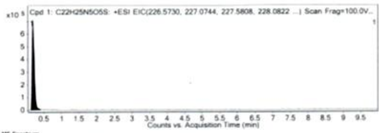

MS Spectrum

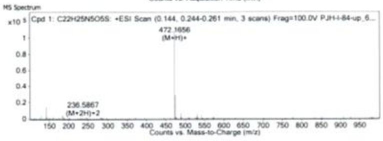

Qualitative Compound Report

MS Zoomed Spectrum

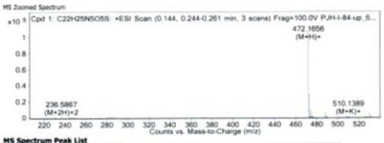

MS Spectrum Peak List

m/z	Calc m/z	Diff(ppm)	z	Abund	Formula	Ion
236.5867	236.5861	2.68	2	1232	C22 H27 N5 O5 S	(M+2H)+2
471.1696	471.1809	-23.97		175	C22 H27 N6 O4 S	(M+NH4)+[-H2O]
472.1656	472.1649	1.43		109436	C22 H26 N5 O5 S	(M+H)+
473.1689	473.1678	2.36		29065	C22 H26 N5 O5 S	(M+H)+
474.1668	474.1655	2.72		9369	C22 H26 N5 O5 S	(M+H)+
475.1684	475.1666	3.67		2008	C22 H26 N5 O5 S	(M+H)+
476.1608	476.1363	51.37		1518	C22 H23 N5 Na O4 S	(M+Na)+[-H2O]
489.2015	489.1915	20.44	1	2403	C22 H29 N6 O5 S	(M+NH4)+
494.1487	494.1469	3.66	1	199	C22 H25 N5 Na O5 S	(M+Na)+
510.1389	510.1208	35.49	1	892	C22 H25 K N5 O5 S	(M+K)+

--- End Of Report ---

FigureS78. MS spectra of compound **19d**

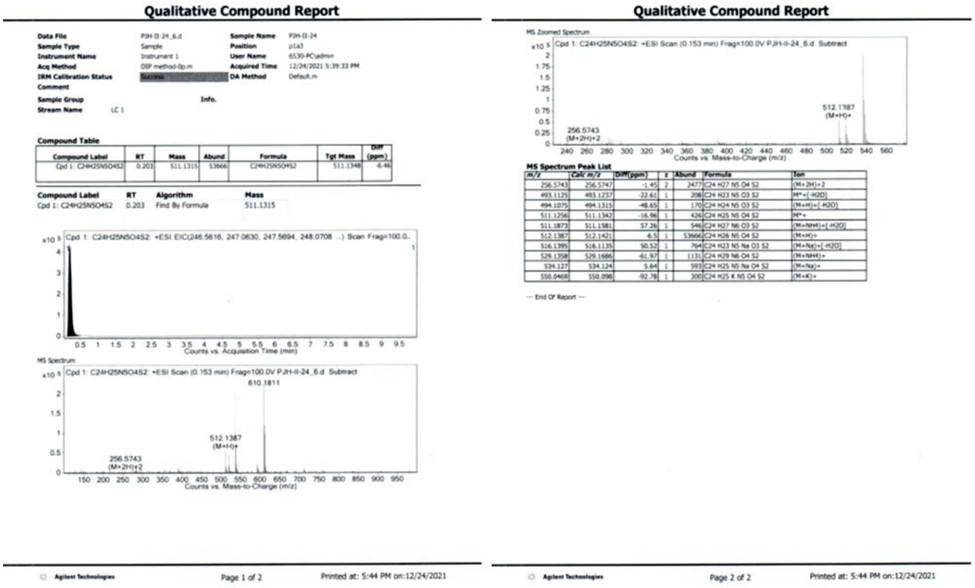

FigureS79. MS spectra of compound **19e**

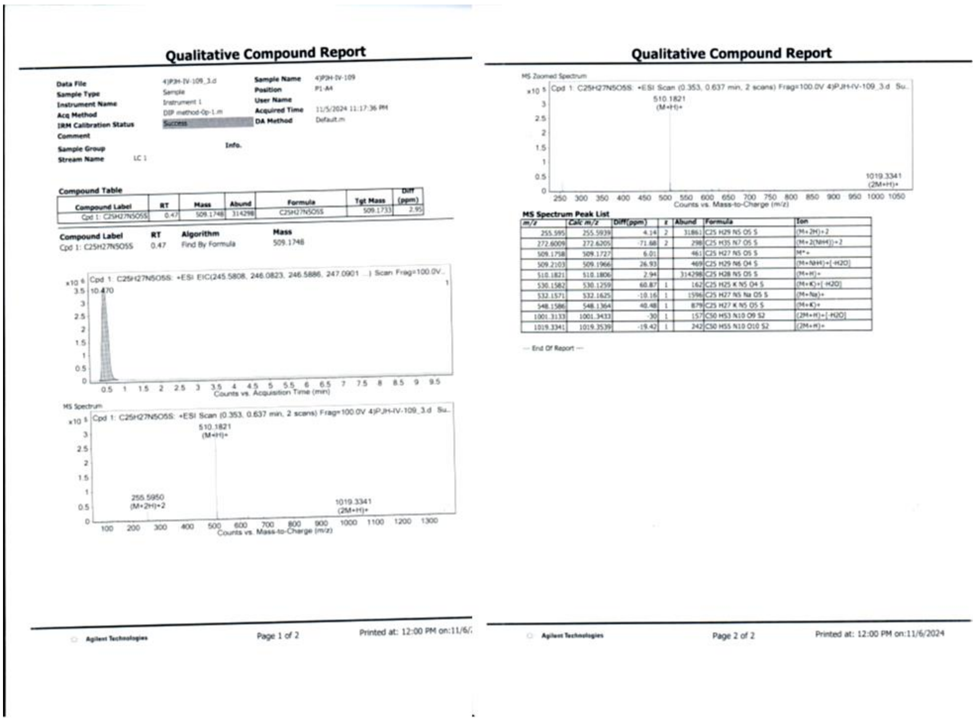

FigureS80. MS spectra of compound **21**

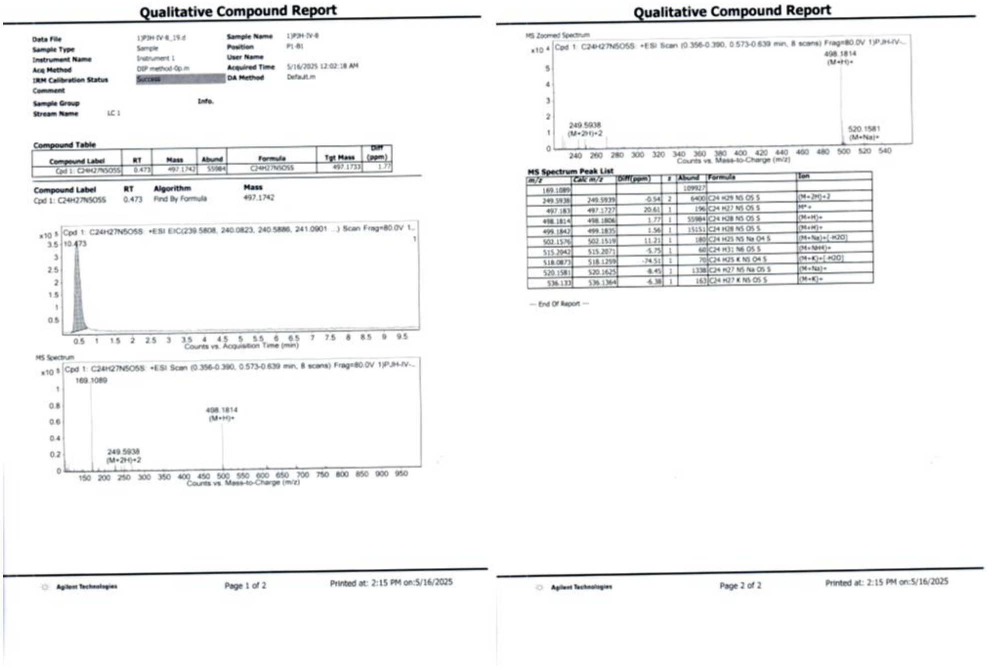

FigureS81. MS spectra of compound **22a**

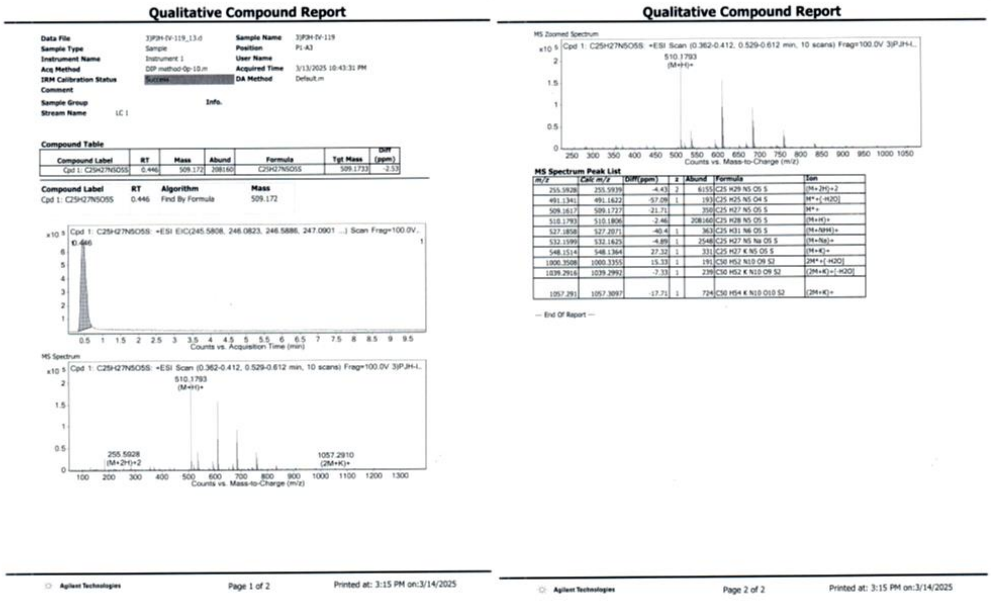

FigureS82. MS spectra of compound **22b**

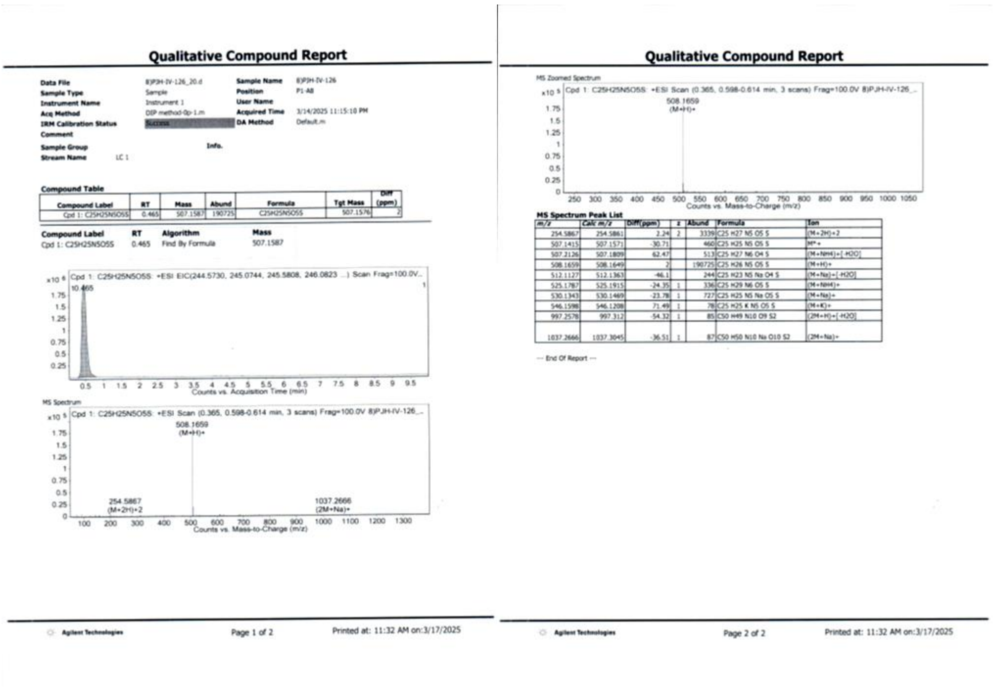

FigureS83. MS spectra of compound **22c**

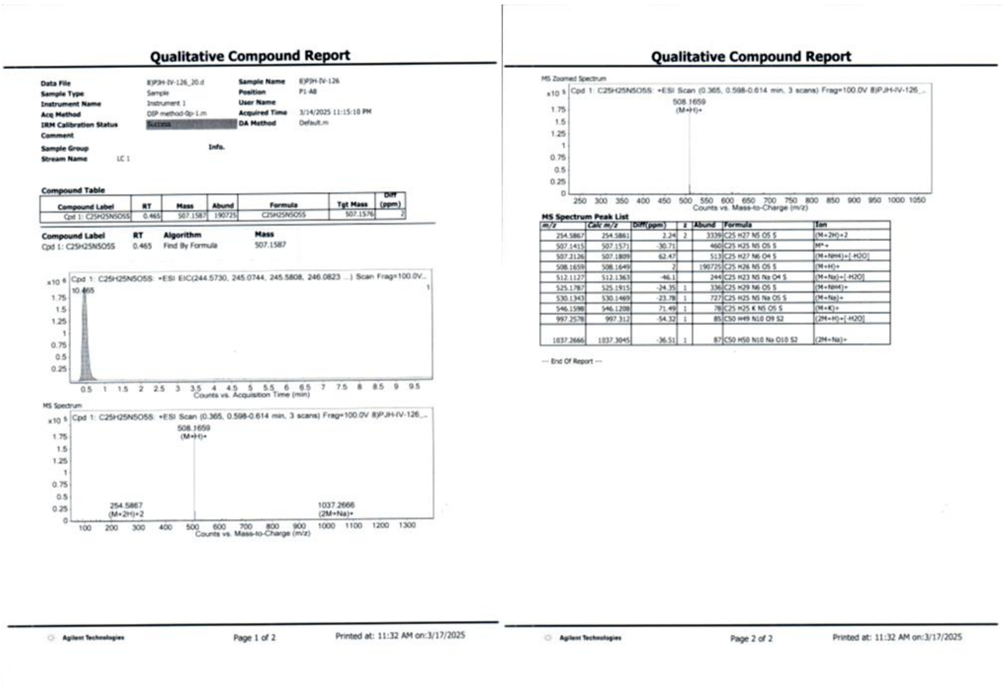

FigureS84. MS spectra of compound **22d**

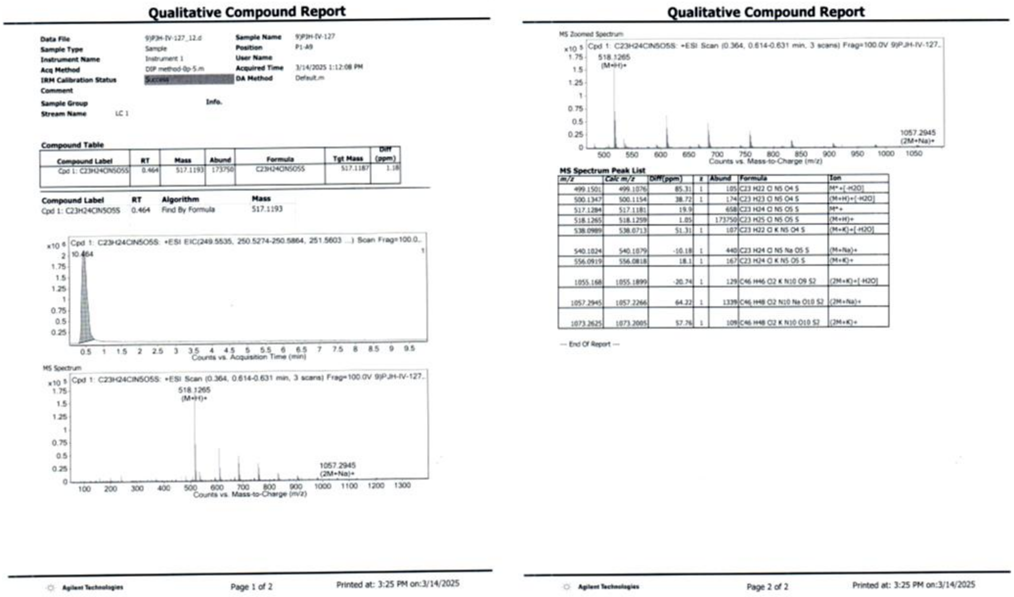

FigureS85. MS spectra of compound **22e**

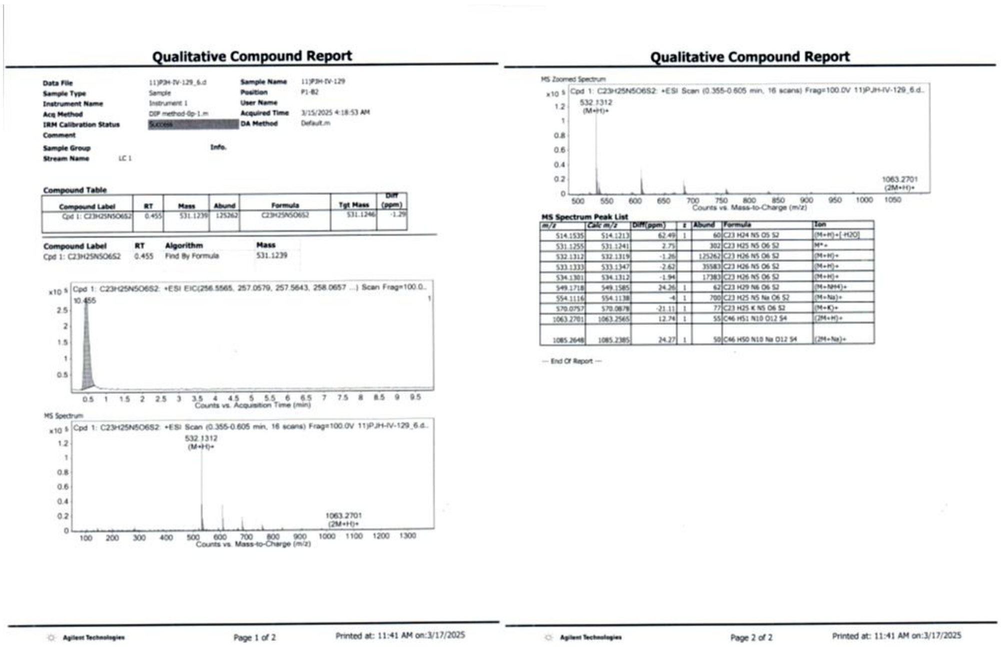

FigureS86. MS spectra of compound **22f**
